# High-altitude cerebral hypoxia promotes mitochondrial dysfunction and apoptosis of mouse neurons

**DOI:** 10.3389/fnmol.2023.1216947

**Published:** 2023-07-12

**Authors:** Yu Huan, Huilin Quan, Bo Jia, Guangzhi Hao, Zuolin Shi, Tianzi Zhao, Ying Yuan, Fang Yuan, Yushu Dong, Guobiao Liang

**Affiliations:** ^1^Department of Neurosurgery, General Hospital of Northern Theater Command, Shenyang, China; ^2^Department of Orthopedics, Xijing Hospital, Air Force Medical University, Xi'an, China; ^3^Department of Neurosurgery, Xijing Hospital, Air Force Medical University, Xi’an, China

**Keywords:** mitochondrial fission, neurons, apoptosis, high-altitude hypoxia, necroptosis

## Abstract

**Introduction:**

Neuronal cell death is an important factor in the pathogenesis of acute high-altitude cerebral hypoxia; however, the underlying molecular mechanism remains unclear. In this study, we tested if high-altitude hypoxia (HAH) causes neuronal death and mitochondrial dysfunction using various *in vivo* and *in vitro* approaches.

**Methods:**

Acute high-altitude cerebral hypoxia was induced by hypobaric hypoxia chamber in male mice. we explored the mechanisms of neuronal cell death using immunofluorescence, western blotting, transmission electron microscopy, and flow cytometry. Next, mitochondrial function and morphology were observed using Jc-1 staining, seahorse assay, western blotting, MitoTracker staining, and transmission electron microscopy. Moreover, open field test, elevated plus test, and Morris water maze were applied for animal behavior.

**Results:**

Results revealed that HAH disrupted mitochondrial function and promoted neuronal apoptosis and necroptosis both in HT-22 cells and in mouse hippocampal neurons. Moreover, the mitochondrial membrane potential and adenosine triphosphate production decreased in neurons after HAH, while oxidative stress and mitochondrial fission increased. Behavioral studies suggested that HAH induced anxiety-like behavior and impaired spatial memory, while it had no effect on athletic ability.

**Discussion:**

These findings demonstrated that HAH promotes mitochondrial dysfunction and apoptosis of mouse neurons, thus providing new insights into the role of mitochondrial function and neuronal cell death in acute high-altitude cerebral hypoxia.

## Highlights

- High altitude exposure induced neuronal cell death including apoptosis and necroptosis.
- High altitude exposure resulted in mitochondrial dysfunction.
- Mitochondrial fission was activated after high altitude hypoxia *in vivo* and *in vitro*.
- High altitude exposure promoted intelligent and emotional behavioral abnormalities.

## 1. Introduction

Acute high-altitude hypoxia (HAH) referres to the syndrome when the people arrive at the altitudes ⩾2,500 m and exhibite headache, nausea, vomiting, and insomnia (West, 2012; Luks et al., 2017; Luks and Hackett, 2022). Some patients even die as a result of high-altitude cerebral edema (West, 2012; Luks and Hackett, 2022). With the broadening of human activities, an increasing number of people travel to or work at high altitudes, which puts forward a new challenge on human health. Neurological deficit is one of the main causes of acute HAH manifestations (Luks et al., 2017), but further mechanisms remain to be elucidated. And clarifying the mechanism is critical for providing novel therapeutic targets and treatment strategies for HAH.

It has been proposed that neuronal damages are the principal reason for the symptoms of central nervous system impairment in HAH (Luks et al., 2017). Recent studies reported that HAH for 24 h reduced the number of neurons in the cortex and hippocampus regions (Zhou et al., 2017), and the ultrastructure of neurons also gets changed (Cai et al., 2021). Further, the mechanism of neuronal damages in HAH include neuroinflammation (Jiang et al., 2022), oxidative stress (Xie et al., 2022), energy metabolism disorders (Wang et al., 2022), etc. Mitochondria are the powerhouse of cells and sensitive to oxygen deficieny. Among the pronounced effects on mitochondria, hypoxia compromises cellular energy availability, damages mitochondrial components, modulates mitochondrial mass and dynamics, and affects the regulation of mitochondrial cell death pathways (Mallet et al., 2023). However, the role of neuronal mitochondria in HAH remain unclear. Whether mitochondrial abnormalities in morphology and function are associated with neuronal damages needed further investigation.

**Figure 1:**
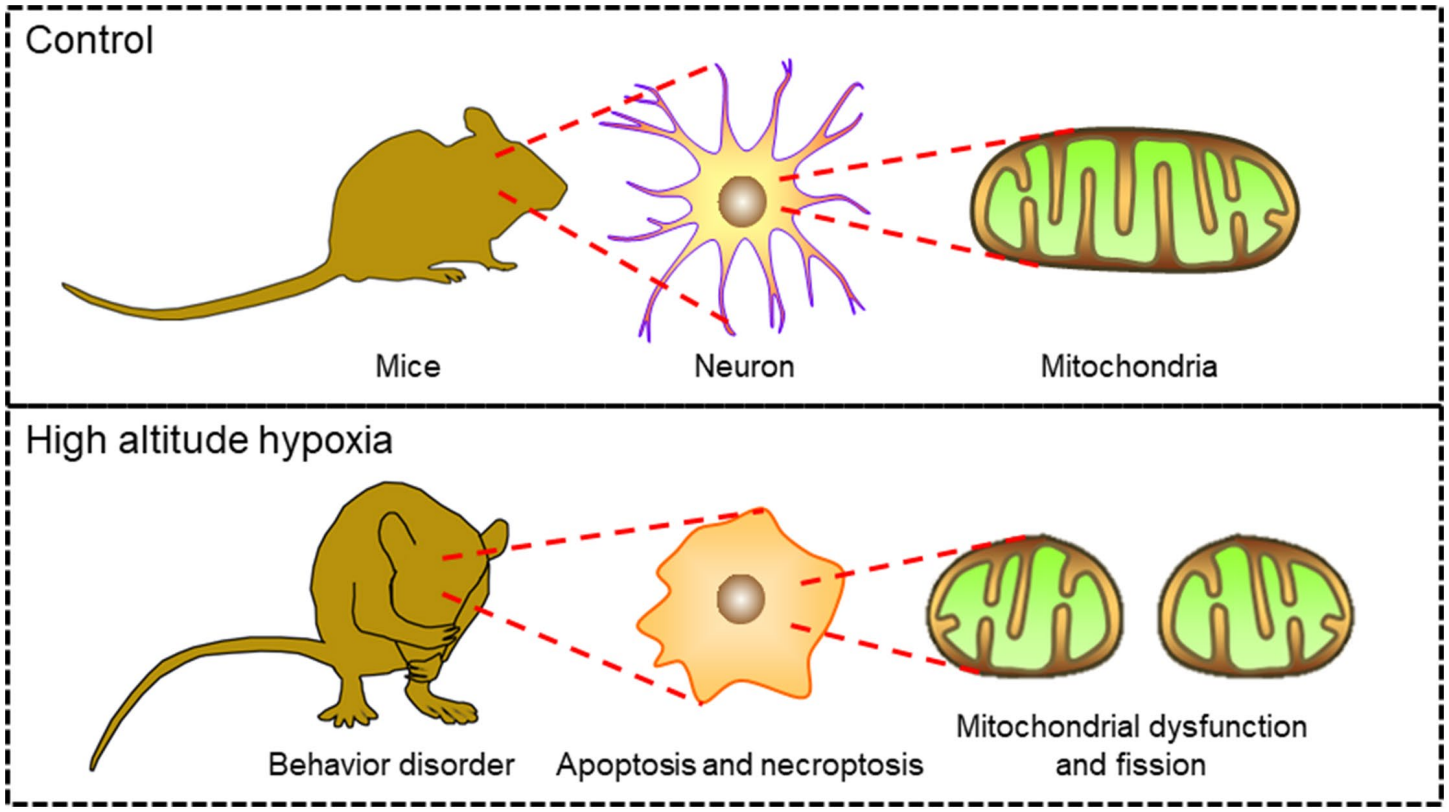
Mitochondrial dysfunction and neuronal cell death in high altitude cerebral hypoxia.

In this study, *in vivo* and *in vitro* HAH models were established to further investigate the mechanism of neuronal damages. Programmed cell death is considered as the main reason of hypoxic diseases (Imray et al., 2010; West, 2012; Mallet et al., 2023). Here, we identified the subtypes of neuronal cell death in both *n* vivo and *in vitro* HAH models. At subcellular level, mitochondrial length decreased and fragmented mitochondria increased in HAH group, which indicated that HAH promoted mitochondrial fission. In addition, mitochondrial function was also effected by HAH. In short, we found that mitochondrial dysfunction and fission promoted HAH-induced behavioral abnormalities via neuronal apoptosis and necroptosis, which gave the necessary theoretical basis for the treatment of high altitude cerebral hypoxia by mitochondrial protection (Figure 1).

In compare with control group, neuronal apoptosis and necroptosis increased after HAH. At the subcellular level, HAH promoted mitochondrial dysfunction and fission of neurons.

## 2. Materials and methods

### 2.1. Experimental animals

All animal procedures were performed in accordance with the Guide for the Care and Use of Laboratory Animals published by the US National Institutes of Health (NIH Publication No. 85-23, revised 1996) and approved by the Animal Ethics Committee of Shenyang General Hospital. Male mice (8 weeks old) were used for *in vivo* experiments. The animals were maintained under specific pathogen-free conditions.

### 2.2. Hypobaric hypoxia exposure and mouse treatment

A commercial hypobaric hypoxia chamber (LAT-SY01, Suzhou Liante Medical Equipment Co., Ltd., China) was used. The chamber pressure was maintained at 35.9 kPa, while oxygen concentration stayed at 20.9%, to imitate the barometric pressure conditions at 8000 m altitude. Both the ascent and descent rates of the hypobaric hypoxia chamber were 91.3 Pa/s. Mice were fed in this chamber for 3 days and sustained normal circadian rhythms.

### 2.3. Cell culture and treatment

HT-22 cells (Institute of Biochemistry and Cell Biology, SIBS, CAS, China) were grown in DMEM (11,965,092, Gibco, United States) plus 10% fetal bovine serum (10,091, Gibco, United States) and 1% penicillin–streptomycin (15,140,122, Sigma-Aldrich, United States). To establish the neuronal hypoxia model, HT-22 cells were placed in a hypoxic (1% O_2_, 5% CO_2_, 37°C) incubator for 24 h.

### 2.4. Immunohistochemistry

Animals were euthanizedand perfused intracardially with 4% cold paraformaldehyde in phosphate buffer (pH 7.4), followed by cryoprotection with 25% sucrose. For each mouse, serial sections were cut to 14 mm in thickness for each section. For immunostaining, the sections were blocked in 0.01 M phosphate-buffered saline (PBS) containing 0.3% Triton X-100 and 3% bovine serum albumin for 1 h. After overnight incubation with primary antibodies followed by washing with PBS, sections were incubated with the corresponding secondary antibodies conjugated with Alexa Fluor 594 or Alexa Fluor 488 for 2–4 h at room temperature, while protected from light. The nuclei were counterstained with 4′,6-Diamidino-2′-phenylindole (DAPI,1:5,000, Sigma-Aldrich, United States). All immunostained sections were imaged using a fluorescence microscope (FV10i, Olympus, Japan).

### 2.5. F-JB staining

. The tissues were sectioned with a freezing sliding microtome at a thickness of 25 μm. The slides were first immersed in a solution containing 1% sodium hydroxide in 80% alcohol for 5 min, followed by 2 min in 70% alcohol and 2 min in distilled water. After incubation in 0.06% potassium permanganate for 10 min, the slides were immersed in 0.0004% F-JB staining solution (AG310, Millipore, Burlington, United States) for 20 min. All immunostained sections were imaged using a fluorescence microscope (FV10i, Olympus, Japan) with the same settings.

### 2.6. Annexin V/propidium iodide staining

After treatment, HT-22 cells were collected. The annexin V/PI staining assay was followed as specified in the kit instructions (40302ES50, Yeasen, China). A 100 μL aliquot of 1 × Binding Buffer was mixed with 5 μL of annexin V and 10 μL PI solution and incubated with the cells for 10 min. After the addition of 400 μL 1 × Binding Buffer, the samples were subjected to flow cytometry analysis.

### 2.7. Western blotting

Total cellular proteins were prepared by direct lysis of cells or tissues using RIPA buffer (R0278, Sigma-Aldrich, United States). A total of 30 μg of total proteins was separated by 10–15% sodium dodecyl sulfate-polyacrylamide gel electrophoresis and blotted on polyvinyl difluoride membranes. The blots were blocked with PBS (pH 7.4) containing 10% serum for 1 h and then incubated overnight at 4°C with primary antibodies. After washing with PBS, the membranes were incubated with HRP-conjugated secondary antibodies at a dilution of 1:3000 for 2 h. At last, images were captured with a Molecular Imager System, and densitometry of the bands was quantified using ImageJ software.

### 2.8. TUNEL assays

Tissue slides and cells were reacted with terminal deoxynucleotidyl transferase enzyme and fluorescently-labeled 2′-deoxyuridine 5′-triphosphate from a TUNEL assay kit (40306ES50, Yeasen, China) at 37°C for 1 h. The nuclei were stained with DAPI. The number of TUNEL-positive cells was determined from fluorescence microscopy images.

### 2.9. Transmission electron microscopy

After treatment, fresh cortex and hippocampus regions of mice brain were fixed in 4% glutaraldehyde for 24 h at 4°C. The samples were then incubated with 1% osmium tetroxide, after which they were dehydrated with alcohol and embedded in Araldite®. Thin sections (85 nm) were stained with uranyl acetate and lead citrate. Ultrastructural analysis was conducted via TEM (FEI, Hillsboro, Oregon) at 80 kV.

### 2.10. Measurement of adenosine triphosphate

ATP level was measured using an ATP Determination kit (S0027, Beyotime Biotechnology, China) according to the manufacturer’s protocol. Luminescence was recorded using a luminescence plate reader.

### 2.11. Measurement of reactive oxygen species

2,7-Dichlorodihydrofluorescein diacetate (DCFH-DA, 50101ES01, Yeasen, China) is an indicator of intracellular ROS. Dihydroethidium (DHE, 50102ES25, Yeasen, China) is a specific fluorescent probe that detects superoxide anions. Briefly, samples were stained with DCFH-DA (10 μM) or DHE (10 μM) at 37°C for 30 min. After washing twice with PBS, the cells were analyzed by confocal microscopy (FV10i, Olympus, Japan).

### 2.12. Measurement of mitochondrial permeability transition pore opening

The mPTP opening rate was determined by loading cells with 5 μmol/L Calcein-AM (C2009S, Beyotime Biotechnology, China) and 2–5 mmol/L cobalt chloride. The cells were then analyzed by confocal microscopy, and the results were presented as the fluorescence intensity relative to the control.

### 2.13. Measurement of mitochondrial membrane potential

JC-1 (C2003S; Beyotime, China) can form red-fluorescent aggregates in normal mitochondria, however, it is transformed into green-fluorescent monomers in depolarized mitochondria. Here, samples were stained with 2 μg/mL JC-1 for 30 min at 37°C. The cells were then analyzed by confocal microscopy, and the ratio of red/green fluorescence was used as a measure of MMP alteration.

### 2.14. Oxygen consumption analysis

Cells were plated at 0.5 × 10^6^ cells/well in a 24-well Seahorse plate 1 day prior to measurement. The plate was incubated in glucose-supplemented Seahorse XF assay medium at 37°C for 1 h without CO_2_. The oxygen consumption rate (OCR) was measured with oligomycin (1 μM), carbonyl cyanide-4-(trifluoromethoxy) phenylhydrazone (FCCP, 1 μM), and rotenone (0.5 μM). The plates were processed on a Seahorse XF-24 analyzer.

### 2.15. Mitochondrial morphology

MitoTracker™ Red (M7512, Invitrogen, China) is a Δψm-dependent dye that can monitor mitochondria. After treatment, cells were stained with MitoTracker™ Red (100 nM) and used to observe mitochondrial morphology by laser scanning confocal microscopy (LSCM, Olympus, Japan). The mitochondrial mass and length were analyzed using ImageJ software.

### 2.16. Behavior assays

#### 2.16.1. Open field test

The open field test was carried out in a white opaque plastic chamber (50 × 50 × 35 cm), divided into 25 squares with the same area. The central nine squares were defined as the central area, while the remainder were considered as peripheral area. For each test, a mouse was gently placed in one corner, and the movement was recorded for 5 min with a video tracking system. The time spent and distance traveled in the central area, and the total distance traveled in the field were measured using SMART software (SMART 3.0, Panlab, United States). Between each test, 75% ethanol was used to clean the open field area.

#### 2.16.2. Elevated plus maze test

The maze was placed 50 cm above the floor and consisted of two open and two closed arms (30 × 5 cm, and a 15 cm wall height for the closed arms). Each mouse was placed on the central area, heading toward the same open arm, and videotaped in the following 5 min. The time spent and moving distance in the open arms, as well as the total movements in both the arms, were analyzed using SMART 3.0 software. The maze was cleaned with 75% ethanol between tests.

#### 2.16.3. Morris water maze test

The mice were trained to find a hidden platform (10 cm in diameter) in a white plastic tank containing water (120 cm in diameter). The mice were subjected to four trials per day, with an intertrial interval of 30 min. For four consecutive days, the learning phase of the water maze test was performed, followed by the probe test on day five. During the probe test, the mice were allowed 1 min to find the removed platform. The target quadrant occupancy and exact number of crossings over the former platform location during the probe test were measured using the EthoVision® XT 10 program (Noldus, Netherlands).

### 2.17. Statistical analysis

The above experiments were repeated independently at least three times, and the results are presented as means ± the standard error of the mean (SEM). Student’s two-tailed *t*-test was used to compare differences between two groups. Differences were analyzed using GraphPad Prism 8.0 software and considered statistically significant at a threshold of *p* < 0.05.

## 3. Results

### 3.1. HAH promotes apoptosis and necroptosis of neurons

**Figure 2:**
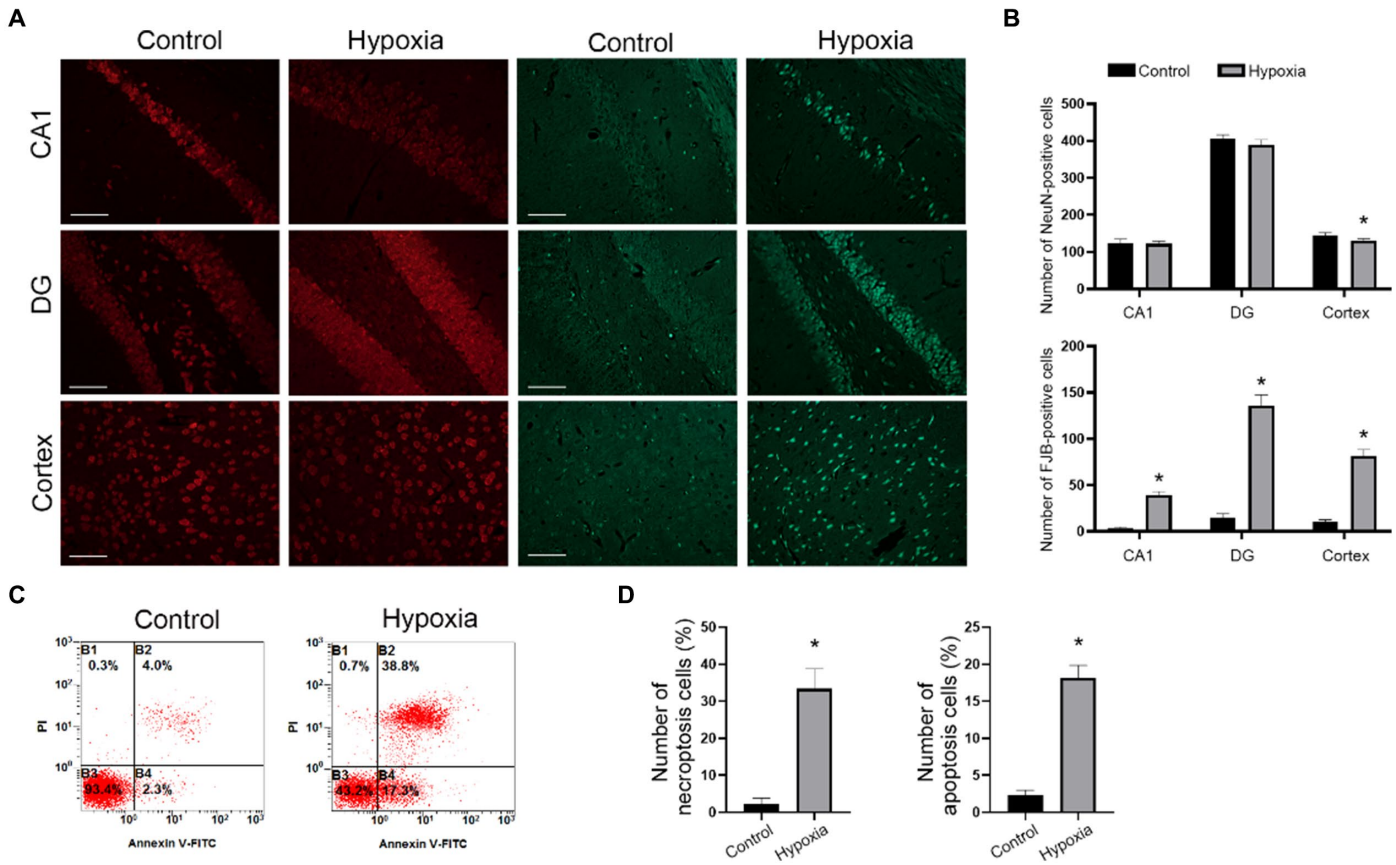
HAH promotes neuronal cell death. **(A)** Representative image of NeuN (red, marker of neurons) and F-JB (green, marker of degenerative neurons) staining in cortex, hippocampus CA1 and DG region of control and hypoxia group; scale bars, 20 μm. **(B)** The numbers of NeuN -positive and F-JB -positive cells were recorded. **(C)** Annexin-V/PI staining was analyzed by flow cytometry. **(D)** The numbers of Annexin-V+/PI+ (necroptosis) and Annexin-V+/PI- (apoptosis) cells were recorded. The data shown are presented as the mean ± SEM (*n* = 3). *p < 0.05.

Cytotoxicity and vascular injury were the main pathophysiology of HAH. To further investigate the condition of neuronal cell death, we first conducted NeuN and F-JB staining in control and hypoxia mice. The results of the NeuN staining showed that there was no difference in the hippocampus CA1 and DG regions of the control and hypoxia groups, and hypoxia decreased the number of neurons in the cortex (Figures 2A,B). In addition, hypoxia increased the number of degenerative neurons (F-JB-positive cells) in the cortex and hippocampus CA1 and DG regions (Figures 2A,B). Next, *in vitro* HT-22 cell hypoxia model was established. Annexin V/PI staining showed that the numbers of annexin V+/PI+ and annexin V+/PI-cells both decreased in the hypoxia group (Figures 2C,D), demonstrating that hypoxia promoted neuronal cell death via apoptosis and necroptosis. In brief, *in vivo* and *in vitro* results confirmed that HAH promoted neuronal cell death.

**Figure 3:**
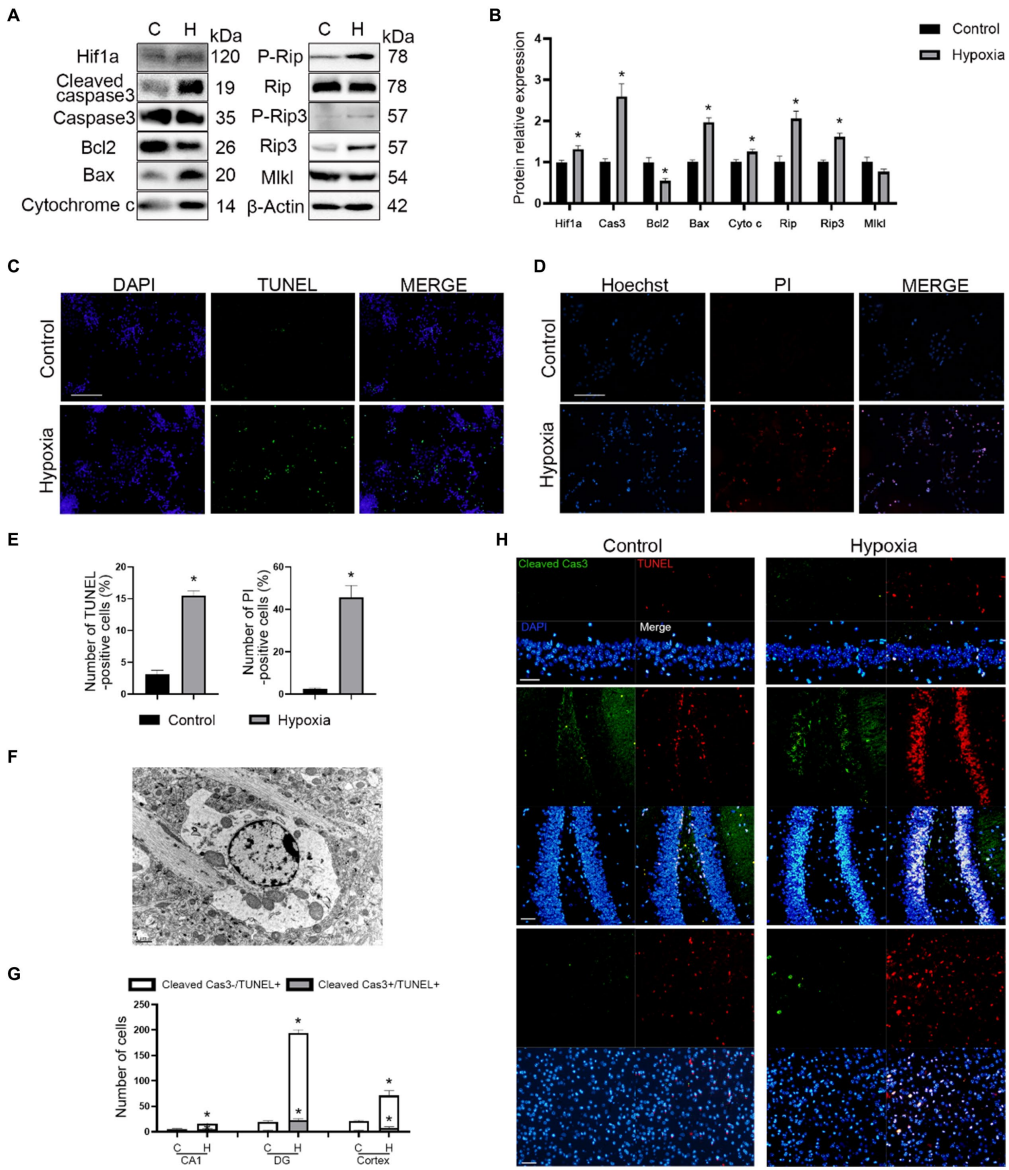
HAH promotes apoptosis and necroptosis of neurons. **(A)** Western blot detection of mitochondrial apoptosis-and necroptosis-related markers in neuronal samples from H (hypoxic group) and C (control group). **(B)** The expression of mitochondrial apoptosis-and necroptosis-related protein was recorded. **(C)** Representative images of TUNEL (green) staining; scale bars, 100 μm. **(D)** Representative images of Hoechst (blue) and PI (red) staining; scale bars, 100 μm. **(E)** The numbers of TUNEL -positive and PI-positive cells were recorded. **(F)** Representative ultrastructural images of programmed cell death of hypoxic mice; scale bars, 1 um. **(G)** The numbers of Cleaved Cas3-/TUNEL+ (necroptosis) and Cleaved Cas3+/TUNEL+ (apoptosis) cells were recorded. **(H)** Representative images of TUNEL (red) and Cleaved Cas3 (green) staining in cortex, hippocampus CA1 and DG region of normoxic and hypoxic mice; scale bars, 50 μm. The data shown are presented as the mean ± SEM (*n* = 3). *p < 0.05.

To further confirm whether both apoptosis and necroptosis participated in this process, we assayed the expression of apoptosis- (Caspase3, Bcl2, Bax, and Cytochrome c) and necroptosis (Rip, Rip3, and Mlkl)-related proteins. The results of western blotting demonstrated that hypoxia promoted apoptosis and necroptosis of neurons *in vitro* (Figures 3A,B). Moreover, TUNEL and Hoechst-PI staining confirmed this phenomenon (Figures 3C–E). Samples of *in vivo* brain tissue after HAH were collected and imaged by TEM. In neurons of hypoxic mice, chromatin aggregated on the nuclear membrane, and the cytoplasmic content was reduced (Figure 3F), indicating that HAH promoted neuronal cell death. Mitochondrial swelling was also observed in neurons of hypoxic mice (Figure 3F), demonstrating that mitochondria could be involved in HAH. The number of cleaved Cas3-/TUNEL+ (necroptosis) and cleaved Cas3+/TUNEL+ (apoptosis) cells increased in the cortex and hippocampus CA1 and DG regions of hypoxic mice (Figures 3G,H).

### 3.2. HAH promotes neuronal mitochondrial dysfunction

**Figure 4:**
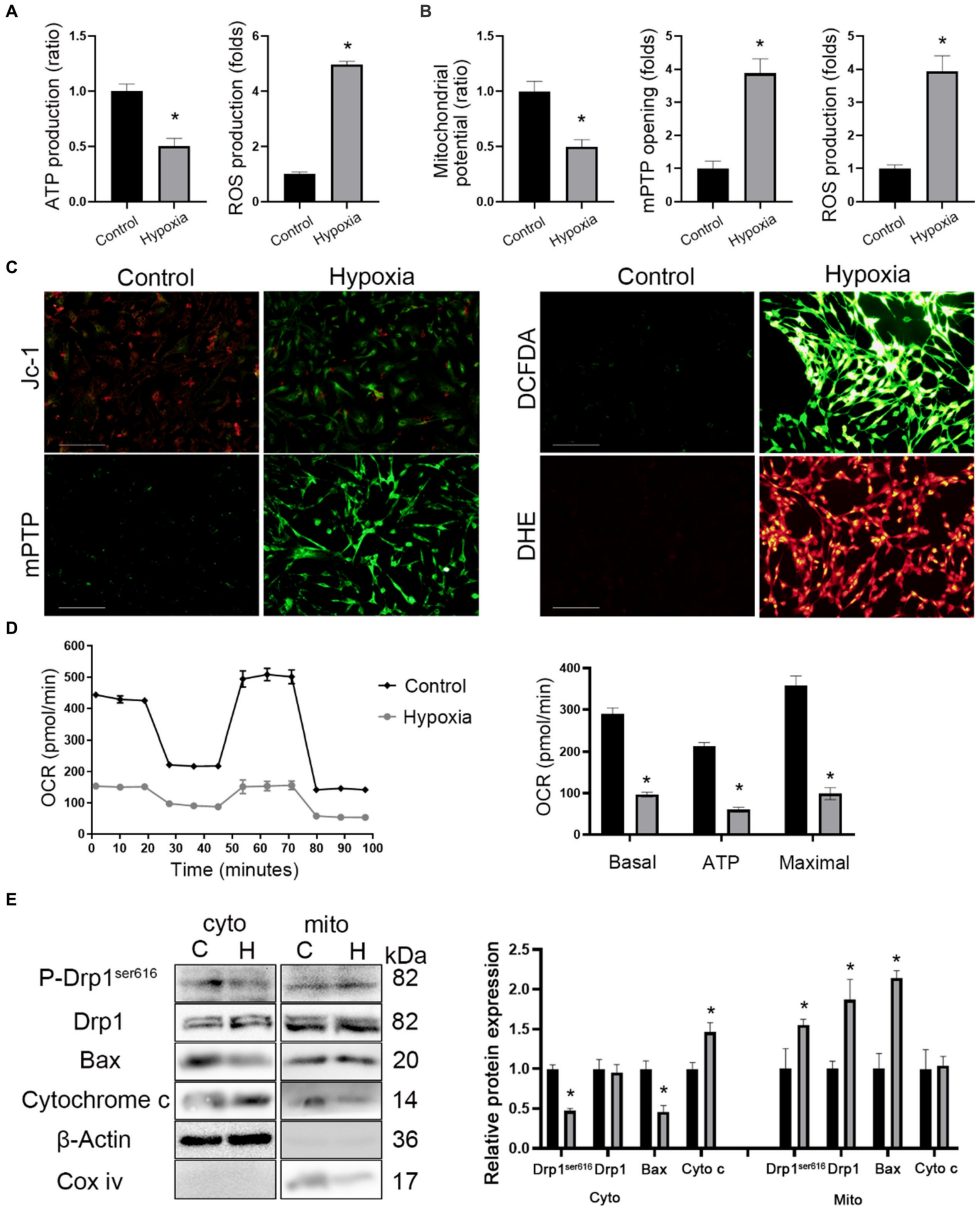
HAH promotes mitochondrial dysfunction of neurons. **(A)** Measurements of ATP and ROS production in hippocampus of normoxic and hypoxic mice. **(B)** Mitochondrial potential, mPTP opening and ROS production were recorded. **(C)** Representative images of HT-22 cells loaded with the mitochondrial membrane potential indicator JC-1, mitochondrial permeability transition pore indicator mPTP, ROS indicator DCFDA; scale bars, 50 μm. **(D)** Oxygen consumption rate (OCR) tested by Seahorse assay. Quantitative levels of basal respiration, maximal respiration and ATP production. **(E)** Cytosolic (cyto) and mitochondrial (mito) fractions were prepared from neurons. The fractions were subjected to western blot analysis using the indicated antibodies. COX IV and β-Actin were examined as mitochondrial and cytosolic marker proteins. The data shown are presented as the mean ± SEM (*n* = 3). *p < 0.05.

The ultrastructural images showed the presence of mitochondrial swelling in hypoxic mice (Figure 3F). Hence, mitochondrial functions such as the membrane potential, oxidative phosphorylation, mPTP opening, and oxidative stress was measured. Firstly, it was found that ATP production decreased while ROS production increased in brain tissue of hypoxic mice (Figure 4A). Next, *in vitro* mitochondrial function in HT-22 cells was assessed, which showed that the mitochondrial potential decreased in hypoxic cells, while mPTP opening and ROS production increased (Figures 4B,C). Seahorse assay was the gold standard for evaluating mitochondrial function. After 24 h of hypoxia, the oxidative phosphorylation level of HT-22 cells was reduced, which was characterized by a reduction in the OCR and ATP production (Figure 4D). The results indicated that apoptosis occurred in neurons after HAH. However, it was unclear whether the mitochondrial apoptosis pathway was activated. Therefore, mitochondria were isolated, and the expression of mitochondrial apoptosis-related proteins was assayed. The results showed that cytoplasmic Cytochrome c and mitochondrial Bax levels increased in hypoxic HT-22 cells (Figure 4E), demonstrating that the mitochondrial apoptosis pathway was activated in hypoxic neurons. In addition, mitochondrial P-Drp1^ser616^ and Drp1 levels were increased in hypoxic HT-22 cells (Figure 4E), suggesting that hypoxia could promote mitochondrial fission in neurons.

### 3.3. HAH promotes mitochondrial fission in neurons

**Figure 5:**
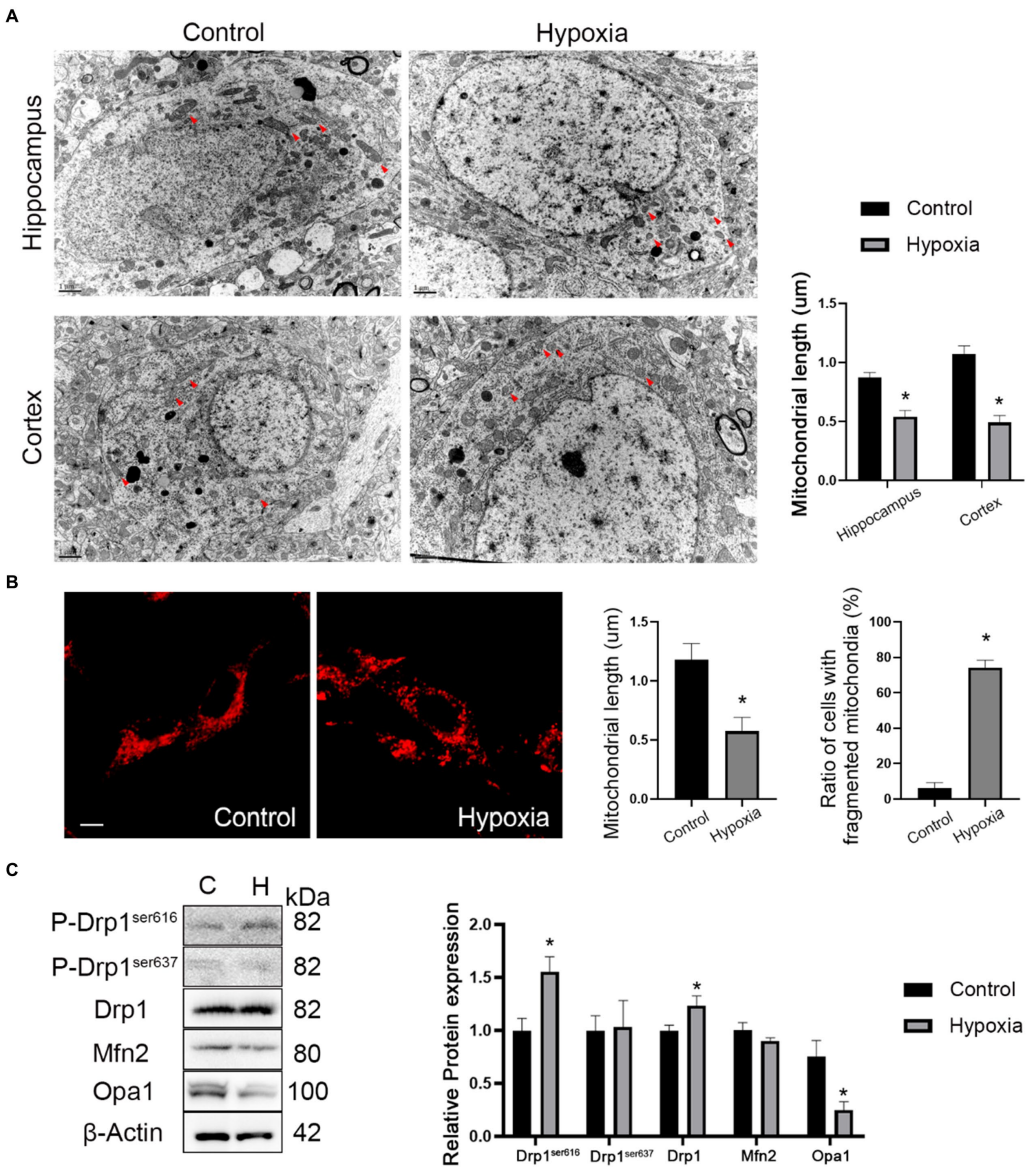
HAH promotes mitochondrial fission of neurons. **(A)** Ultrastructural images of mitochondria in hippocampus and cortex of normoxia and hypoxia mice captured by TEM. Representative images of mitochondria are indicated (Red arrows); scale bars, 1 um. **(B)** Representative images of MitoTracker-Red stain of normoxic and hypoxic HT-22 cells; scale bars, 10 μm. The average length of mitochondria and the ratio of fragmented mitochondria were recorded. **(C)** Western blot detection of mitochondrial dynamic-related proteins. The data shown are presented as the mean ± SEM (*n* = 3). *p < 0.05.

The mitochondrial morphology of *in vivo* neurons in cortex and hippocampus regions was observed by TEM. The ultrastructural images showed that the mitochondrial length was reduced in hypoxic neurons of the cortex and hippocampus regions (Figure 5A). *In vitro* mitochondrial morphology was assessed by MitoTracker™ staining and indicated that the mitochondrial length decreased in hypoxic HT-22 cells while fragmented mitochondria increased (Figure 5B). In short, *in vivo* and *in vitro* mitochondrial morphologies showed that HAH promoted mitochondrial fission in neurons. Subsequently, the expression of mitochondrial dynamics-related proteins was assayed by western blotting. The results showed that the expression of P-Drp1^ser616^ and Drp1 (markers of mitochondrial fission) increased in hypoxic HT-22 cells, while the expression of Opa1 (a marker of mitochondrial fusion) decreased (Figure 5C).

### 3.4. HAH induces anxiety-like behavior and impairs spatial memory

**Figure 6:**
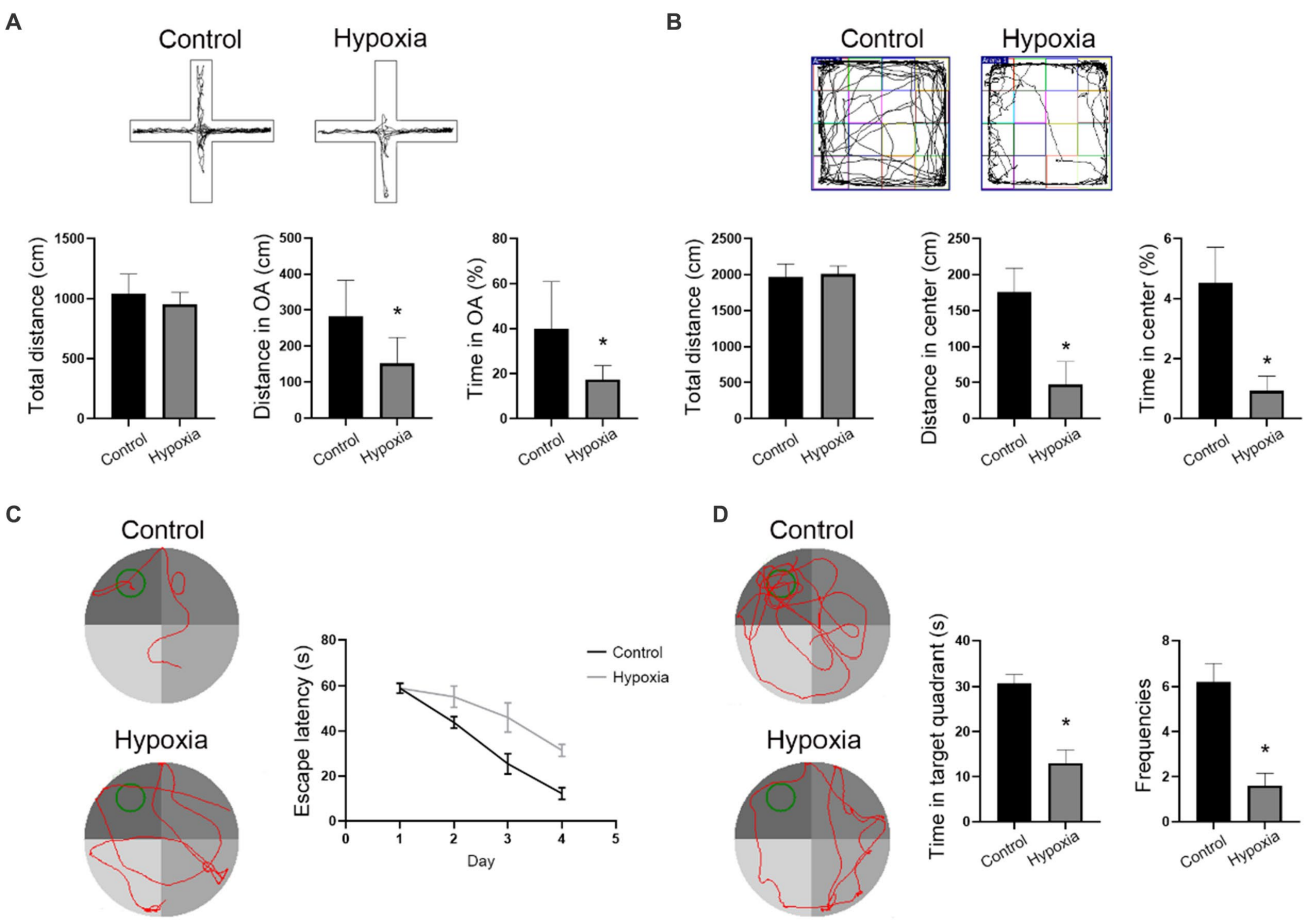
HAH induces anxiety-like behavior and impairs spatial memory. **(A)** Open field test. **(B)** Elevated plus test. **(C,D)** Morris water maze. The data shown are presented as the mean ± SEM (*n* = 3). *p < 0.05.

Hypoxia reportedly causes neuronal cell death in cortex and hippocampus regions, which is associated with brain function including spatial navigation, memory, and emotion. Open field and elevated plus maze tests showed that the hypoxic mice stayed less in the central area or open arm, suggesting that hypoxia could result in anxiety and depression (Figures 6A,B). Moreover, there was no significant difference in the total distance covered by the two groups (Figures 6A,B), suggesting that hypoxia does not influence athletic ability. In the navigation tests of Morris water maze, hypoxic mice spent more time finding the platform (Figure 6C). After removing the platform,time spent in the target quadrant and crossing frequencies were reduced in the hypoxic mice (Figure 6D). The results of the Morris water maze showed that HAH induced intelligential disorder. Briefly, HAH induced behavioral abnormalities.

## 4. Discussion

This study provides novel evidence of mitochondrial dysfunction and fission in high altitude cerebral hypoxia. Firstly, the results implicate that neuronal cell death, especially necroptosis, was involved in high attitude cerebral hypoxia, which provided a new clue to protest against HAH. Subsequently, mitochondria disorders in morphology and function were also fund in HAH. The results of MitoTracker™ Red staining, TEM and western blot confirmed that HAH promoted mitochondrial fission and Drp1 expression. Finally, behavioral abnormalities in cognition and emotion were verified in mice suffering HAH. In brief, mitochondrial dysfunction and neuronal cell death were involved in high-altitude cerebral hypoxia, providing critical new insights into the mechanism and treatment of HAH.

Several studies have found that hypobaric hypoxia at high altitudes could change the structure and function of human brain (Zhou et al., 2017; Zegarra-Rodriguez et al., 2022; Mallet et al., 2023). A retrospective, cross-sectional study revealed that people living at high altitudes had a higher incidence of depression than those living at low altitudes, suggesting that there was a statistically significant association between high altitude and depressive symptoms (Zegarra-Rodriguez et al., 2022). Additionally, functional magnetic resonance imaging results showed that the emotional changes at high altitudes, including depression, fatigue, and energy levels, were related to connectivity between the limbic system (Dalong et al., 2022). In this study, the open field test and elevated plus maze also found that mice in HAH group showed anxiety-like behavior. And neuronal cell death occurred in cortex and hippocampus region after HAH, which confirmed the conclusion that hypobaric hypoxia at high altitudes could change the structure and function of brain. In addition, cognitive functions, especially attention, inhibitory control, and working memory function, were also affected by low atmospheric pressure and hypoxia, which was associated with effects on the parietal occipital lobe, anterior cingulate cortex, prefrontal lobe, temporal lobe, and other brain regions (Li and Wang, 2022). From a pathophysiological point of view, potential mechanisms of high-altitude cerebral hypoxia include brain edema as well as increased brain volume and intracranial pressure (Imray et al., 2010). Using imaging, subcortical edema and cortical atrophy (enlarged cerebral sulci) have been observed in people suffering from HAH (Zhang and Zhang, 2022), suggesting that reduction in neurons and glial cells were the reason for the physiological effects of high-altitude cerebral hypoxia. With further investigation in behavioral neuroscience, the role of more cerebral areas needed to be clarified in HAH, which may be helpful for the treatment and prevention of HAH.

Resting-state functional magnetic resonance imaging has revealed that acute HAH decreased the connection between the visual and motor cortex (Zhang et al., 2022), suggesting that the white matter was the target site of HAH. Moreover, immunofluorescence microscopic imaging has shown that neuronal apoptosis increases in the DG and CA1 regions of the hippocampus (Koester-Hegmann et al., 2018). The reduction in the number of neurons was consistent with the decrease in cortical thickness in magnetic resonance imaging, which interpreted the radiographic changes in patients with HAH. An increasing number of investigators have explored the molecular mechanism and potential therapeutic target of high-altitude cerebral hypoxia. HAH reportedly disturbs formaldehyde metabolism and induces formaldehyde accumulation, which might be the mechanism of neuronal injury (Wang et al., 2022). In addition, endoplasmic reticulum stress, oxidative stress, NMDA-mediated apoptosis, and ferroptosis also appear to be involved in HAH neuronal injury (Ji et al., 2021; Zhou et al., 2021; Wang et al., 2022). In this study, we investigated the mechanism of neuronal apoptosis in terms of subcellular structure, especially mitochondrial morphology, and found that the degeneration and cell death of neurons occurred after acute HAH. Both apoptosis and necroptosis were involved in this process. Necroptosis has been reported to participate in high-altitude pulmonary edema (Wang et al., 2022). After HAH, the expression of Rip, Rip3, and Mlkl (markers of necroptosis) increased in the lung tissues, indicating that necroptosis was the mechanism of high-altitude hypoxic injury (Wang et al., 2022). In our experiments, we found that HAH promoted the expression of necroptosis markers and other characteristics of necroptosis morphology, indicating that necroptosis could be a potential therapeutic target for high-altitude cerebral hypoxia.

As the cellular powerhouse, mitochondria are sensitive to hypoxia. Therefore, mitochondria are starting to become a key topic in HAH research. HAH reportedly promotes mitochondrial biogenesis,but ATP production and complex 1 activity are inhibited in neurons (Hota et al., 2012), indicating that HAH adversely impacts neuronal mitochondrial function. Moreover, mitochondria are an important organelle associated with oxidative stress. After HAH, the expression of oxidation products (MDA and GSSG) increases, while the expression of antioxidant products (SOD and GSH) decreases (Hou et al., 2019). In our study, the increased expression of Caspase3, Bax, and Cytochrome c suggested that the mitochondrial pathway of apoptosis participated in this process, which was consistent with other studies (Wang et al., 2019). With the development of microscopy, electron microscopy has been applied in studies of high-altitude cerebral hypoxia. Abnormal mitochondria, characterized by clear swelling of mitochondria, unclear or disrupted membranes, and reduced cristae, have been observed in the neurons of hippocampus regions after HAH (Zhang et al., 2018; Chen et al., 2022; Choudhary et al., 2022). However, the impact of HAH on the mitochondrial morphology of neurons was still unclear. We conducted *in vivo* and *in vitro* experiments and found that the mitochondrial length decreased and fragmented mitochondria increased in neurons in the HAH group, indicating that HAH promoted neuronal mitochondrial fission. The expression of mitochondrial dynamics-related proteins supports this conclusion. In addition, HAH adversely impacted mitochondrial functions, including the membrane potential, oxidative phosphorylation, etc.

In conclusion, we found that mitochondrial dysfunction and neuronal cell death were the potential cellular mechanisms underlying acute high-altitude cerebral hypoxia. However, this study had several limitations. Firstly, as an observational study, this study lacked an interventional approach. Moreover, the roles of mitochondrial fission and Drp1 in neuronal cell death after HAH need to befurther investigated. Secondly, although necroptosis was found to be involved in HAH neuronal cell death, necroptosis inhibition was not applied in this study. Whether necroptosis could be a therapeutic target of HAH require further investigations. In this study, we presented evidence that necroptosis and mitochondrial fission were the potential mechanisms underlying acute high-altitude cerebral hypoxia, thus providing a novel perspective of HAH.

## Data availability statement

The original contributions presented in the study are included in the article/Supplementary material, further inquiries can be directed to the corresponding authors.

## Ethics statement

The animal study was reviewed and approved by Animal Ethics Committee of Shenyang General Hospital.

## Author contributions

YD, GL, and YH were involved in the conception and design of the study, performance of experiments, data analysis and interpretation, and manuscript writing. YH, HQ, and BJ were involved in development of the methodology. YH, GH, and ZS acquired the data. YH, HQ, BJ, TZ, YY, and FY analyzed and interpreted data. YD and GL obtained financial support, supervised the study and approved the final manuscript. All authors contributed to the article and approved the submitted version.

## Funding

The work was supported by Research Project of Shenyang Bureau of Science and Technology (20-205-4-017), National Natural Science Foundation of China (81971133 and 82071481), Liaoning Key Research and Development Project (2019JH8/10300085 and 2021JH2/10300059), and Liaoning Revitalization Talents Program (XLYC2002109).

## Conflict of interest

The authors declare that the research was conducted in the absence of any commercial or financial relationships that could be construed as a potential conflict of interest.

## Publisher’s note

All claims expressed in this article are solely those of the authors and do not necessarily represent those of their affiliated organizations, or those of the publisher, the editors and the reviewers. Any product that may be evaluated in this article, or claim that may be made by its manufacturer, is not guaranteed or endorsed by the publisher.
