# Supplementary figures and images for: High-altitude cerebral hypoxia promotes mitochondrial dysfunction and apoptosis of mouse neurons (part 1 of 2)
Source: Front Mol Neurosci. 2023 Jul 12;16:1216947. doi: 10.3389/fnmol.2023.1216947 (PMC10370763; doi:10.3389/fnmol.2023.1216947)

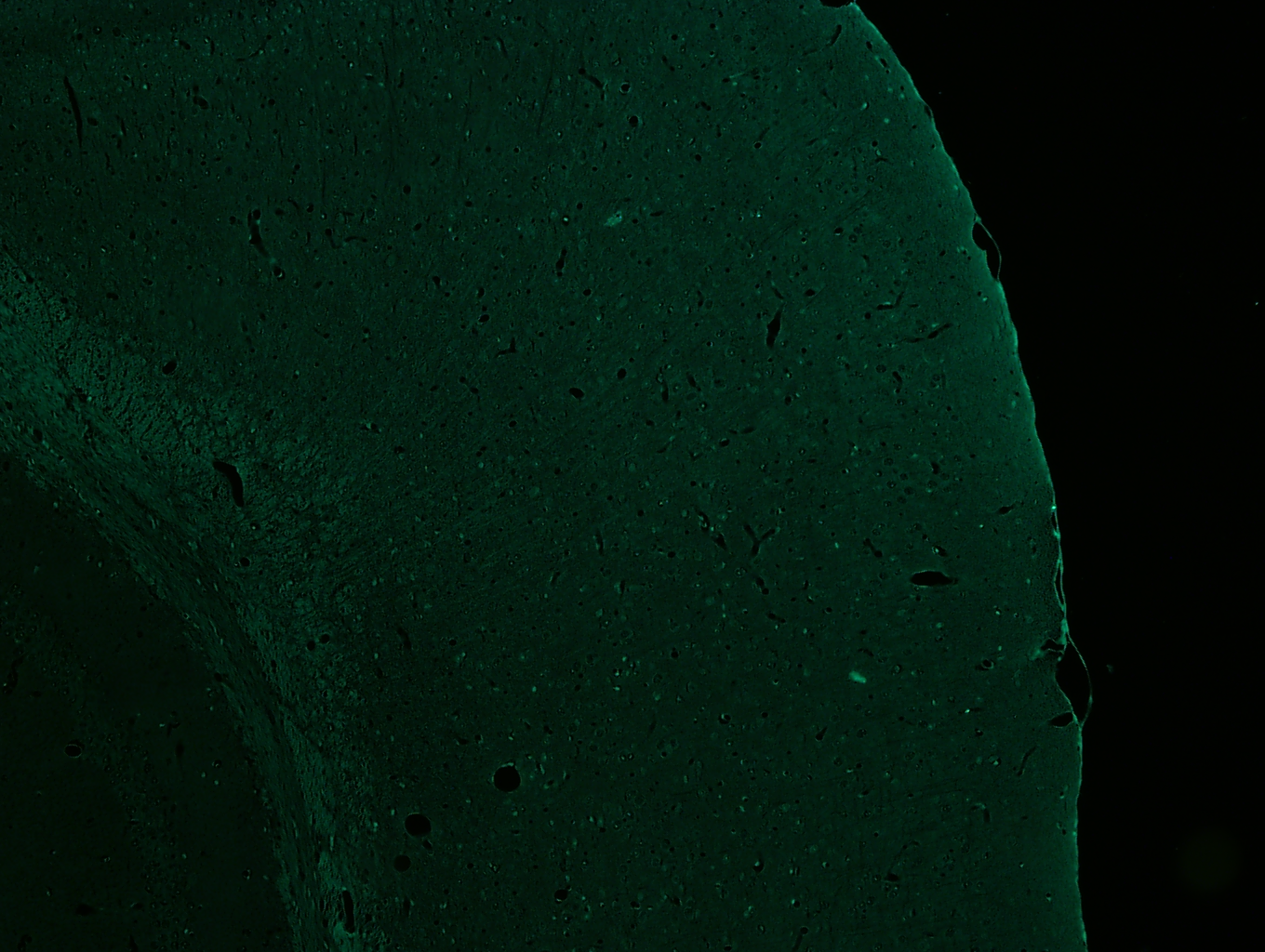

Supplement: Supplementary file 1 [file Data_Sheet_1.ZIP › control/cortex-10x.tif]

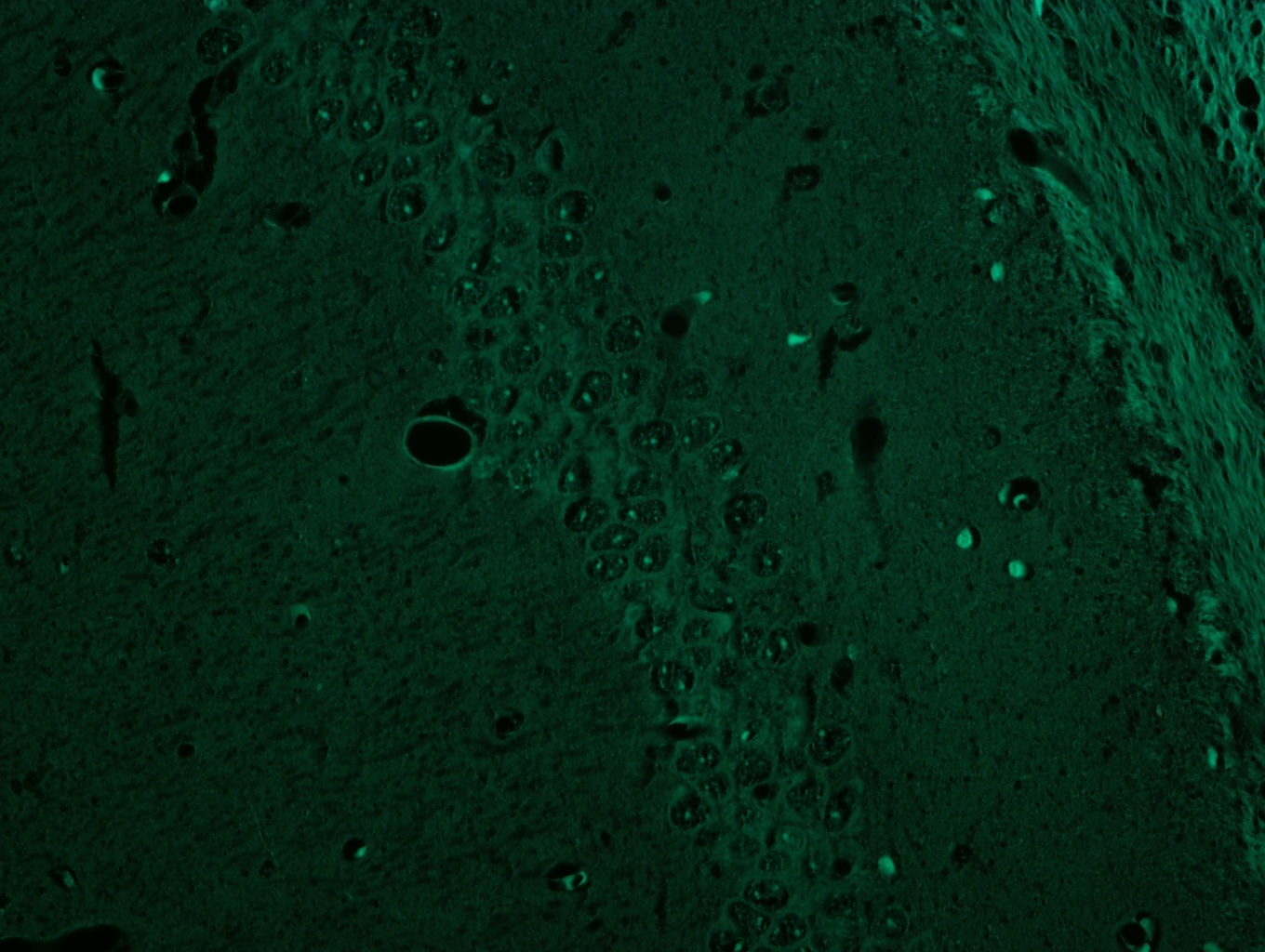

Supplement: Supplementary file 1 [file Data_Sheet_1.ZIP › control/hippo-CA1-40x.tif]

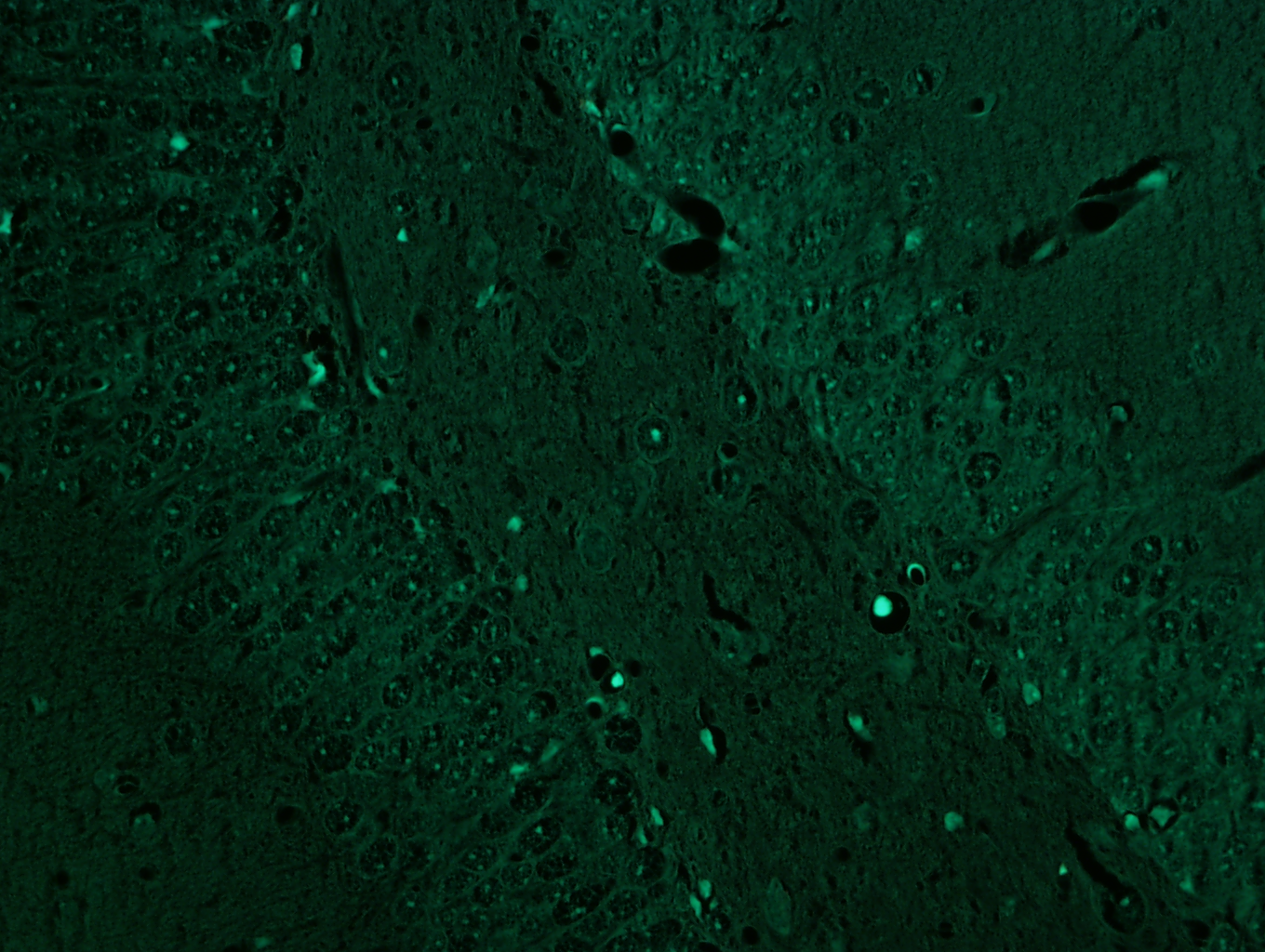

Supplement: Supplementary file 1 [file Data_Sheet_1.ZIP › control/hippo-DG-40x.tif]

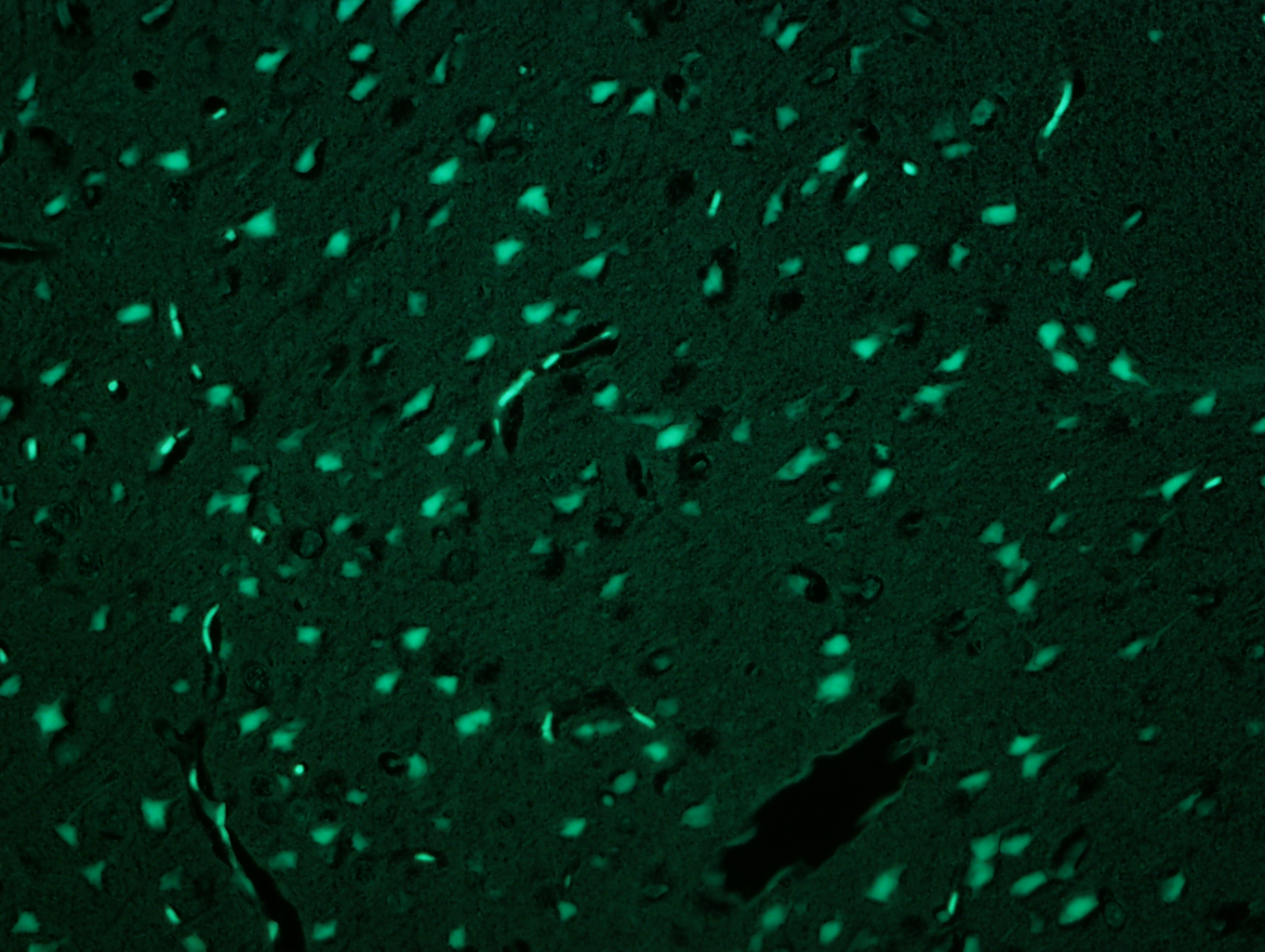

Supplement: Supplementary file 1 [file Data_Sheet_1.ZIP › hypoxia/cortex-40x.tif]

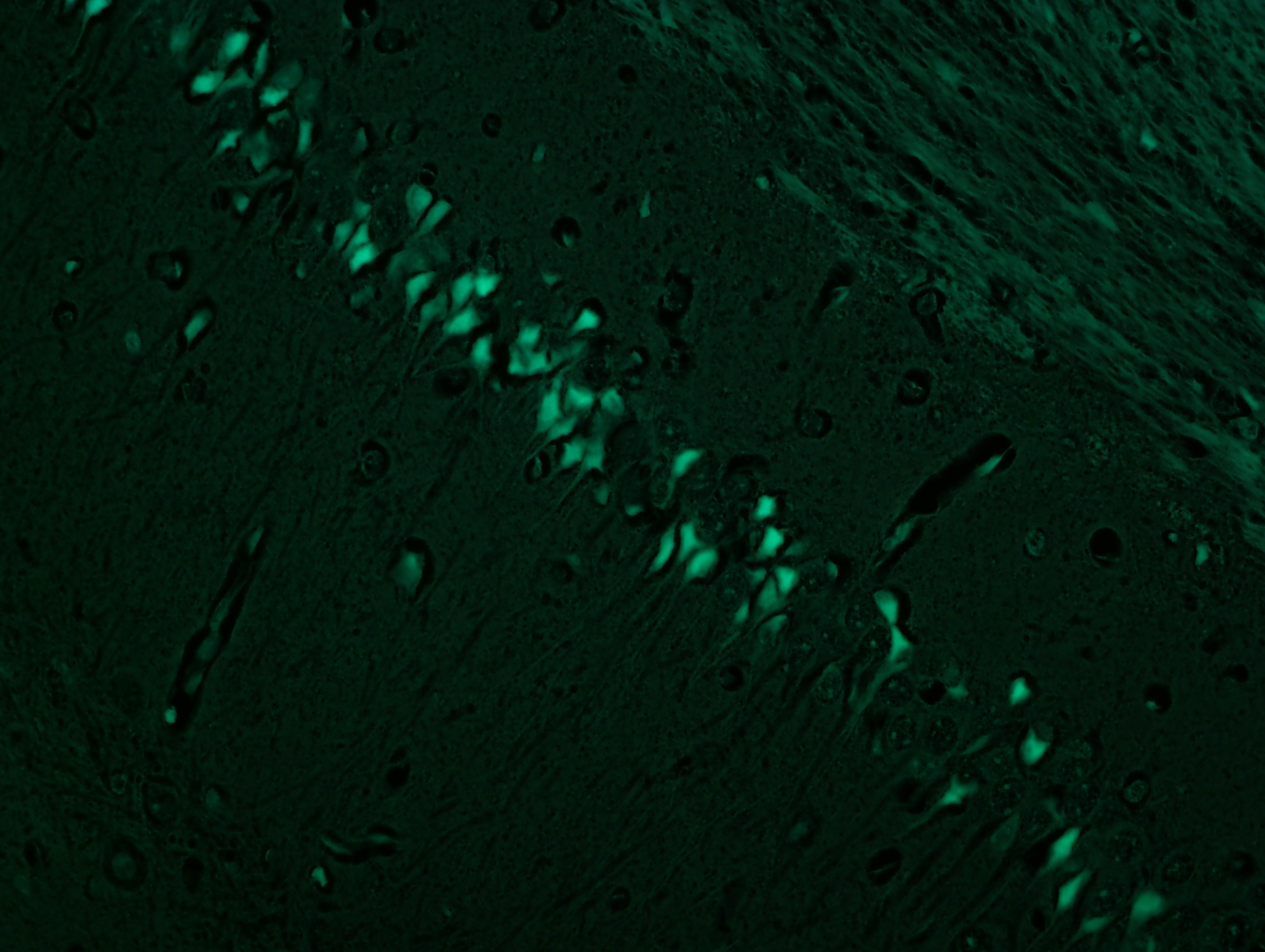

Supplement: Supplementary file 1 [file Data_Sheet_1.ZIP › hypoxia/hipp-CA1-40x.tif]

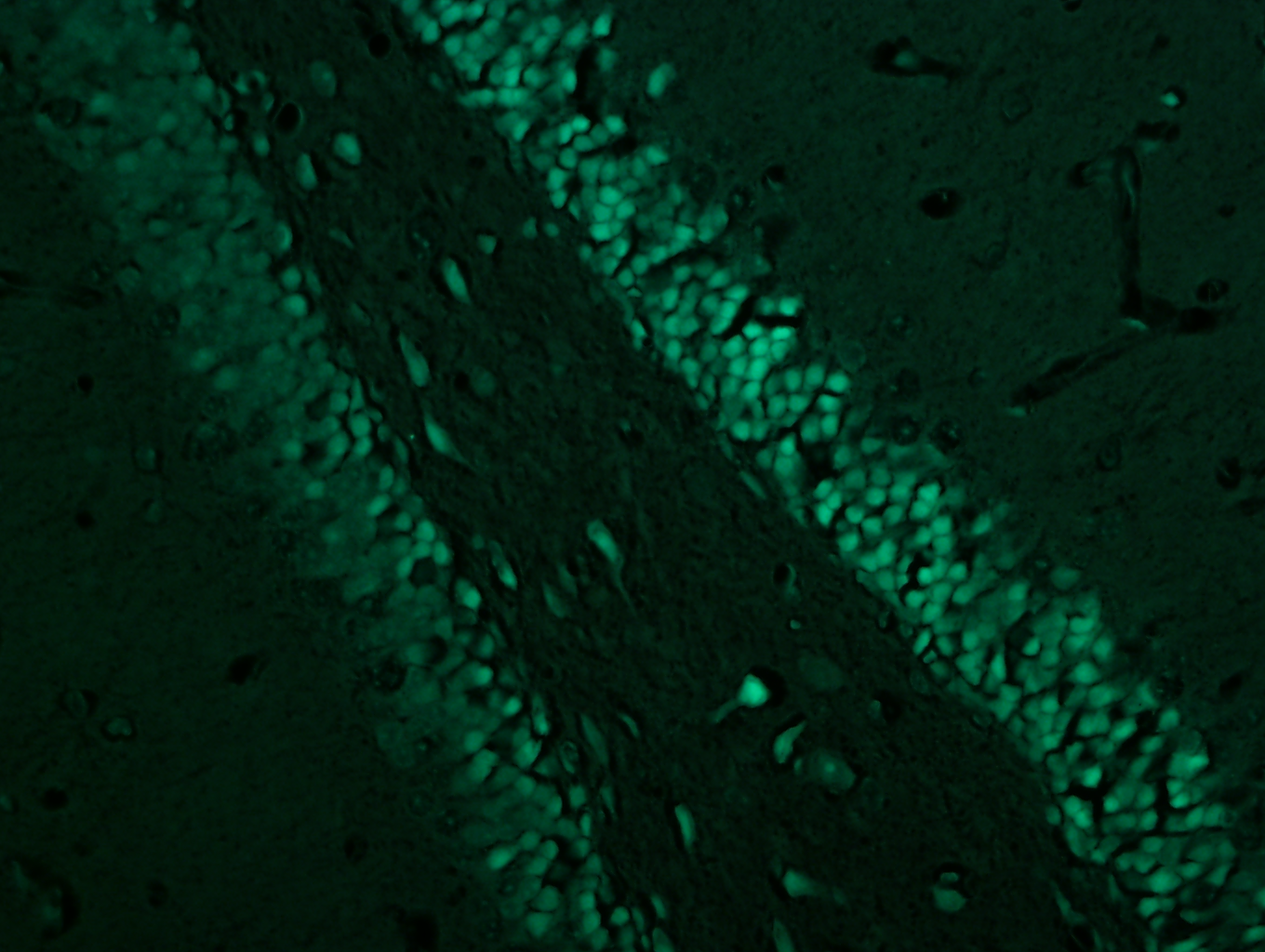

Supplement: Supplementary file 1 [file Data_Sheet_1.ZIP › hypoxia/hipp-DG-40x.tif]

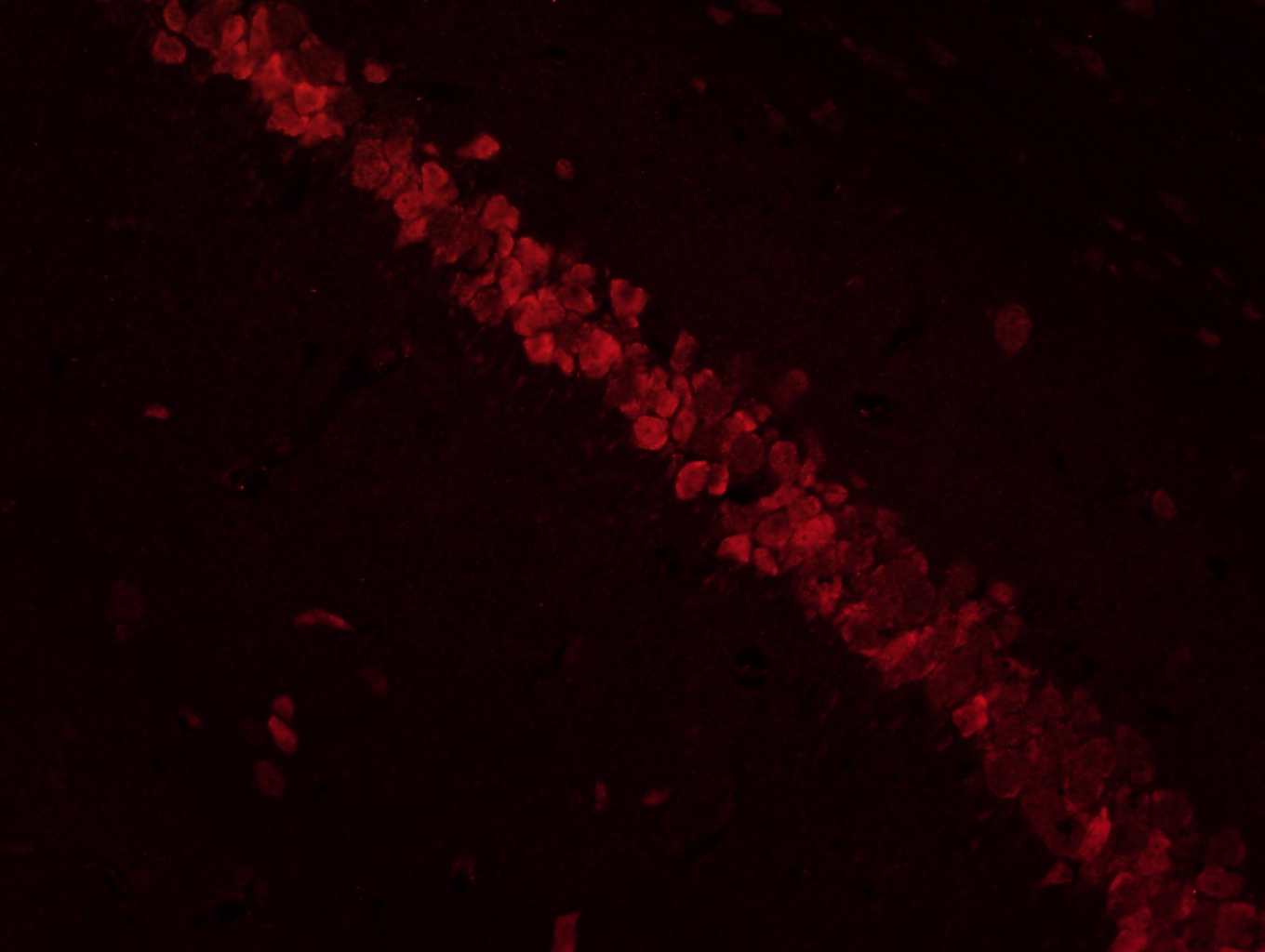

Supplement: Supplementary file 2 [file Data_Sheet_2.ZIP › c/CA1-40x-1.tif]

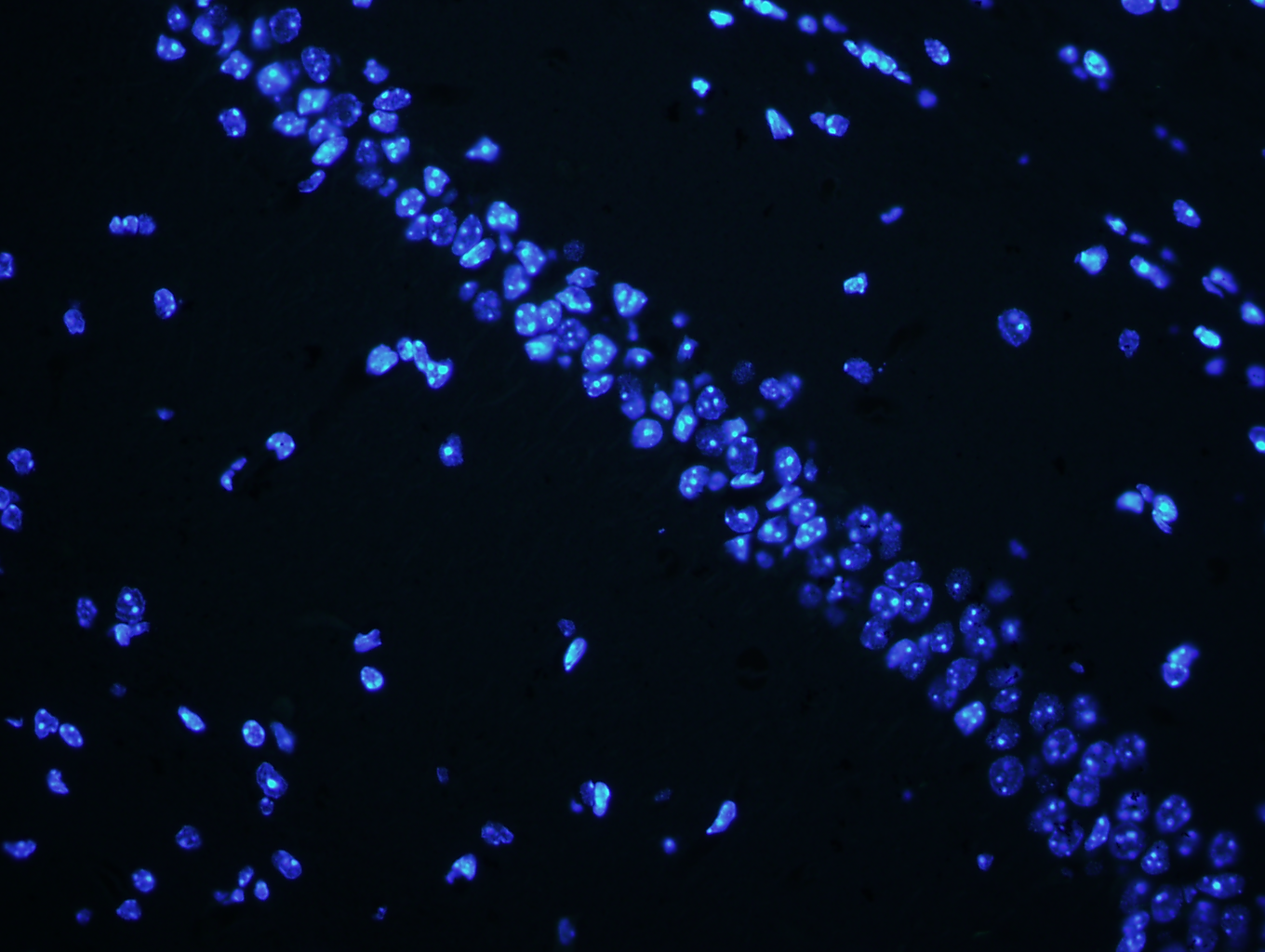

Supplement: Supplementary file 2 [file Data_Sheet_2.ZIP › c/CA1-40x-2.tif]

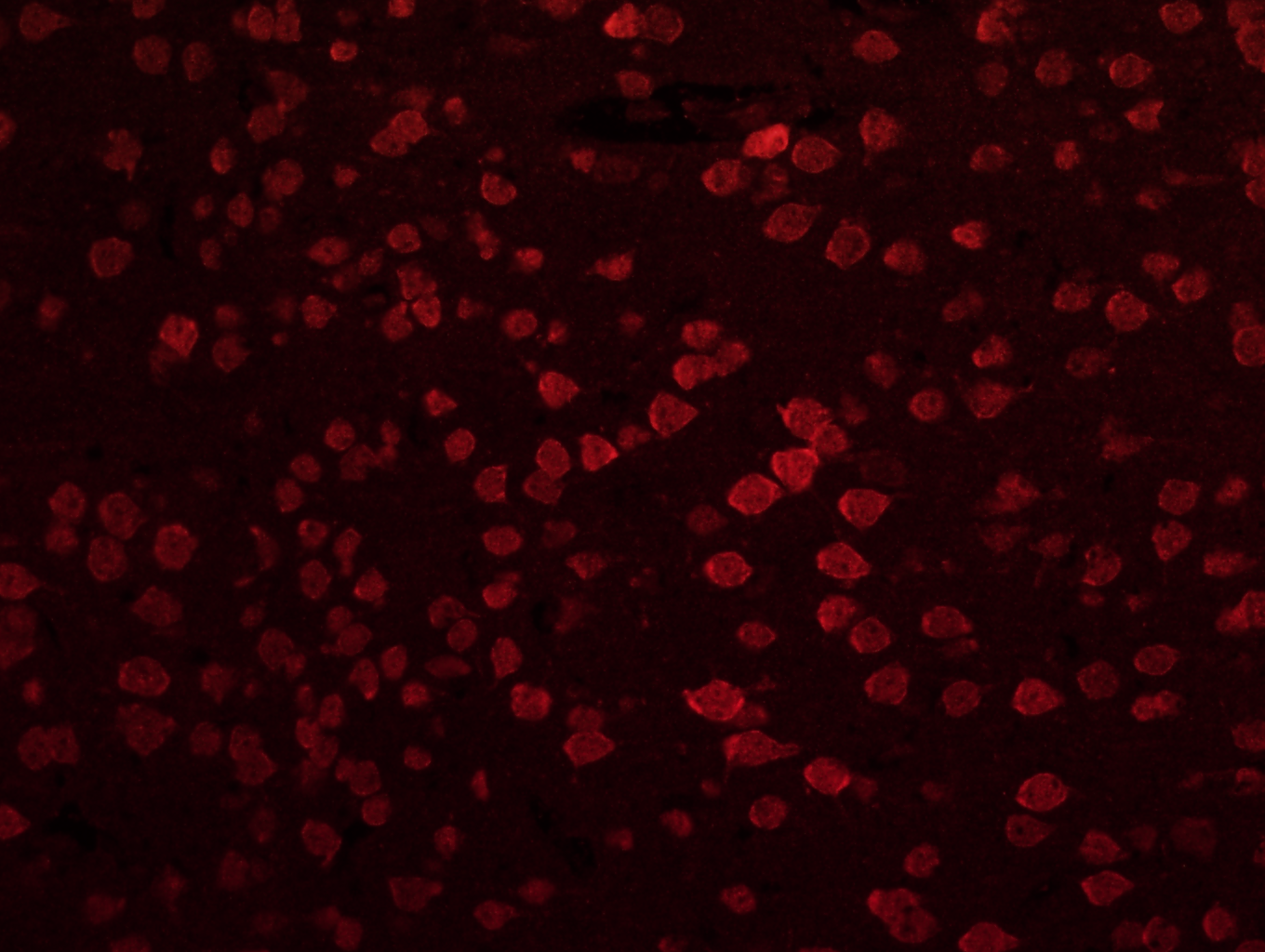

Supplement: Supplementary file 2 [file Data_Sheet_2.ZIP › c/cortex-40x-1.tif]

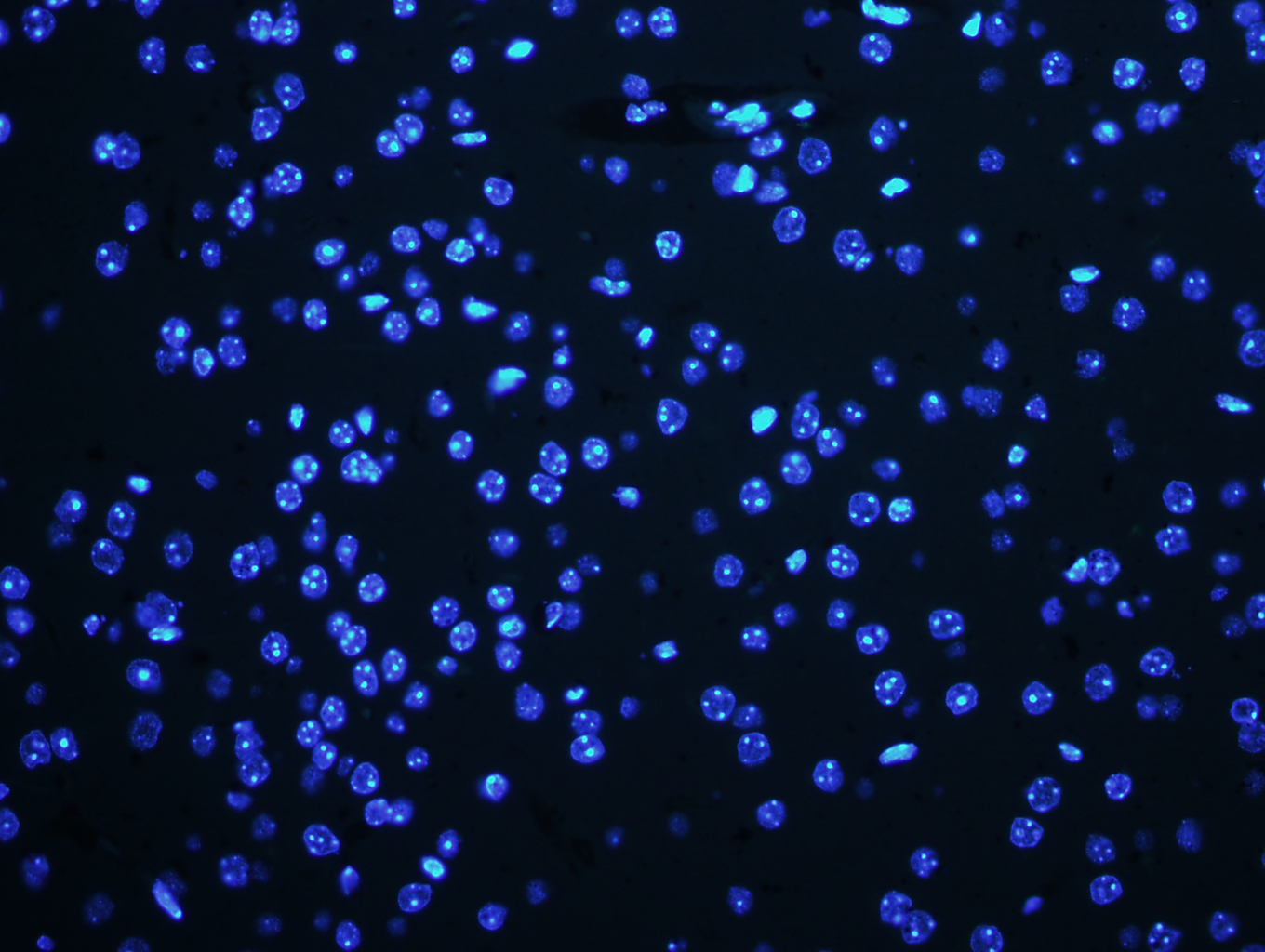

Supplement: Supplementary file 2 [file Data_Sheet_2.ZIP › c/cortex-40x-2.tif]

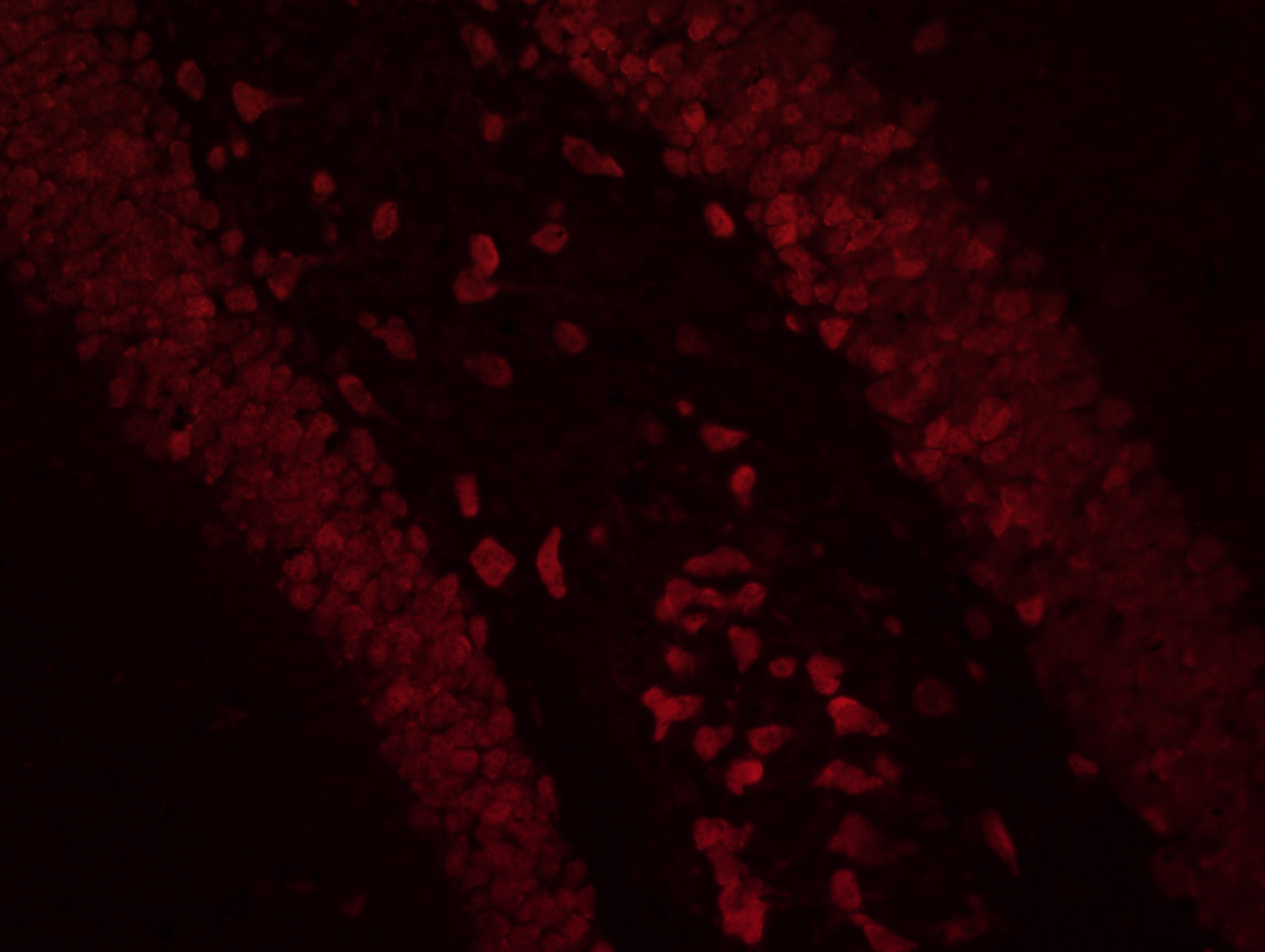

Supplement: Supplementary file 2 [file Data_Sheet_2.ZIP › c/DG-40x-1.tif]

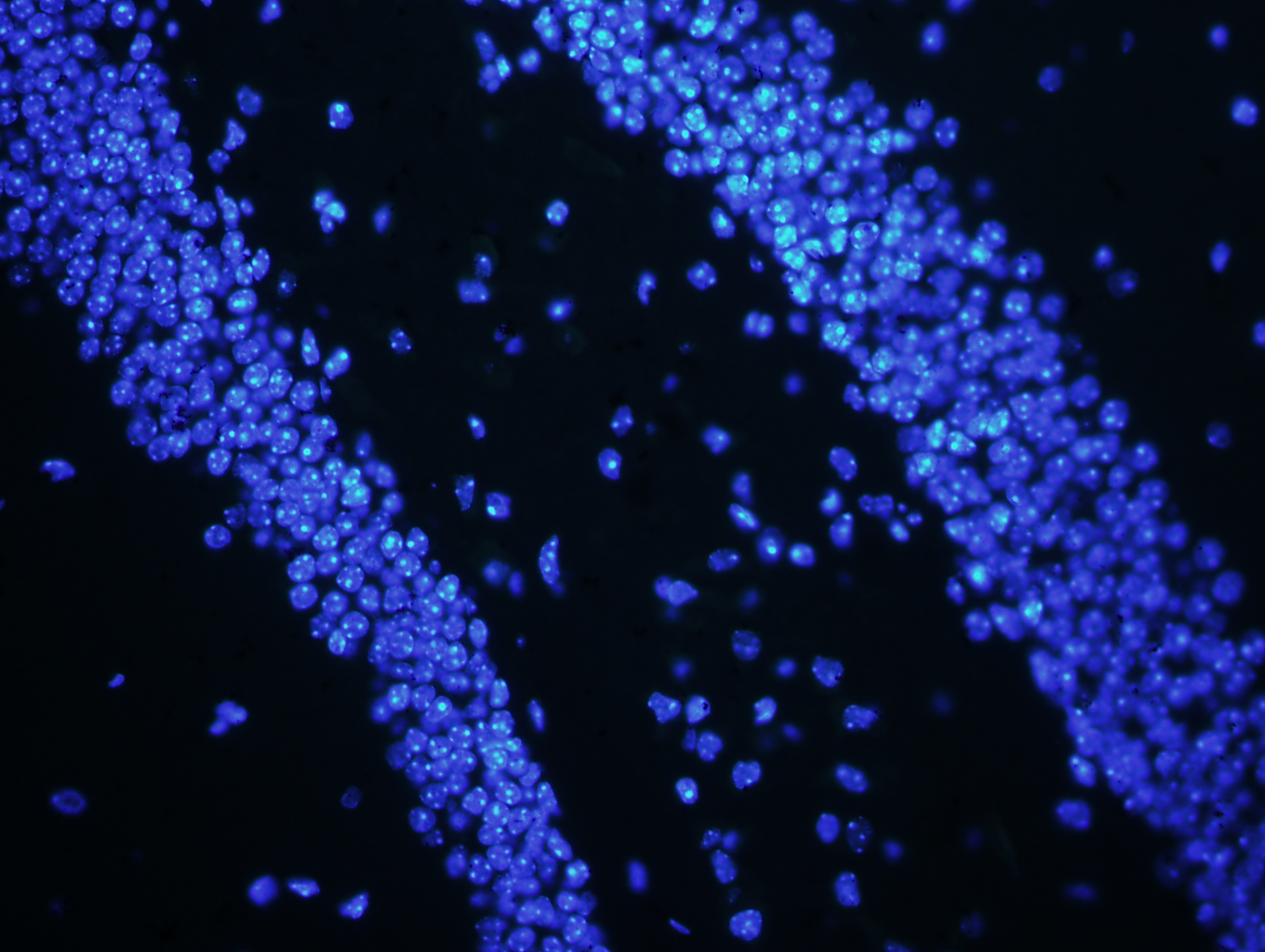

Supplement: Supplementary file 2 [file Data_Sheet_2.ZIP › c/DG-40x-2.tif]

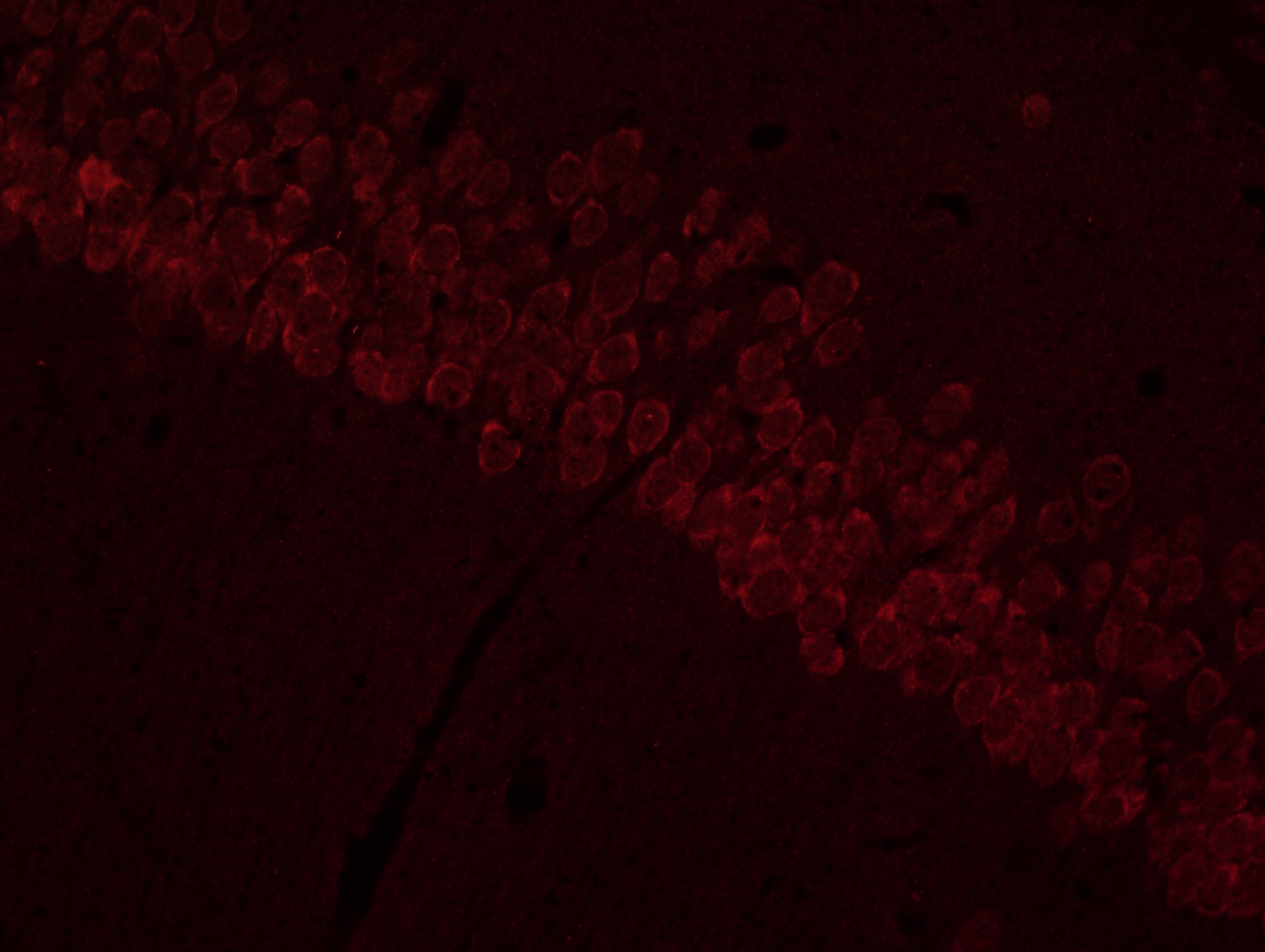

Supplement: Supplementary file 2 [file Data_Sheet_2.ZIP › h/CA1-40x-1.tif]

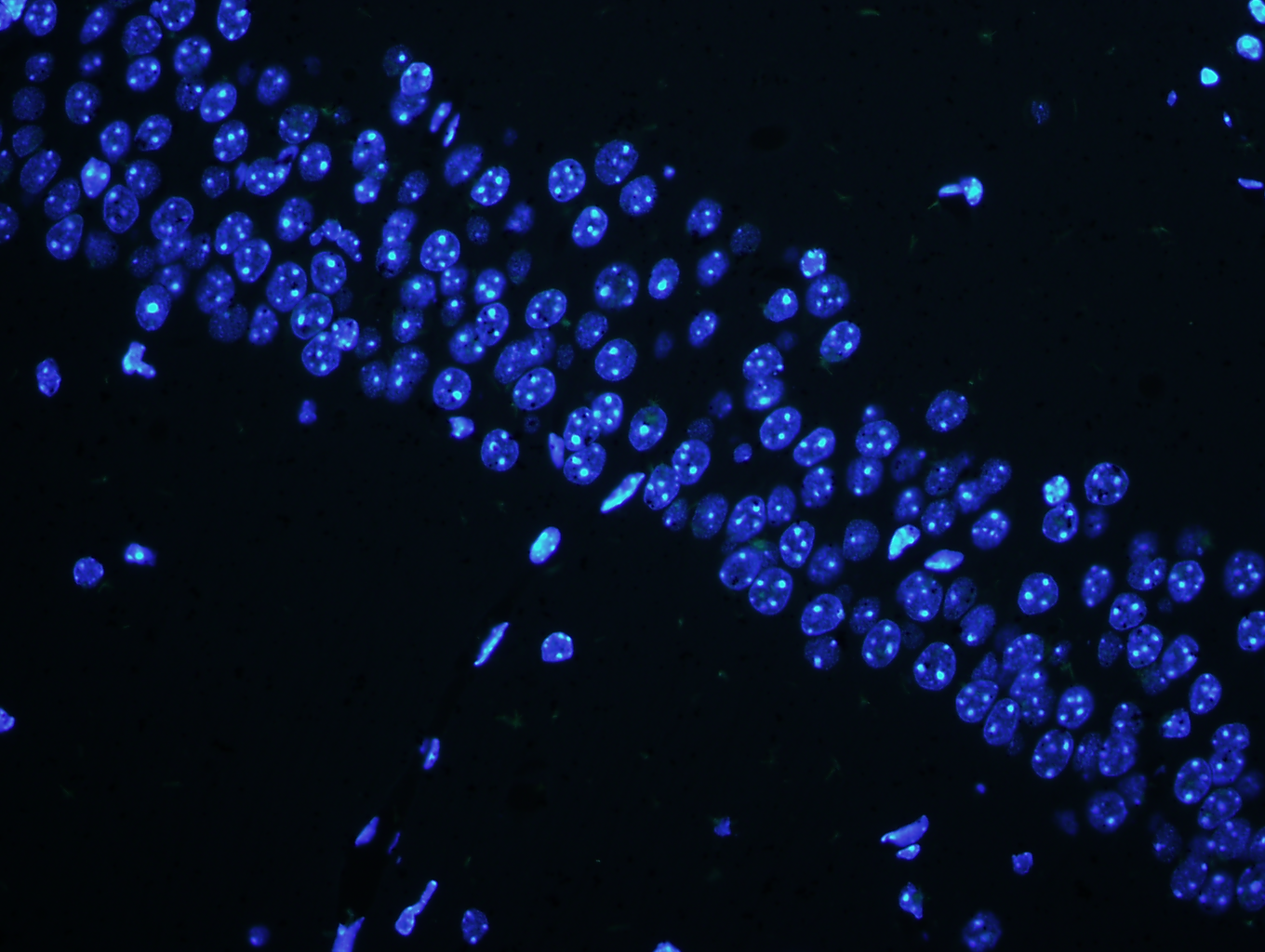

Supplement: Supplementary file 2 [file Data_Sheet_2.ZIP › h/CA1-40x-2.tif]

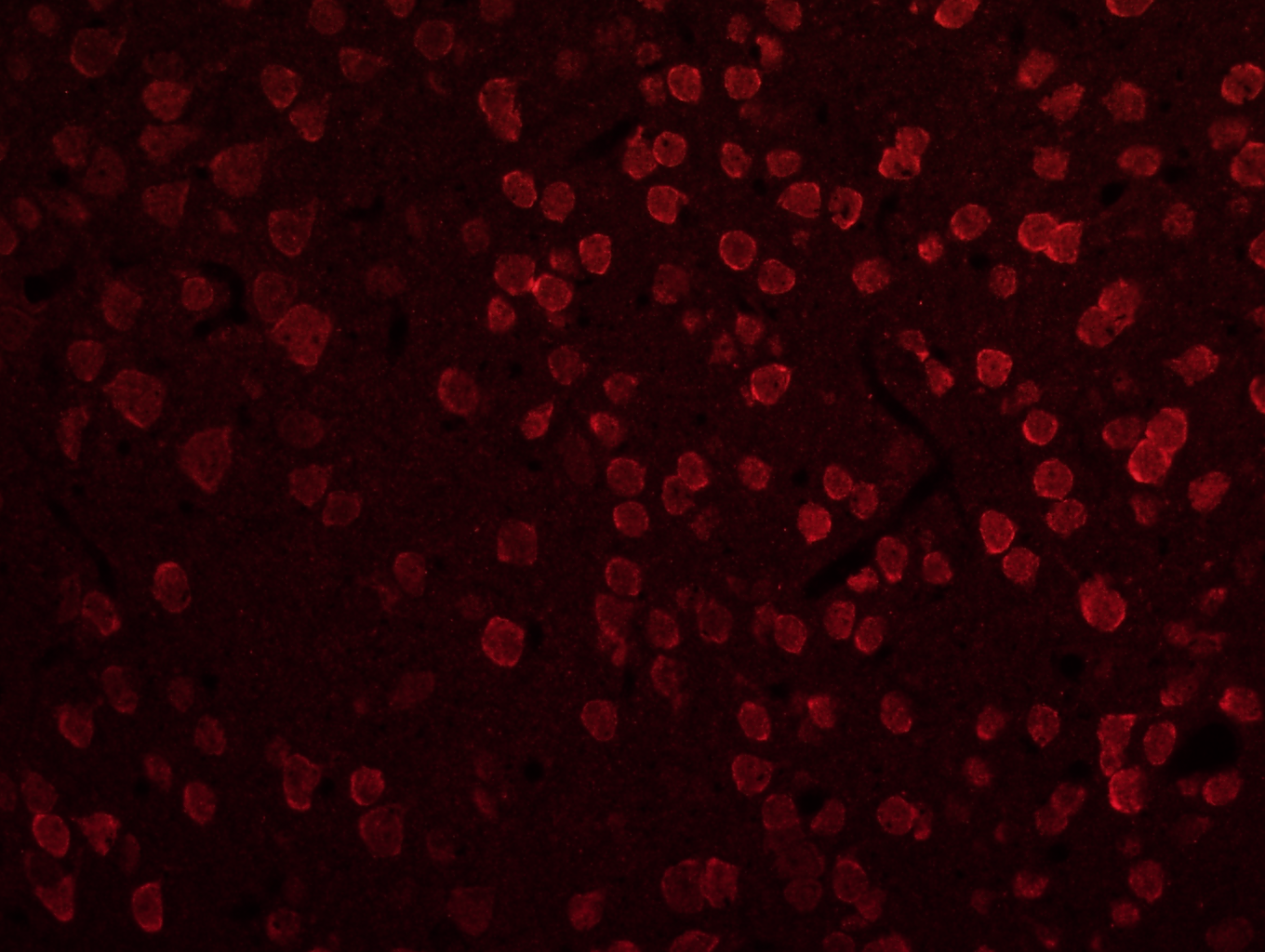

Supplement: Supplementary file 2 [file Data_Sheet_2.ZIP › h/cortex-40x-1.tif]

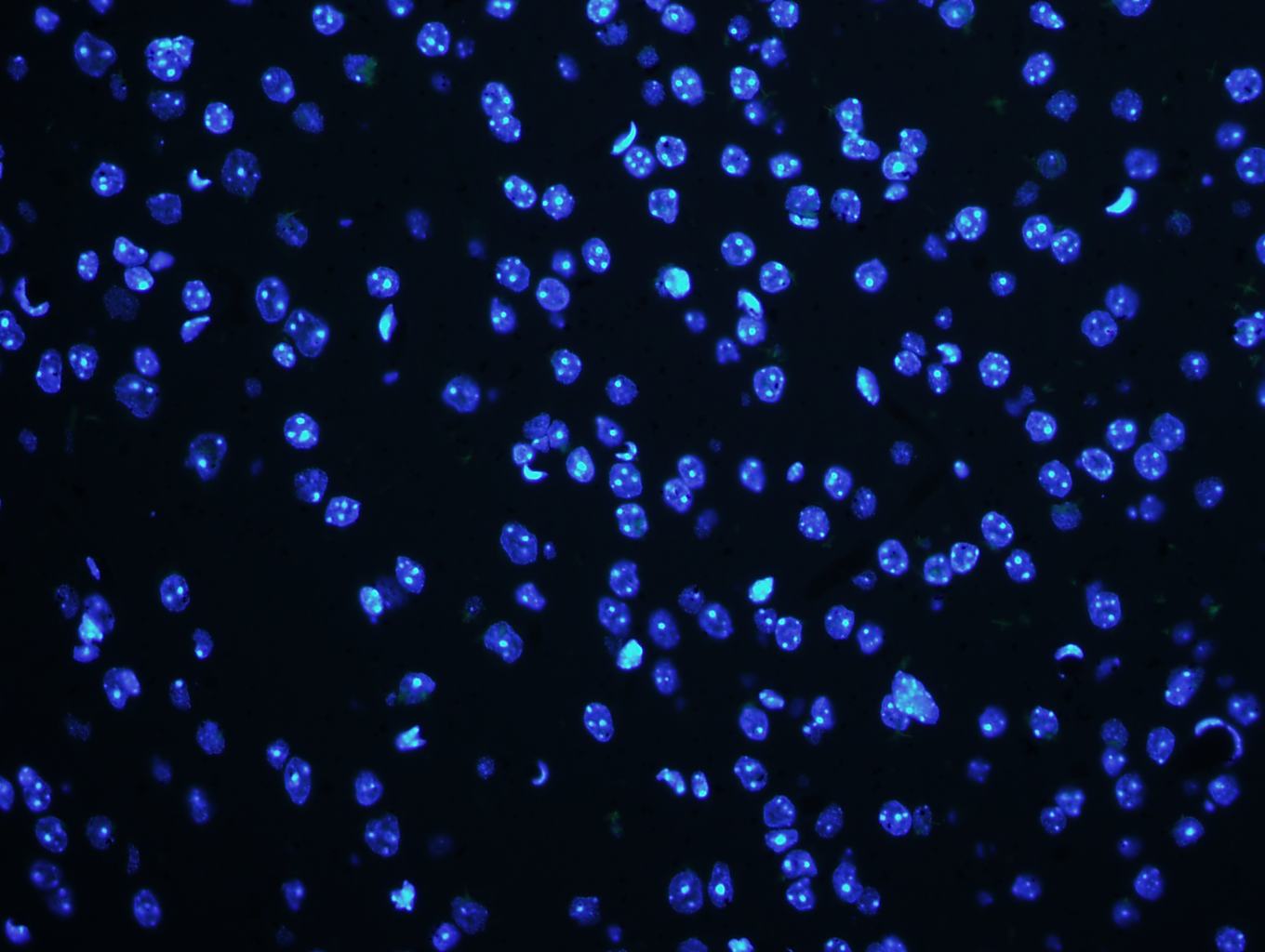

Supplement: Supplementary file 2 [file Data_Sheet_2.ZIP › h/cortex-40x-2.tif]

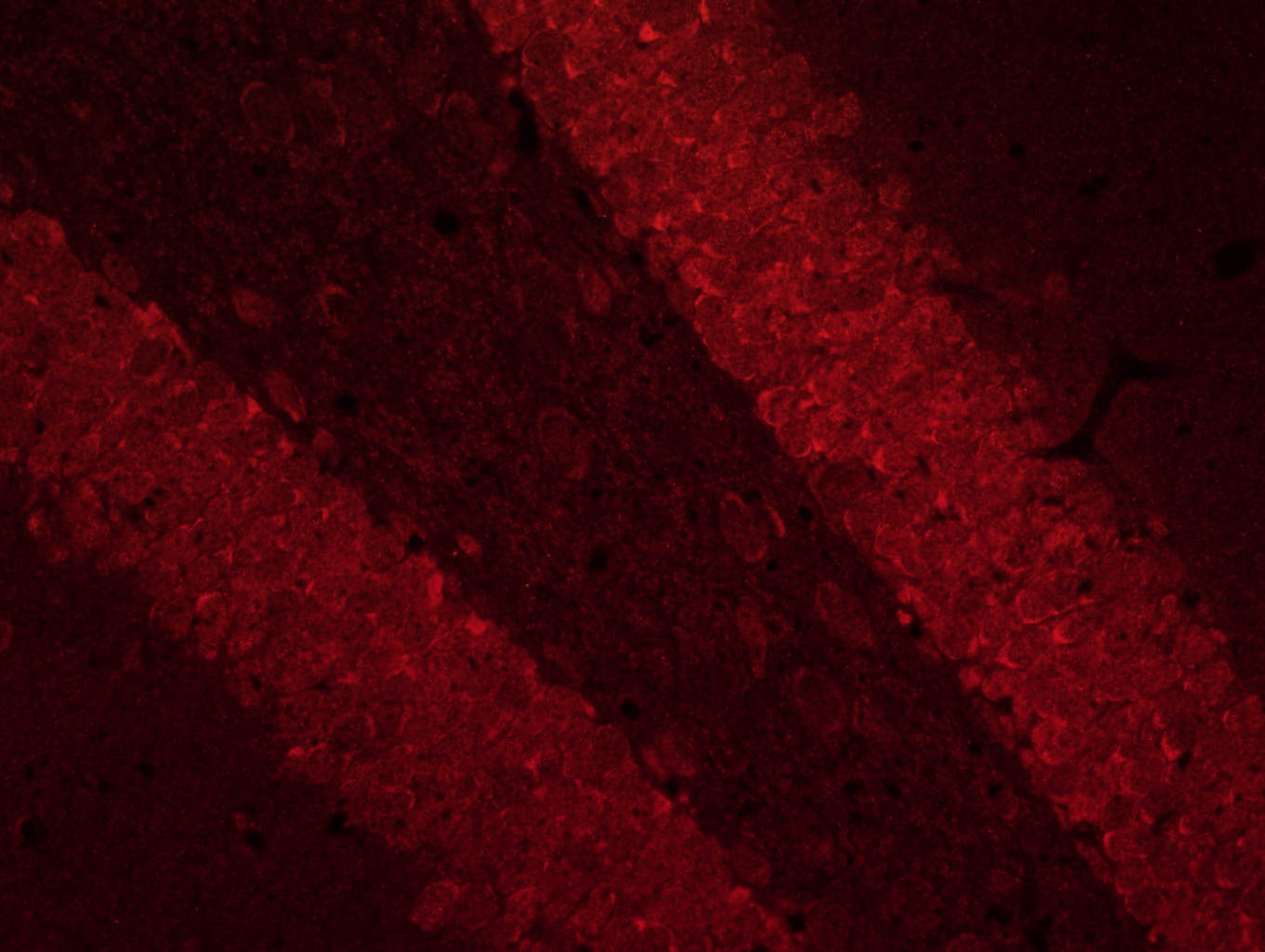

Supplement: Supplementary file 2 [file Data_Sheet_2.ZIP › h/DG-40x-1.tif]

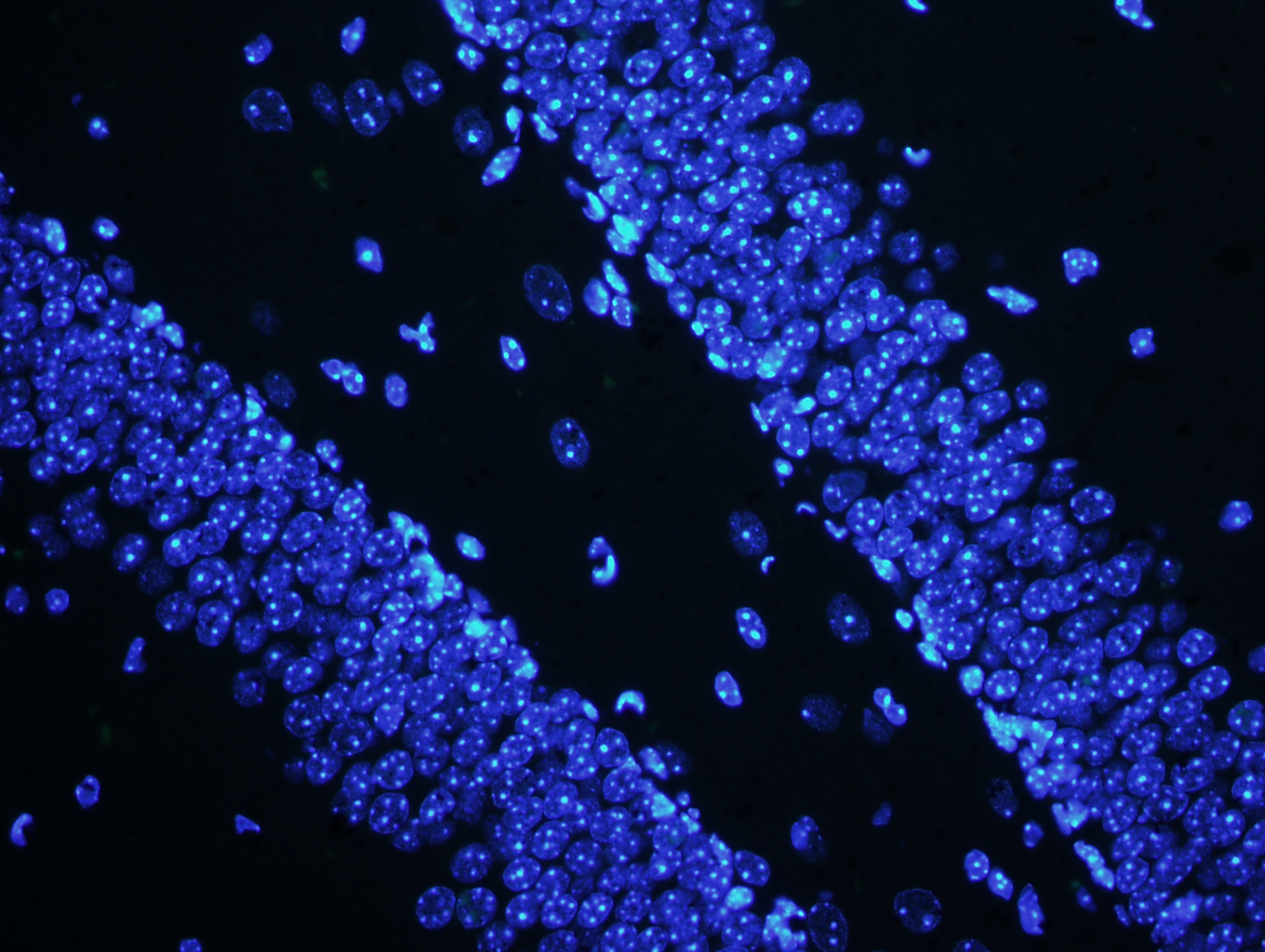

Supplement: Supplementary file 2 [file Data_Sheet_2.ZIP › h/DG-40x-2.tif]

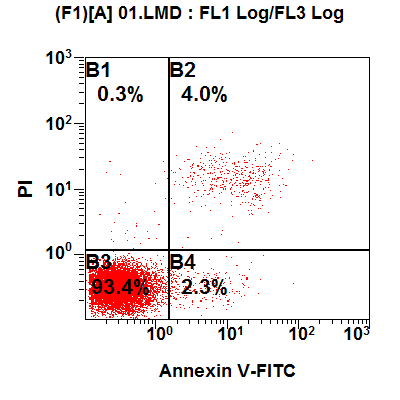

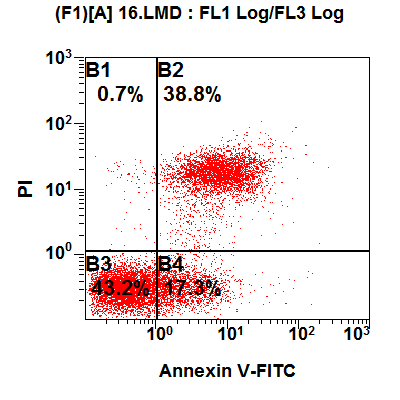

Supplement: Supplementary file 3 [file Data_Sheet_3.ZIP › 新建 Microsoft Word 文档.docx]

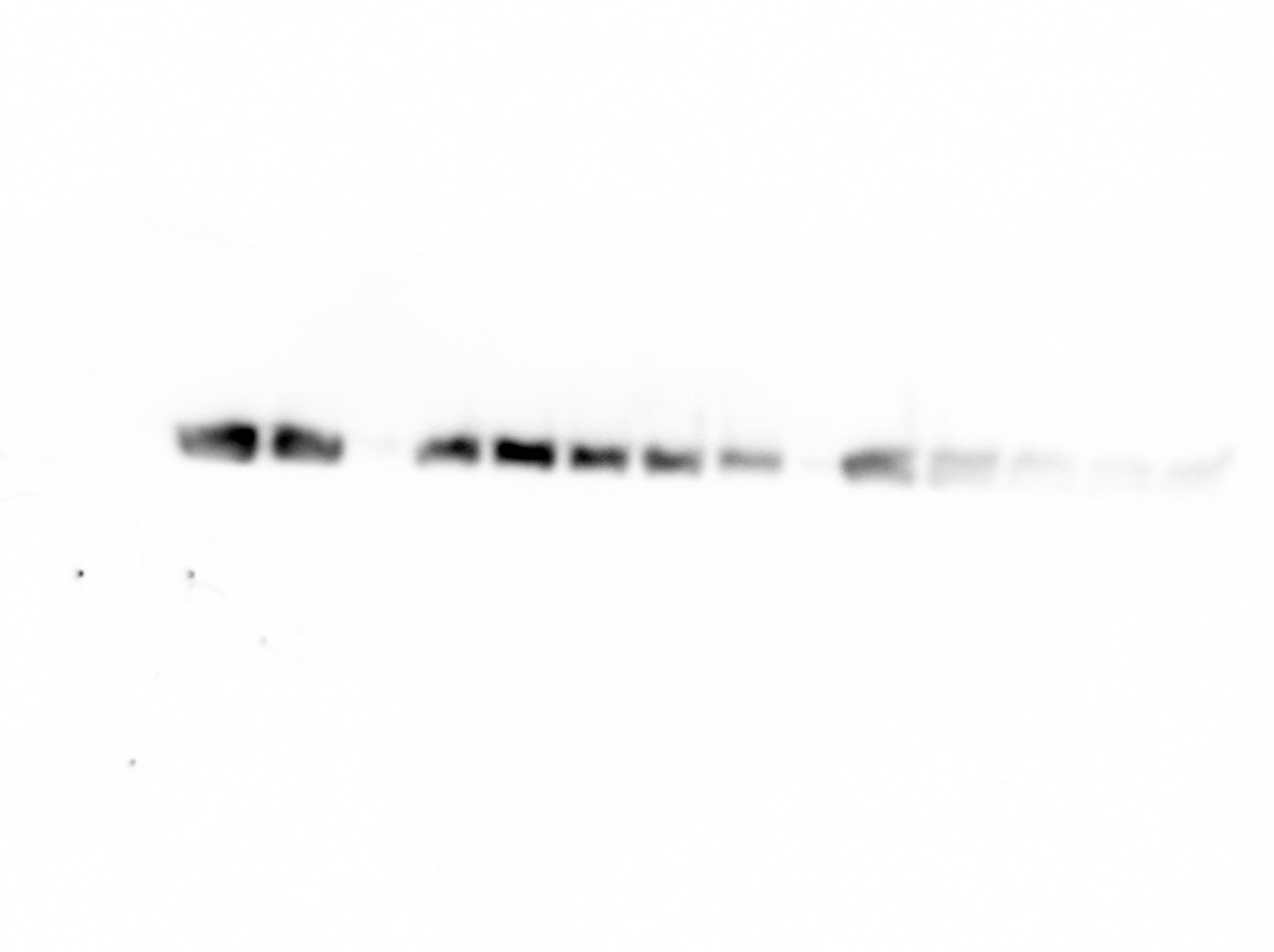

Supplement: Supplementary file 4 [file Data_Sheet_4.ZIP › 1-cas3-1.tif]

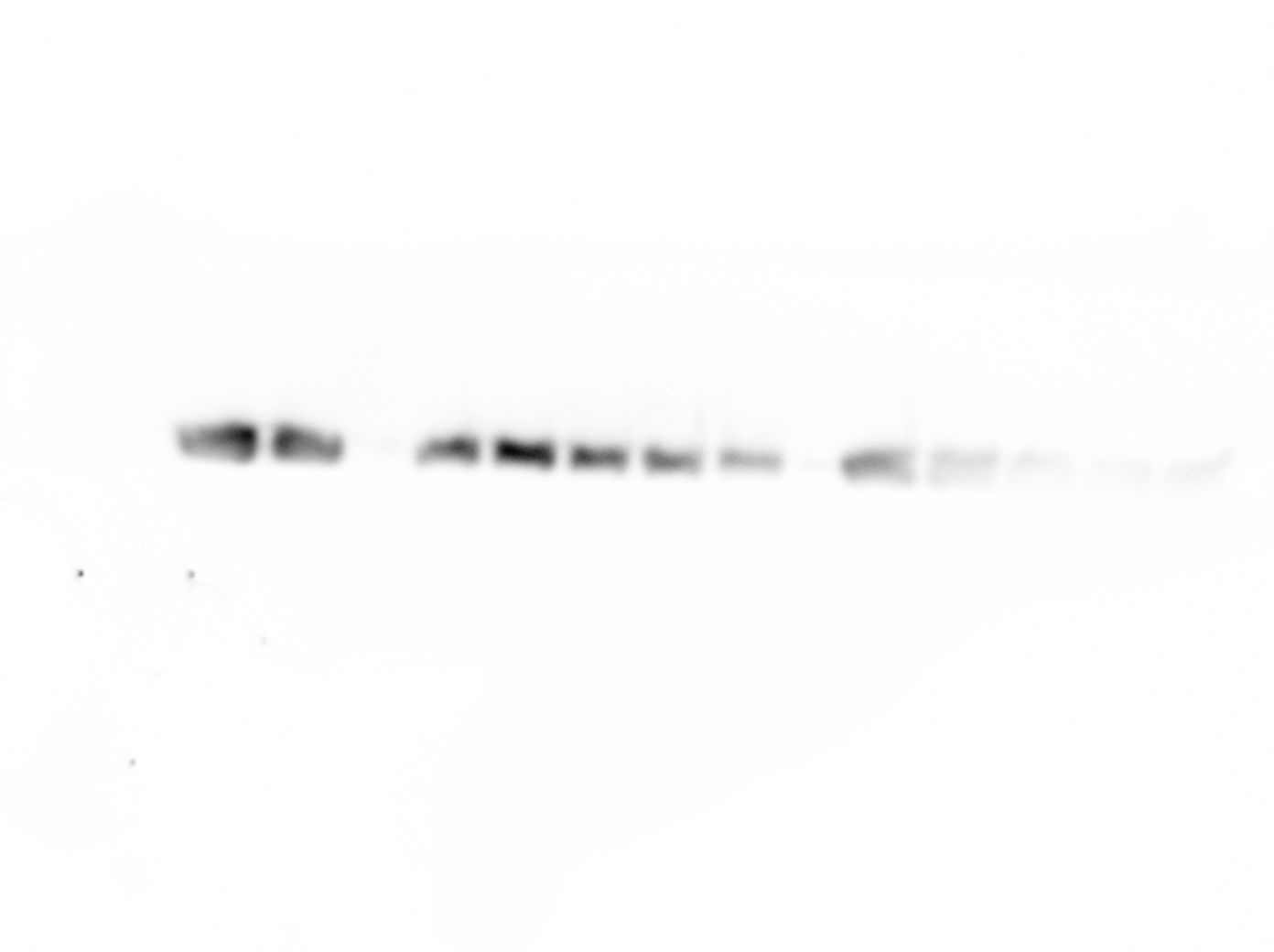

Supplement: Supplementary file 4 [file Data_Sheet_4.ZIP › 1-cas3-2.tif]

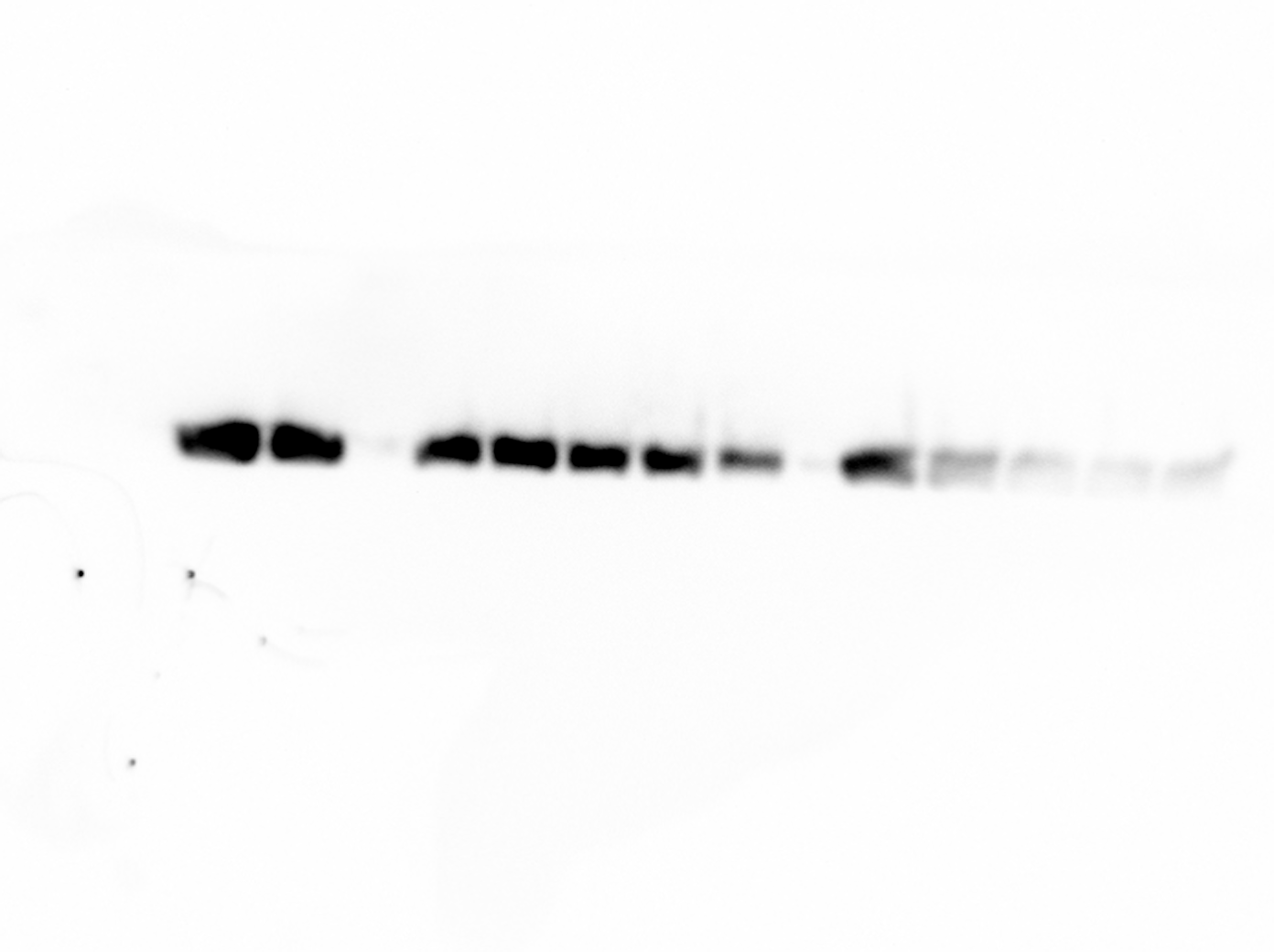

Supplement: Supplementary file 4 [file Data_Sheet_4.ZIP › 1-cas3-3.tif]

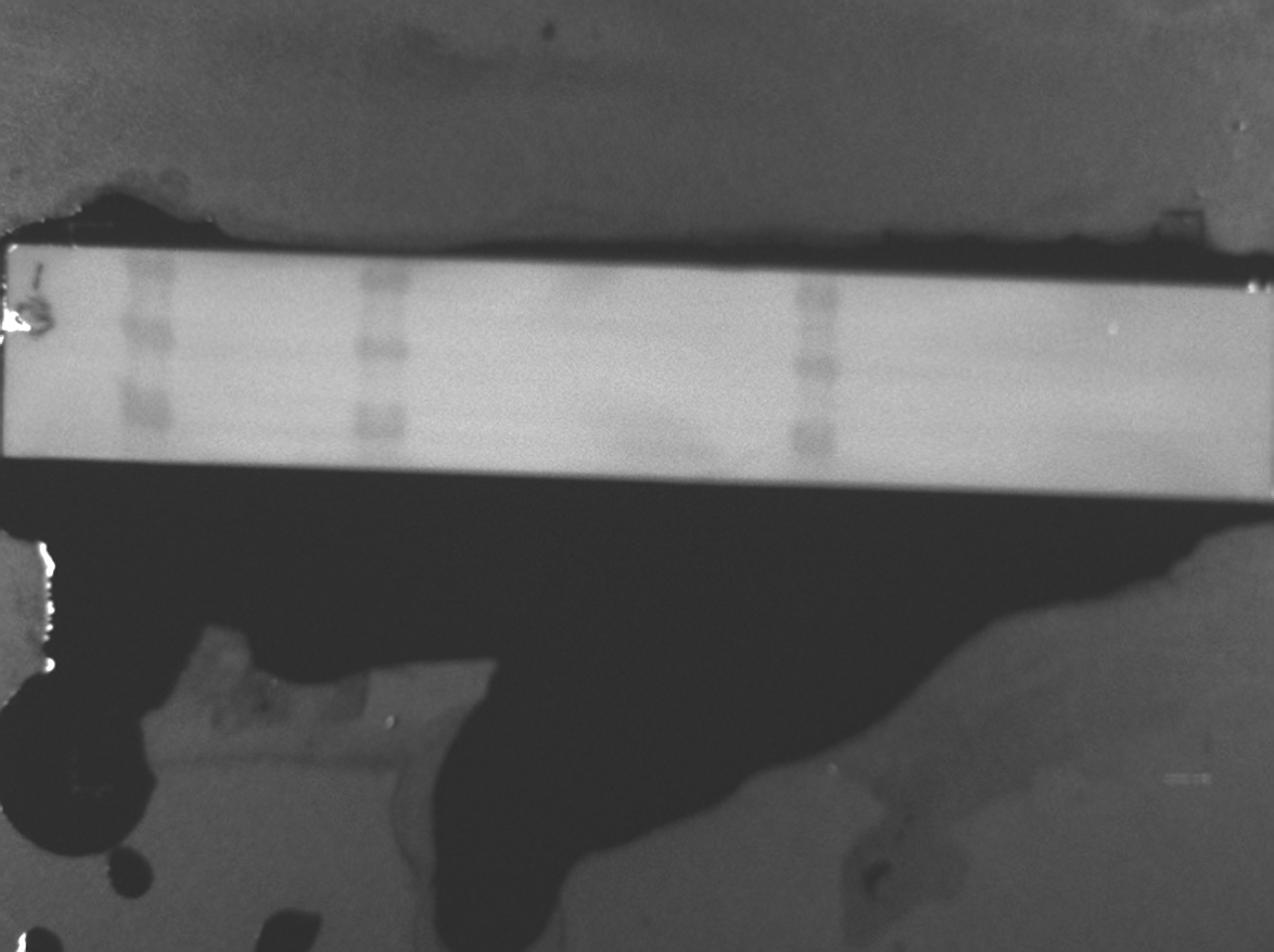

Supplement: Supplementary file 4 [file Data_Sheet_4.ZIP › 1-cas3-4.tif]

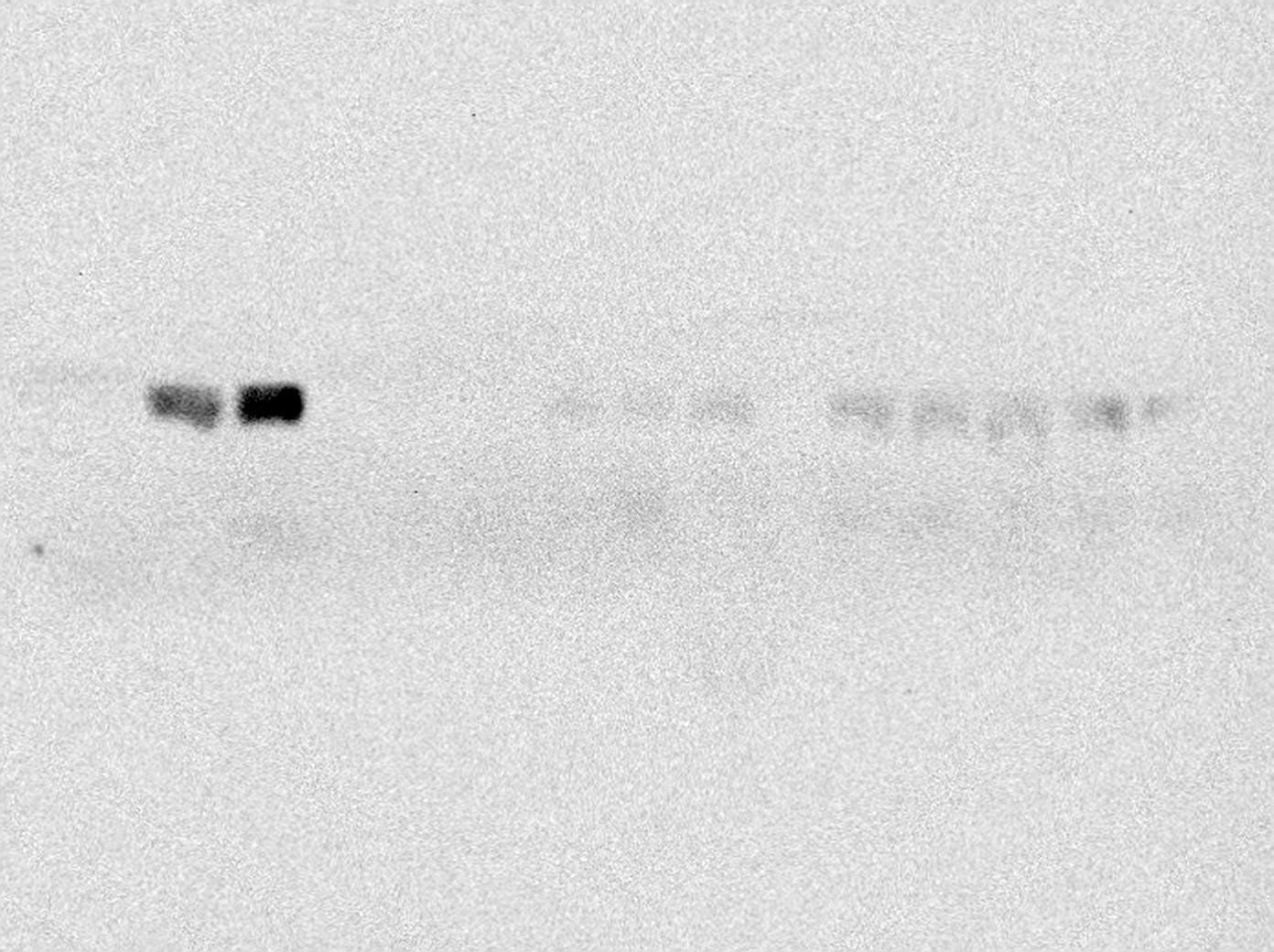

Supplement: Supplementary file 4 [file Data_Sheet_4.ZIP › 1-ccas3-1.tif]

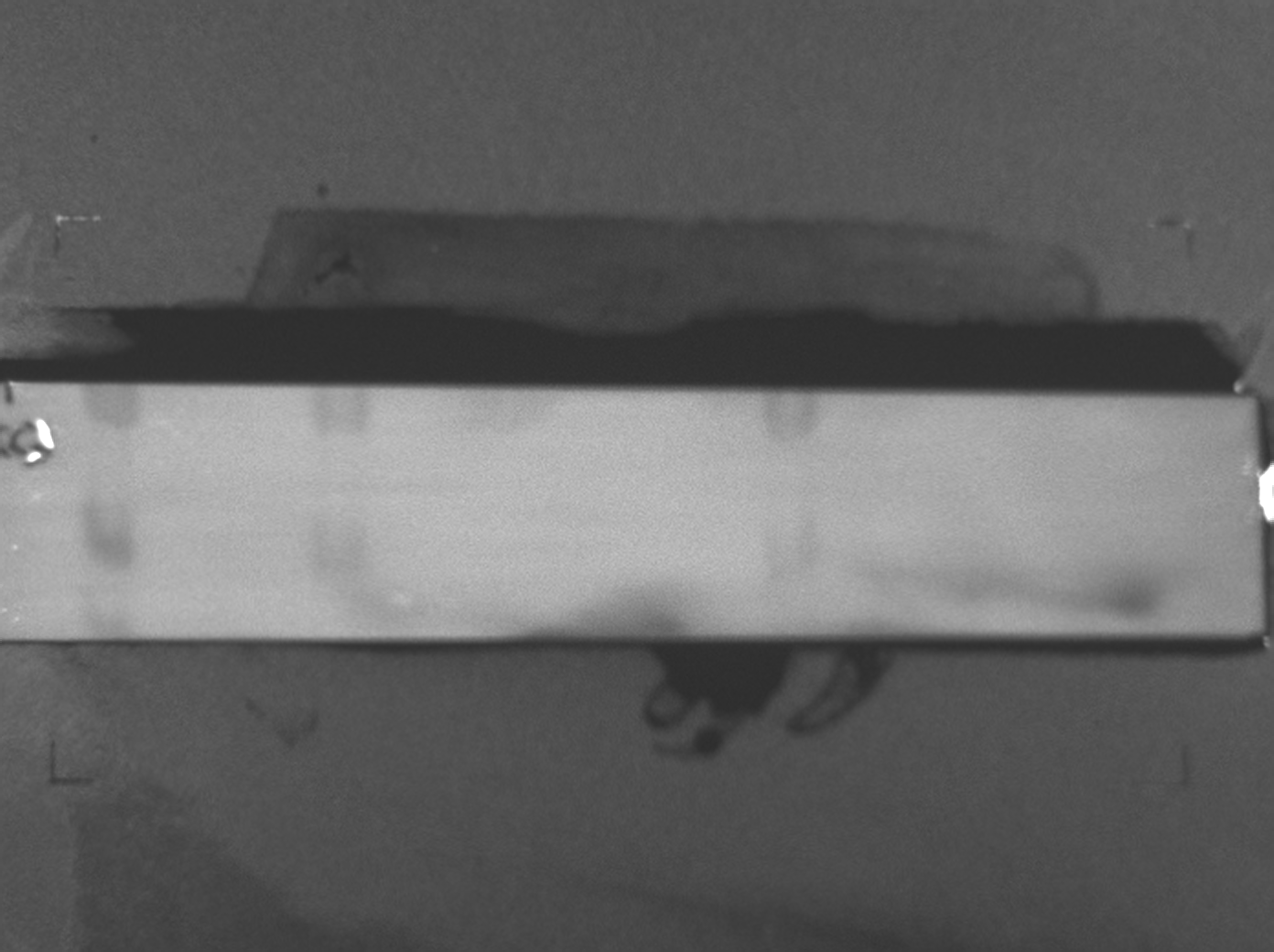

Supplement: Supplementary file 4 [file Data_Sheet_4.ZIP › 1-ccas3-2.tif]

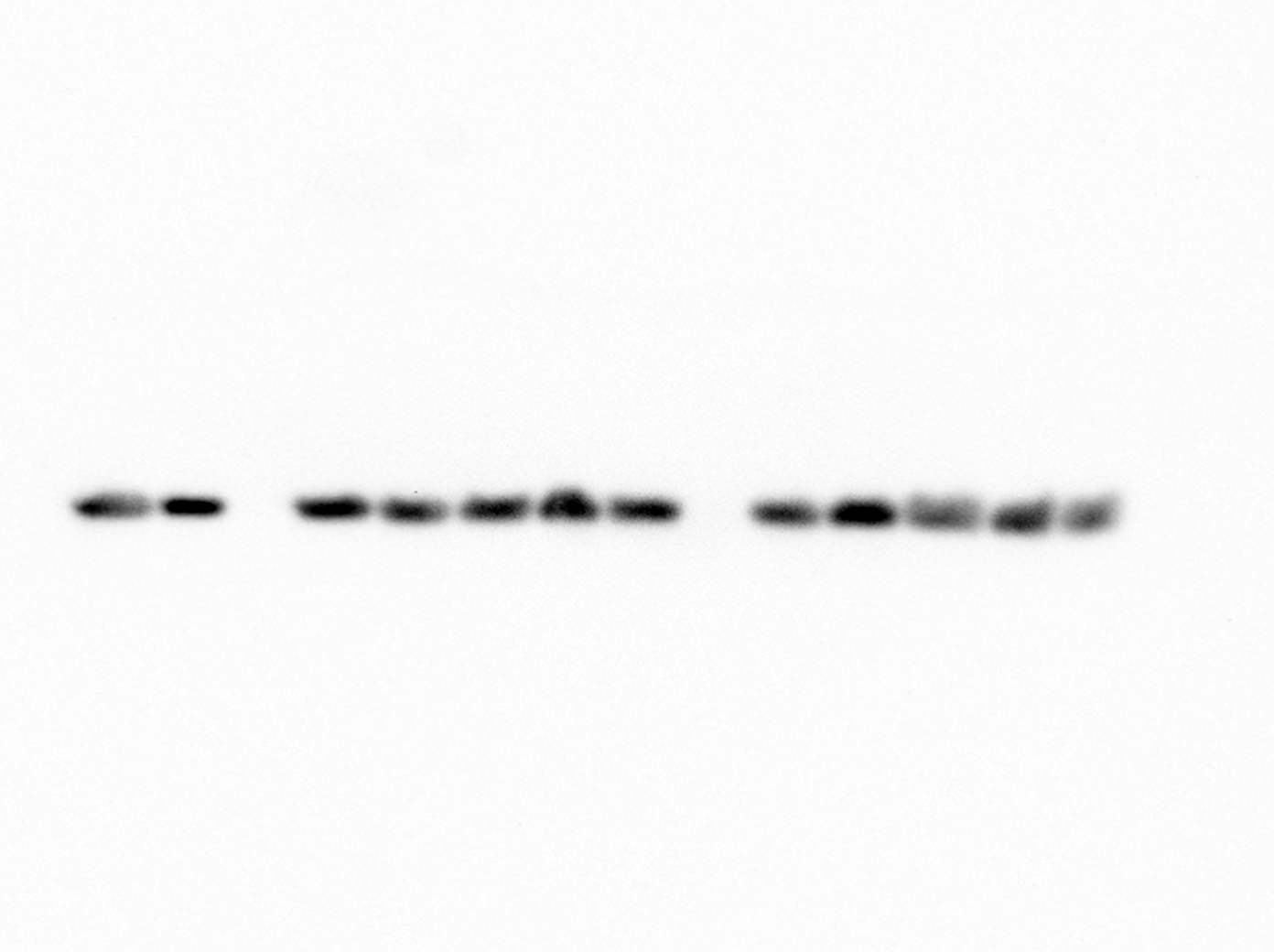

Supplement: Supplementary file 4 [file Data_Sheet_4.ZIP › 2-cyto c-1.tif]

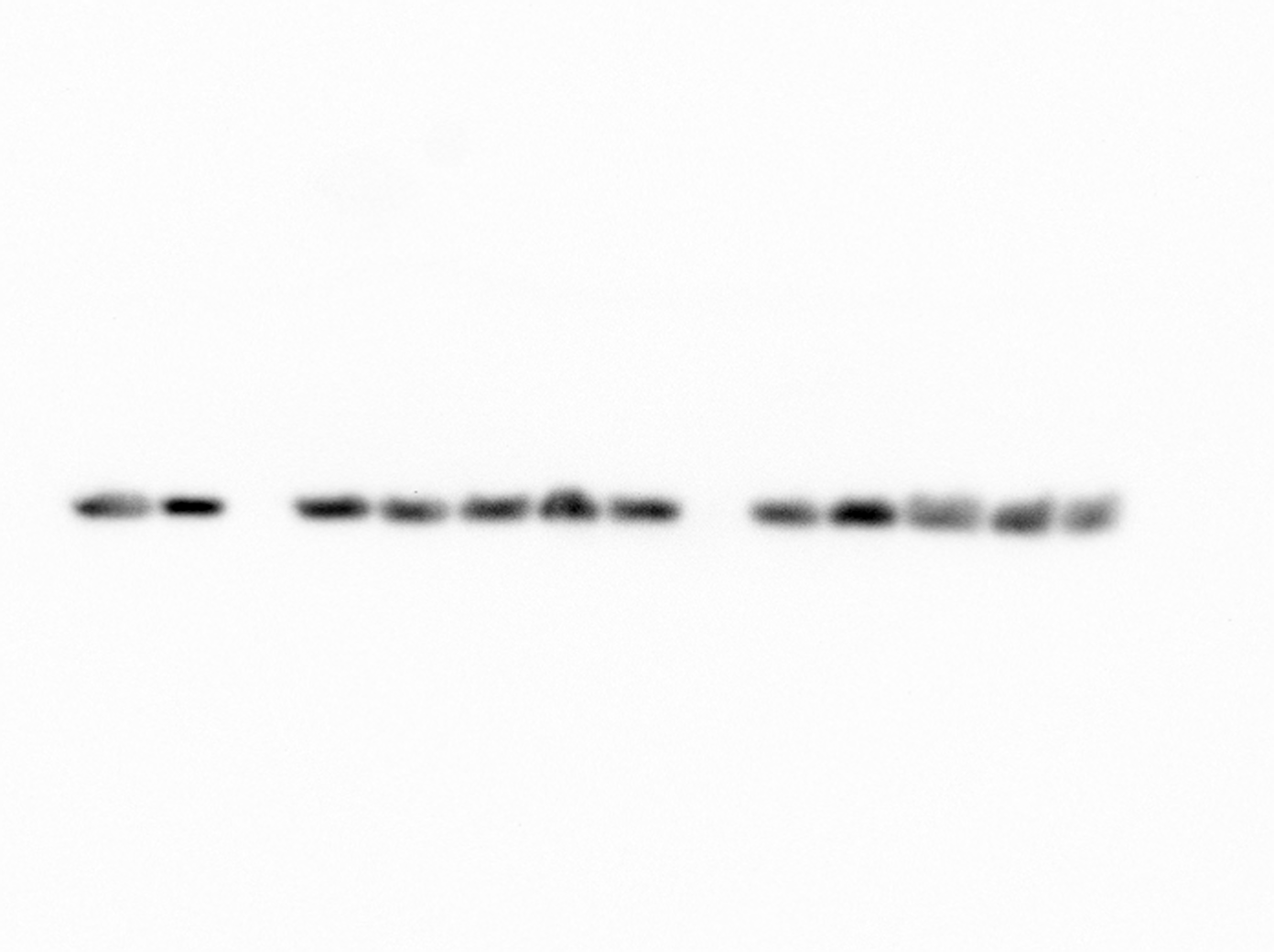

Supplement: Supplementary file 4 [file Data_Sheet_4.ZIP › 2-cyto c-2.tif]

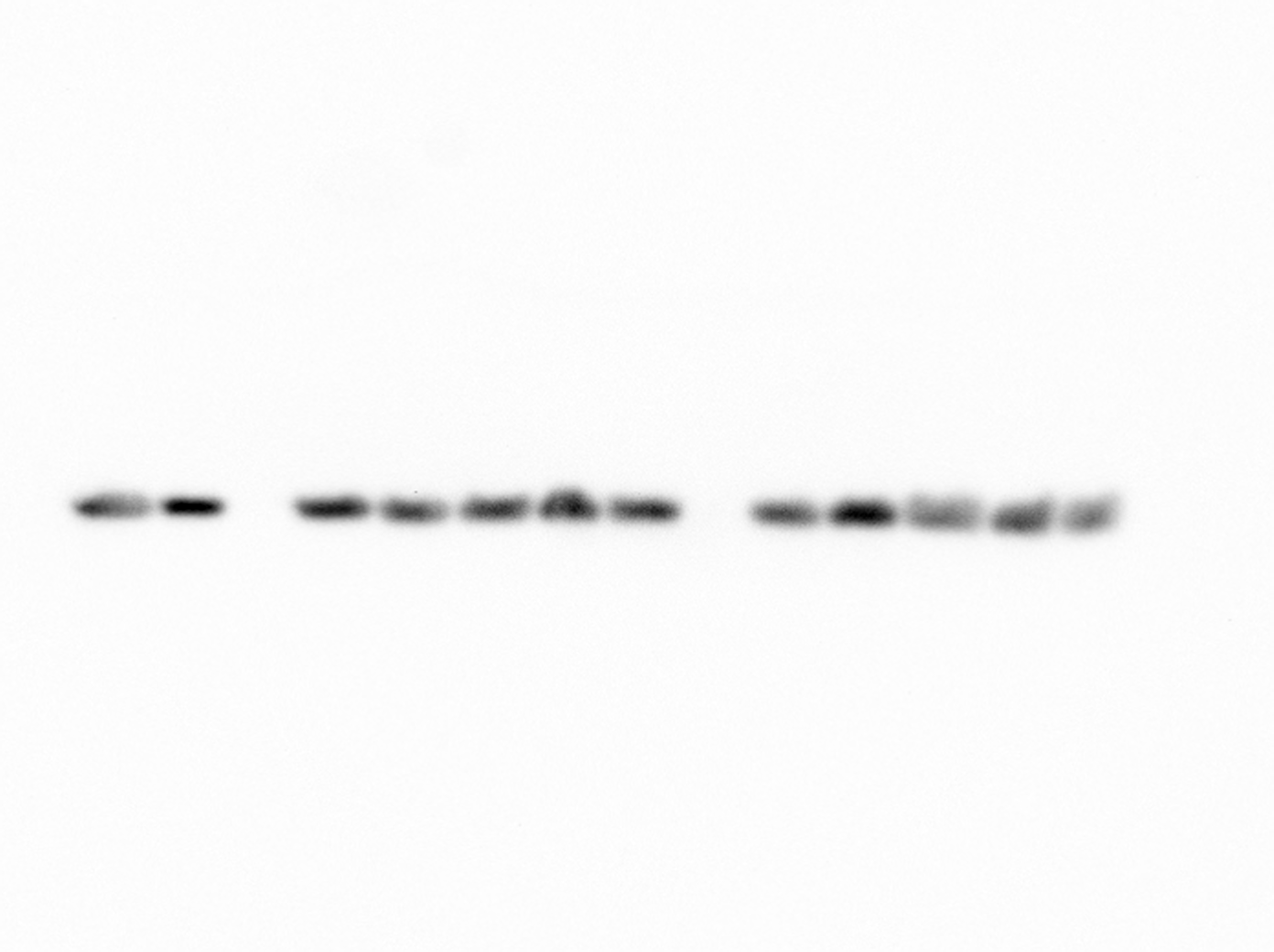

Supplement: Supplementary file 4 [file Data_Sheet_4.ZIP › 2-cyto c-3.tif]

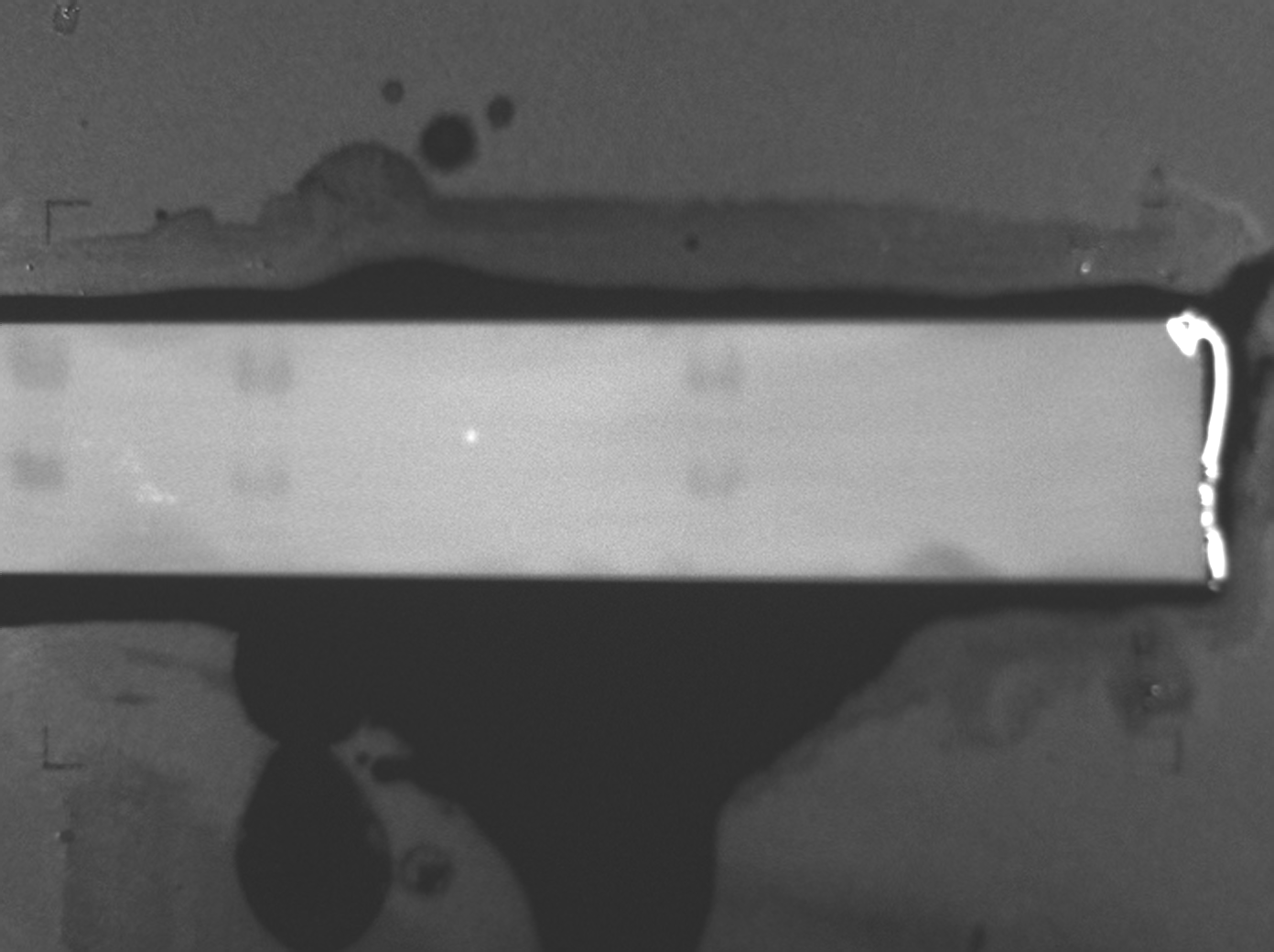

Supplement: Supplementary file 4 [file Data_Sheet_4.ZIP › 2-cyto c-4.tif]

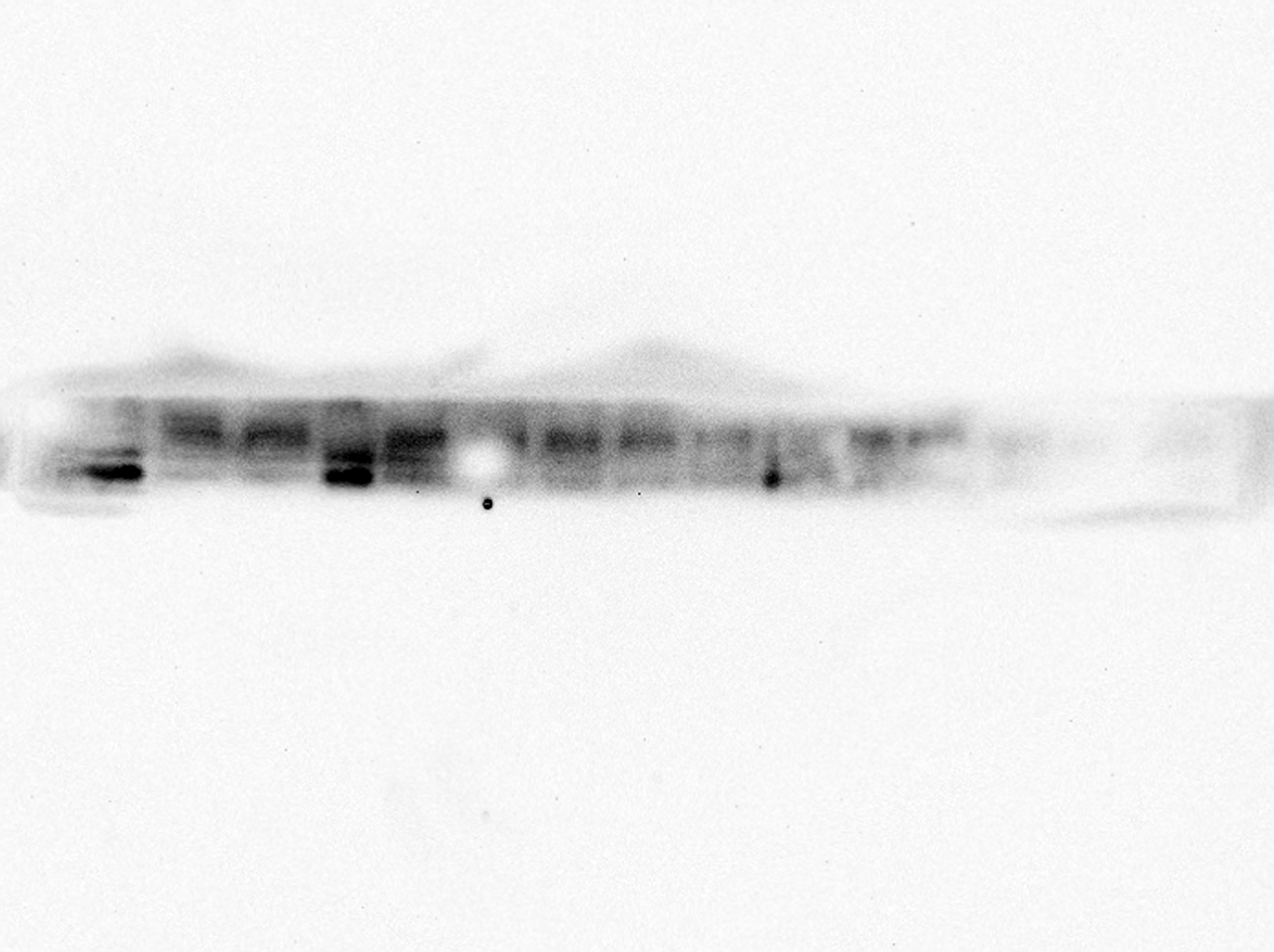

Supplement: Supplementary file 4 [file Data_Sheet_4.ZIP › 4-hif-1.tif]

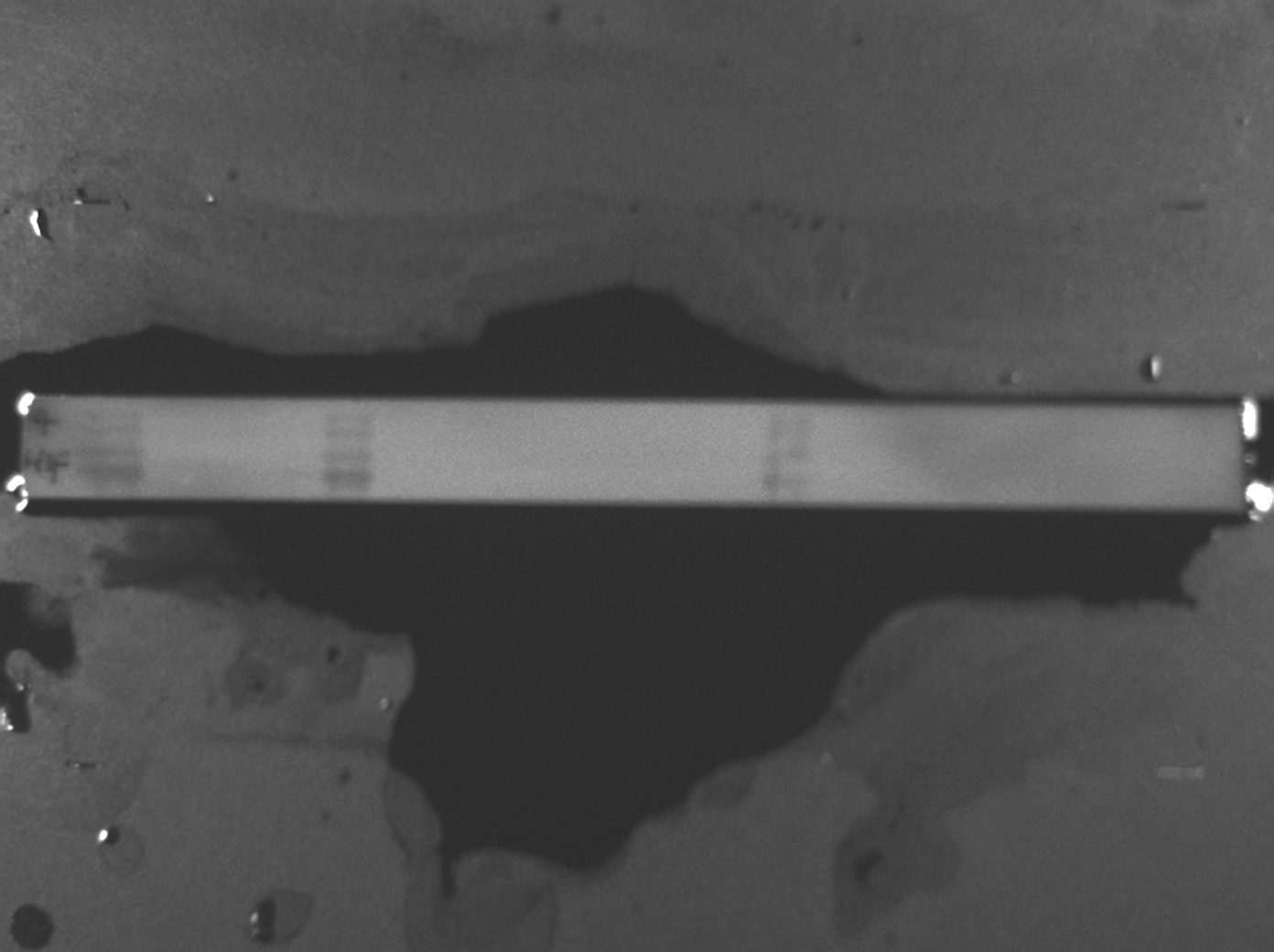

Supplement: Supplementary file 4 [file Data_Sheet_4.ZIP › 4-hif-2.tif]

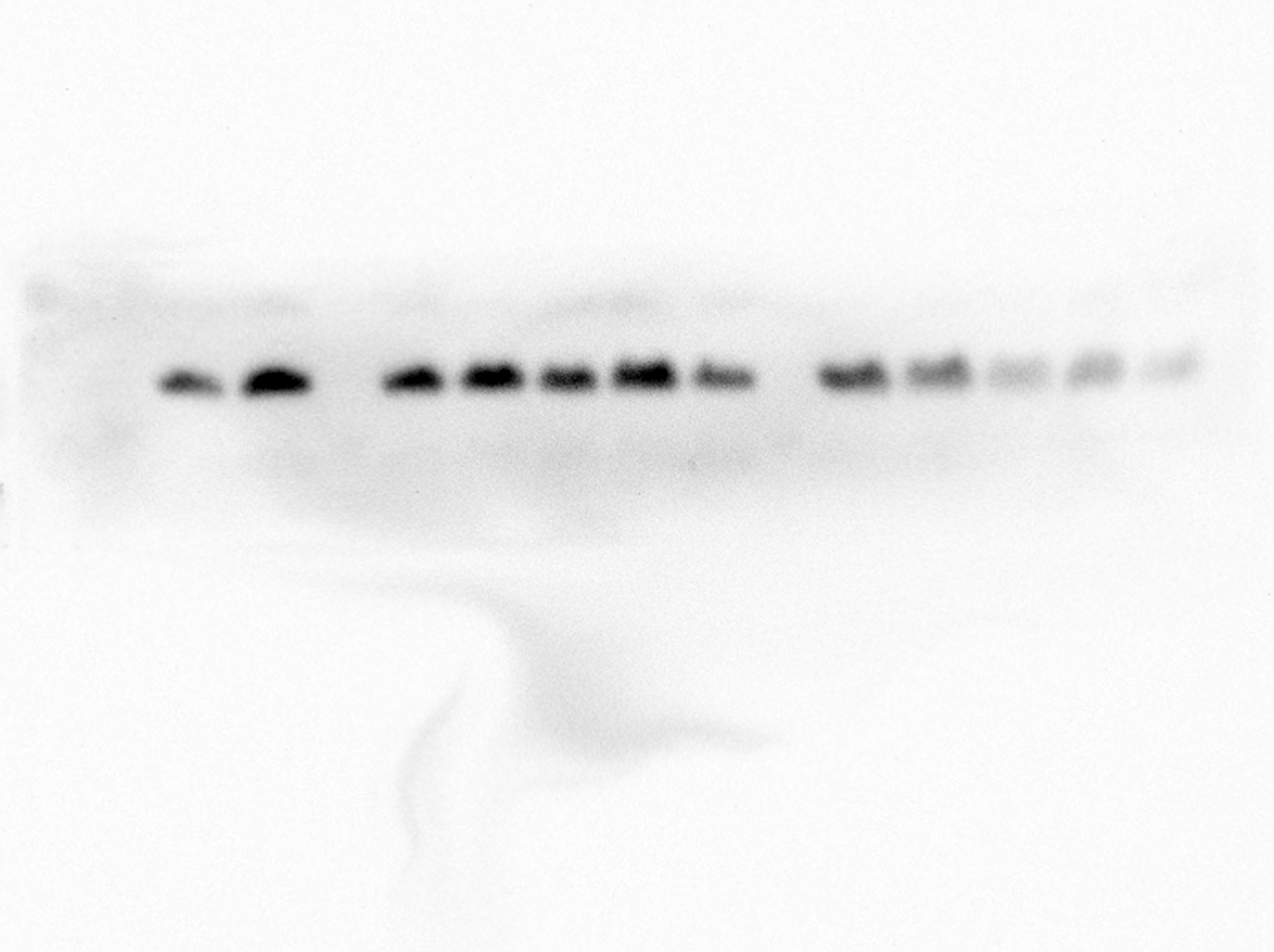

Supplement: Supplementary file 4 [file Data_Sheet_4.ZIP › 5-bax-1.tif]

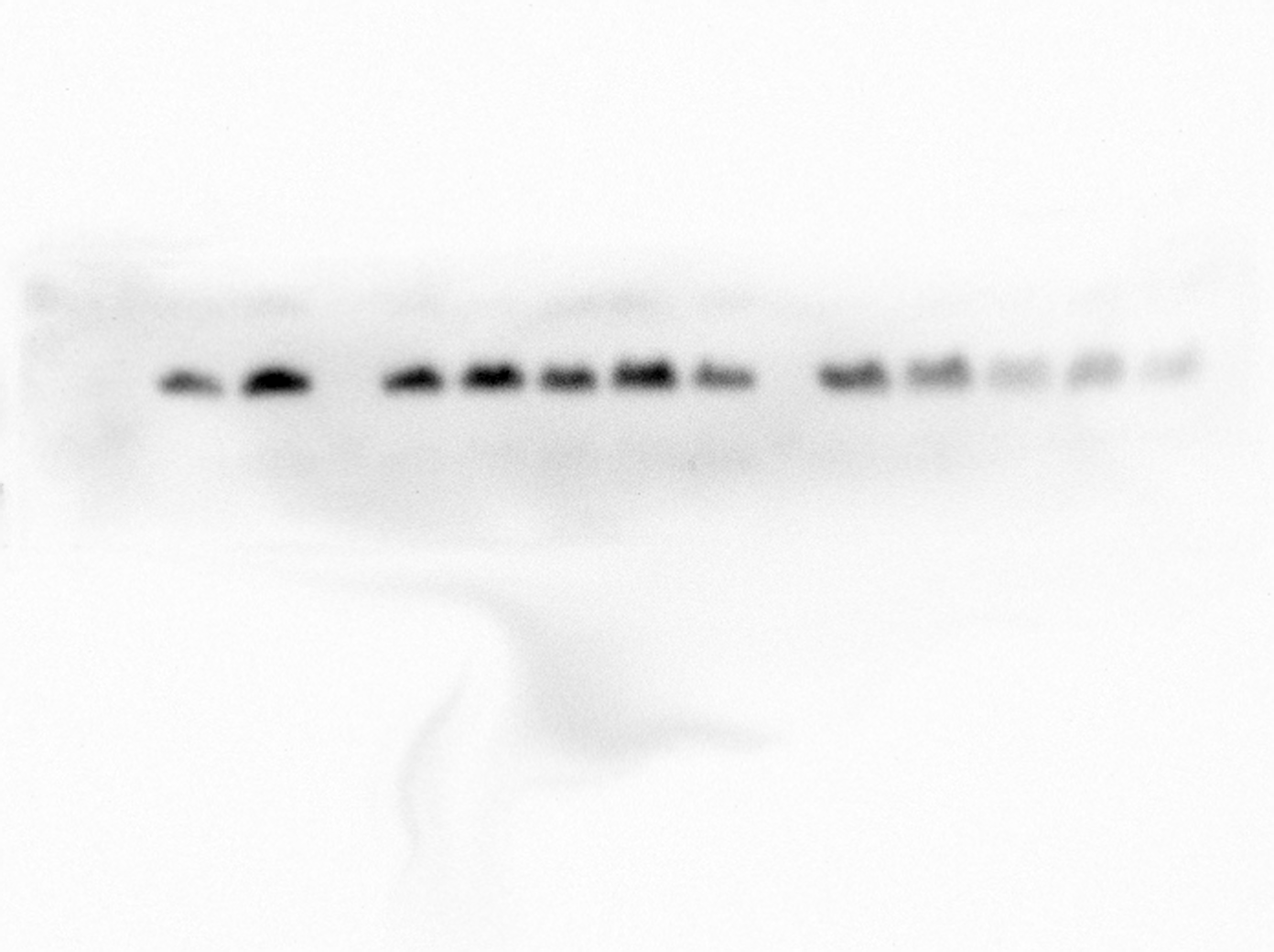

Supplement: Supplementary file 4 [file Data_Sheet_4.ZIP › 5-bax-2.tif]

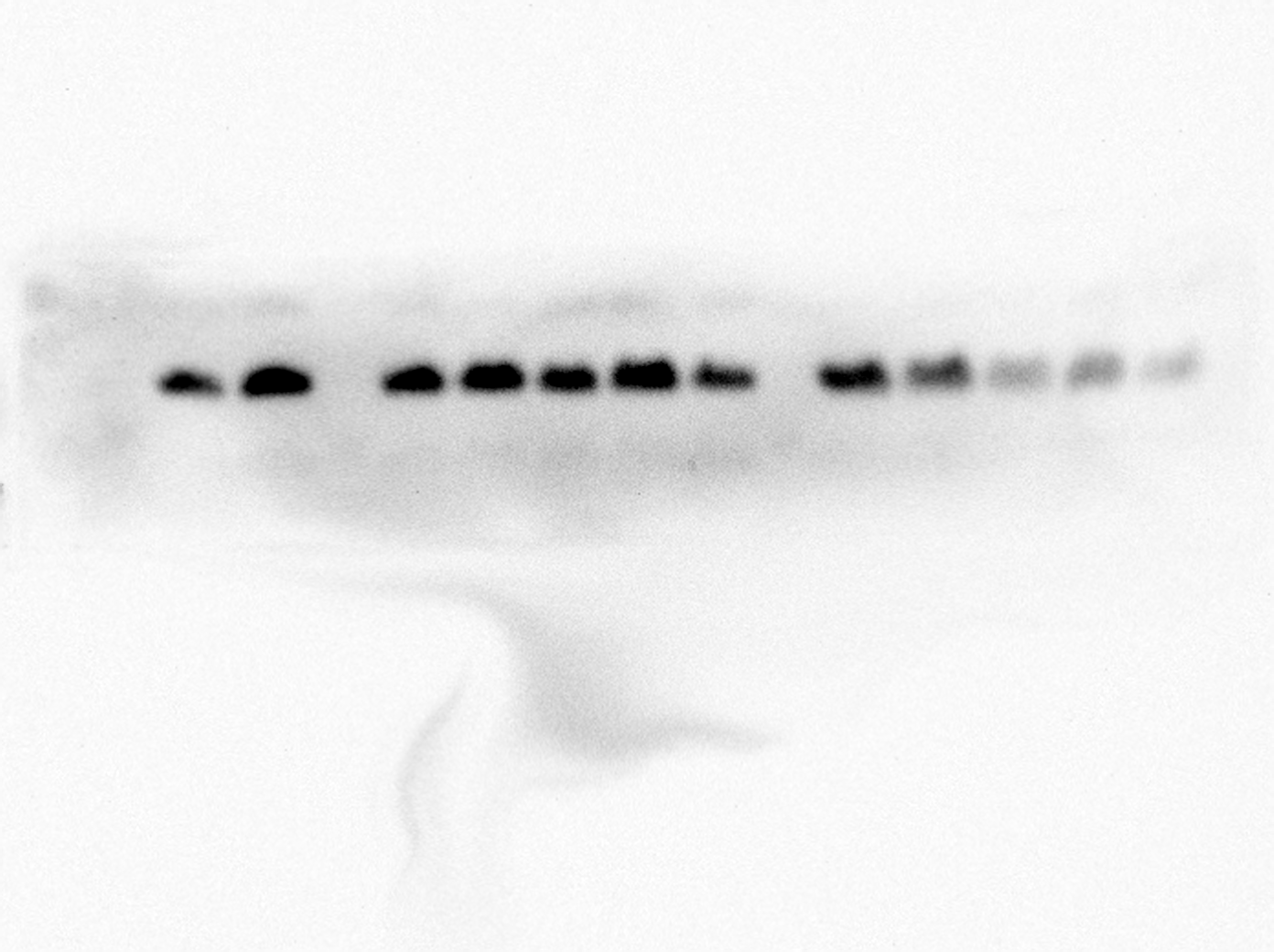

Supplement: Supplementary file 4 [file Data_Sheet_4.ZIP › 5-bax-3.tif]

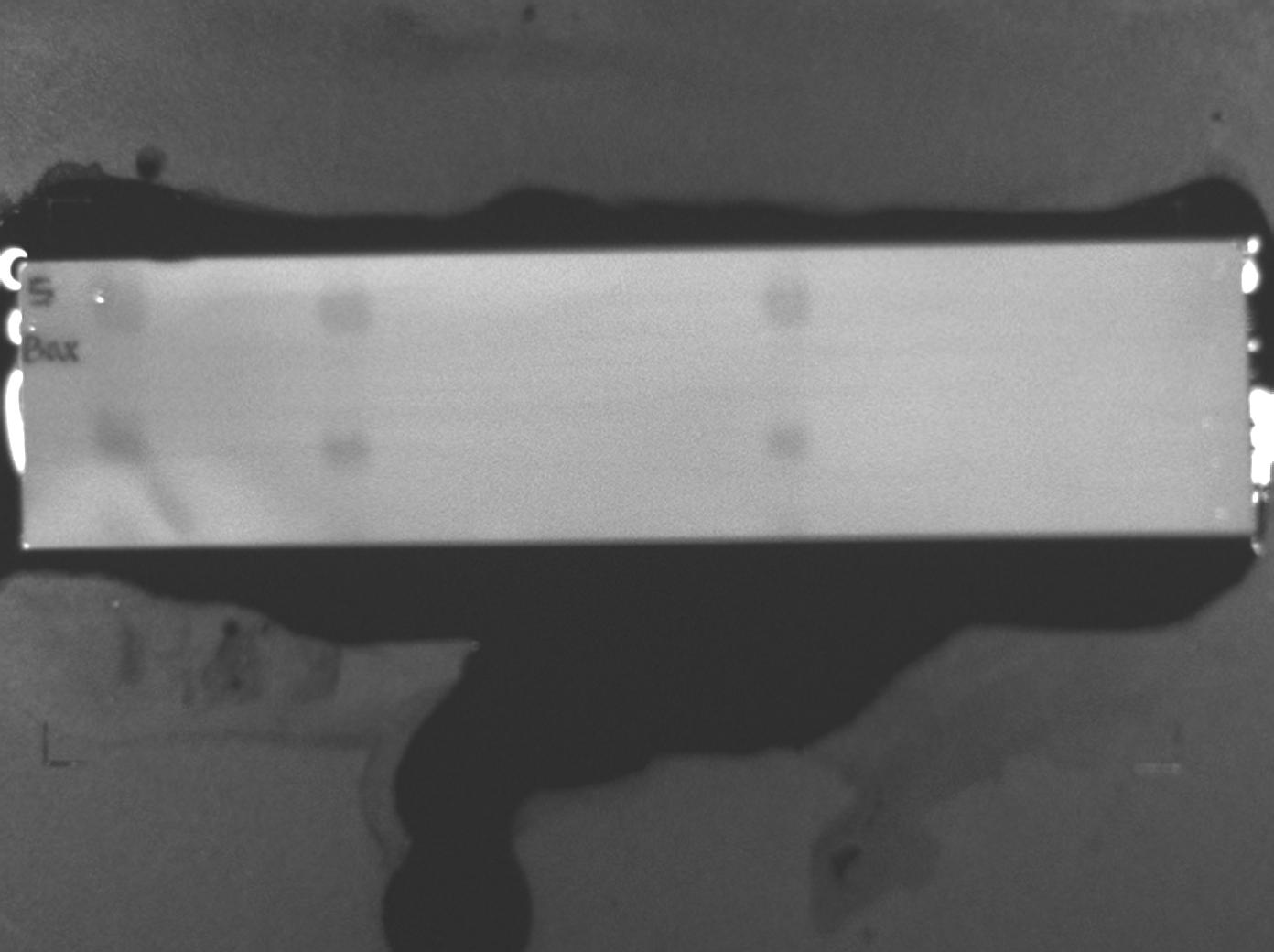

Supplement: Supplementary file 4 [file Data_Sheet_4.ZIP › 5-bax-4.tif]

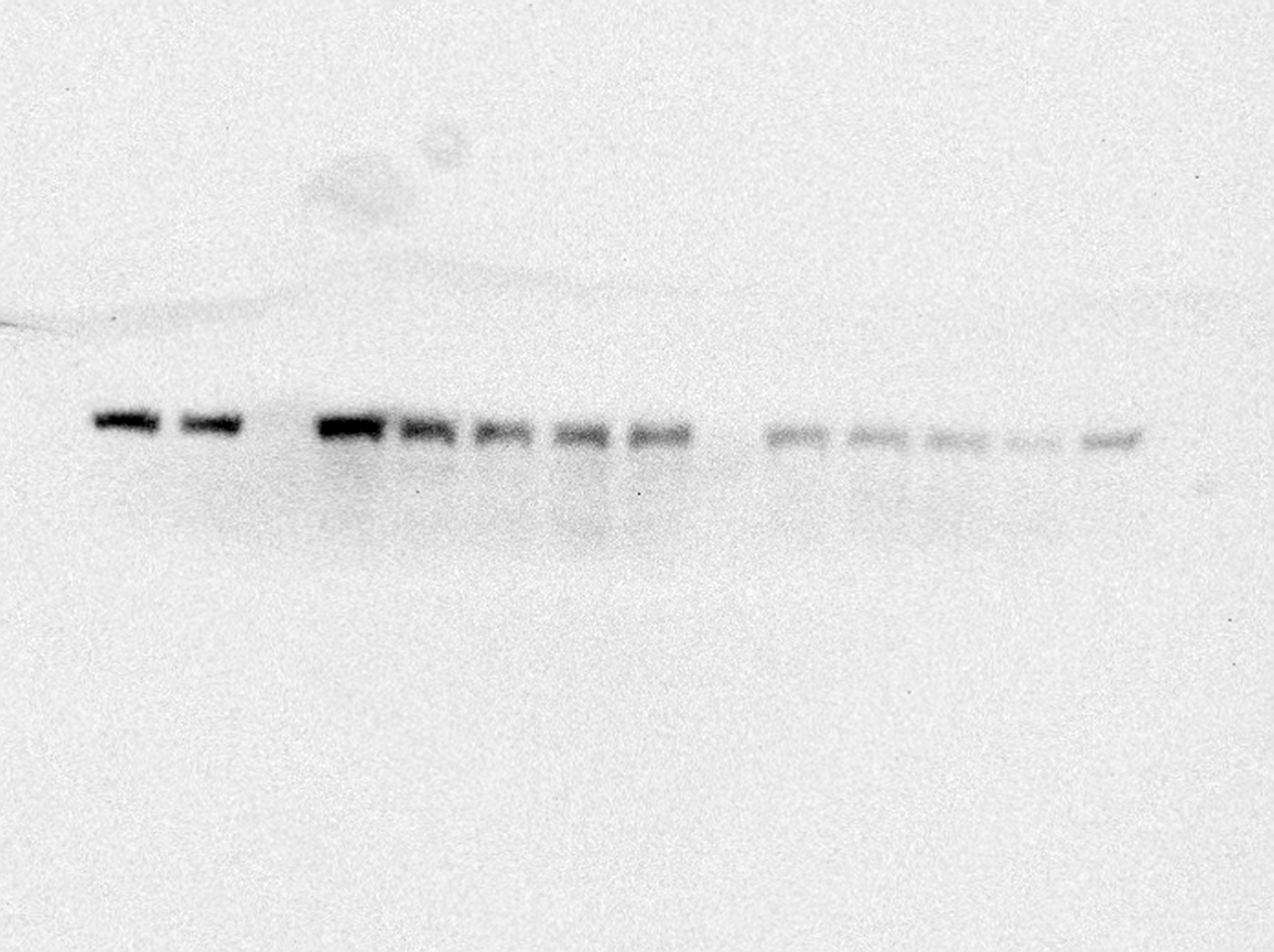

Supplement: Supplementary file 4 [file Data_Sheet_4.ZIP › 5-cas9-2.tif]

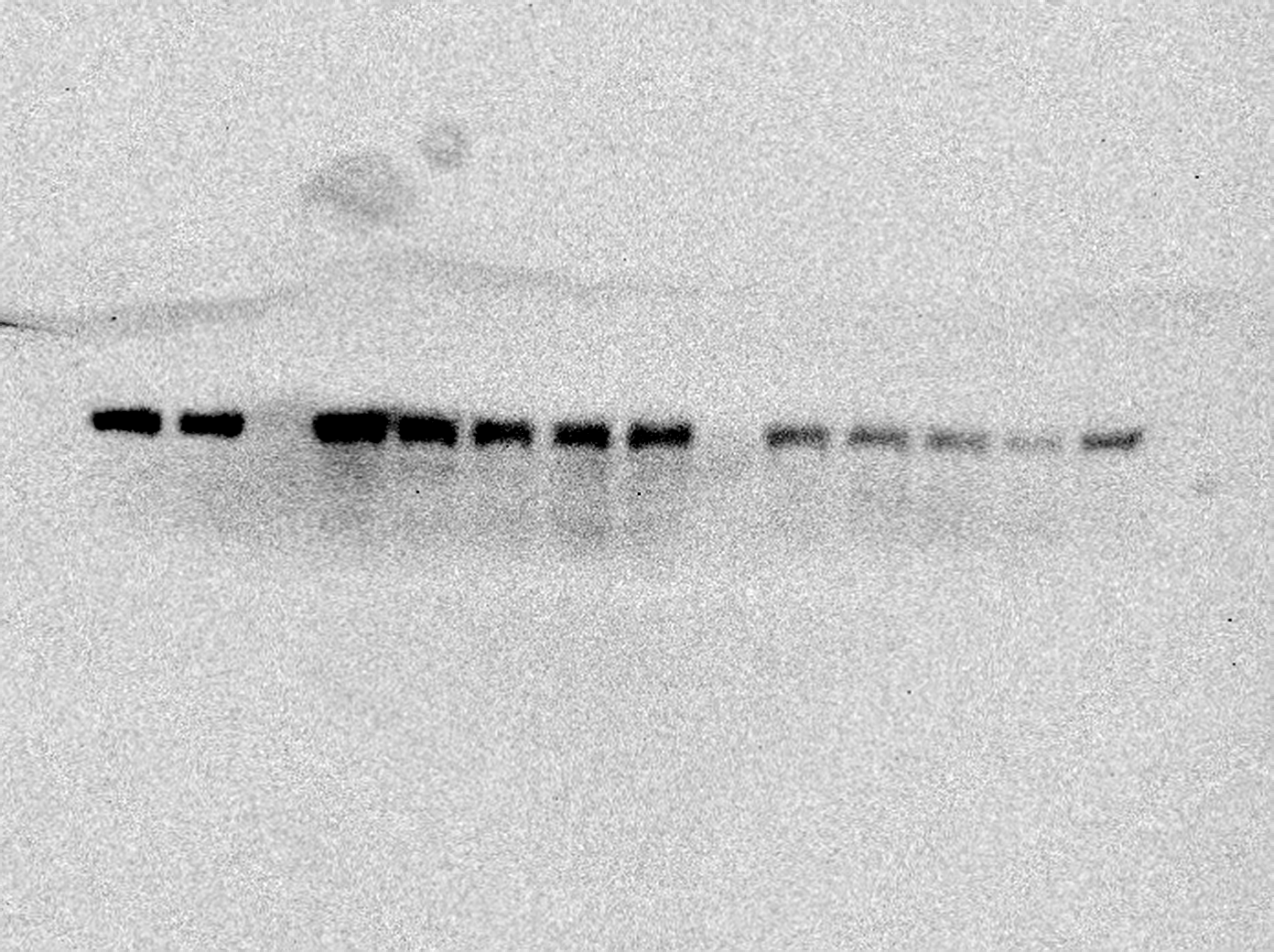

Supplement: Supplementary file 4 [file Data_Sheet_4.ZIP › 5-cas9-3.tif]

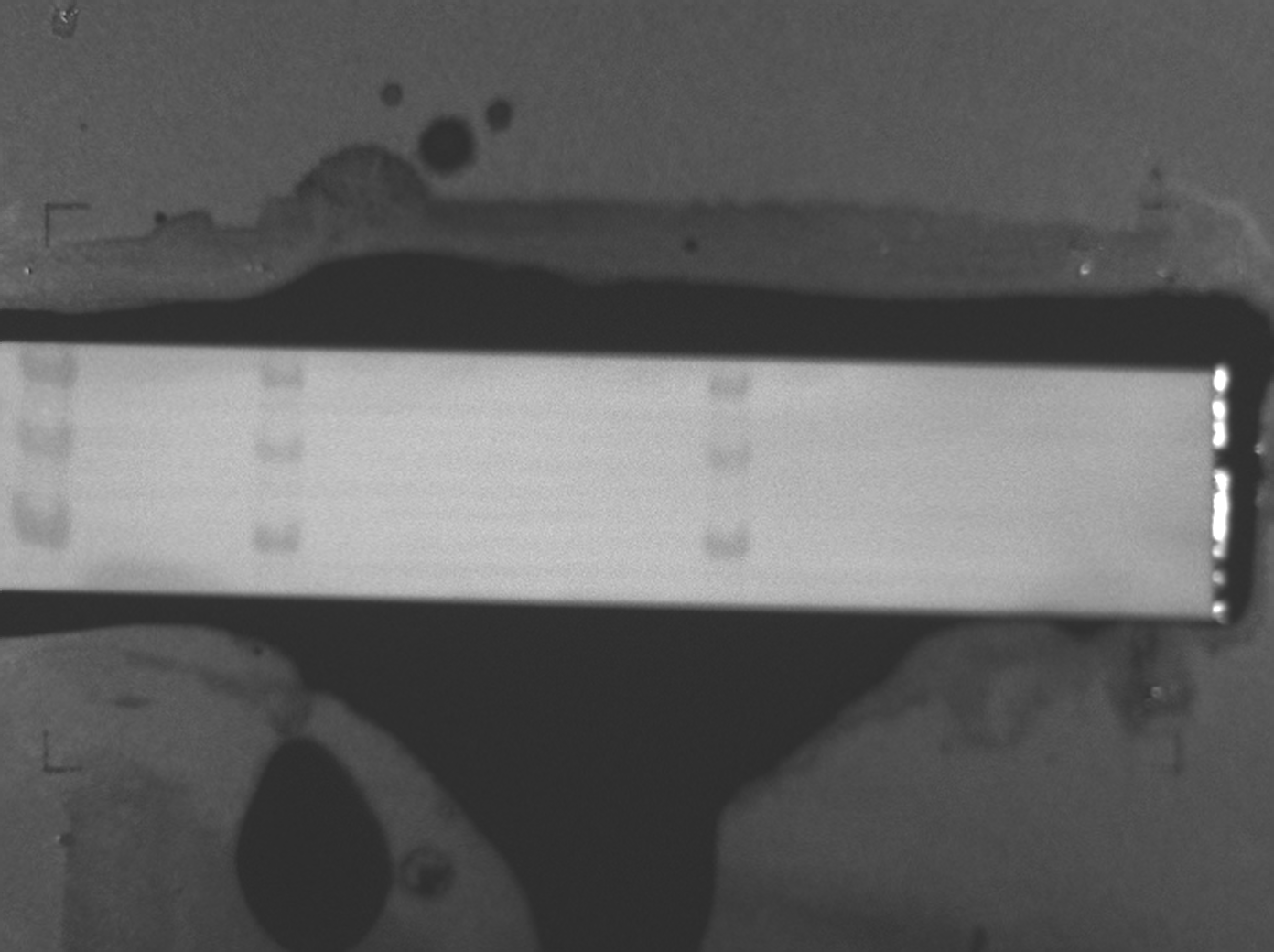

Supplement: Supplementary file 4 [file Data_Sheet_4.ZIP › 5-cas9-4.tif]

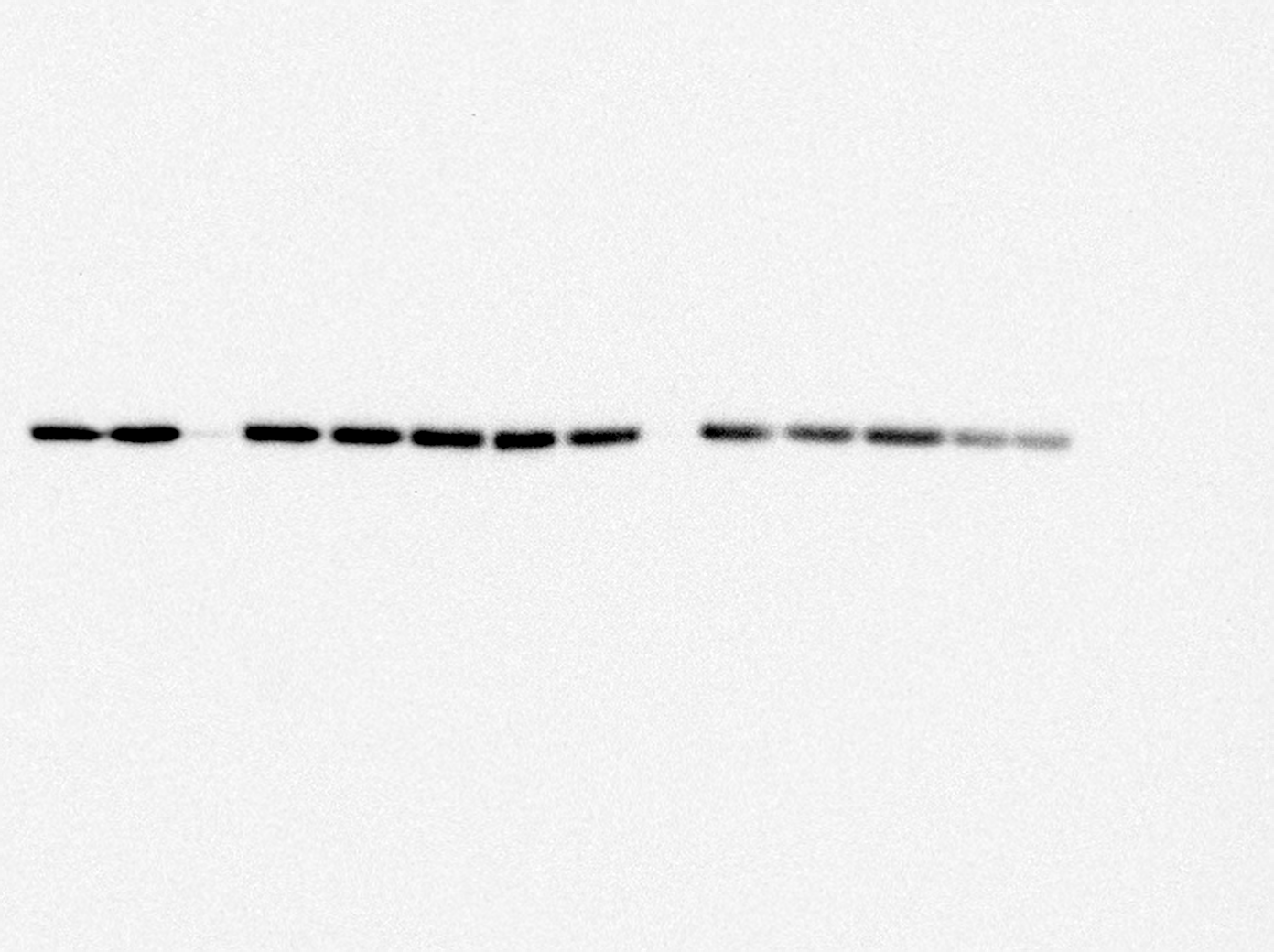

Supplement: Supplementary file 4 [file Data_Sheet_4.ZIP › 6-actin-1.tif]

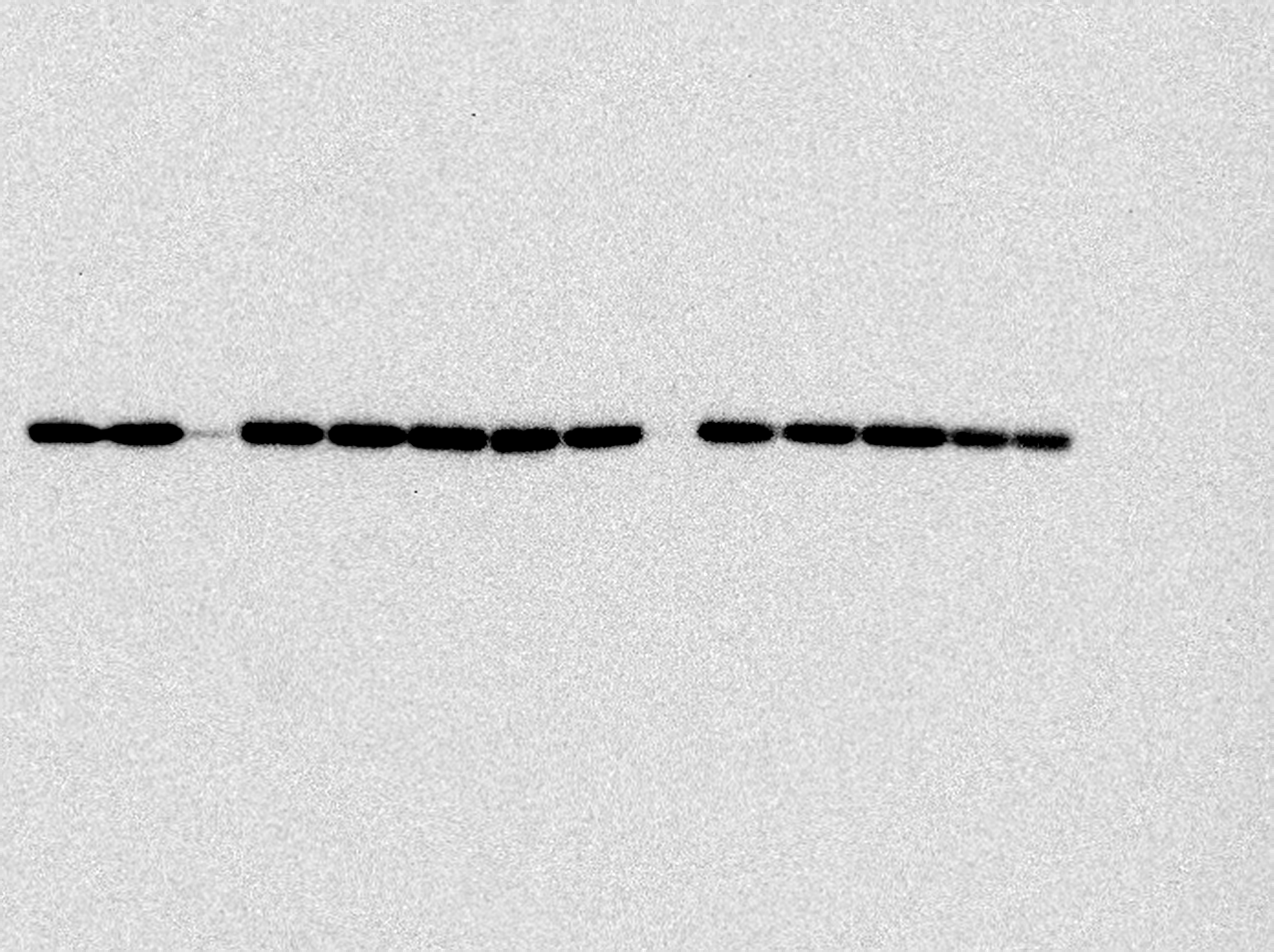

Supplement: Supplementary file 4 [file Data_Sheet_4.ZIP › 6-actin-2.tif]

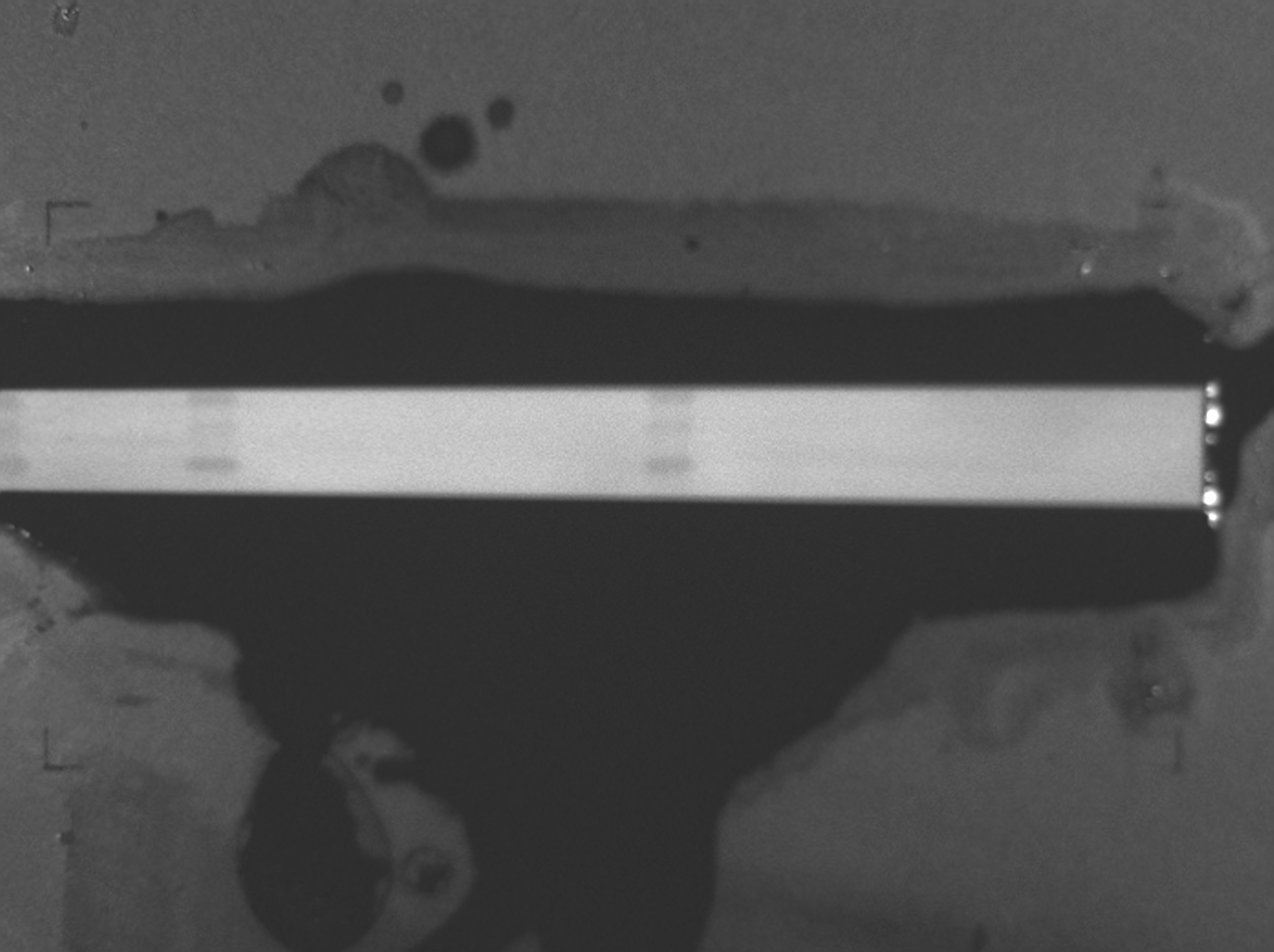

Supplement: Supplementary file 4 [file Data_Sheet_4.ZIP › 6-actin-3.tif]

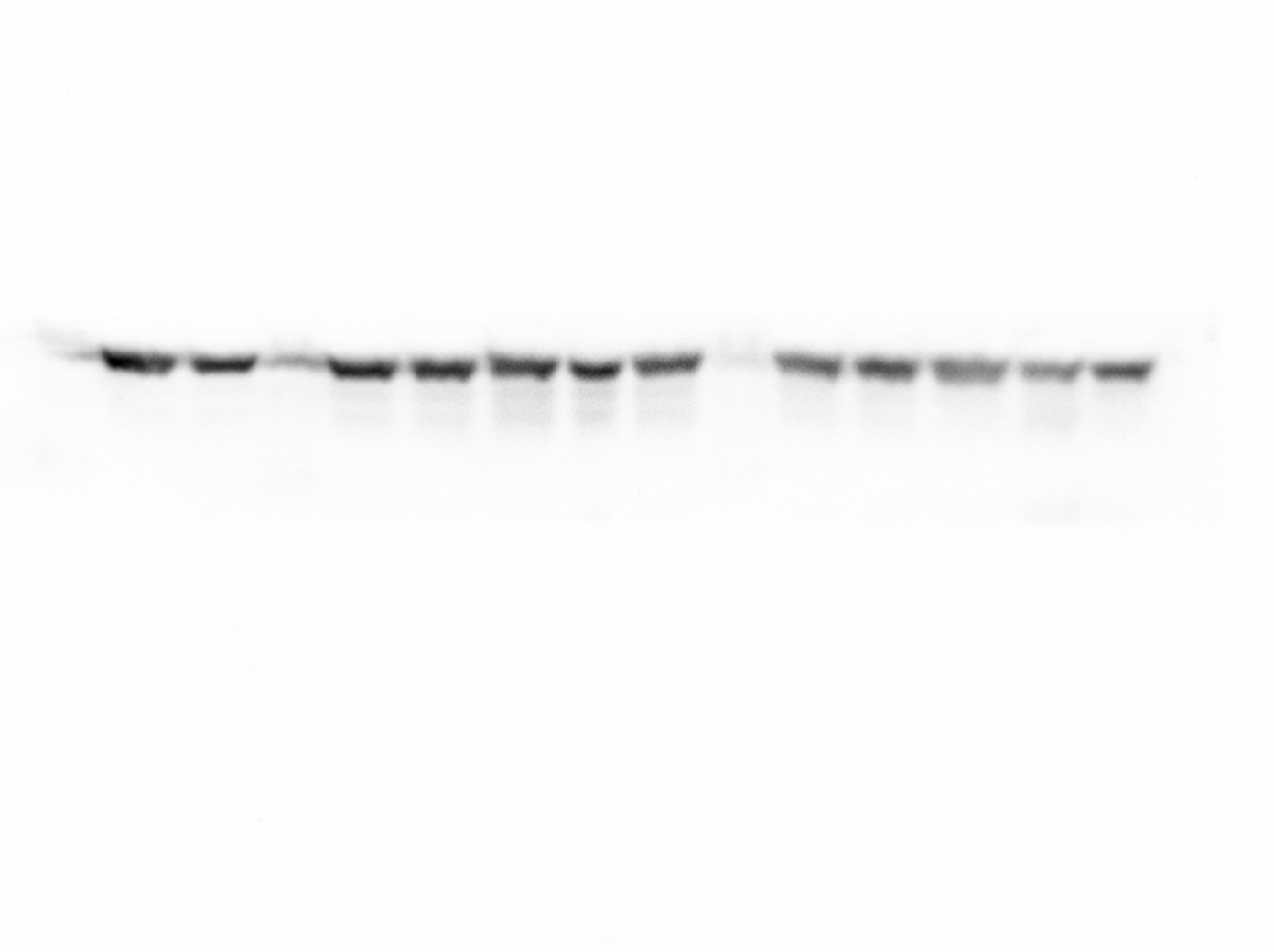

Supplement: Supplementary file 5 [file Data_Sheet_5.ZIP › 2-mlkl-1.tif]

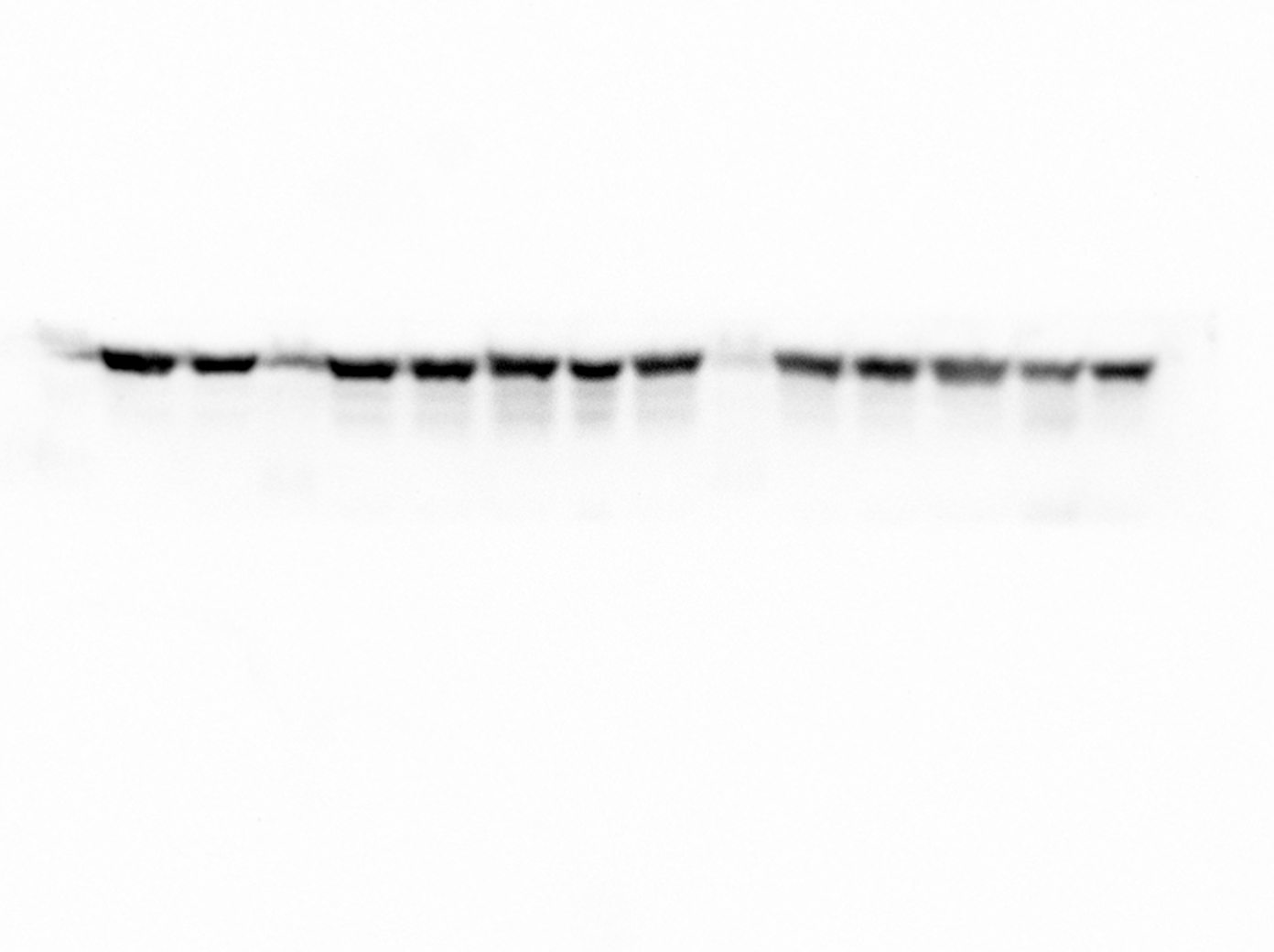

Supplement: Supplementary file 5 [file Data_Sheet_5.ZIP › 2-mlkl-3.tif]

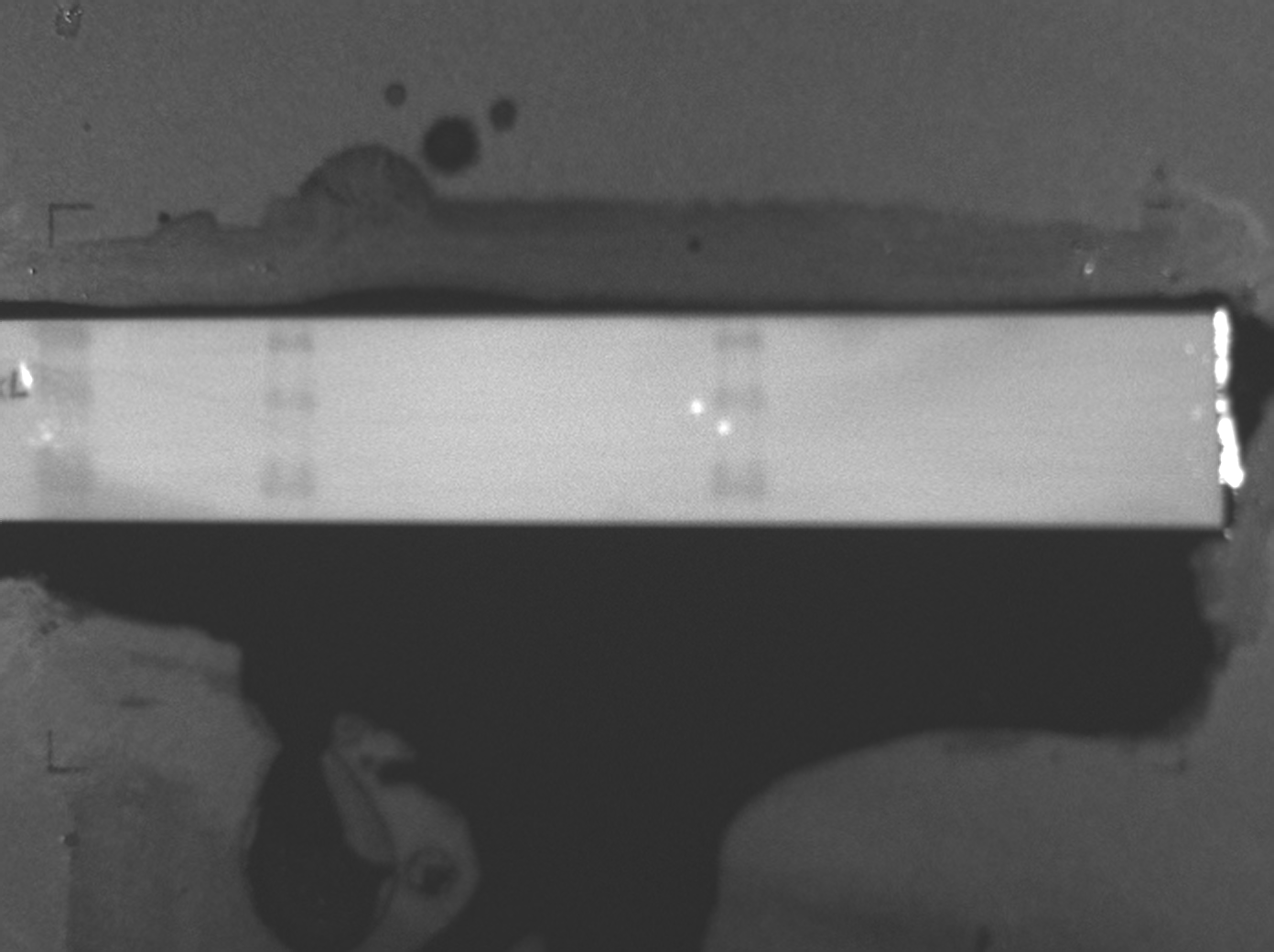

Supplement: Supplementary file 5 [file Data_Sheet_5.ZIP › 2-mlkl-4.tif]

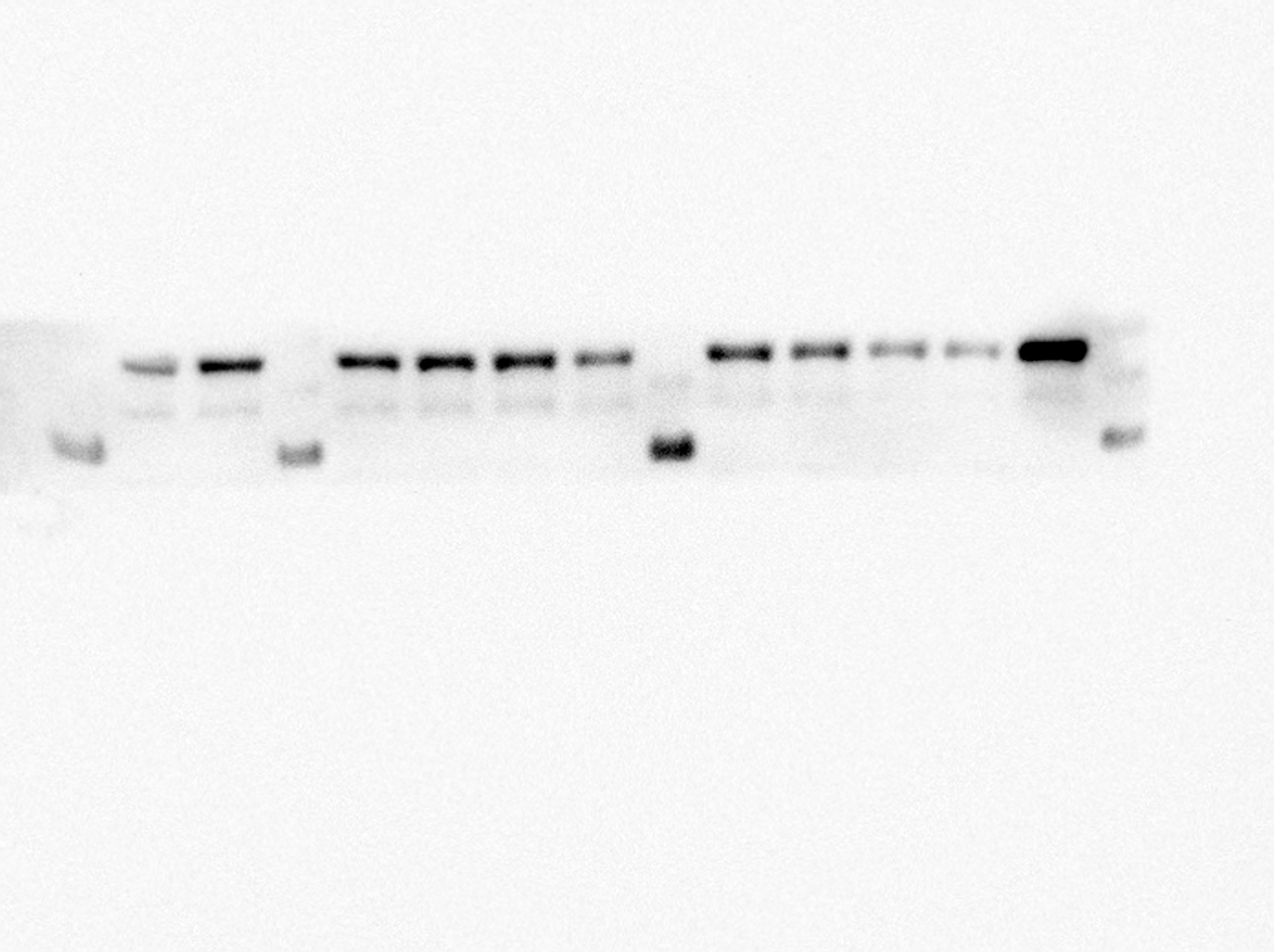

Supplement: Supplementary file 5 [file Data_Sheet_5.ZIP › 3 rip3-1.tif]

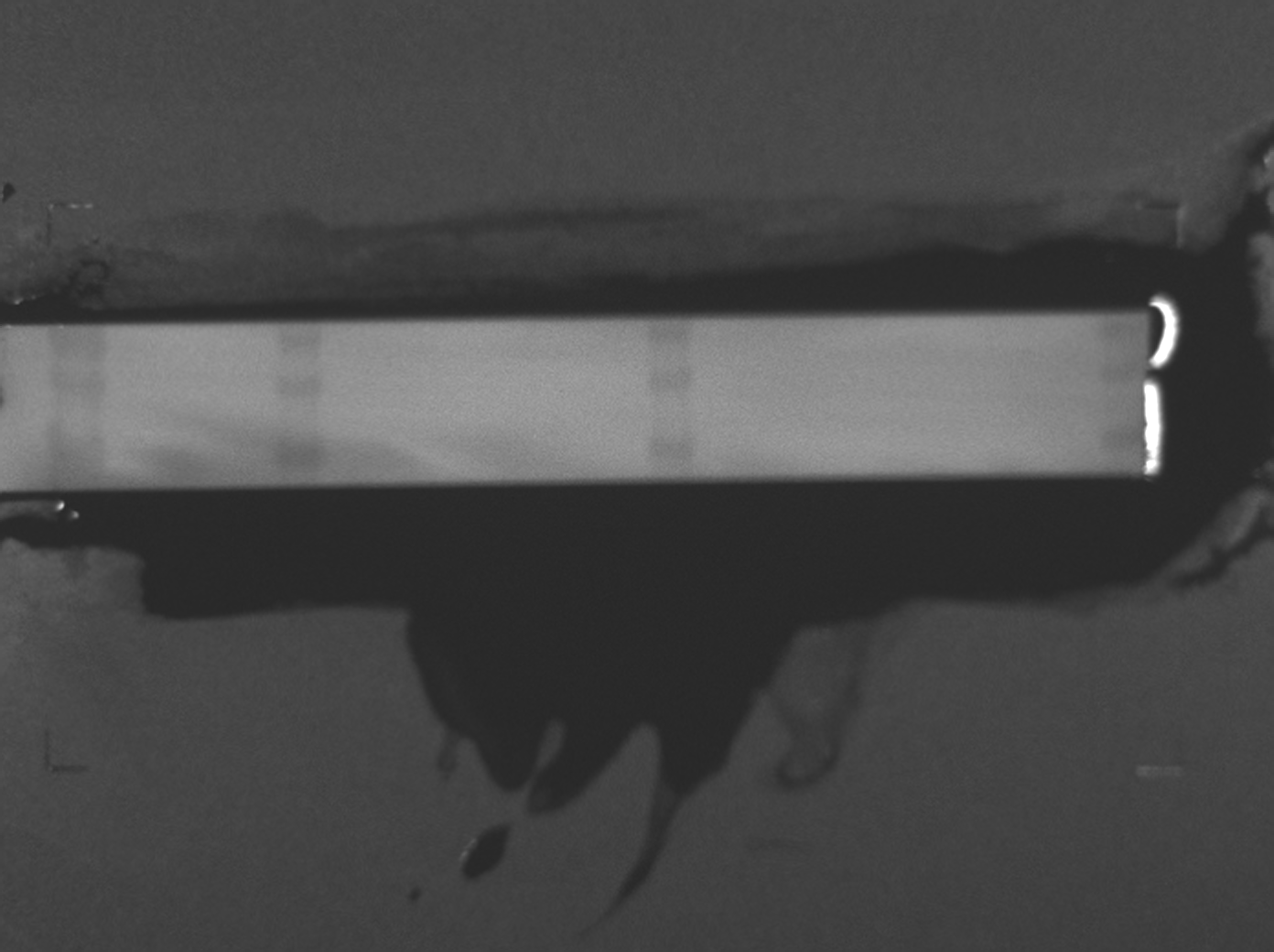

Supplement: Supplementary file 5 [file Data_Sheet_5.ZIP › 3 rip3-2.tif]

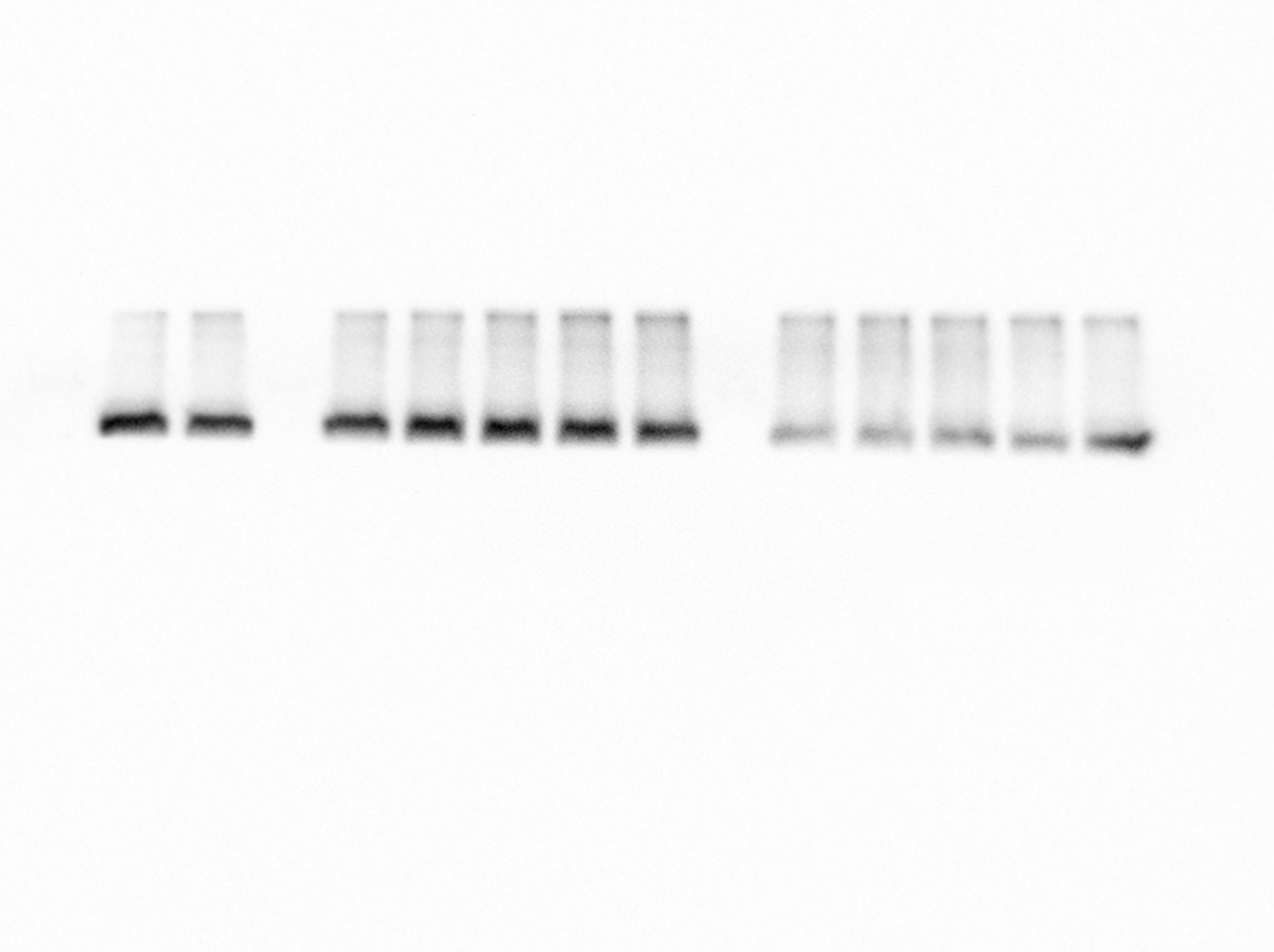

Supplement: Supplementary file 5 [file Data_Sheet_5.ZIP › 5-rip-1.tif]

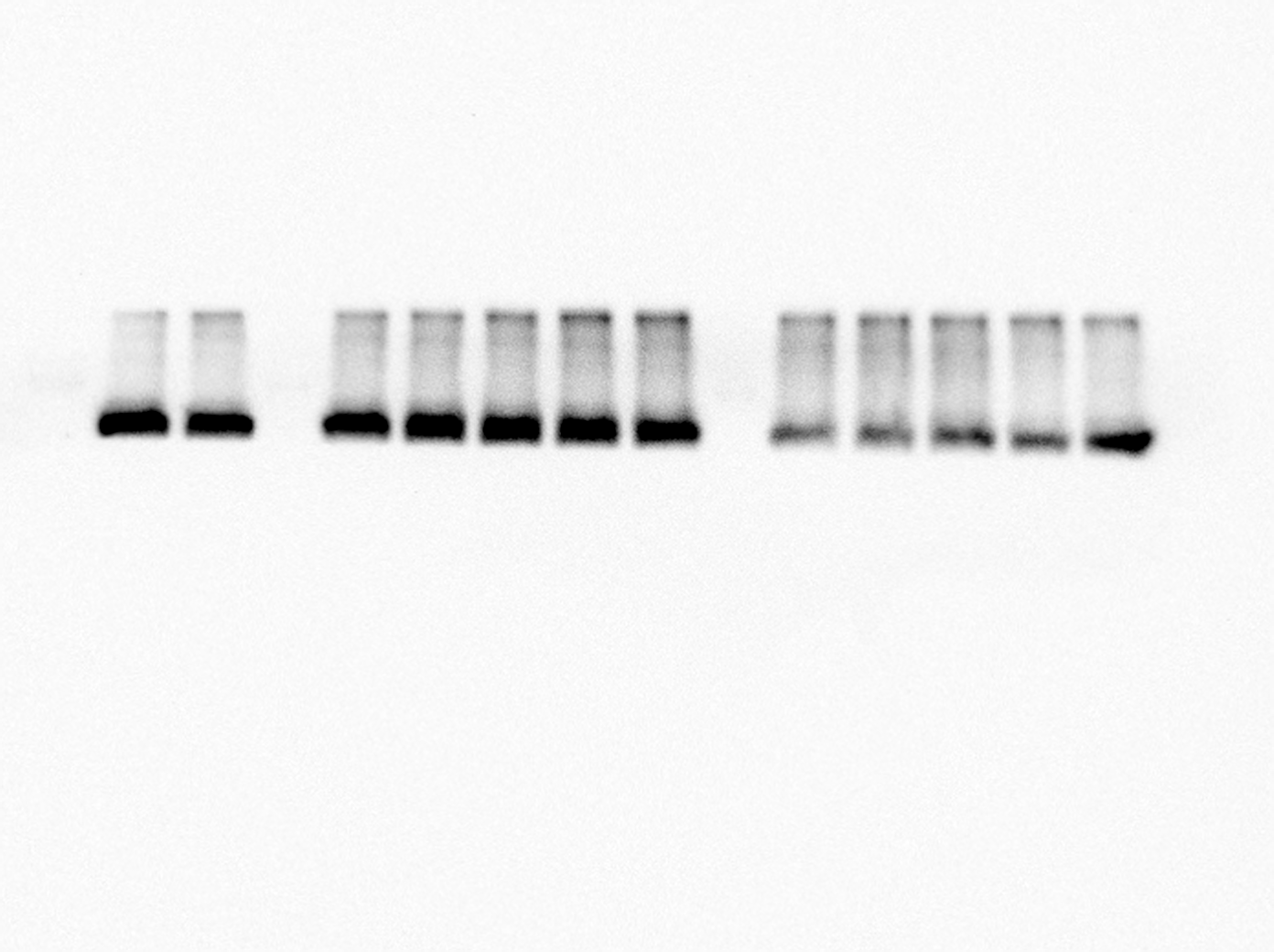

Supplement: Supplementary file 5 [file Data_Sheet_5.ZIP › 5-rip-3.tif]

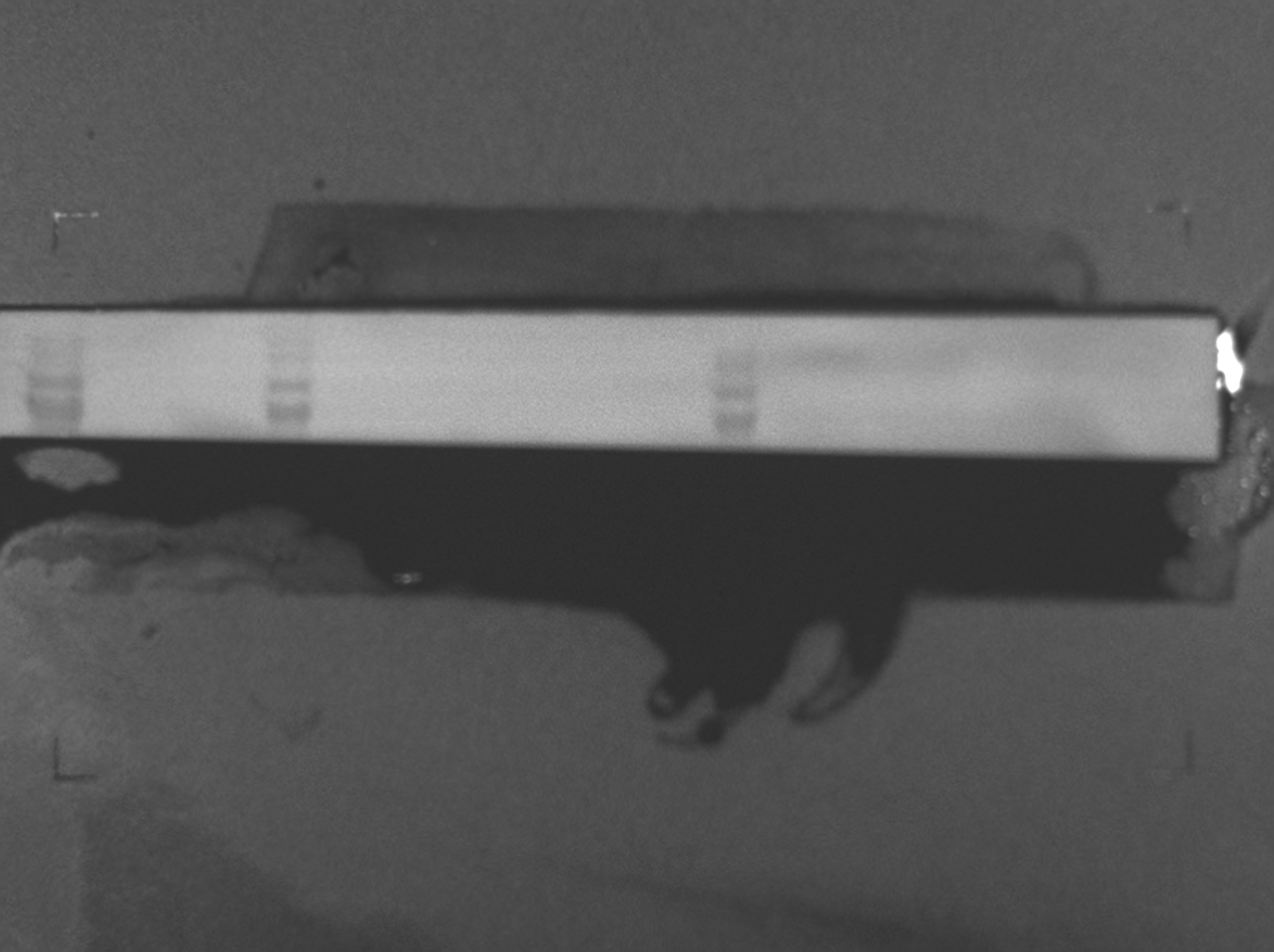

Supplement: Supplementary file 5 [file Data_Sheet_5.ZIP › 5-rip-4.tif]

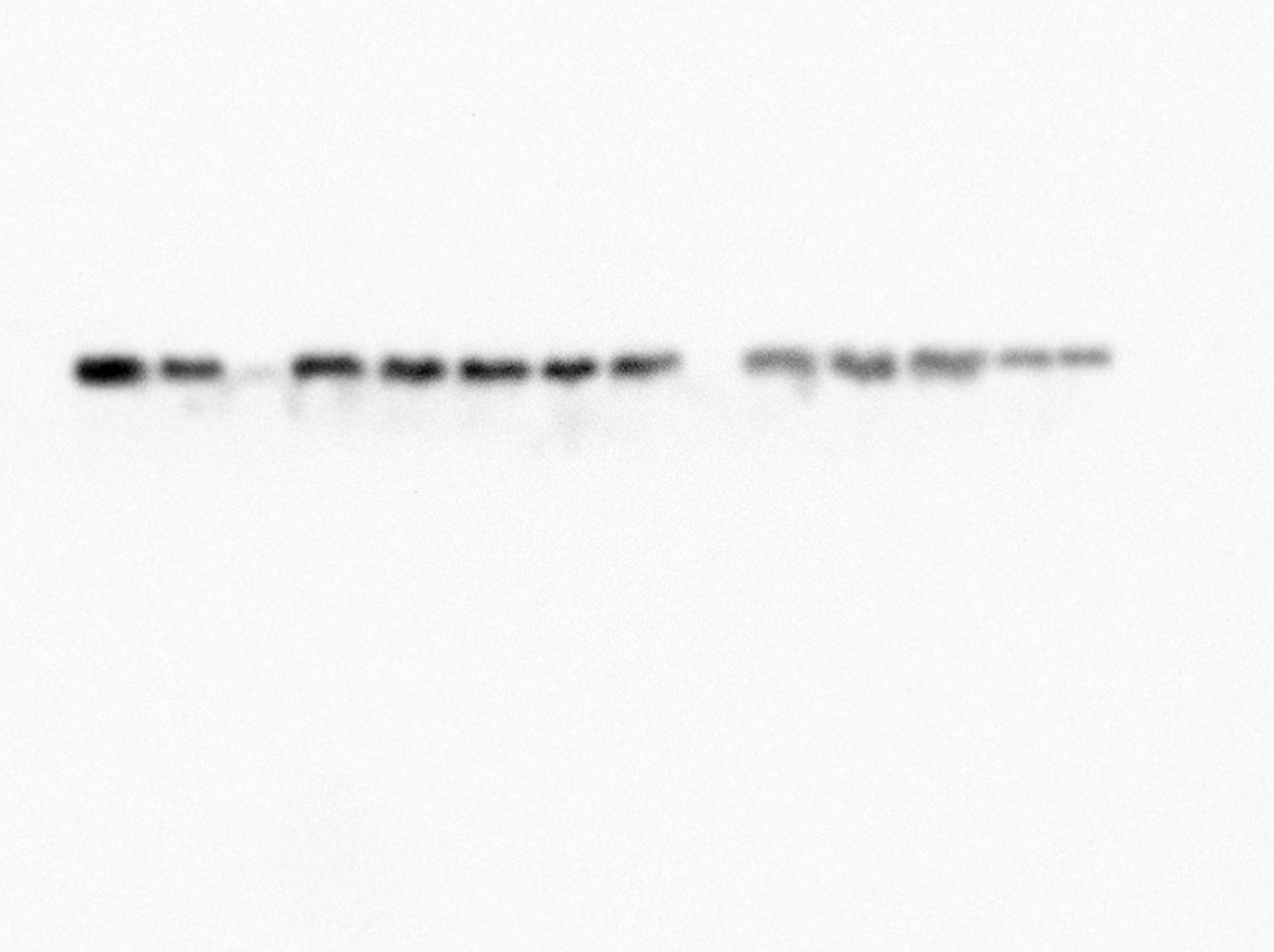

Supplement: Supplementary file 5 [file Data_Sheet_5.ZIP › 6-bcl2-1.tif]

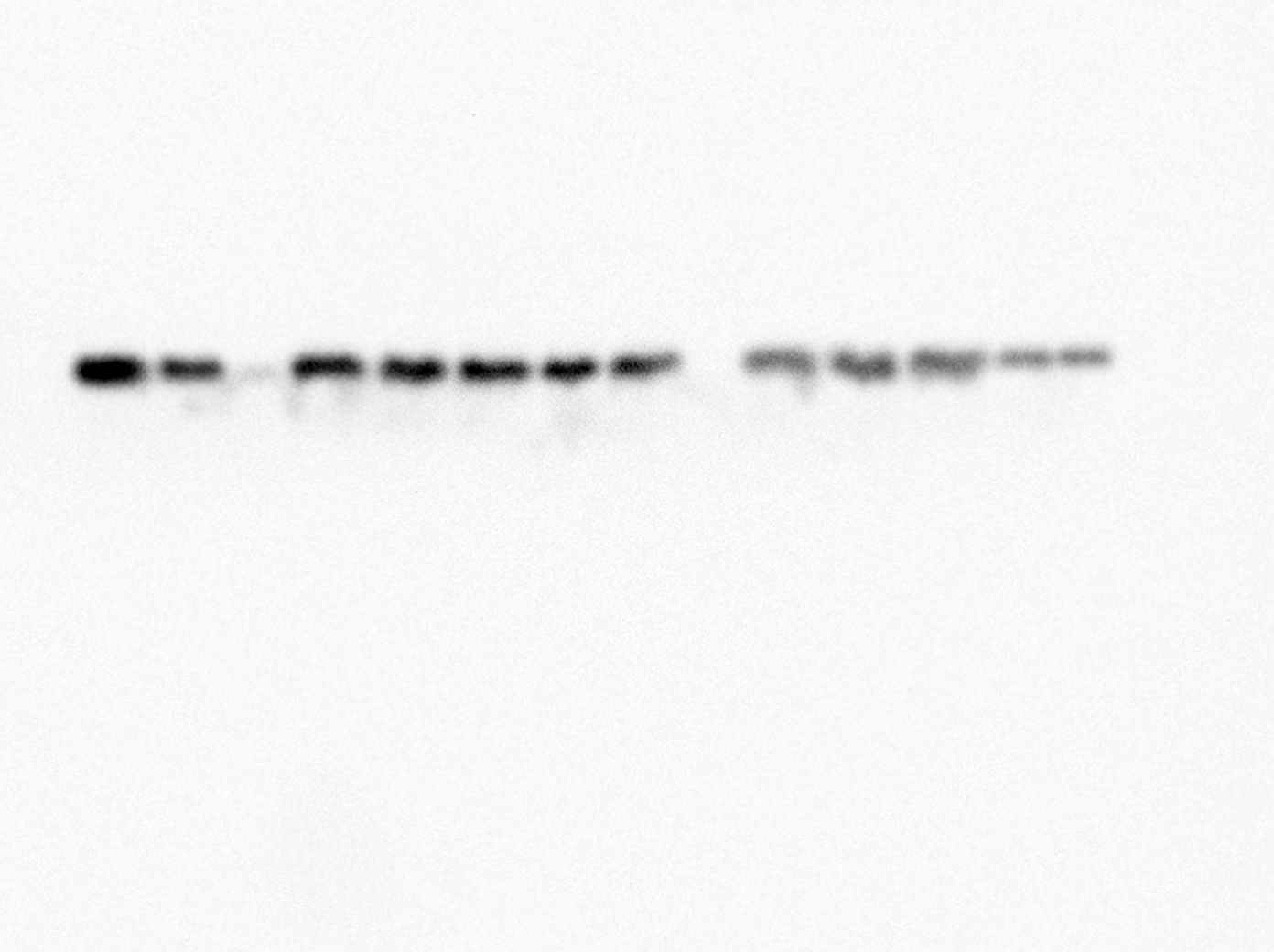

Supplement: Supplementary file 5 [file Data_Sheet_5.ZIP › 6-bcl2-2.tif]

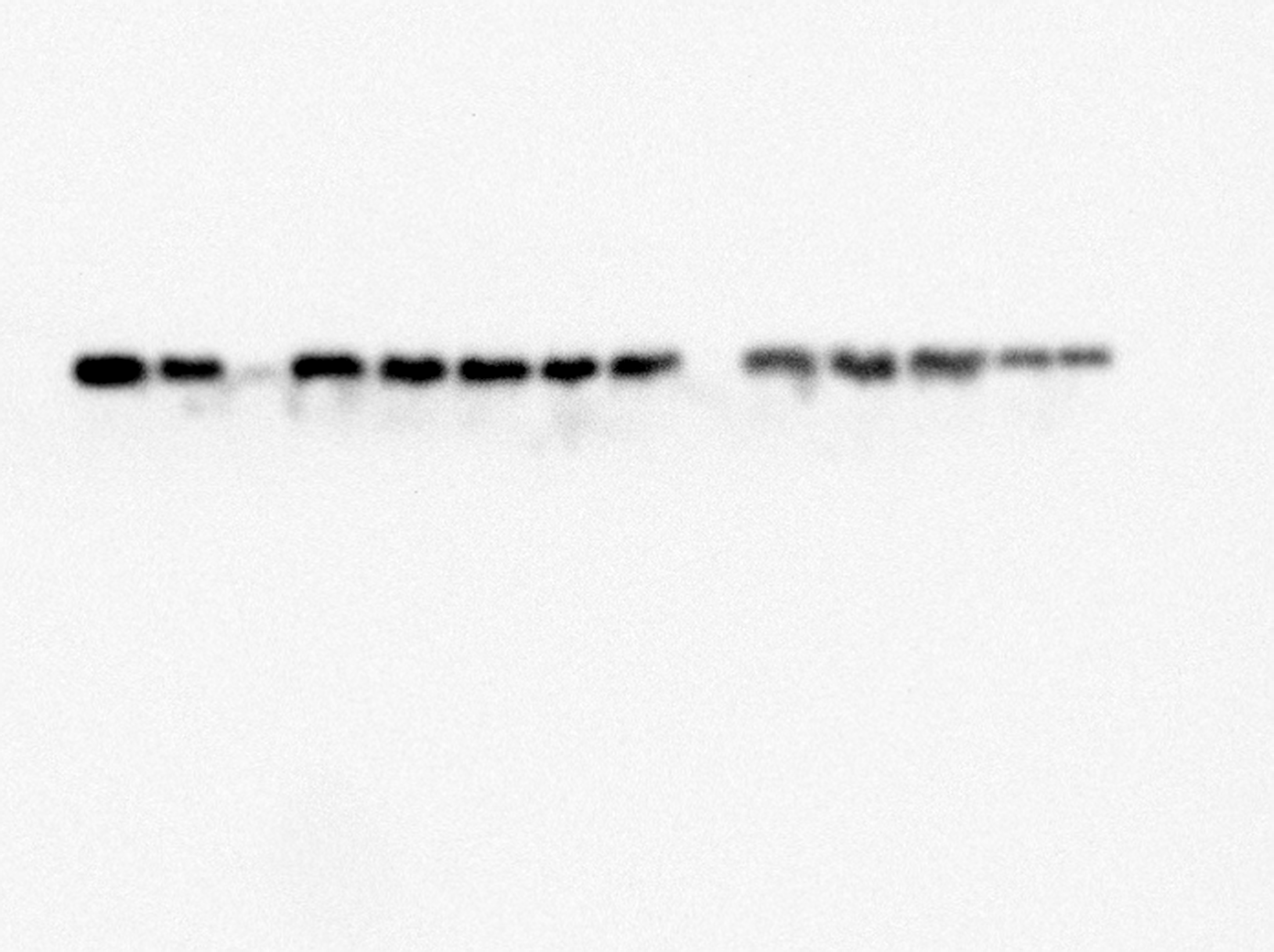

Supplement: Supplementary file 5 [file Data_Sheet_5.ZIP › 6-bcl2-3.tif]

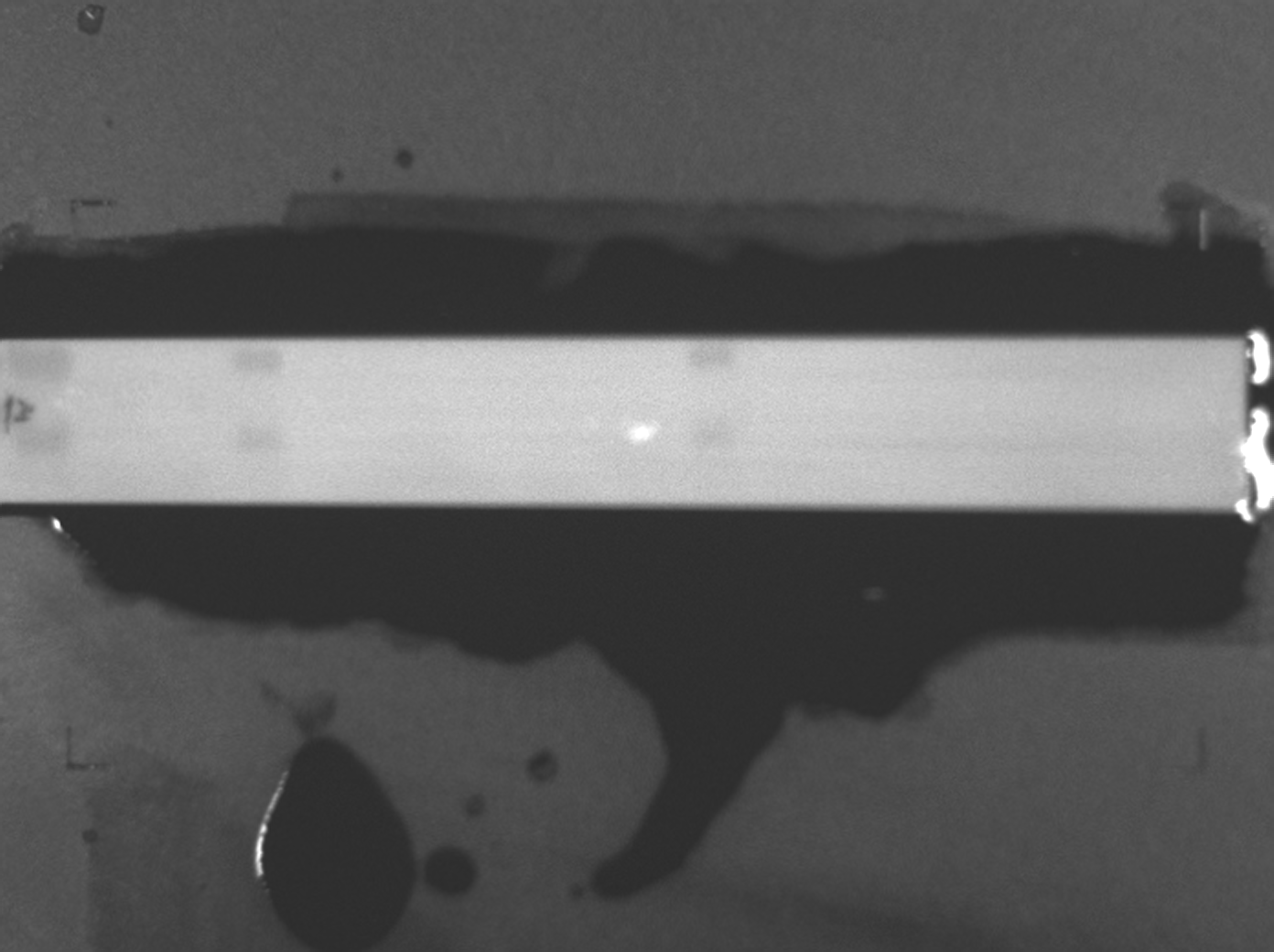

Supplement: Supplementary file 5 [file Data_Sheet_5.ZIP › 6-bcl2-4.tif]

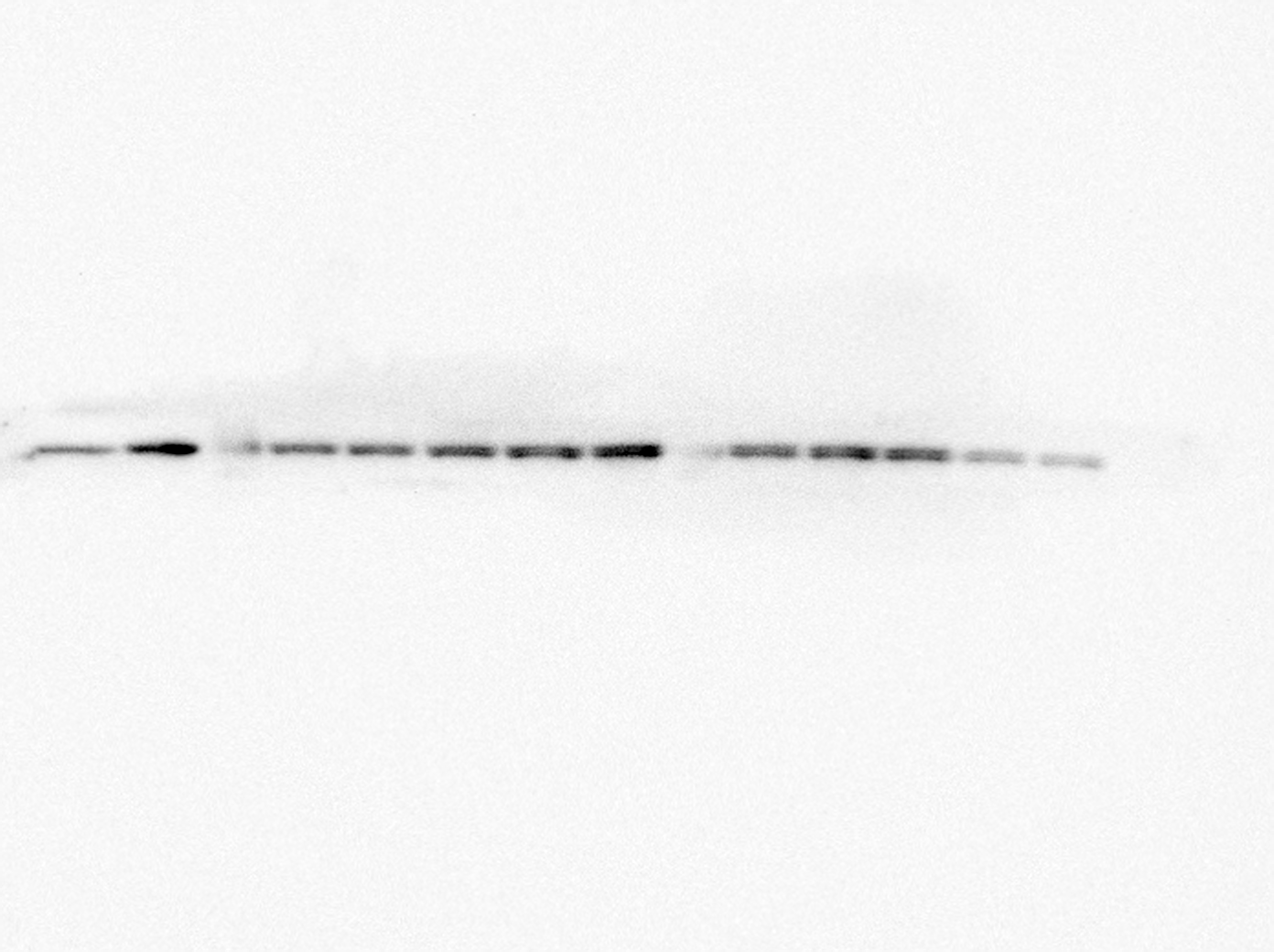

Supplement: Supplementary file 5 [file Data_Sheet_5.ZIP › 6-prip-1.tif]

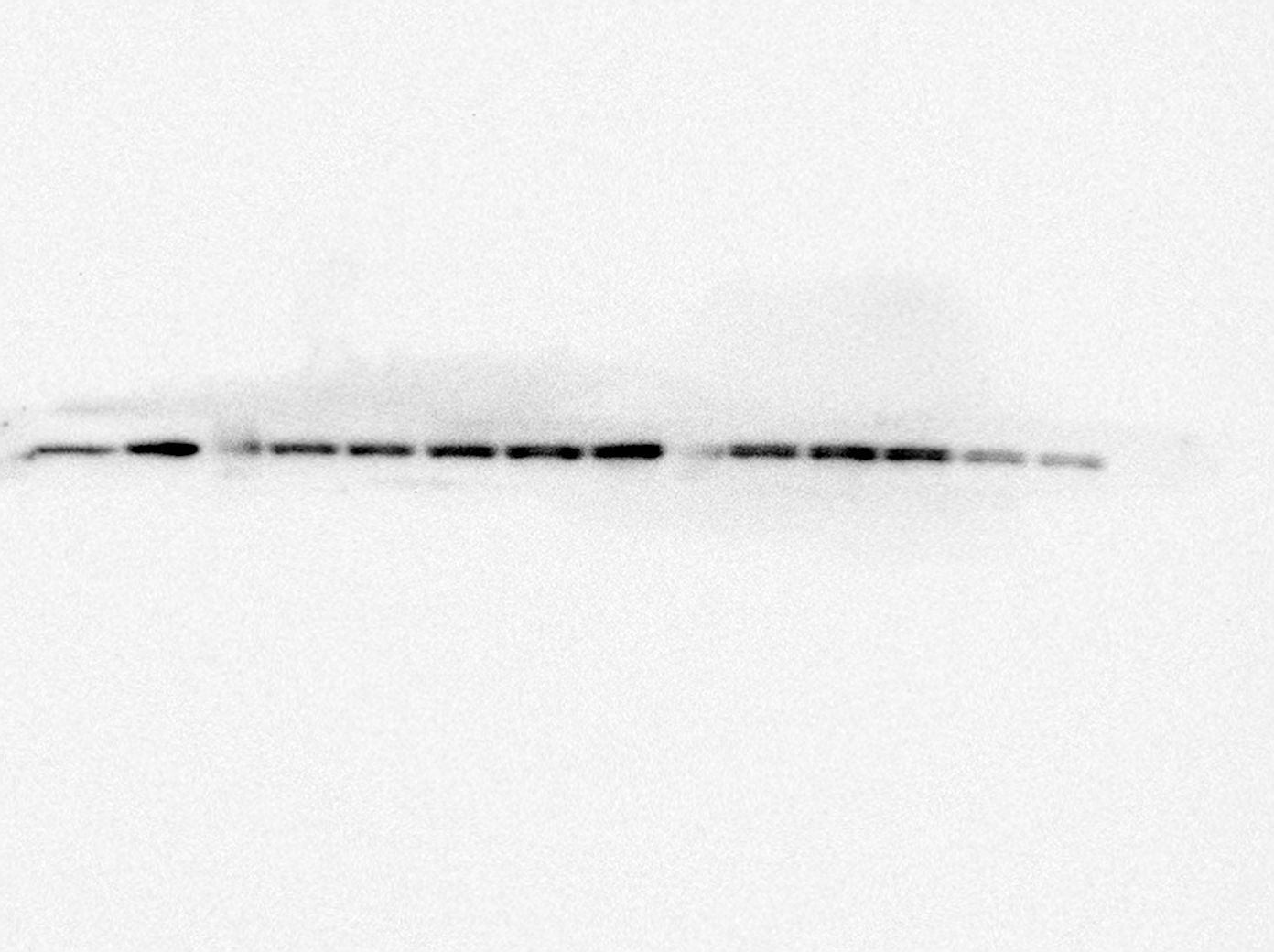

Supplement: Supplementary file 5 [file Data_Sheet_5.ZIP › 6-prip-2.tif]

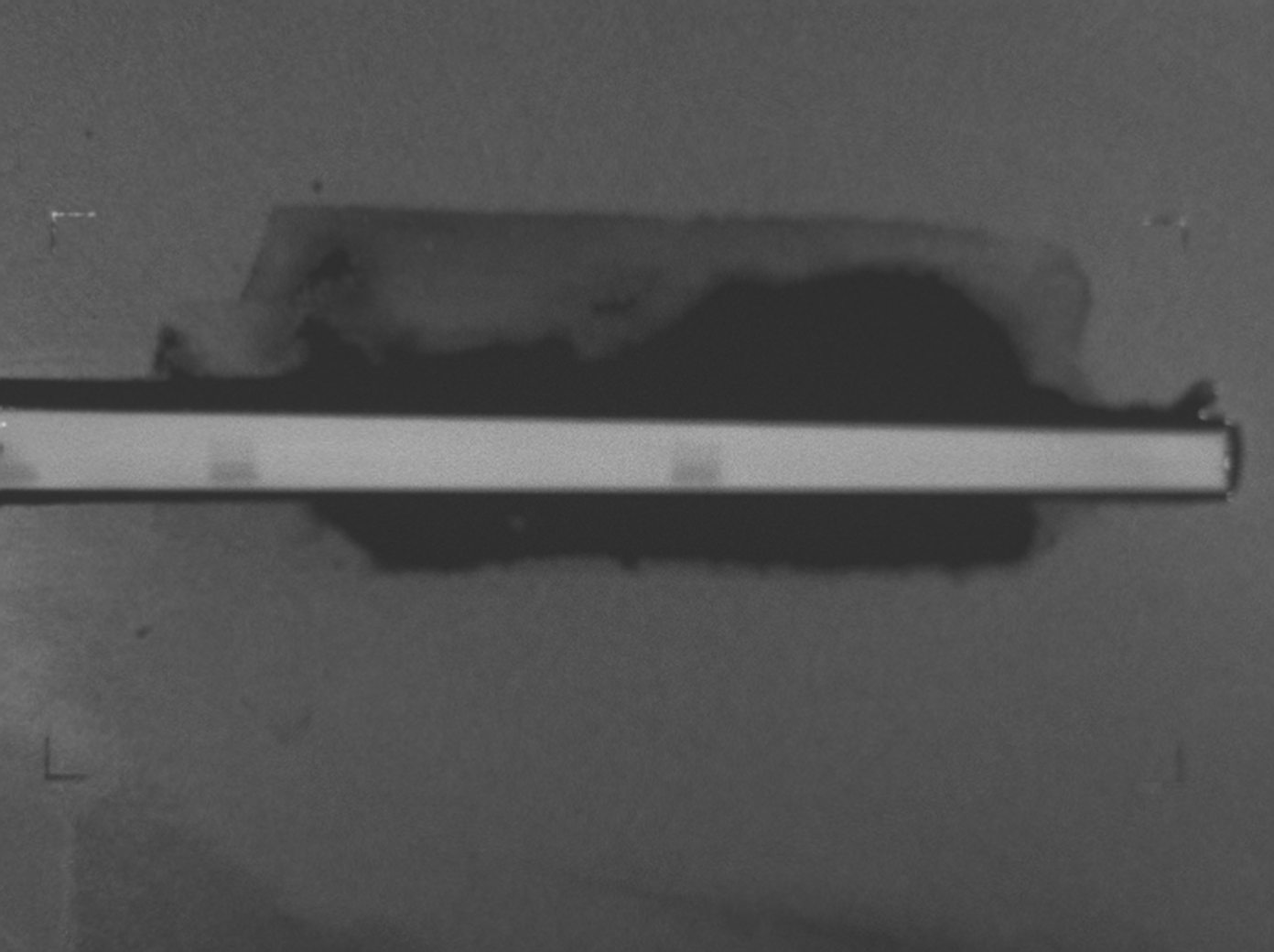

Supplement: Supplementary file 5 [file Data_Sheet_5.ZIP › 6-prip-3.tif]

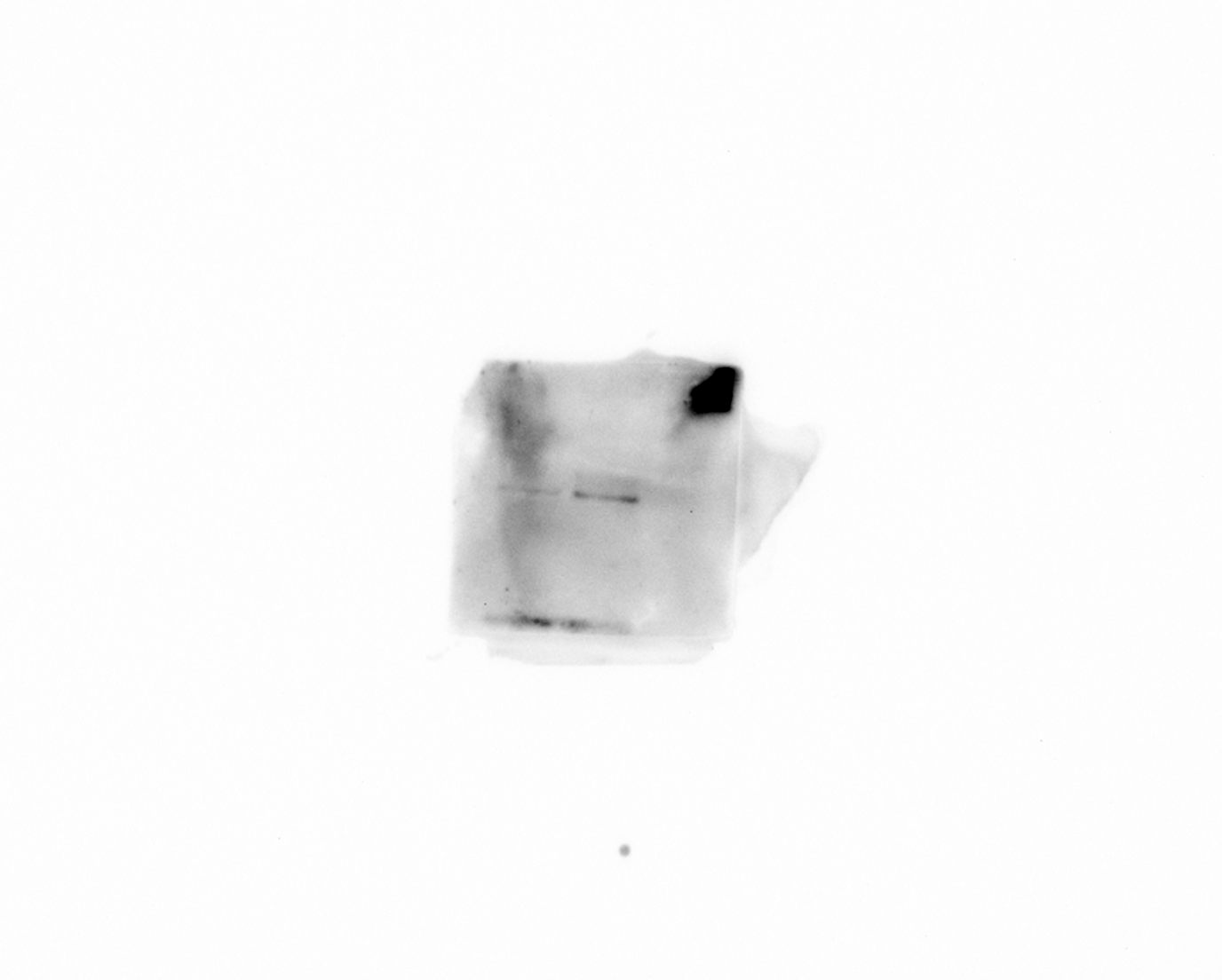

Supplement: Supplementary file 5 [file Data_Sheet_5.ZIP › prip3.Tif]

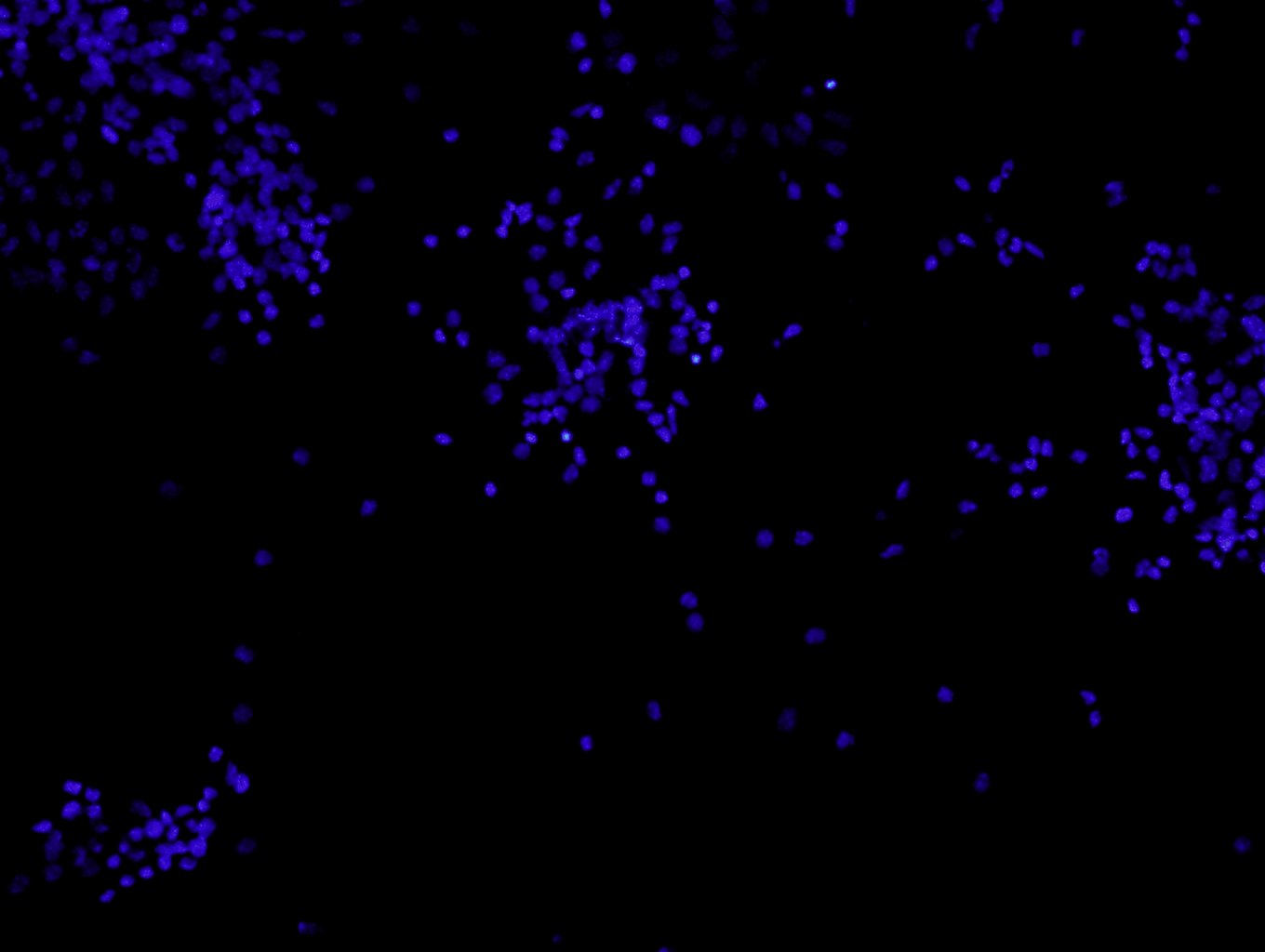

Supplement: Supplementary file 6 [file Data_Sheet_6.ZIP › con-1.tif]

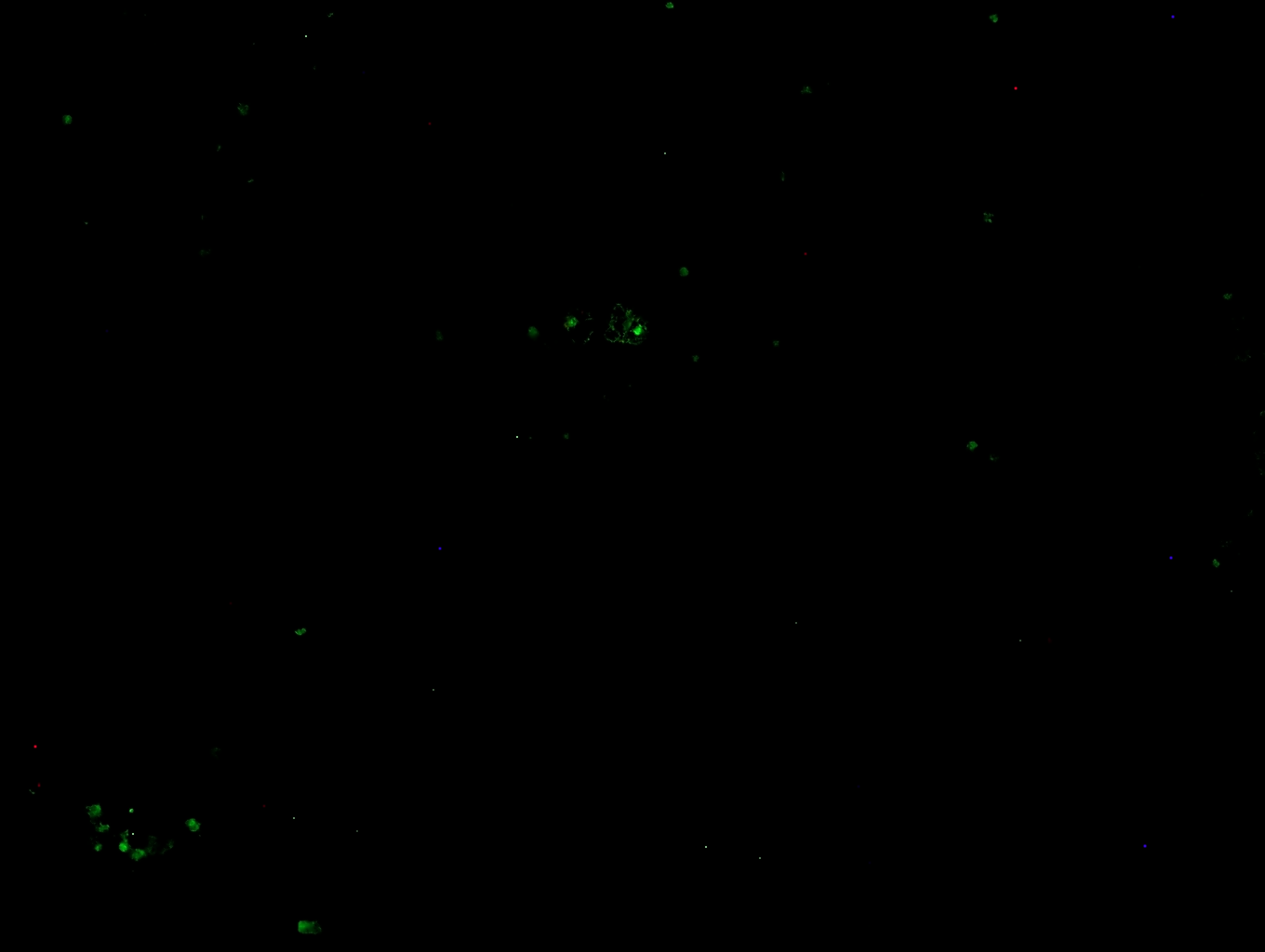

Supplement: Supplementary file 6 [file Data_Sheet_6.ZIP › con-2.tif]

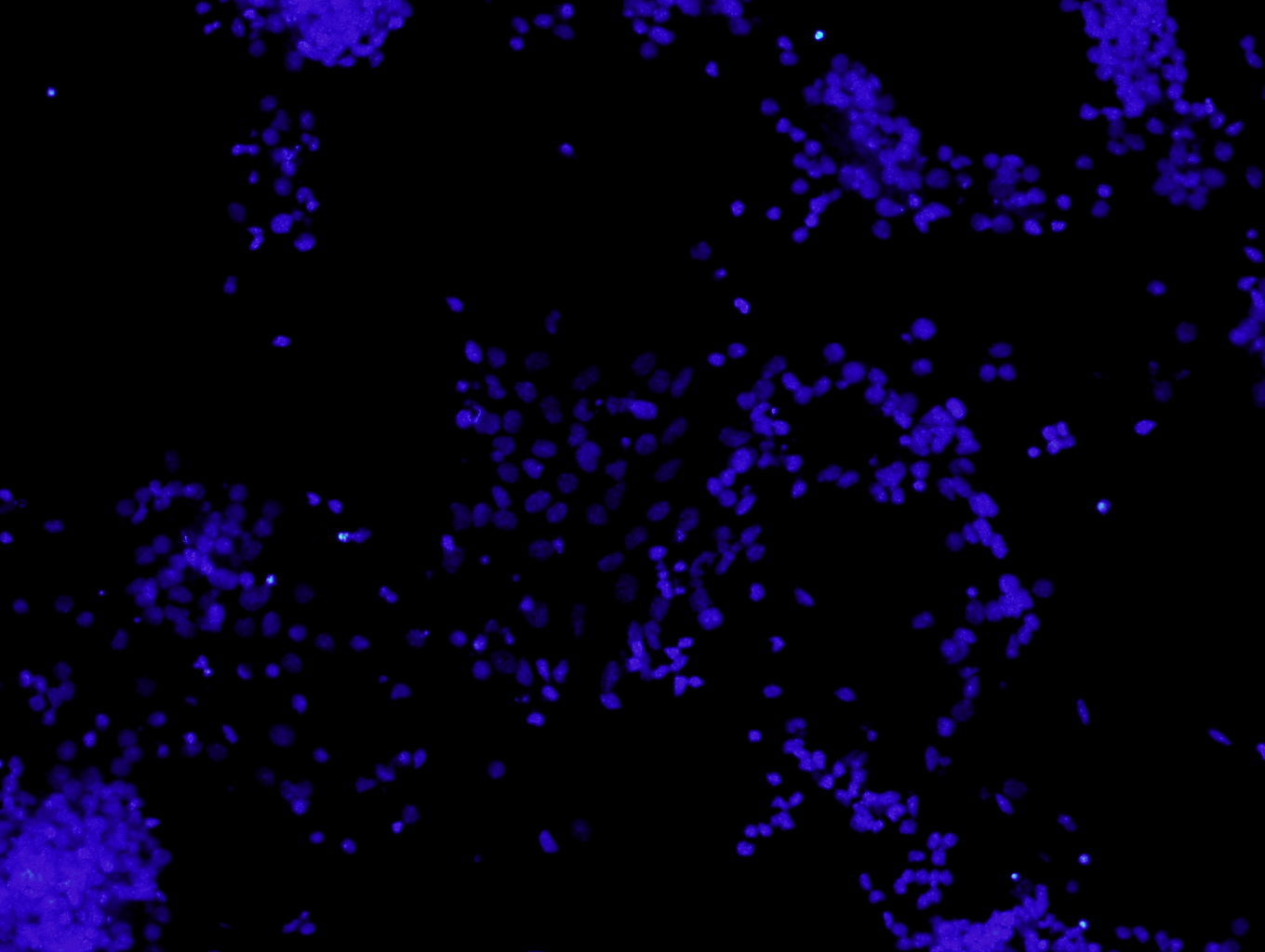

Supplement: Supplementary file 6 [file Data_Sheet_6.ZIP › hypoxia-1.tif]

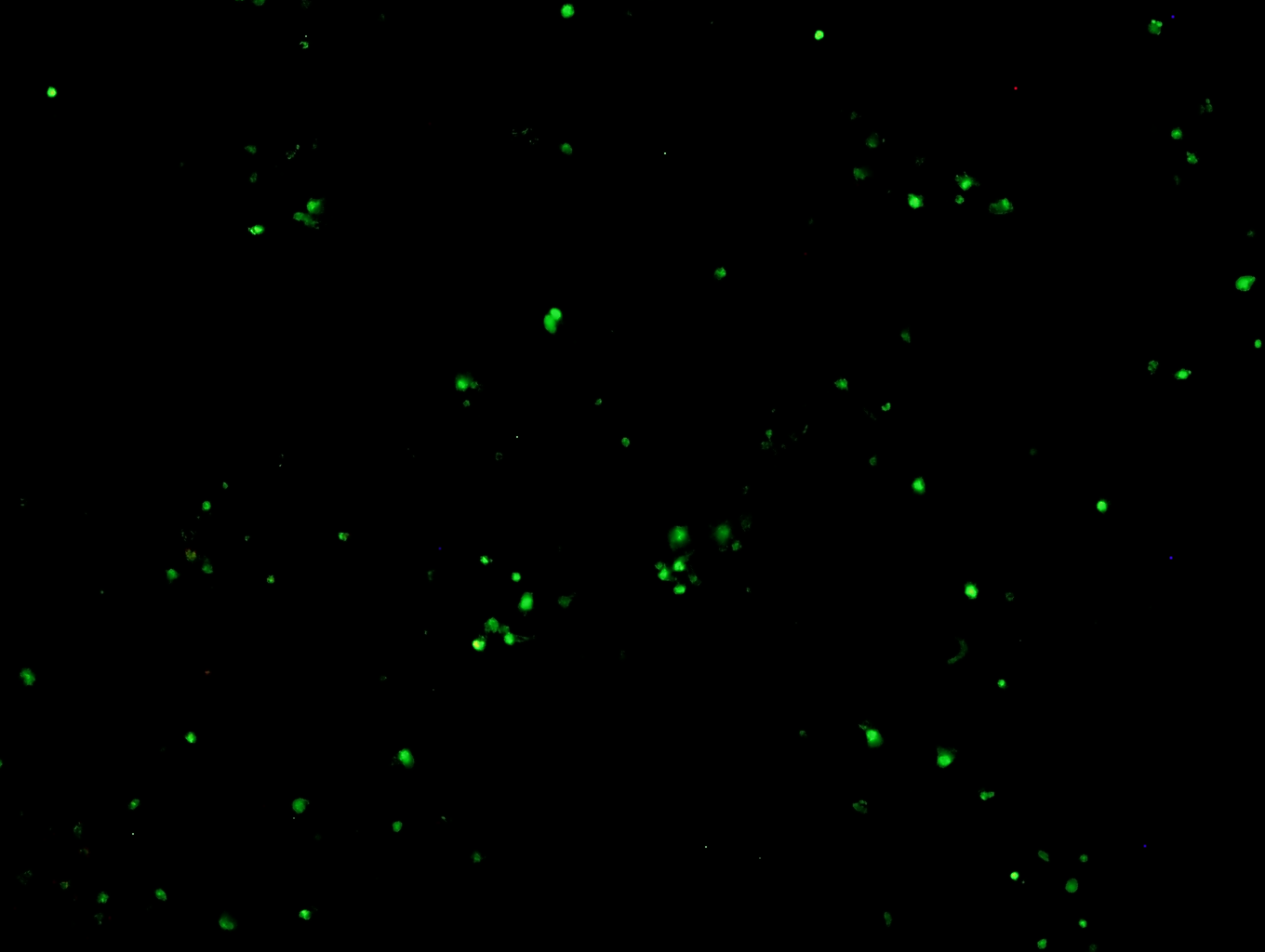

Supplement: Supplementary file 6 [file Data_Sheet_6.ZIP › hypoxia-2.tif]

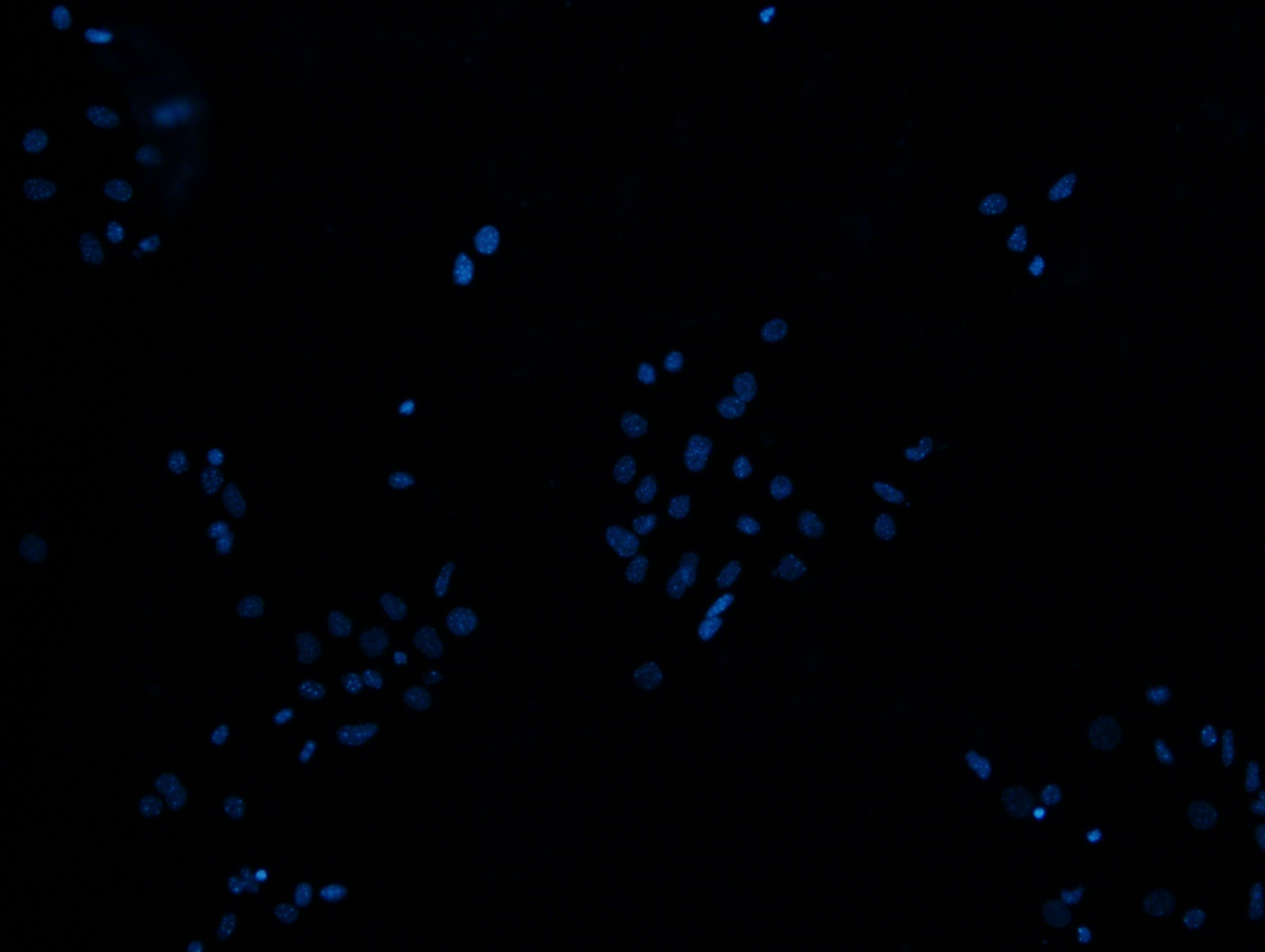

Supplement: Supplementary file 7 [file Data_Sheet_7.ZIP › control/1-1.tif]

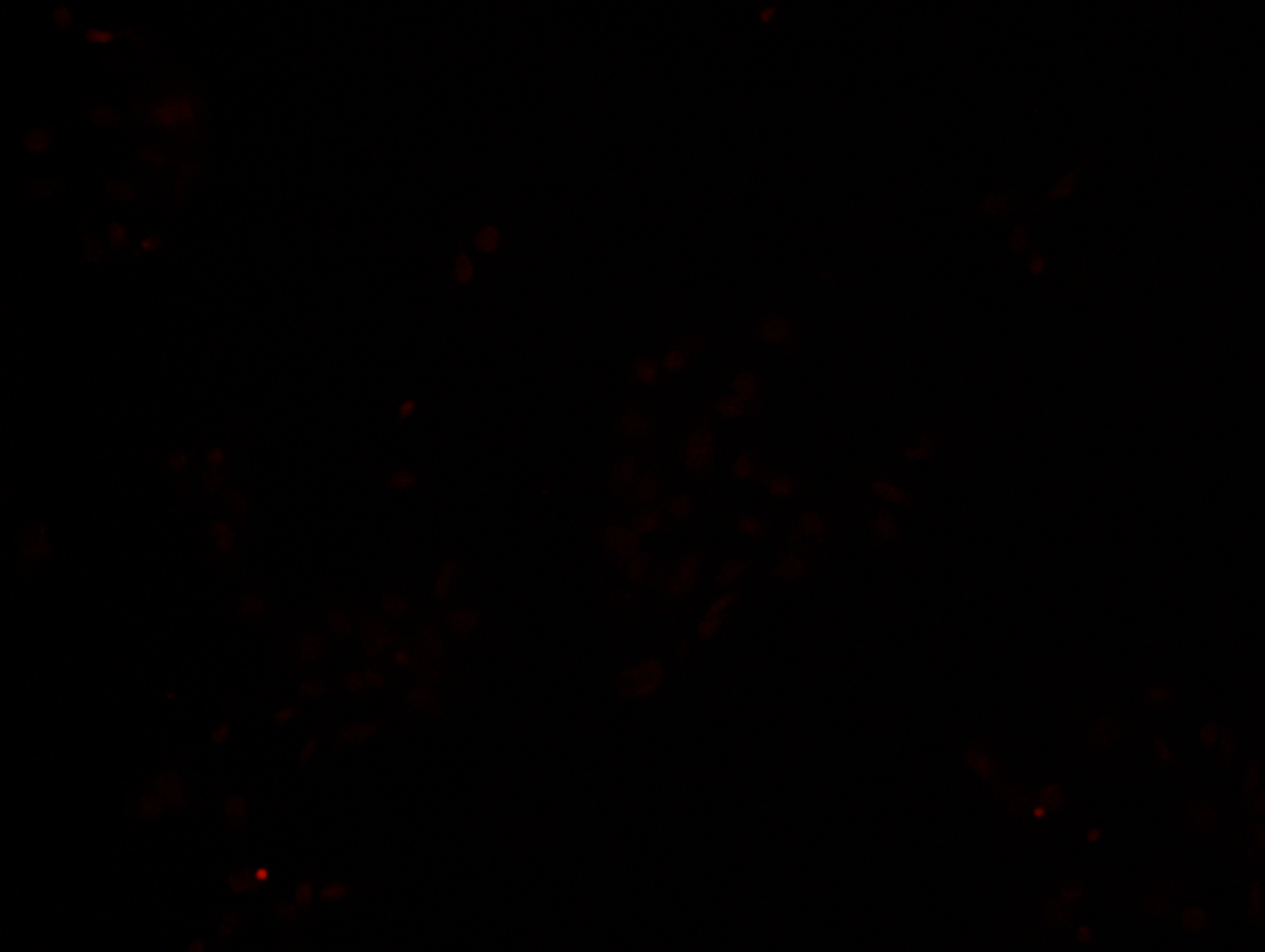

Supplement: Supplementary file 7 [file Data_Sheet_7.ZIP › control/1-2.tif]

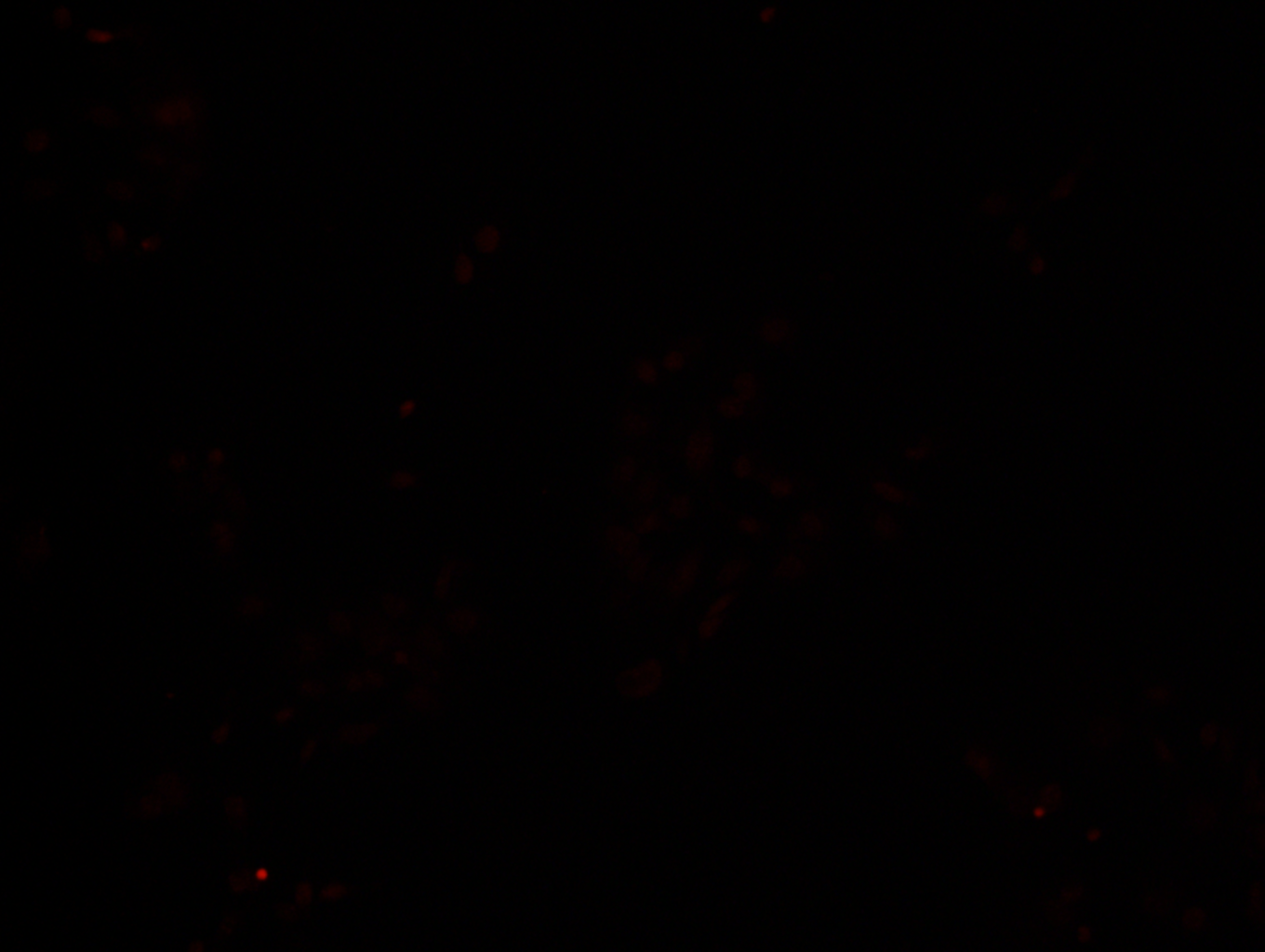

Supplement: Supplementary file 7 [file Data_Sheet_7.ZIP › control/1-3.tif]

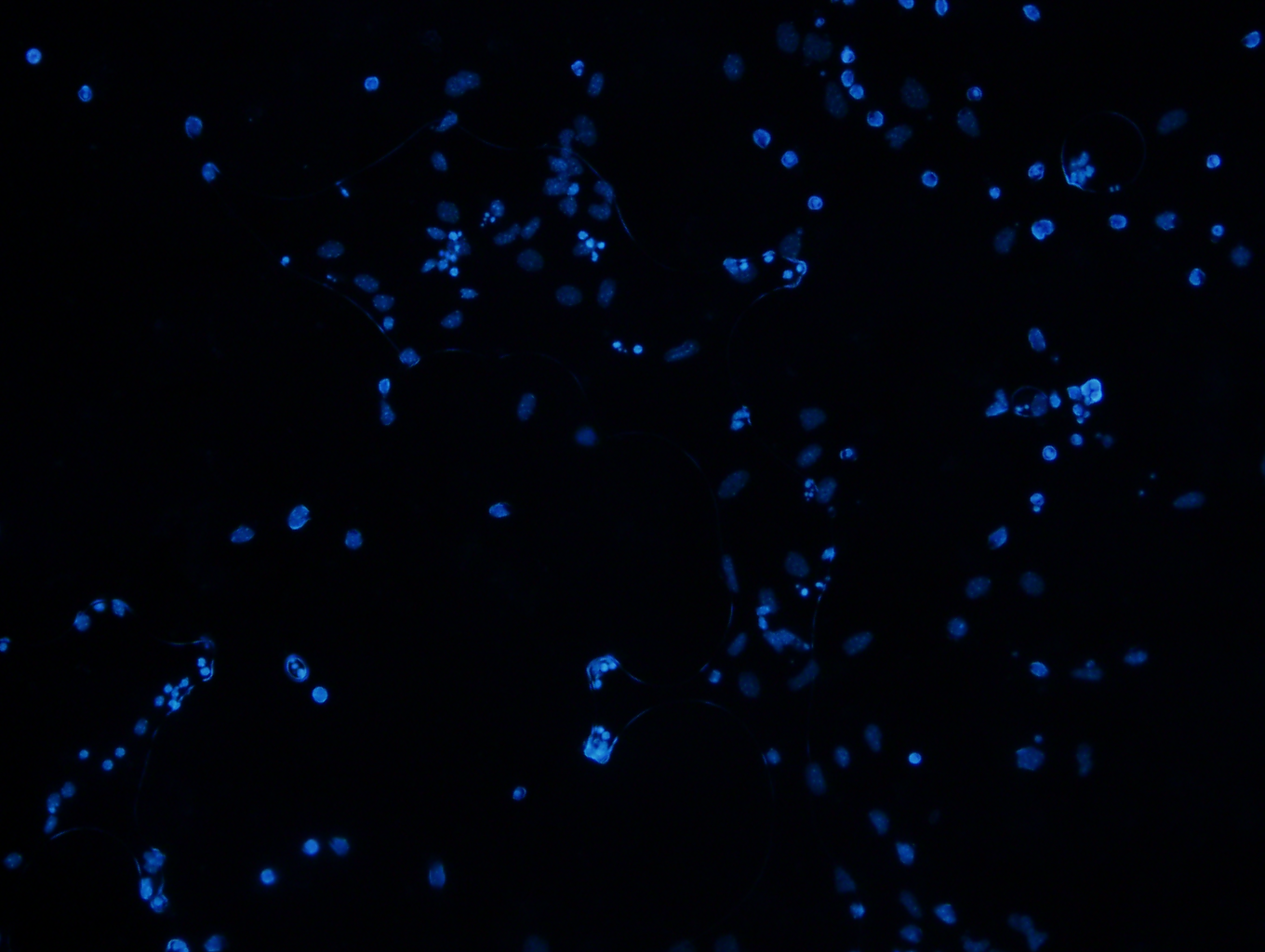

Supplement: Supplementary file 7 [file Data_Sheet_7.ZIP › hypoxia/1-1.tif]

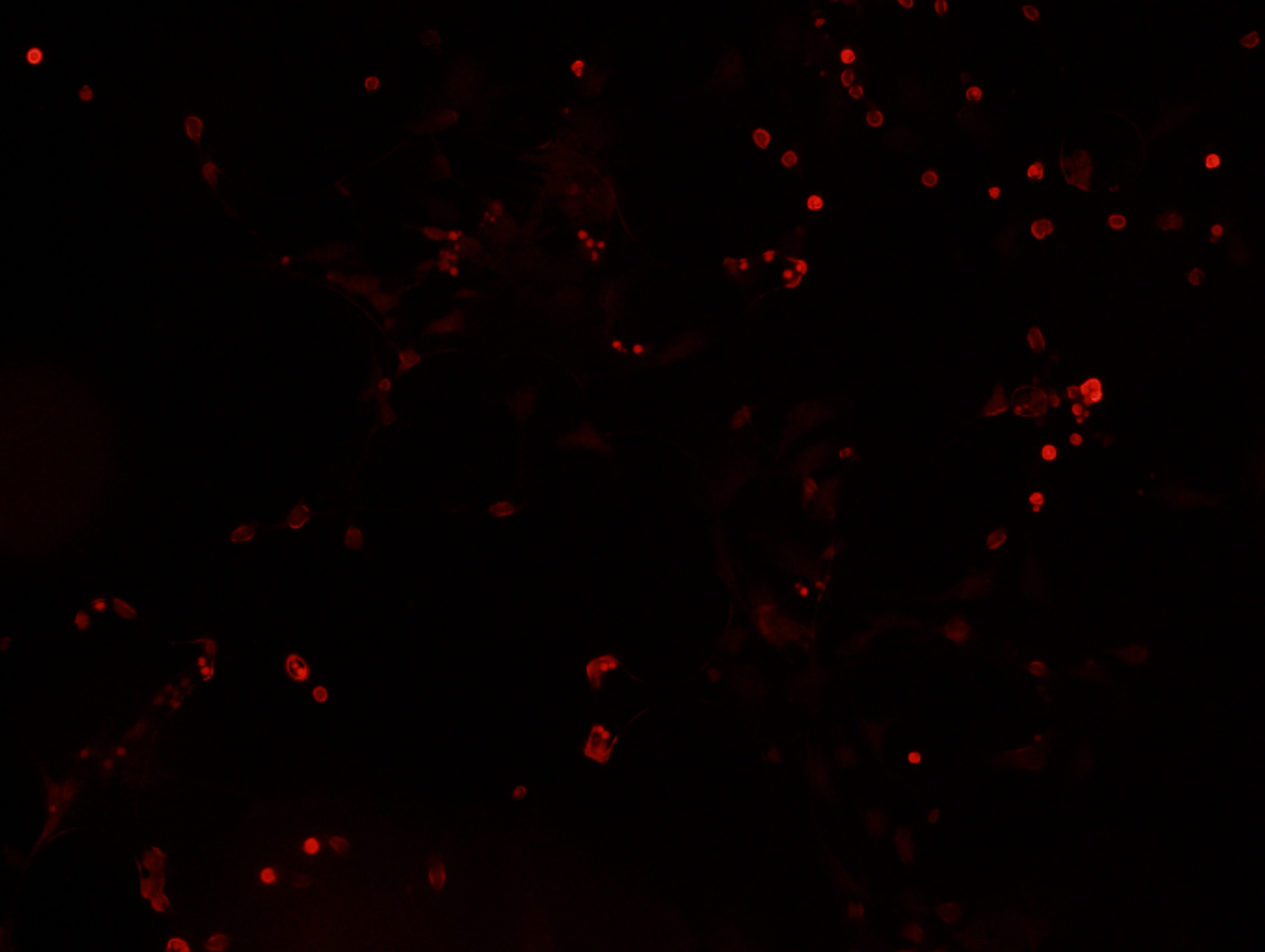

Supplement: Supplementary file 7 [file Data_Sheet_7.ZIP › hypoxia/1-2.tif]

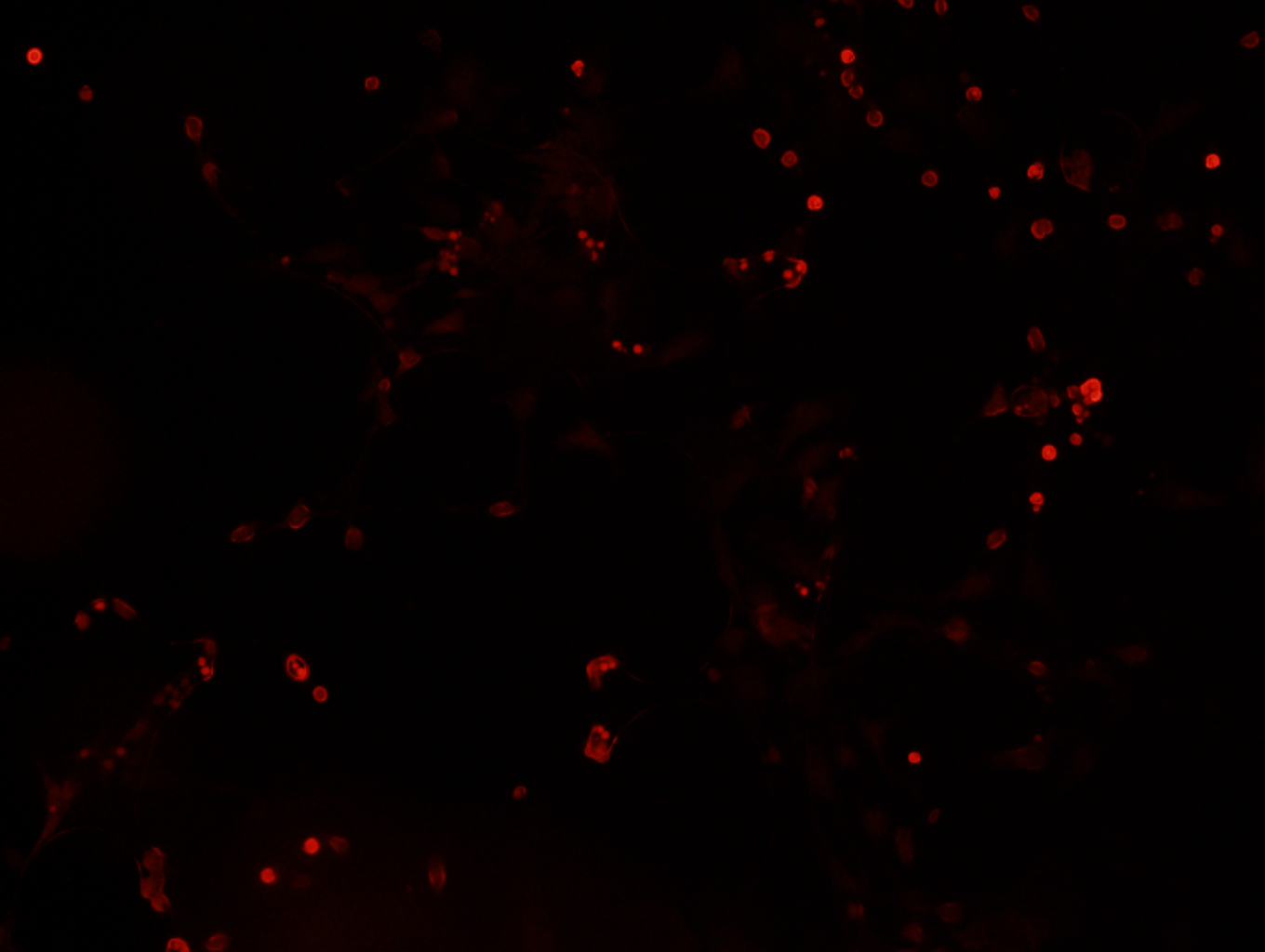

Supplement: Supplementary file 7 [file Data_Sheet_7.ZIP › hypoxia/1-3.tif]

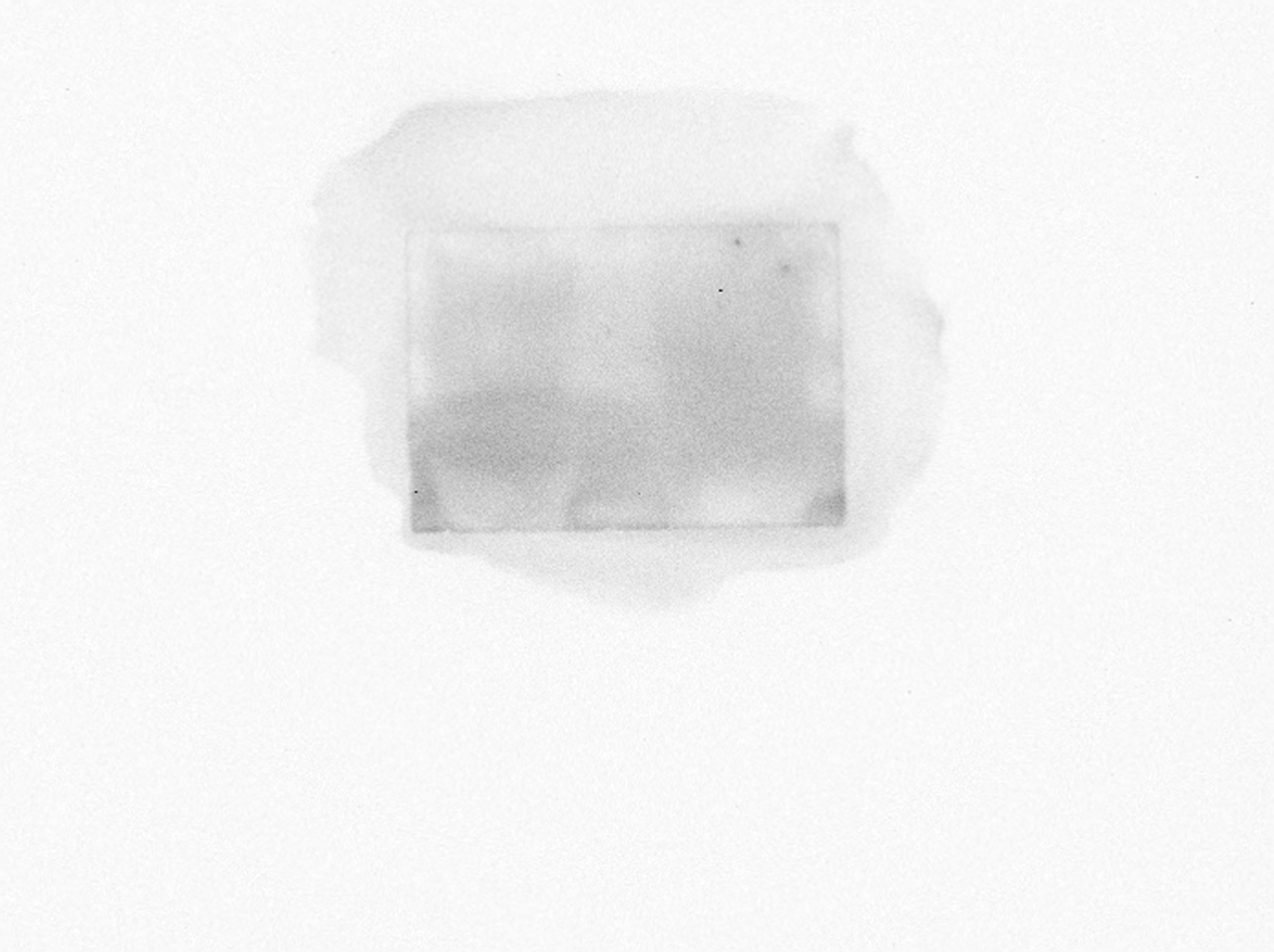

Supplement: Supplementary file 8 [file Data_Sheet_8.ZIP › cyto/1-4 cox4-1.tif]

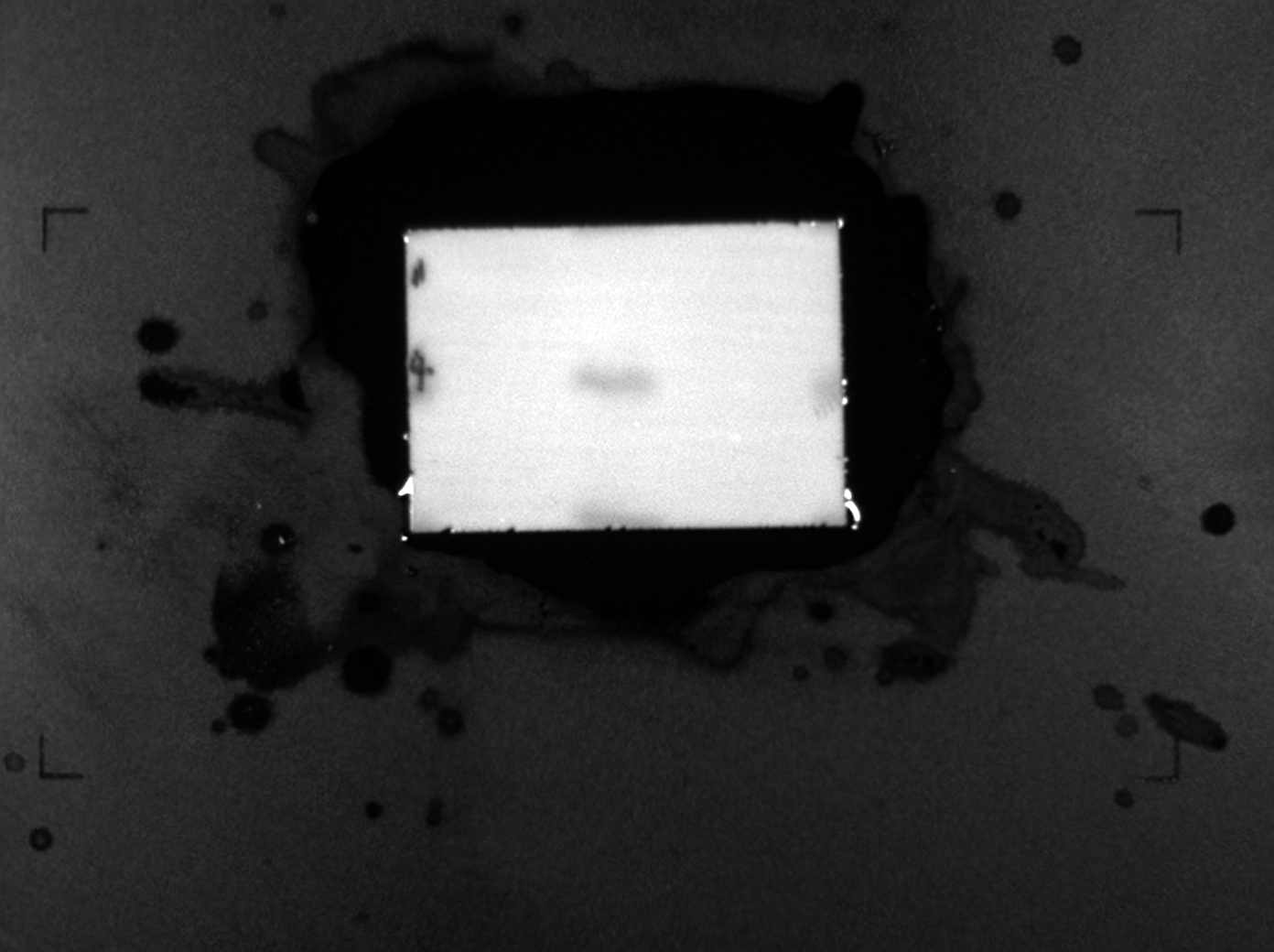

Supplement: Supplementary file 8 [file Data_Sheet_8.ZIP › cyto/1-4 cox4-2.tif]

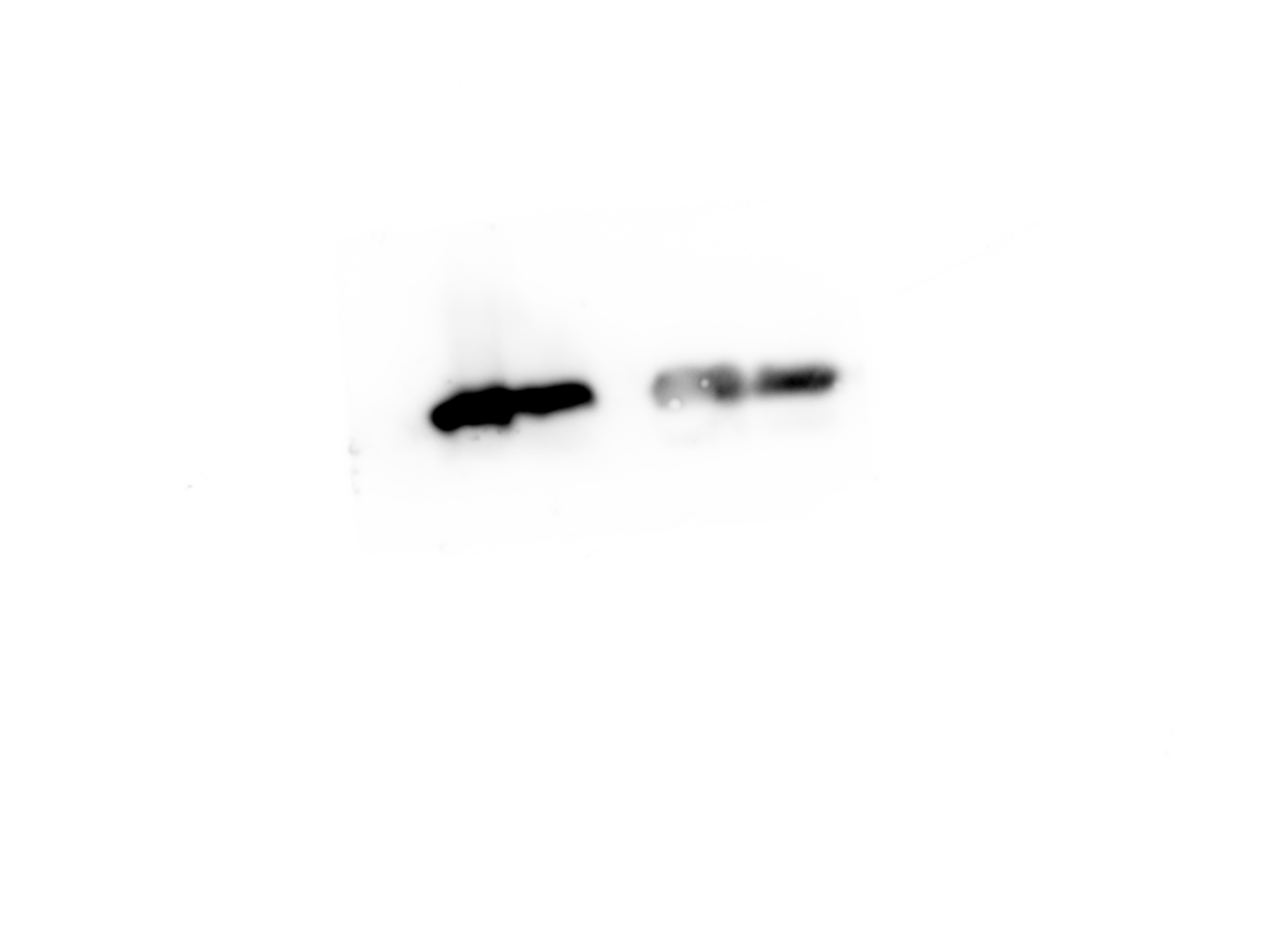

Supplement: Supplementary file 8 [file Data_Sheet_8.ZIP › cyto/2-1 cytoc-1.tif]

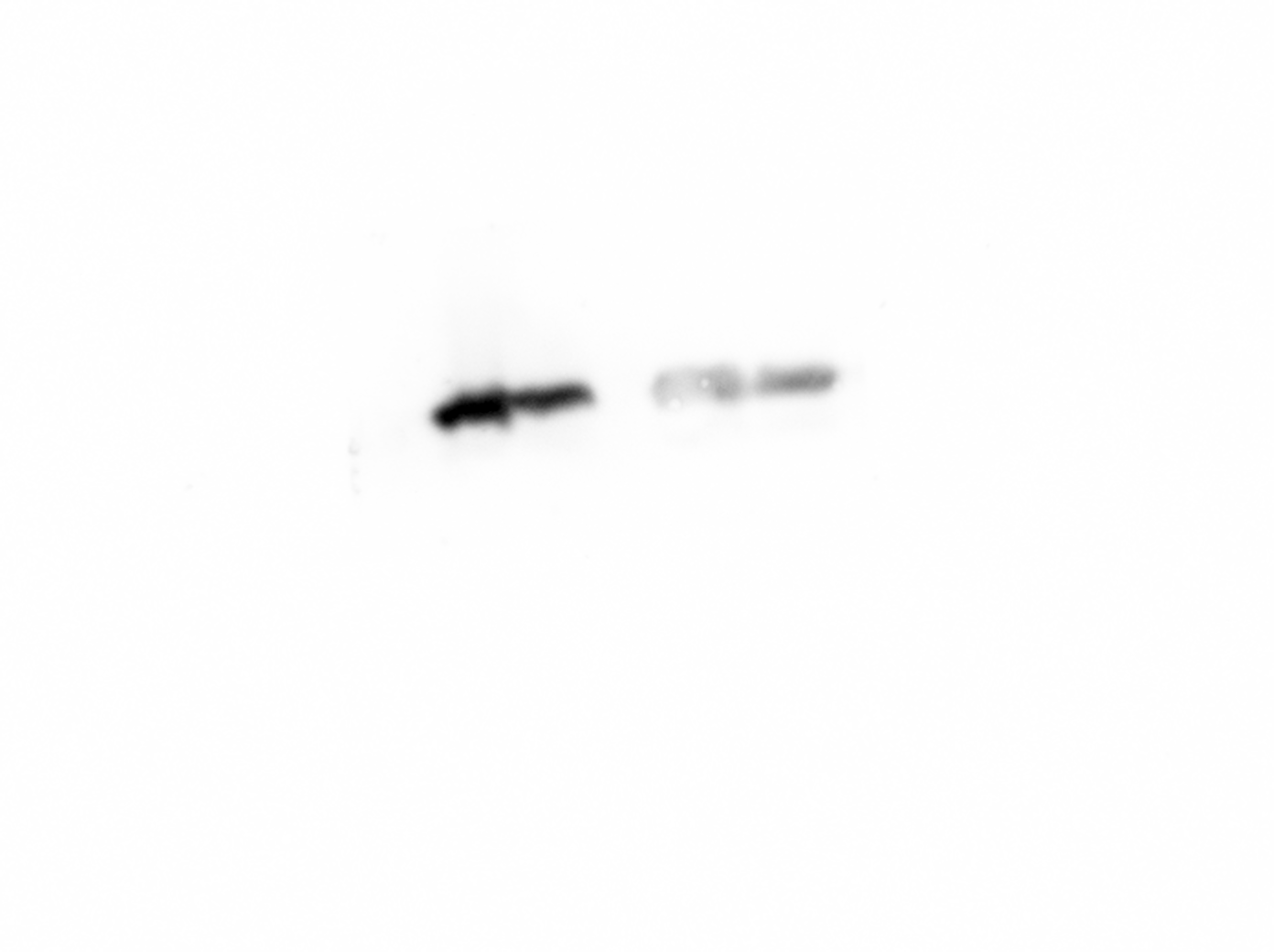

Supplement: Supplementary file 8 [file Data_Sheet_8.ZIP › cyto/2-1 cytoc-2左.tif]

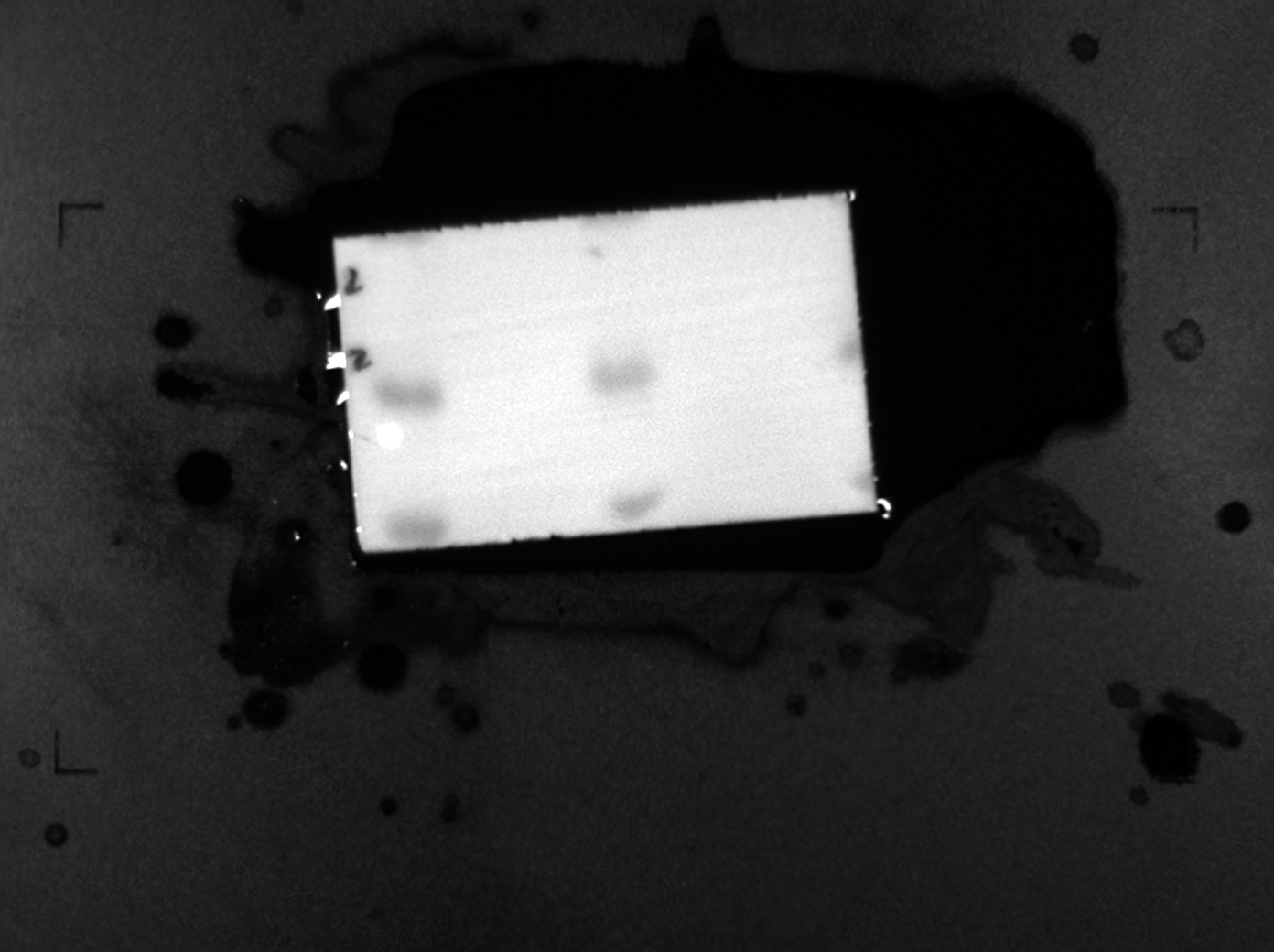

Supplement: Supplementary file 8 [file Data_Sheet_8.ZIP › cyto/2-1 cytoc-3.tif]

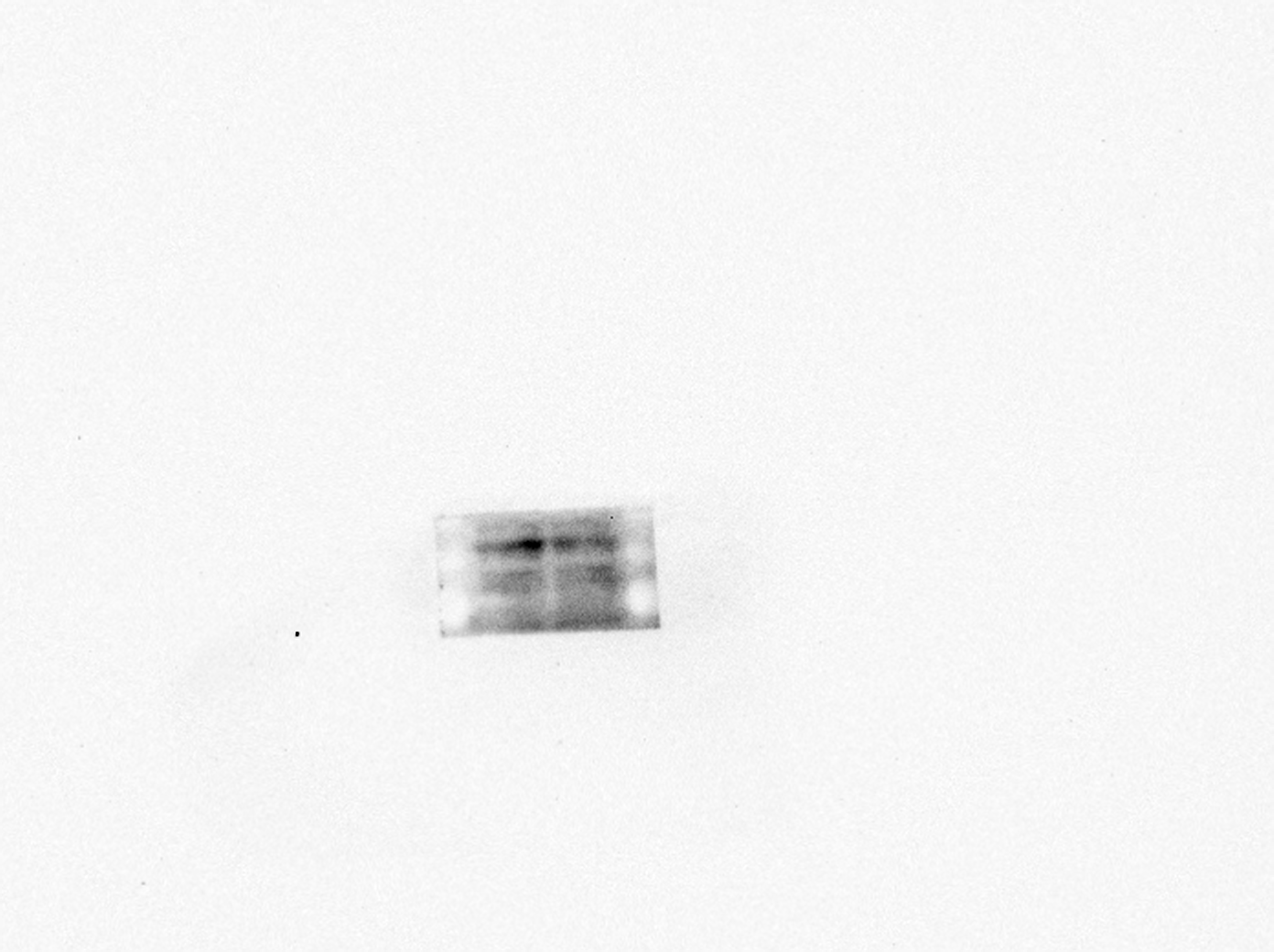

Supplement: Supplementary file 8 [file Data_Sheet_8.ZIP › cyto/cyto 616-1.tif]

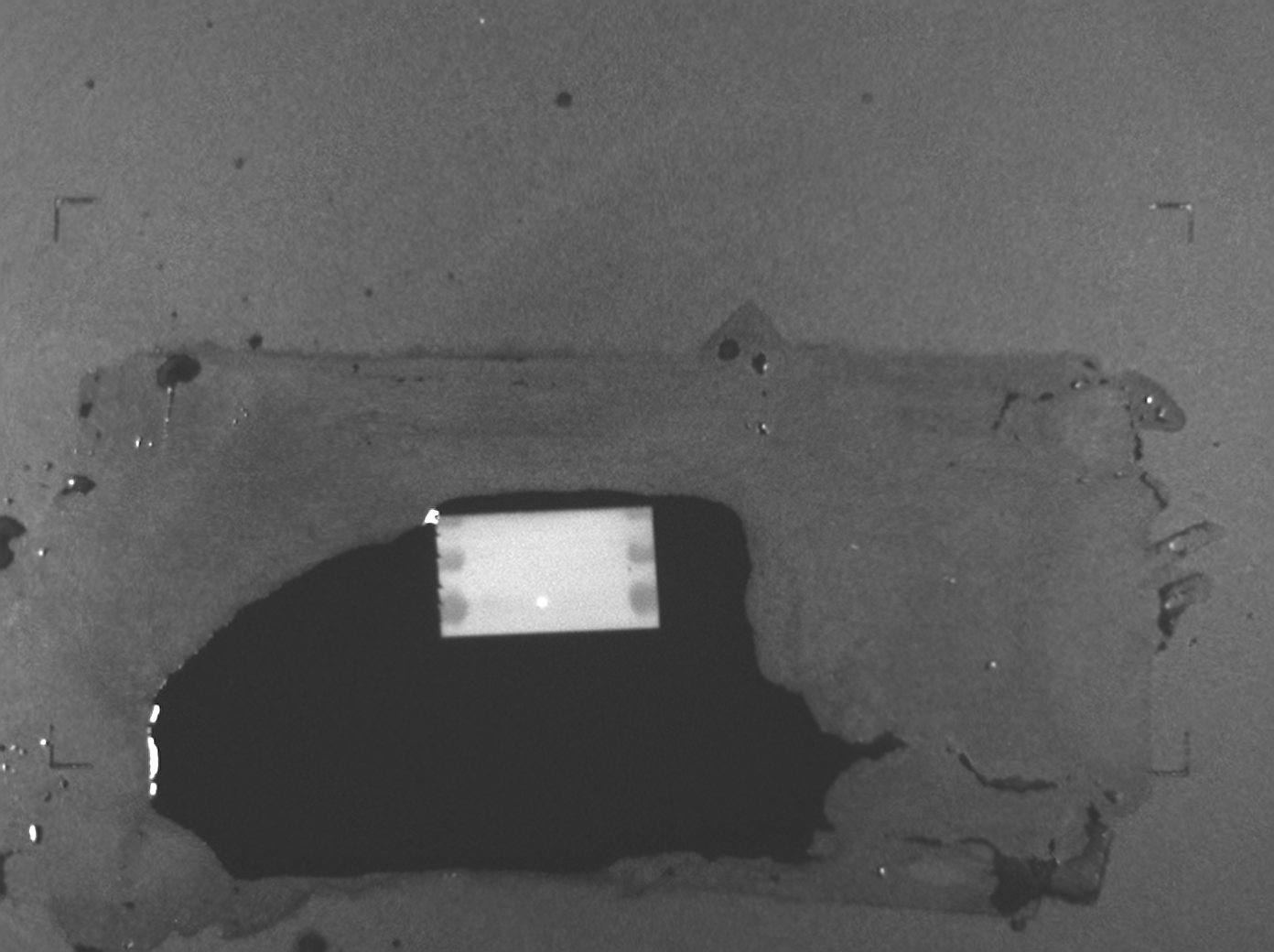

Supplement: Supplementary file 8 [file Data_Sheet_8.ZIP › cyto/cyto 616-2.tif]

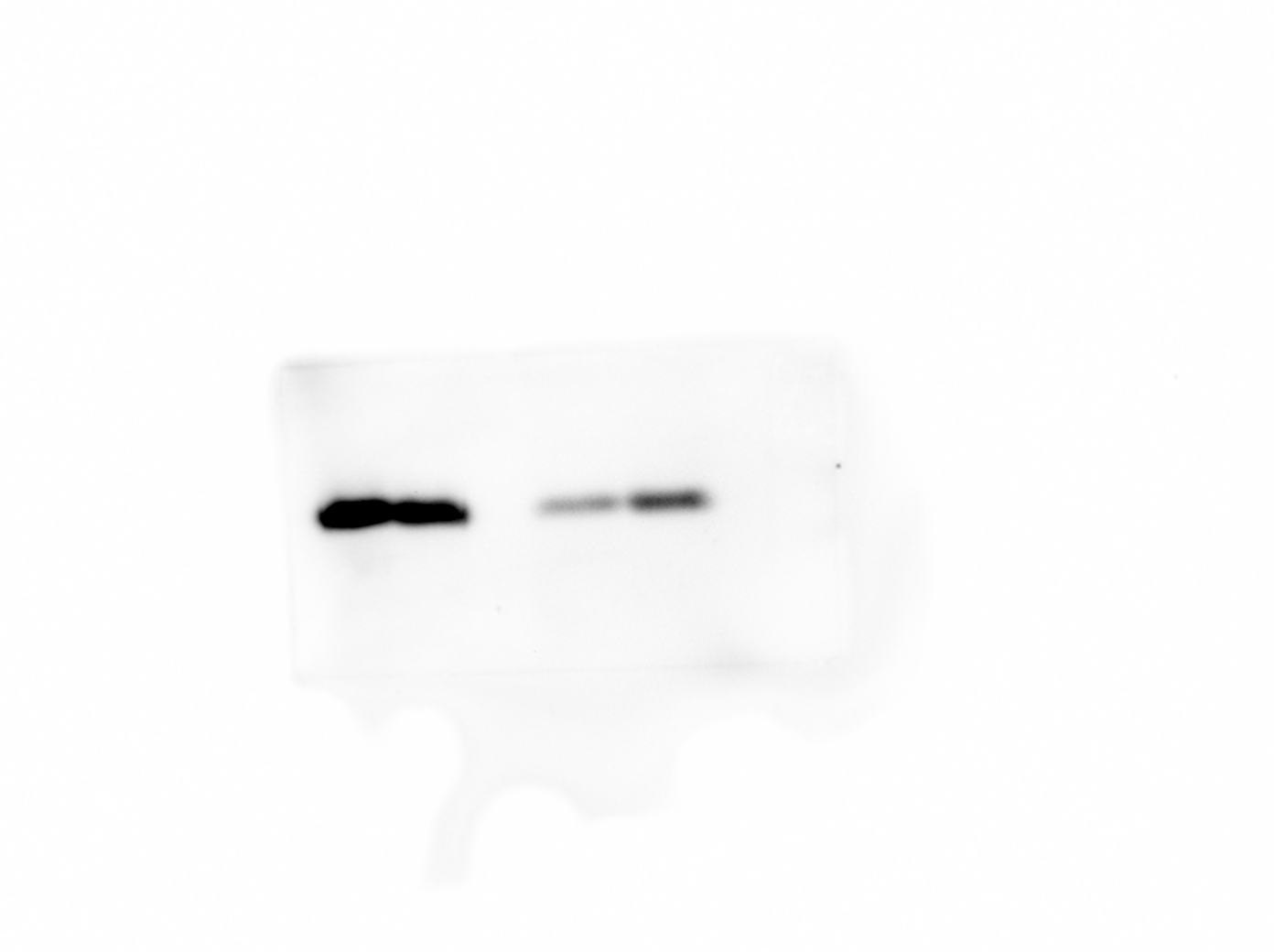

Supplement: Supplementary file 8 [file Data_Sheet_8.ZIP › cyto/cyto bax-1.tif]

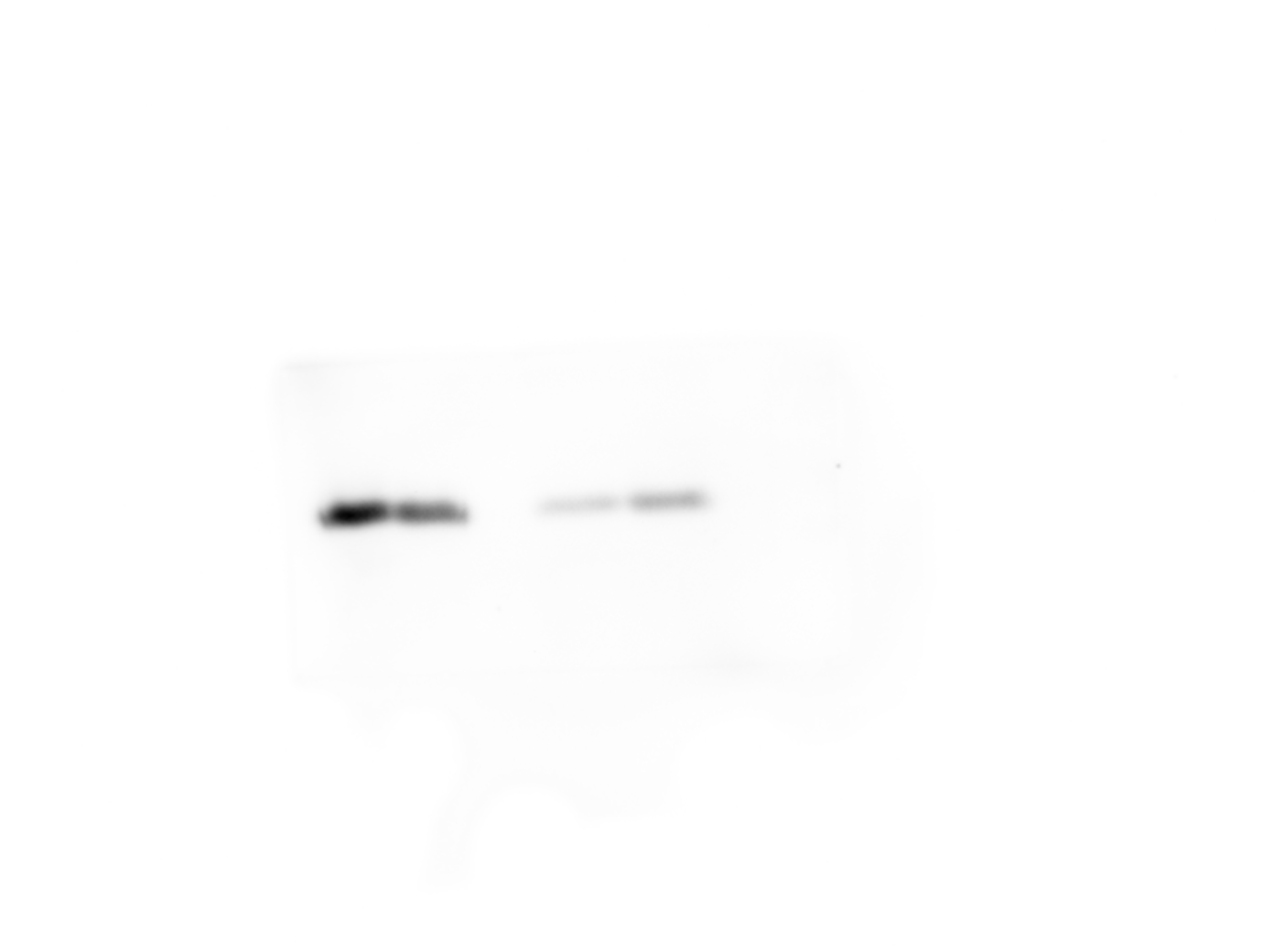

Supplement: Supplementary file 8 [file Data_Sheet_8.ZIP › cyto/cyto bax-2.tif]

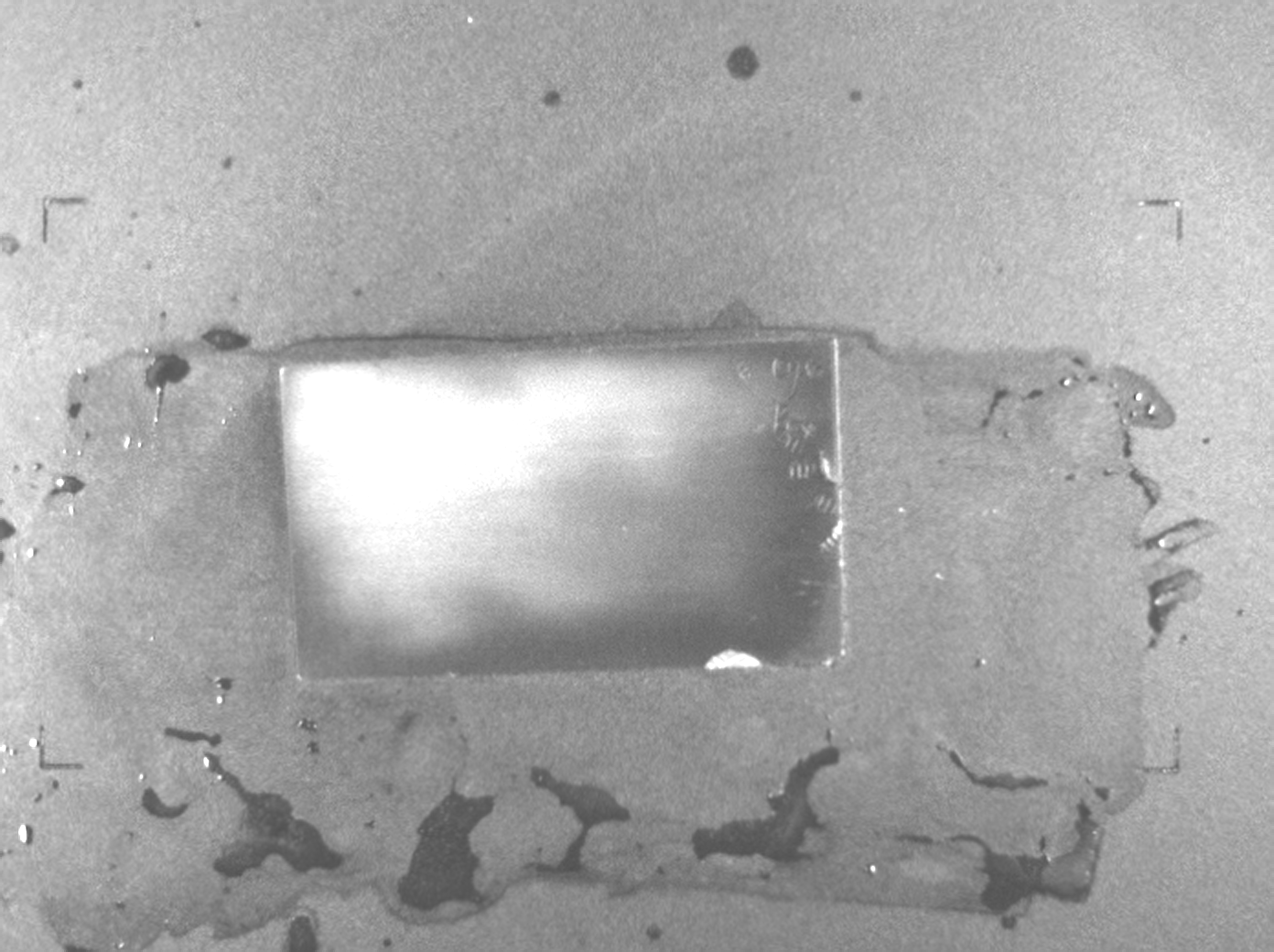

Supplement: Supplementary file 8 [file Data_Sheet_8.ZIP › cyto/cyto bax-3.tif]

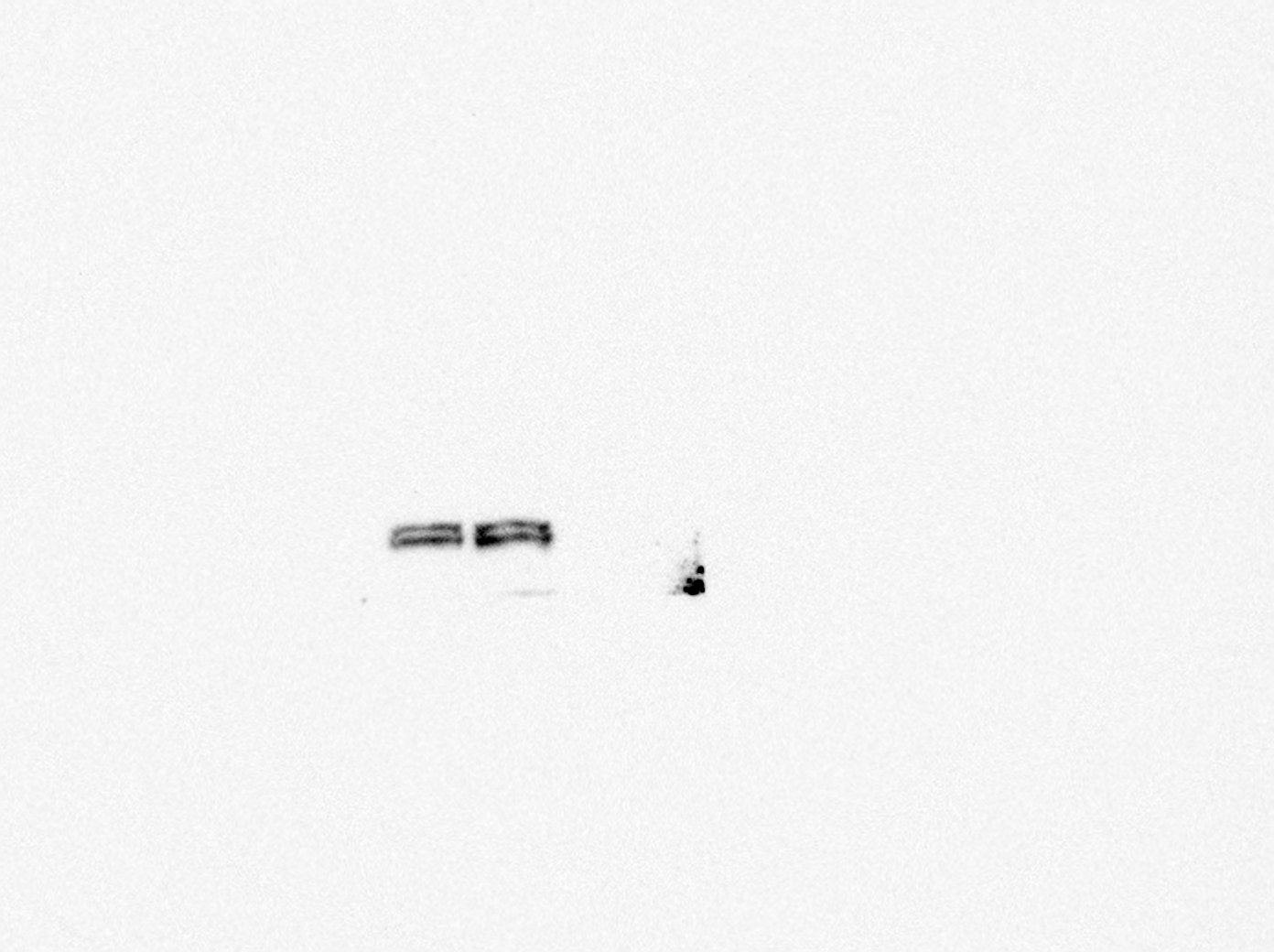

Supplement: Supplementary file 8 [file Data_Sheet_8.ZIP › cyto/cyto drp1-3.tif]

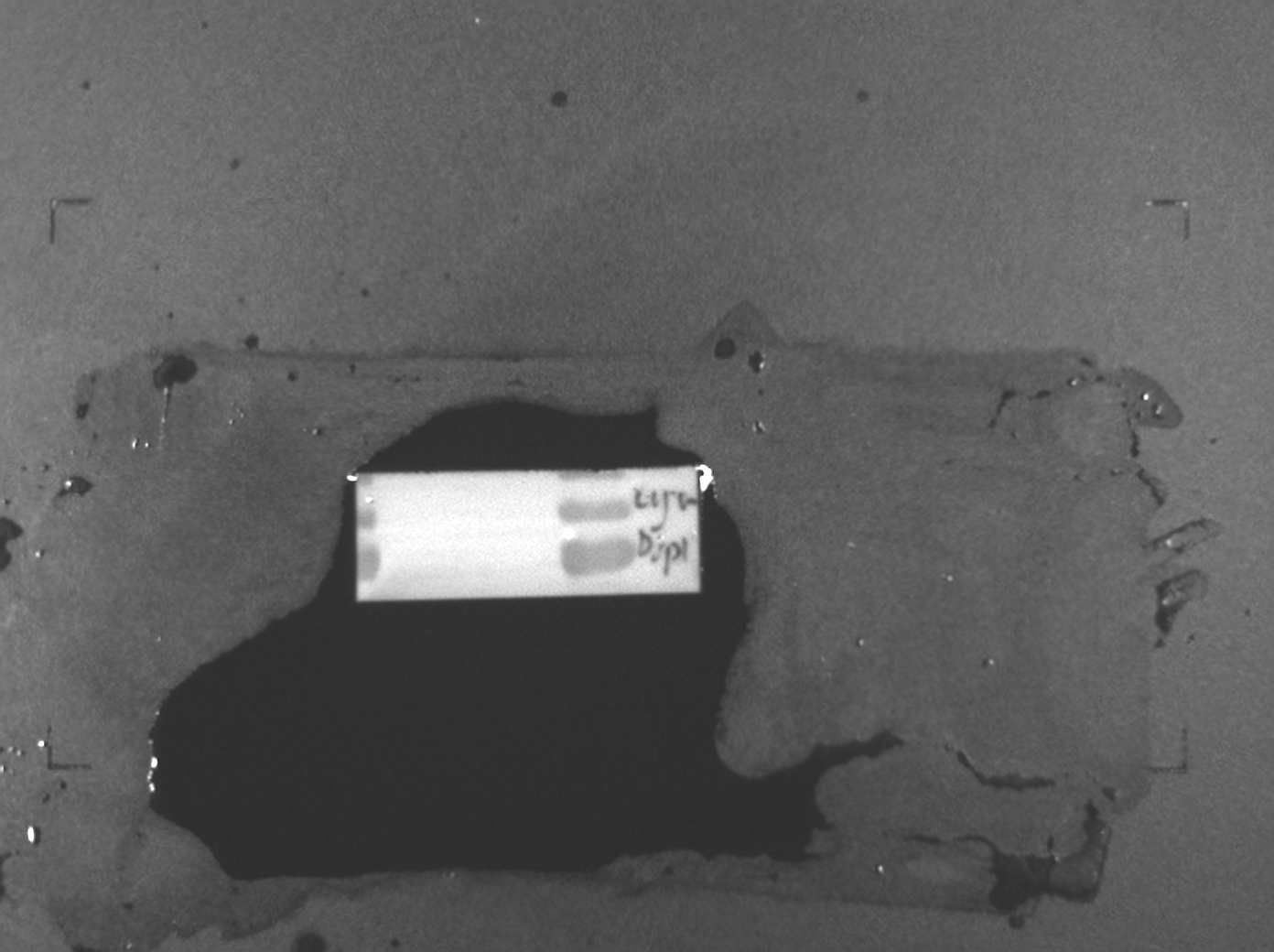

Supplement: Supplementary file 8 [file Data_Sheet_8.ZIP › cyto/cyto drp1-4.tif]

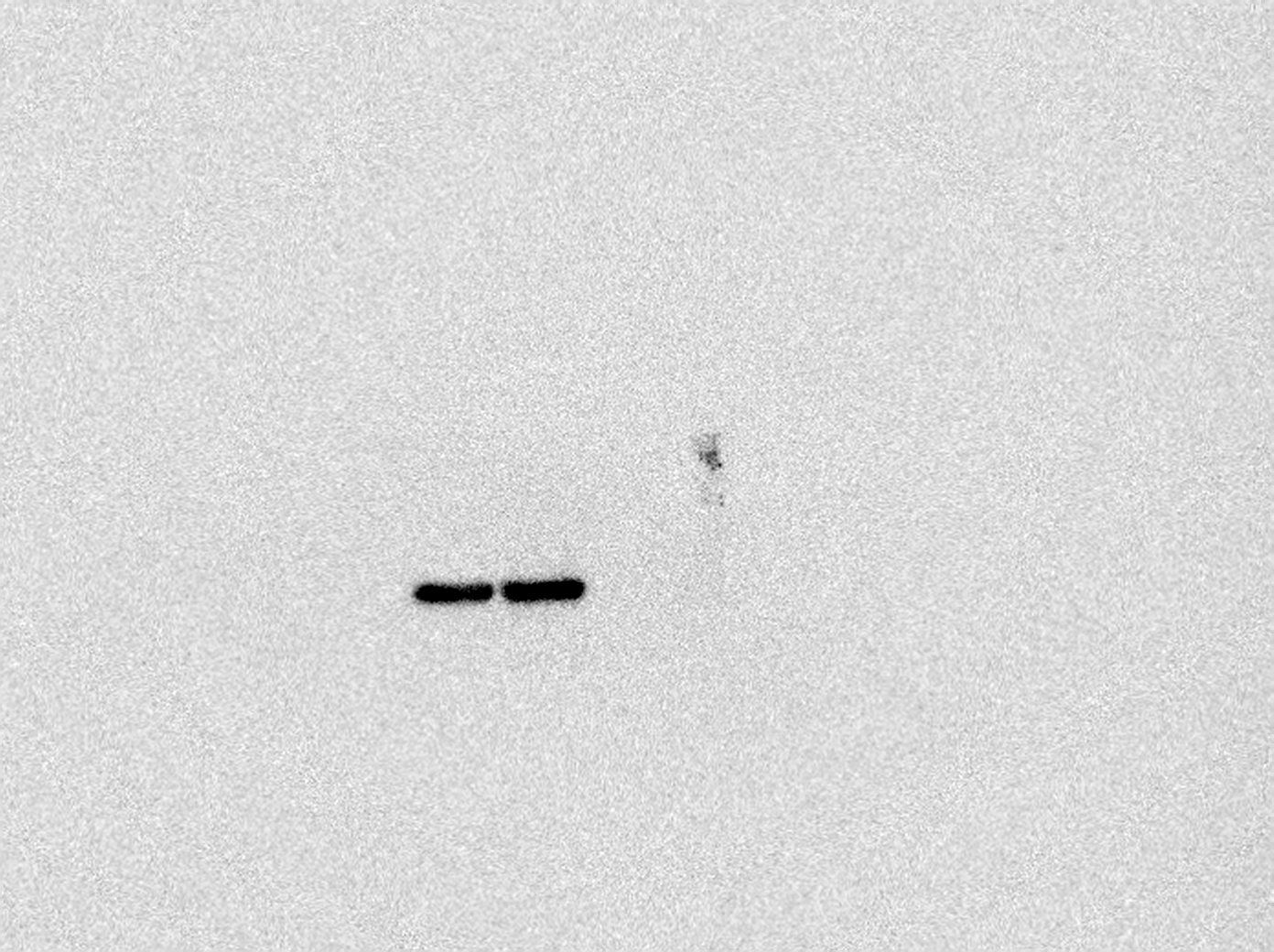

Supplement: Supplementary file 8 [file Data_Sheet_8.ZIP › cyto/cyto-actin-3.tif]

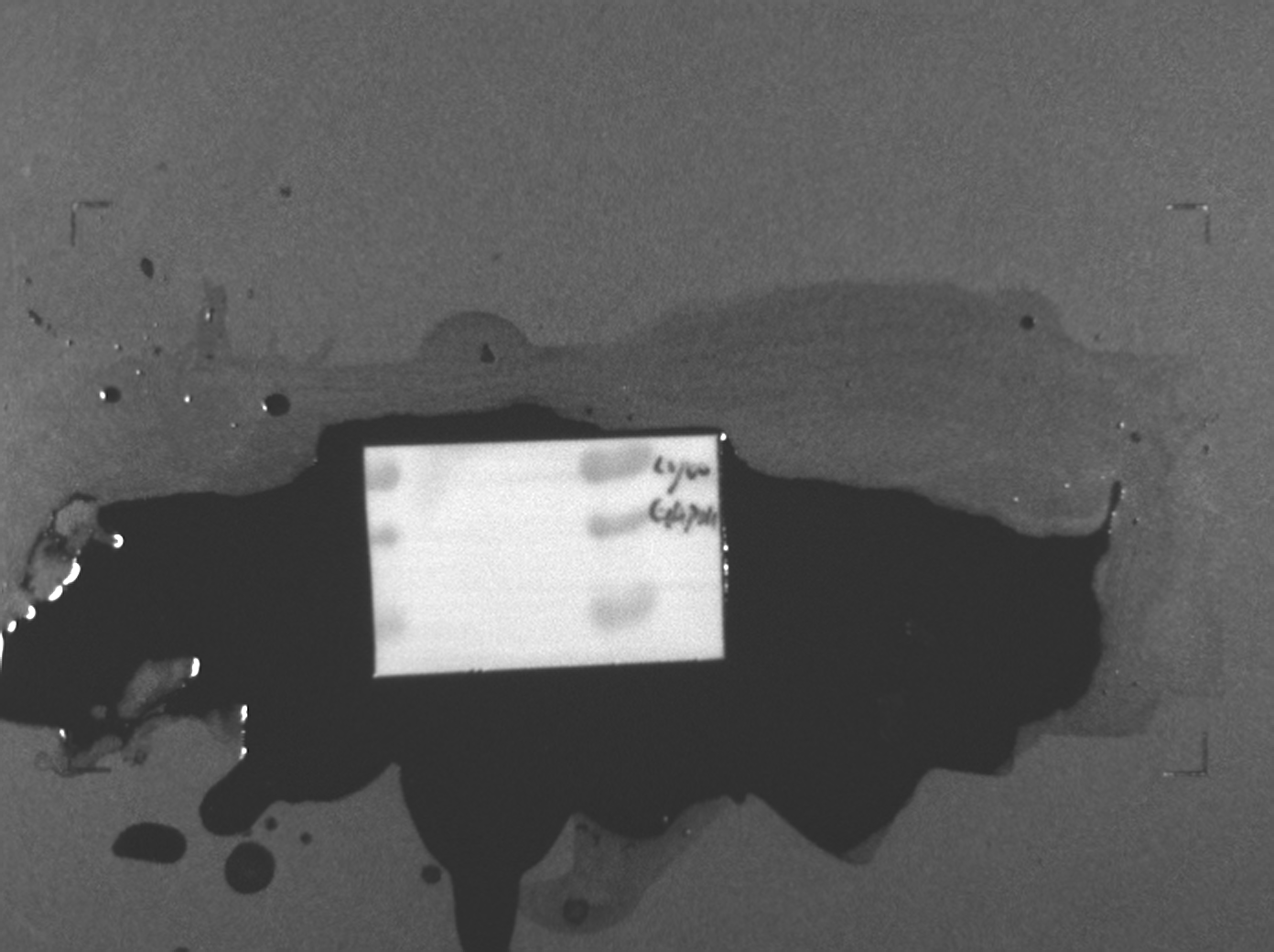

Supplement: Supplementary file 8 [file Data_Sheet_8.ZIP › cyto/cyto-actin-4.tif]

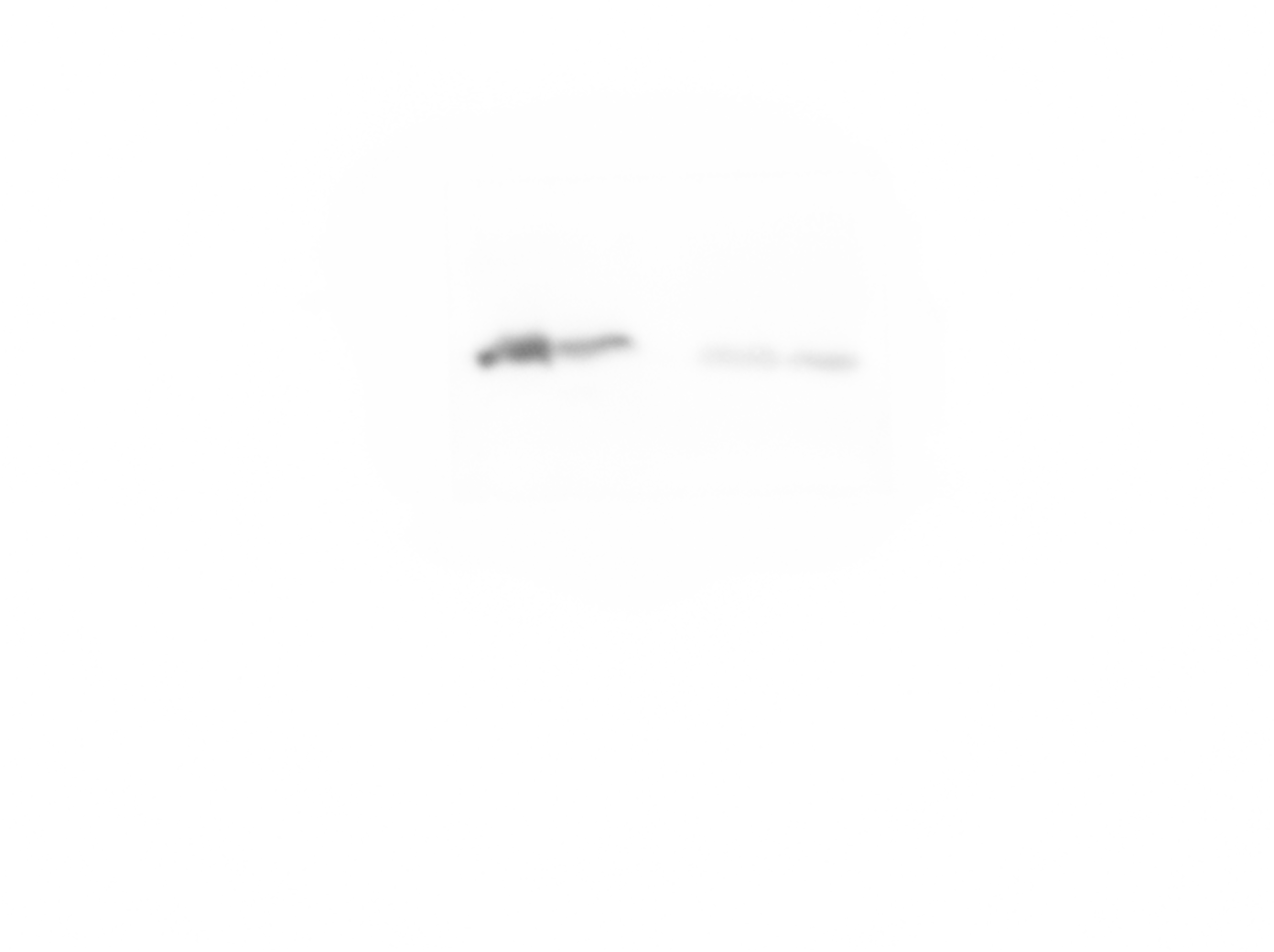

Supplement: Supplementary file 8 [file Data_Sheet_8.ZIP › mito/2-4 cox4-1.tif]

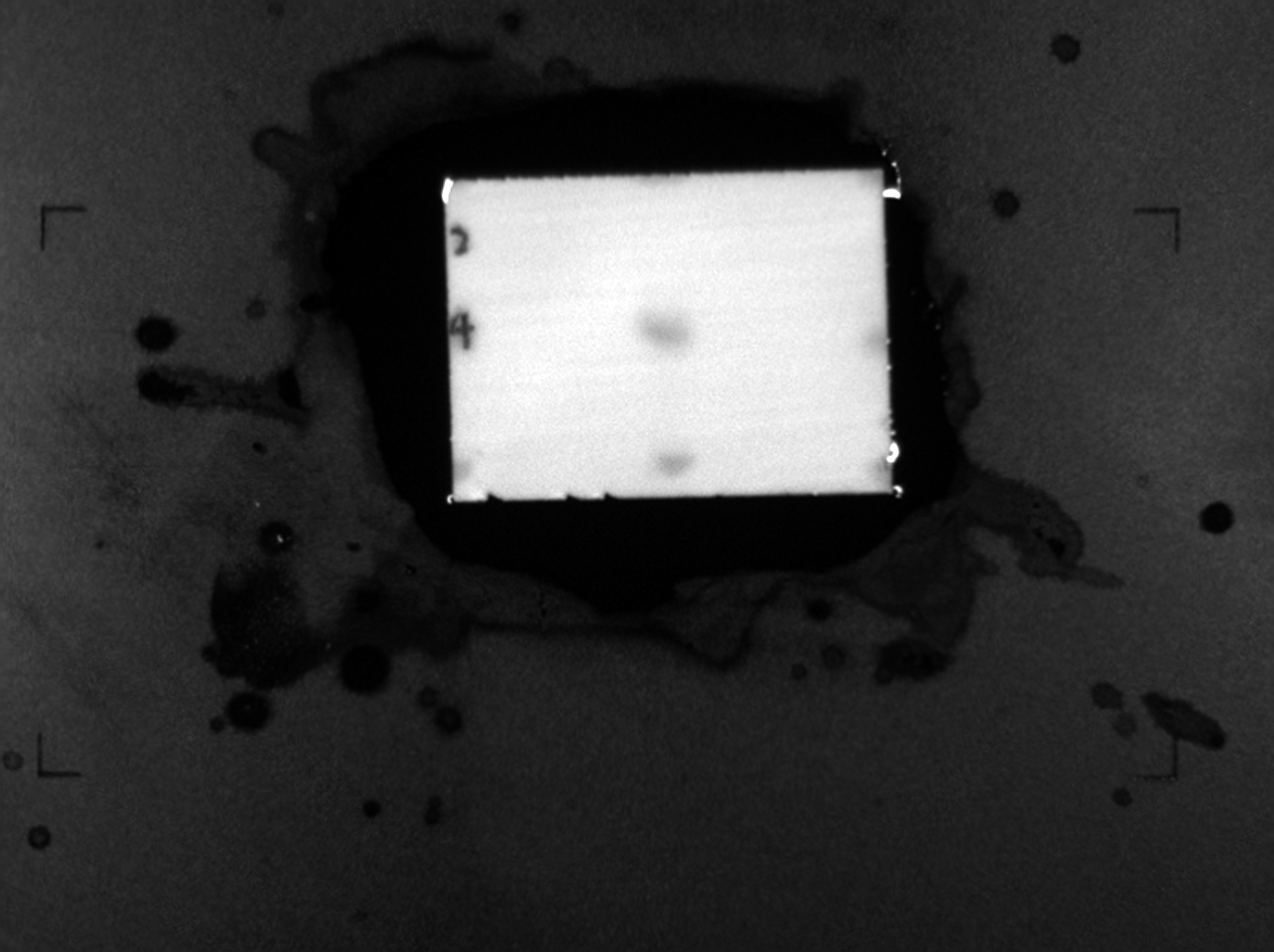

Supplement: Supplementary file 8 [file Data_Sheet_8.ZIP › mito/2-4 cox4-2.tif]

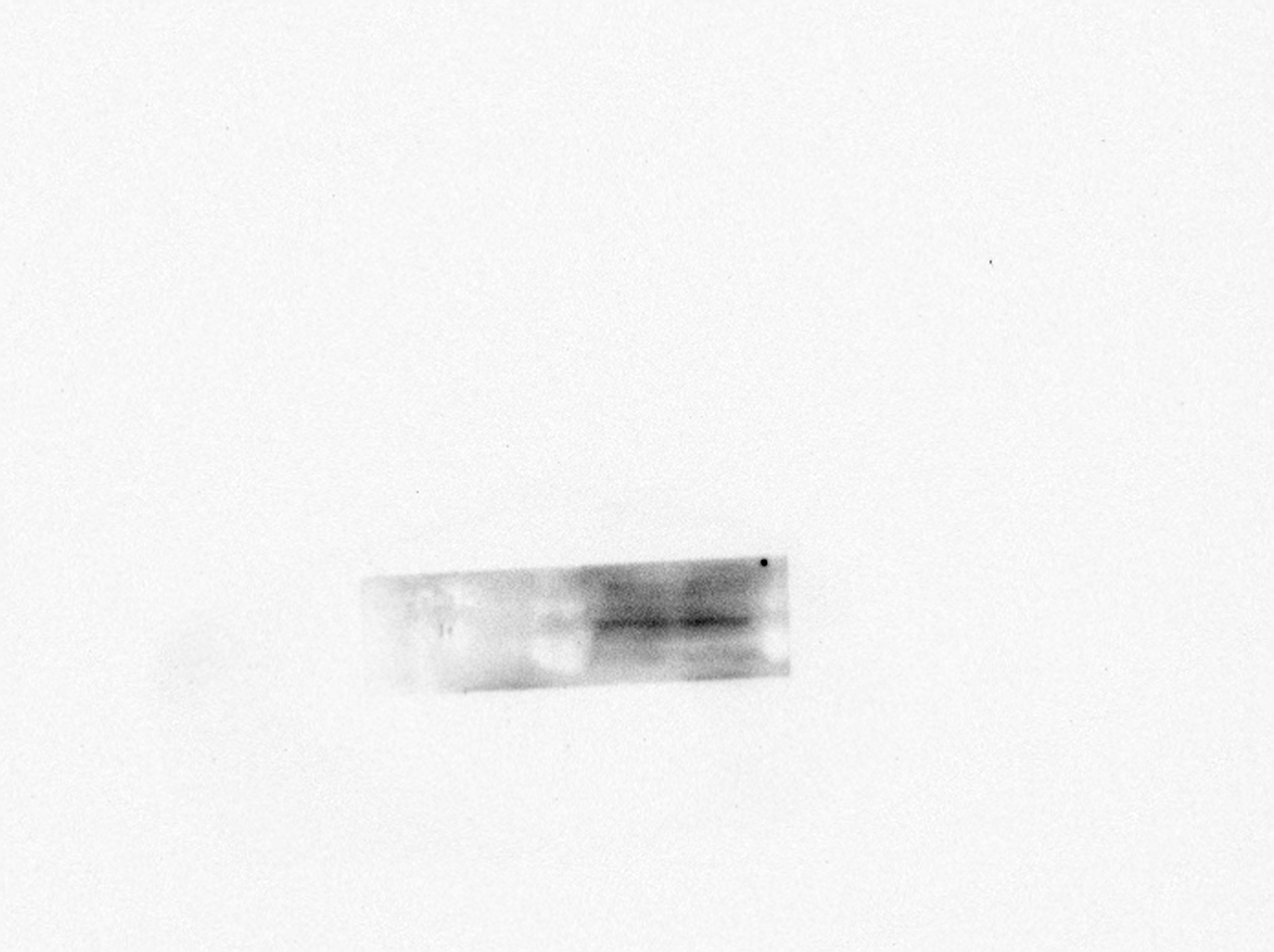

Supplement: Supplementary file 8 [file Data_Sheet_8.ZIP › mito/mito 616-3.tif]

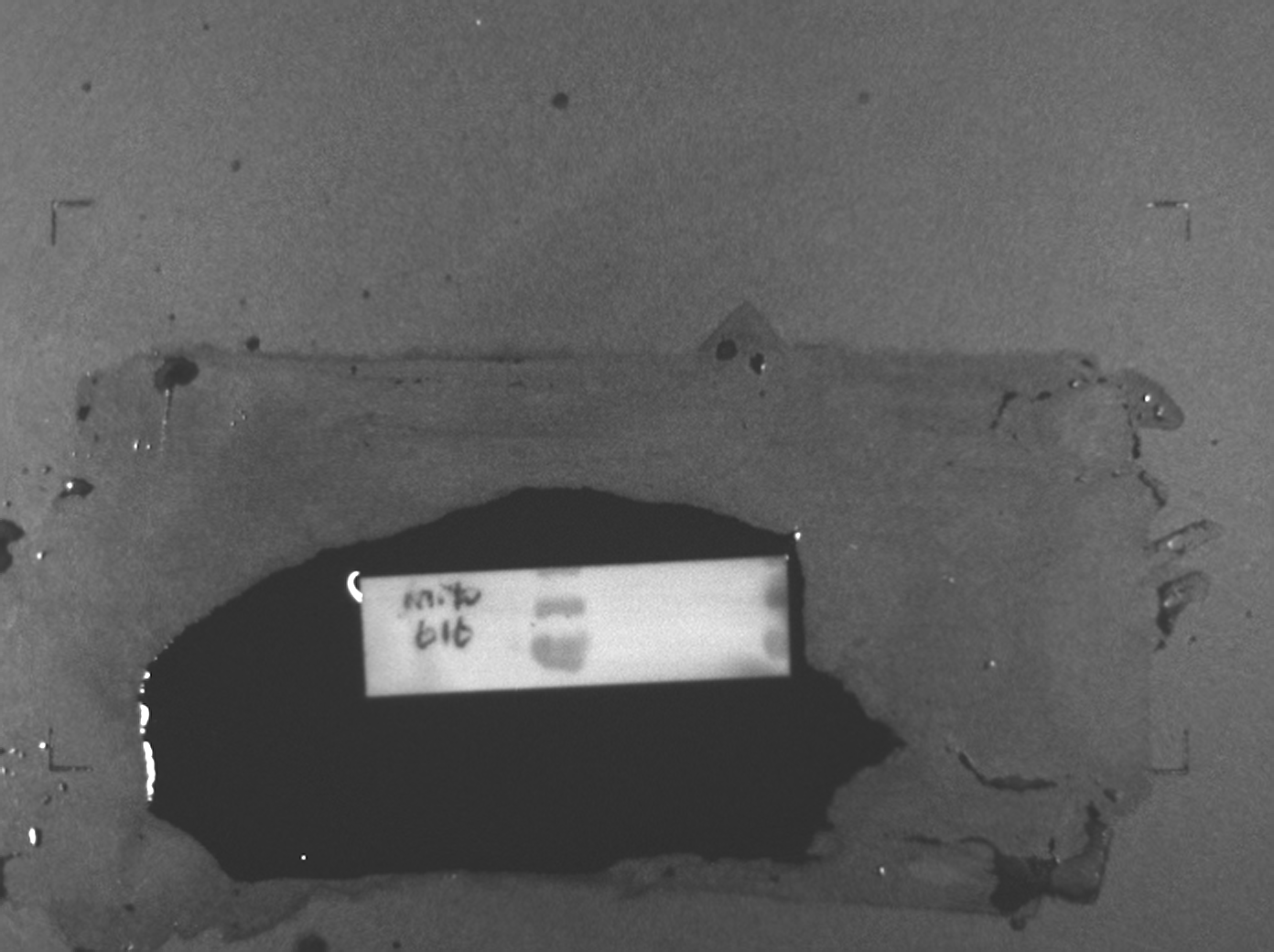

Supplement: Supplementary file 8 [file Data_Sheet_8.ZIP › mito/mito 616-4.tif]

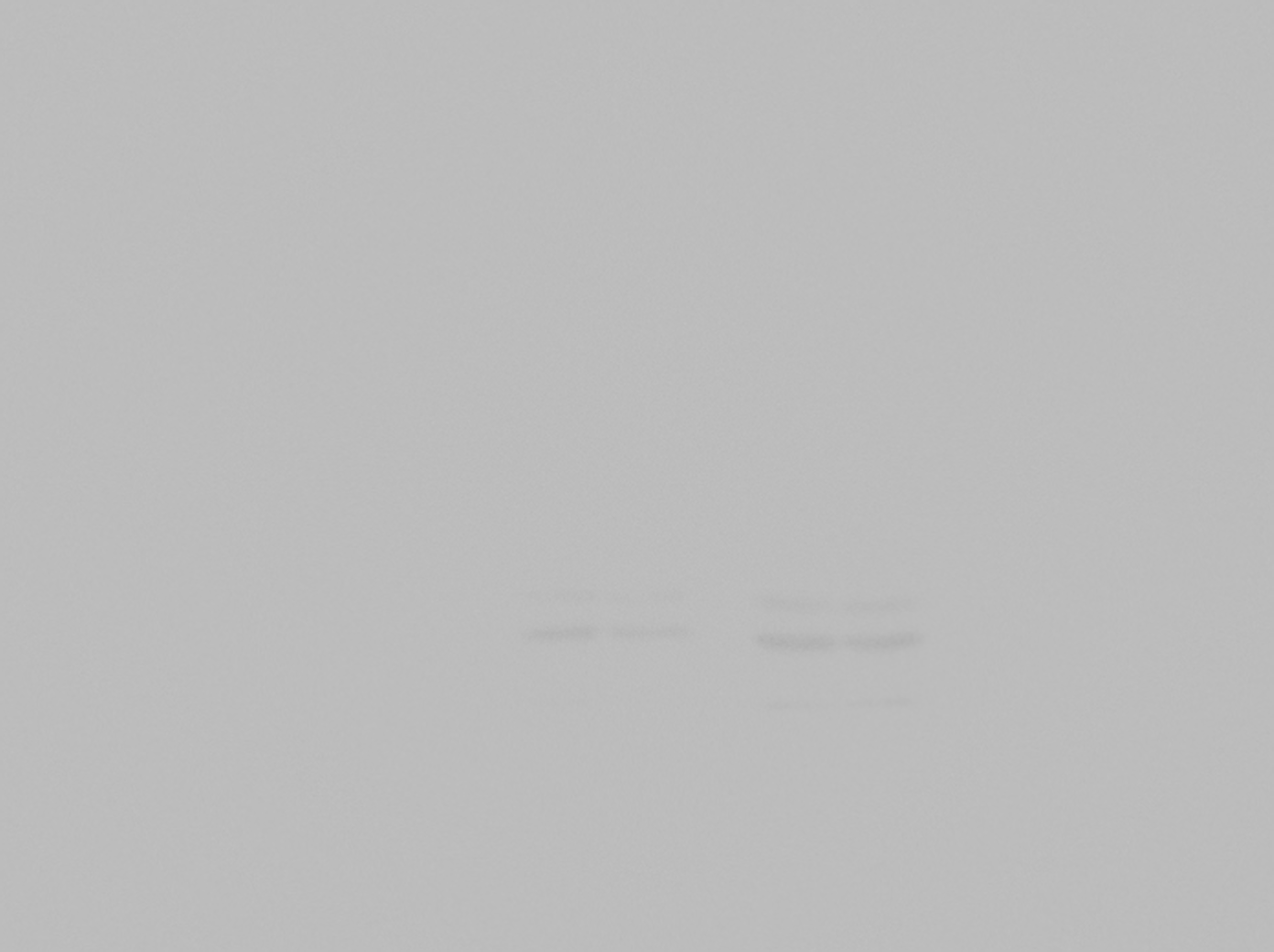

Supplement: Supplementary file 8 [file Data_Sheet_8.ZIP › mito/mito actin-1.tif]

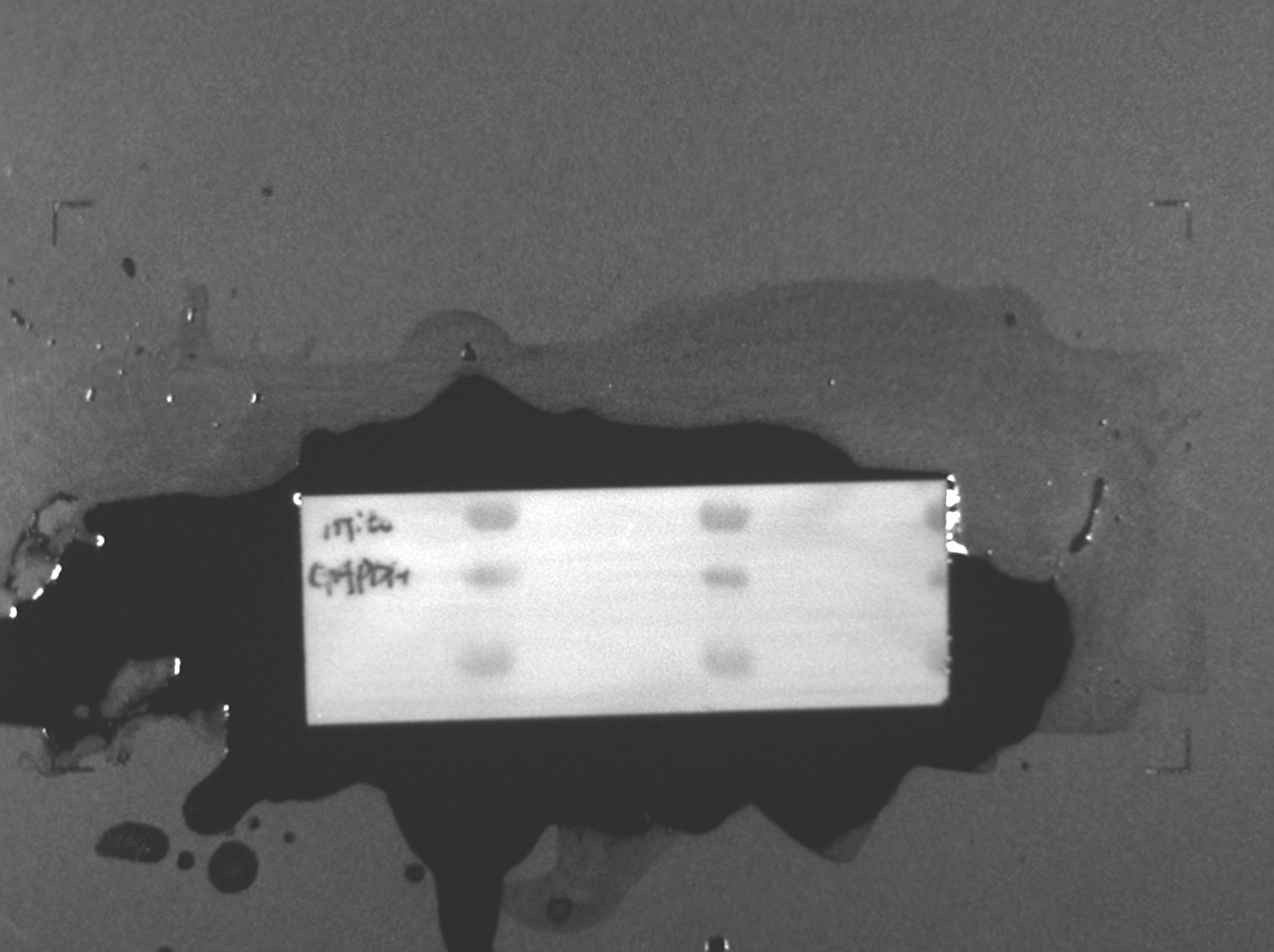

Supplement: Supplementary file 8 [file Data_Sheet_8.ZIP › mito/mito actin-2.tif]

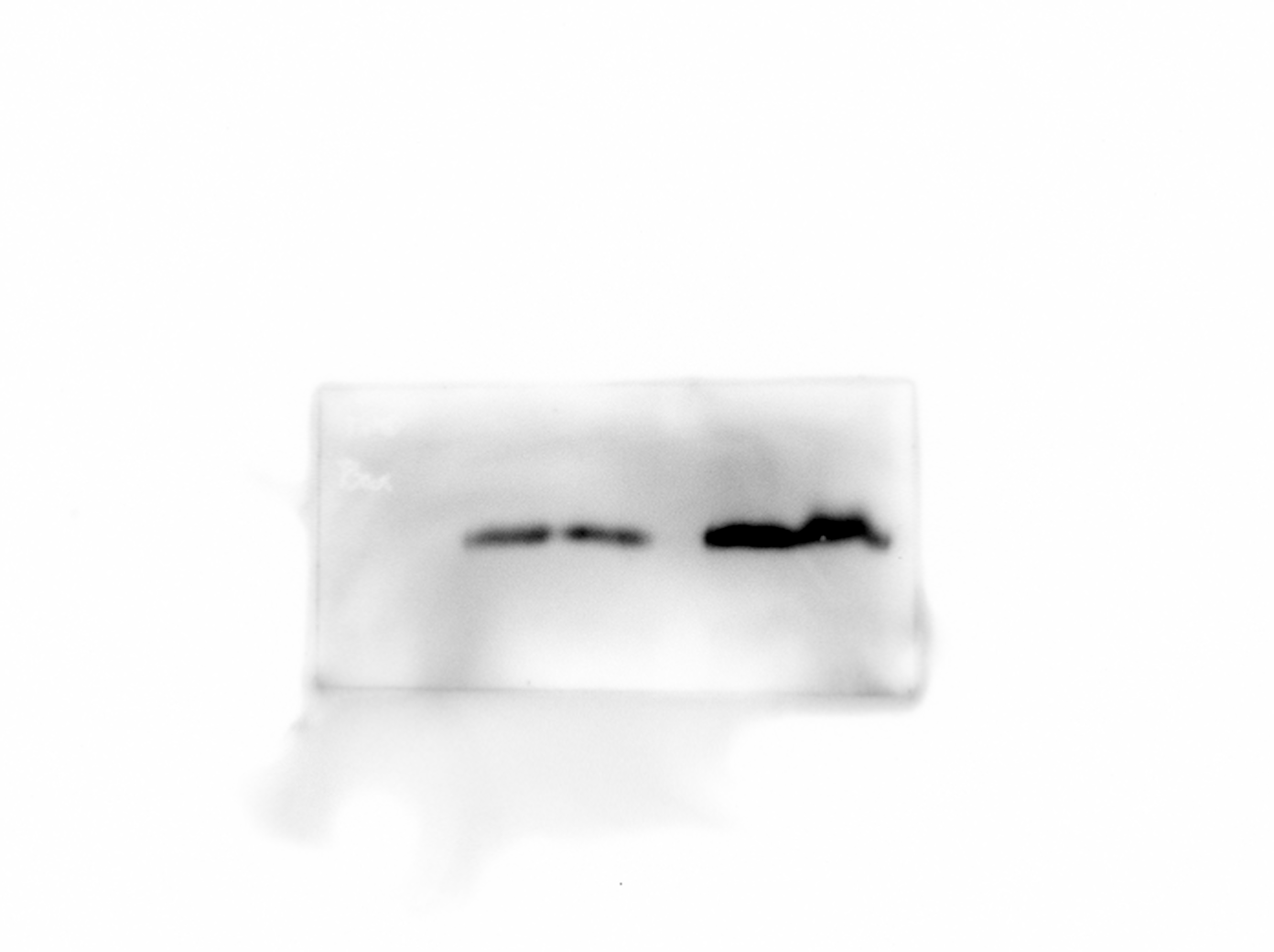

Supplement: Supplementary file 8 [file Data_Sheet_8.ZIP › mito/mito bax-1.tif]

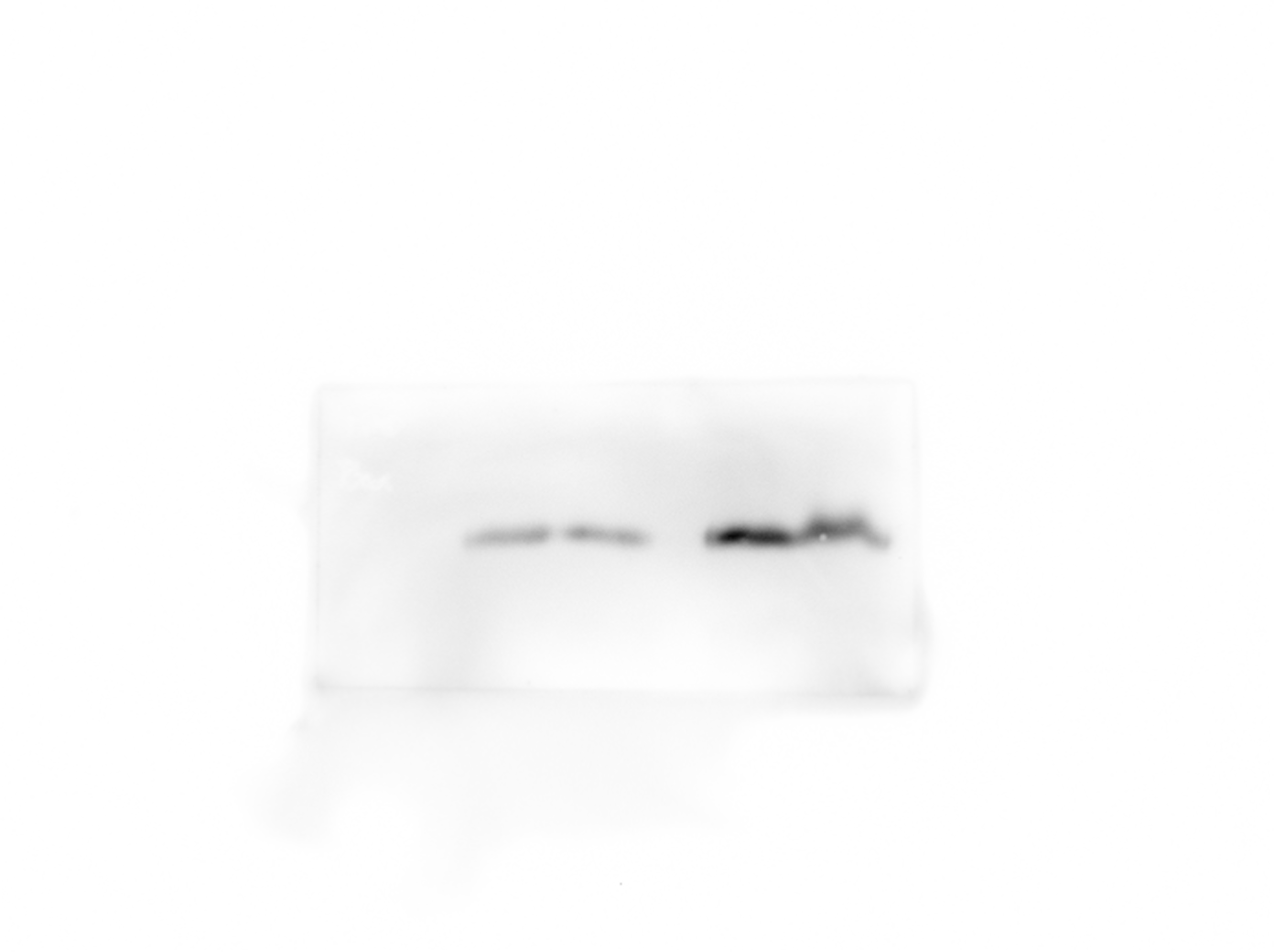

Supplement: Supplementary file 8 [file Data_Sheet_8.ZIP › mito/mito bax-2.tif]

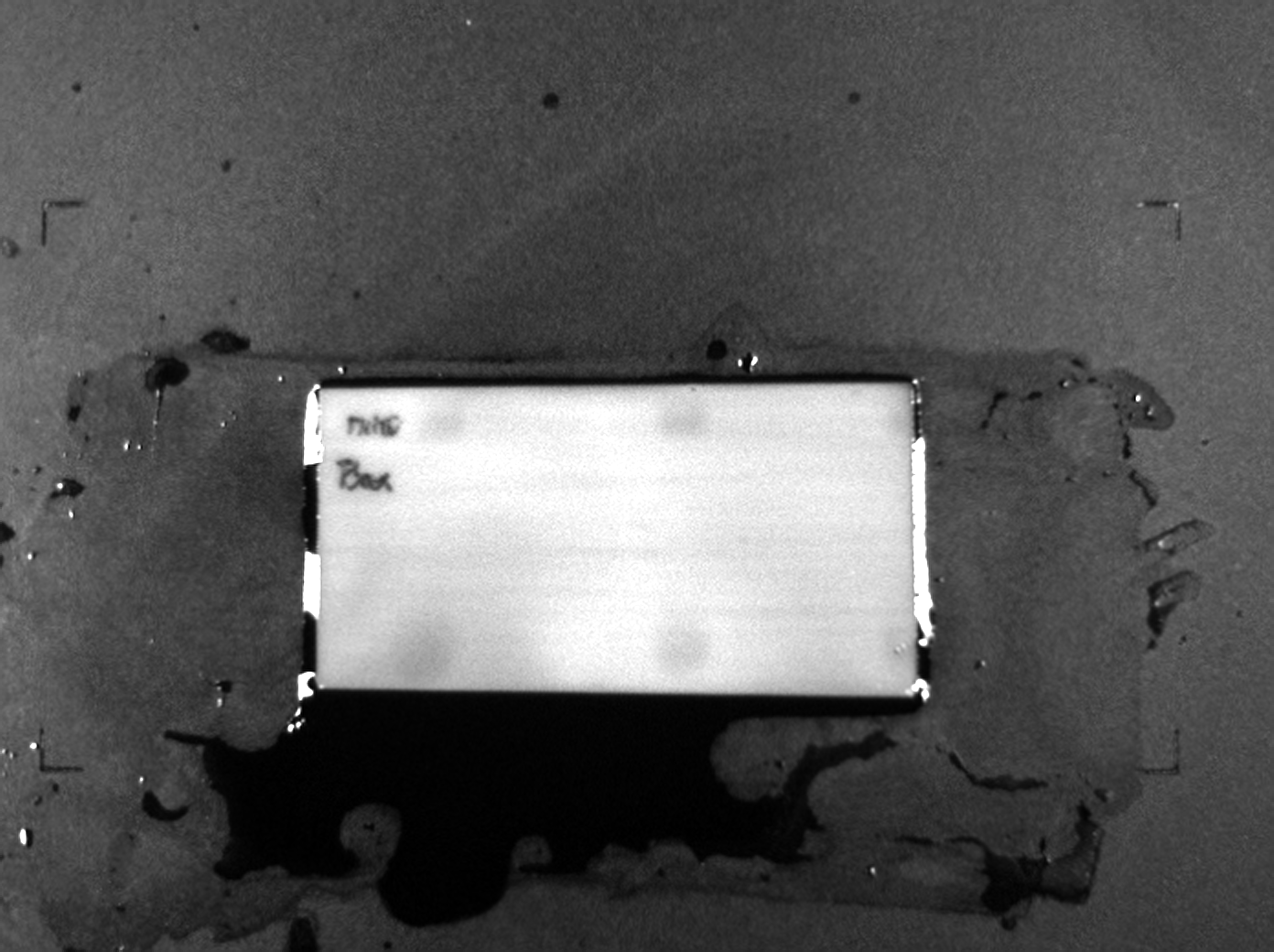

Supplement: Supplementary file 8 [file Data_Sheet_8.ZIP › mito/mito bax-3.tif]

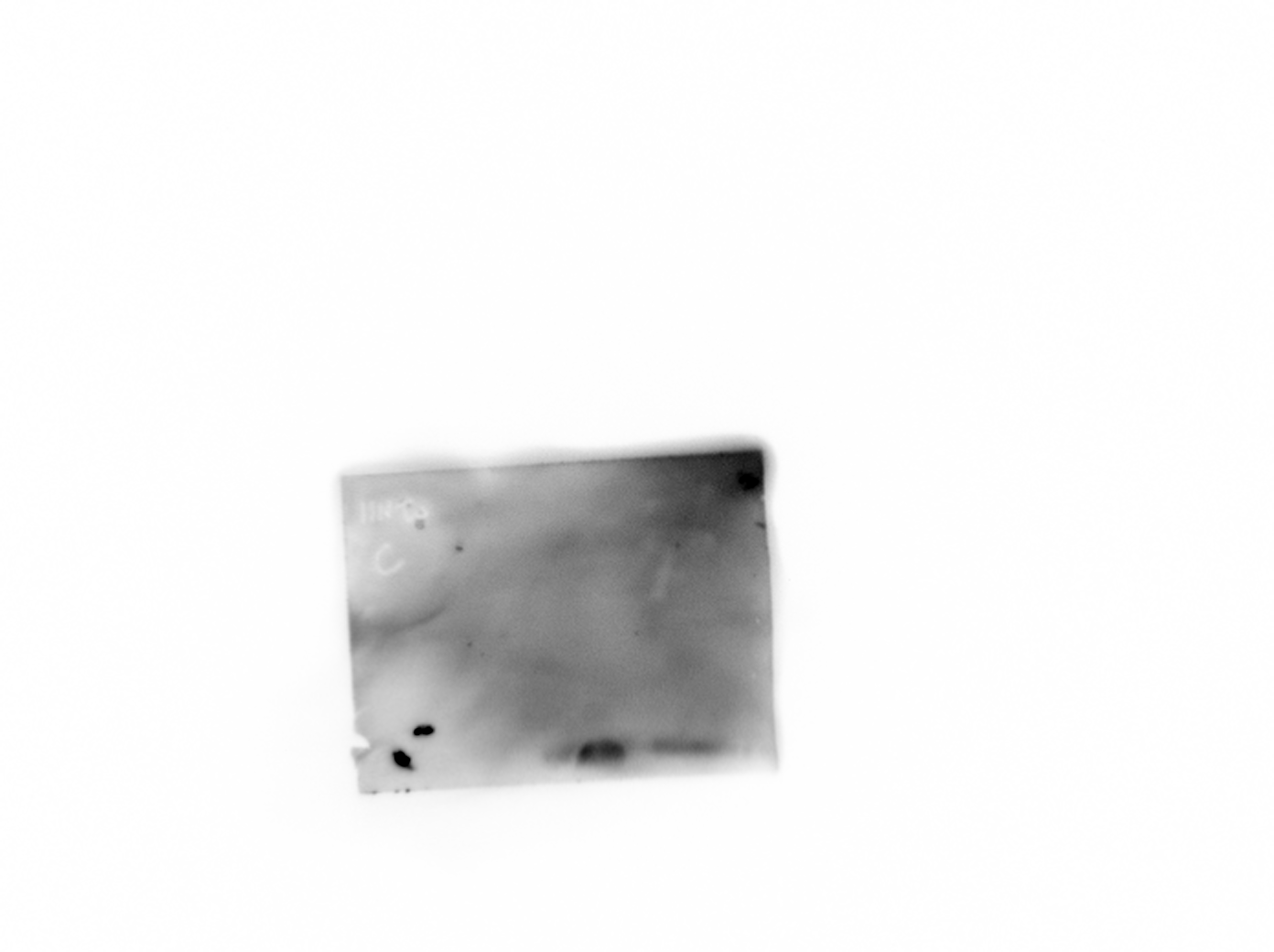

Supplement: Supplementary file 8 [file Data_Sheet_8.ZIP › mito/mito c-7.tif]

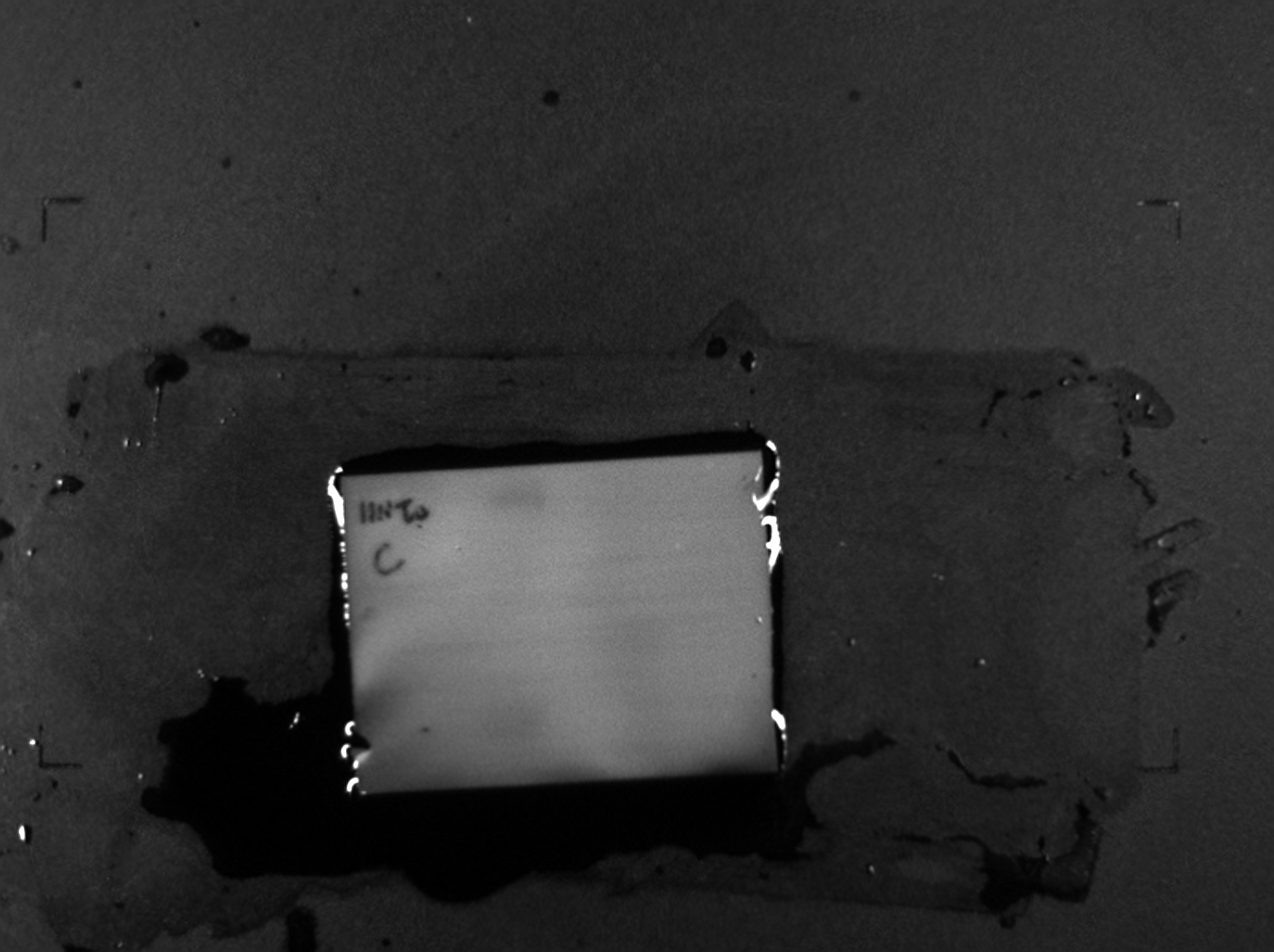

Supplement: Supplementary file 8 [file Data_Sheet_8.ZIP › mito/mito c-8.tif]

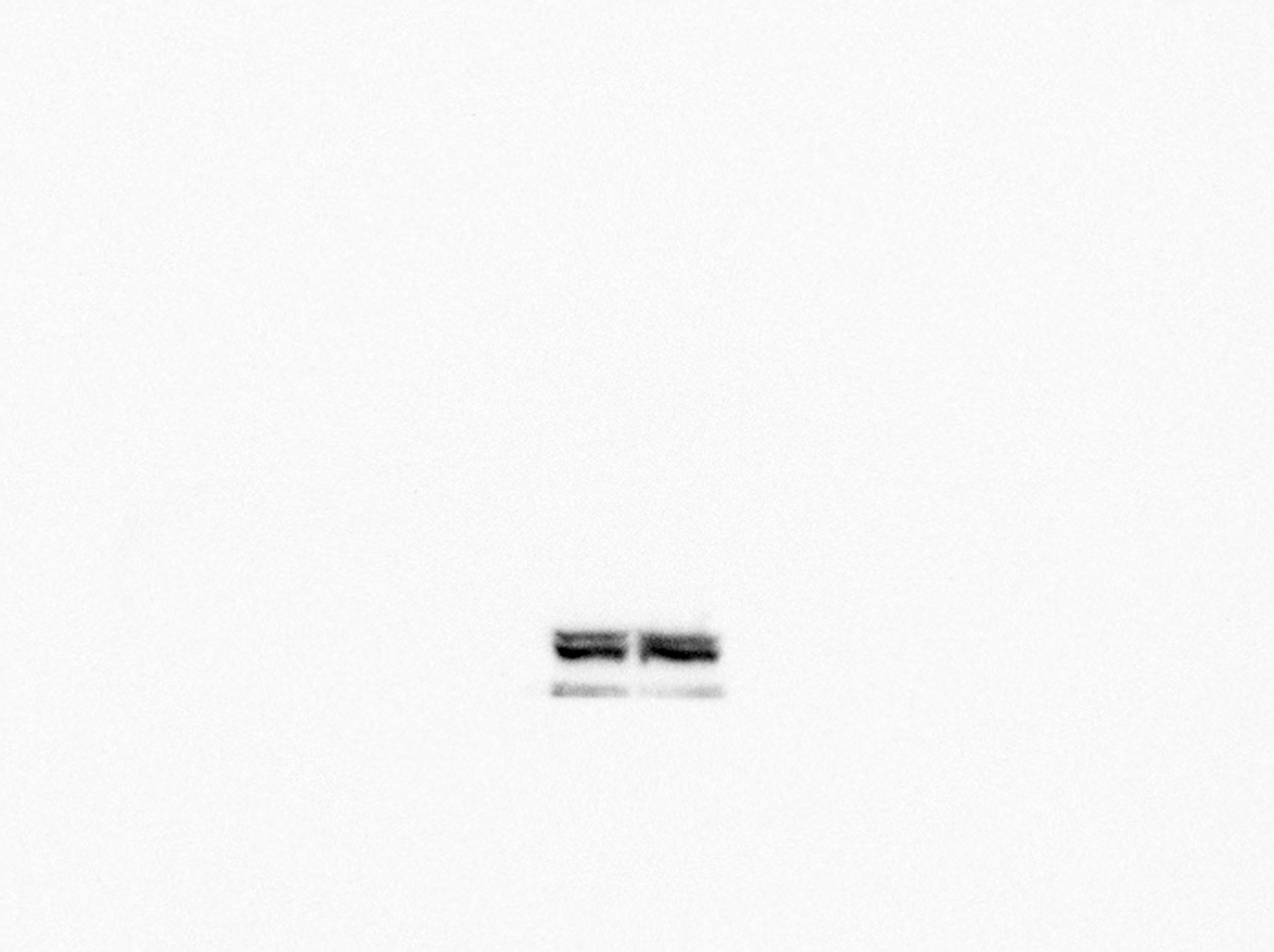

Supplement: Supplementary file 8 [file Data_Sheet_8.ZIP › mito/mito drp1-1.tif]

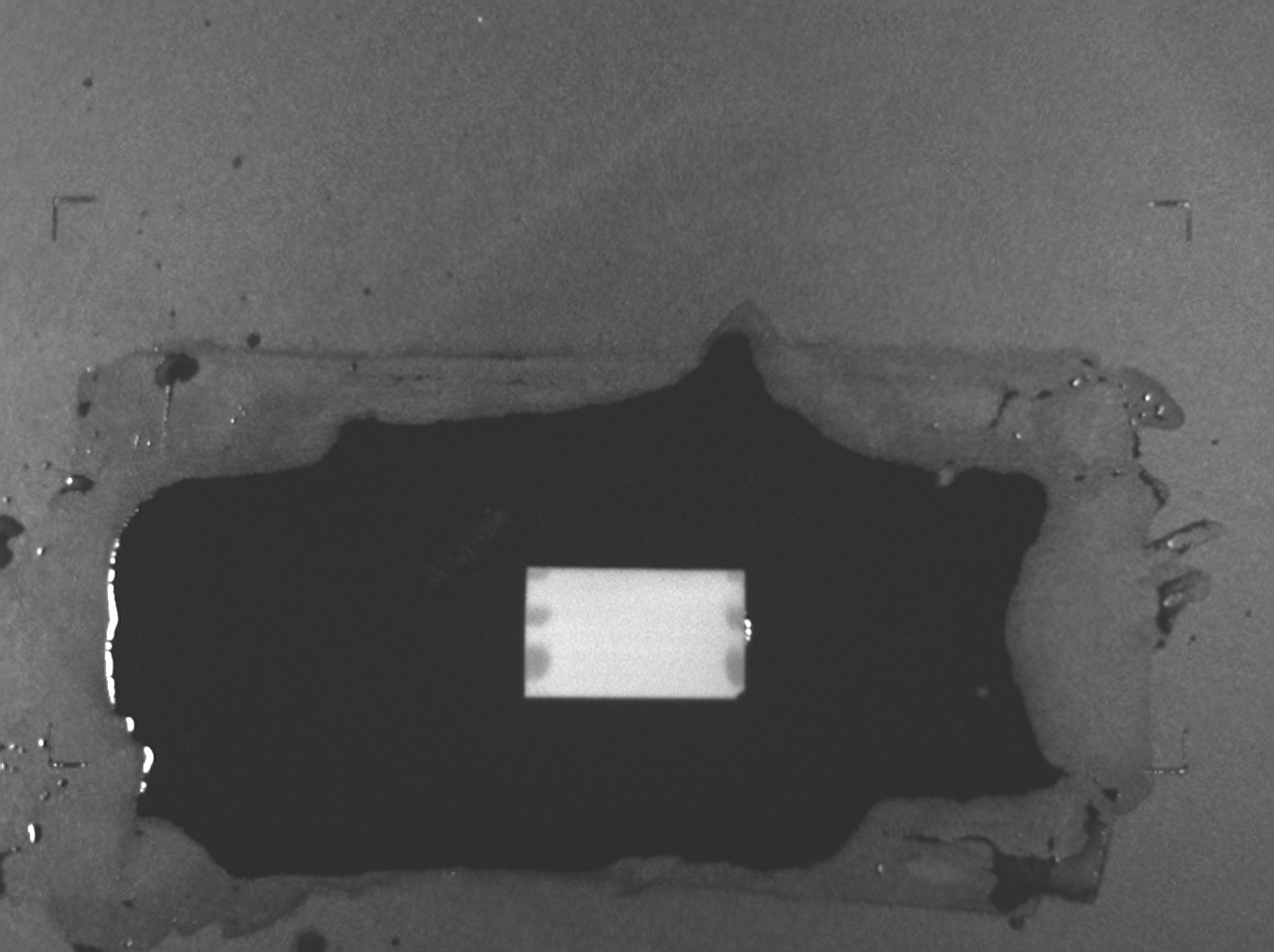

Supplement: Supplementary file 8 [file Data_Sheet_8.ZIP › mito/mito drp1-2.tif]

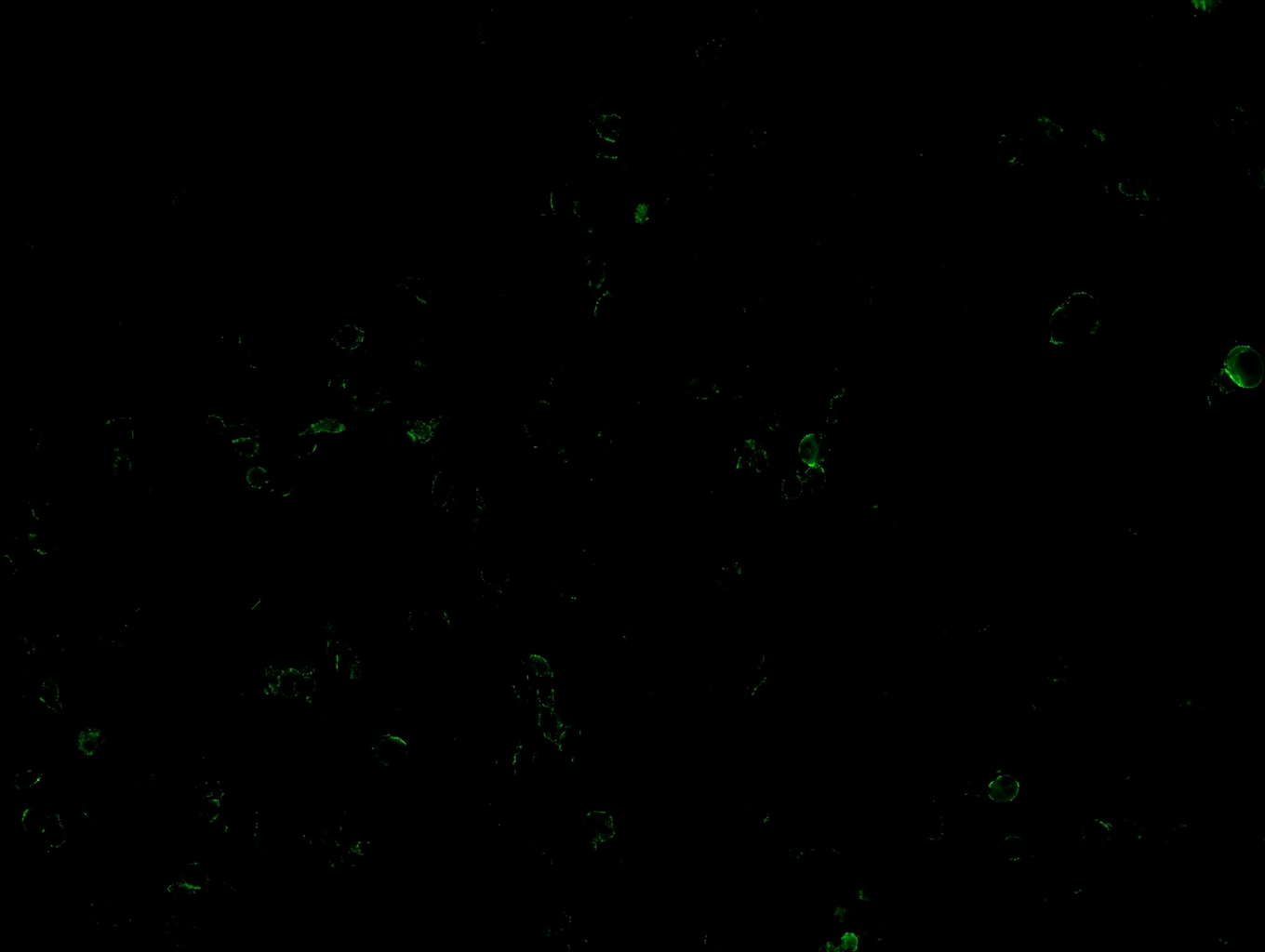

Supplement: Supplementary file 9 [file Data_Sheet_9.ZIP › dcfda/control.tif]

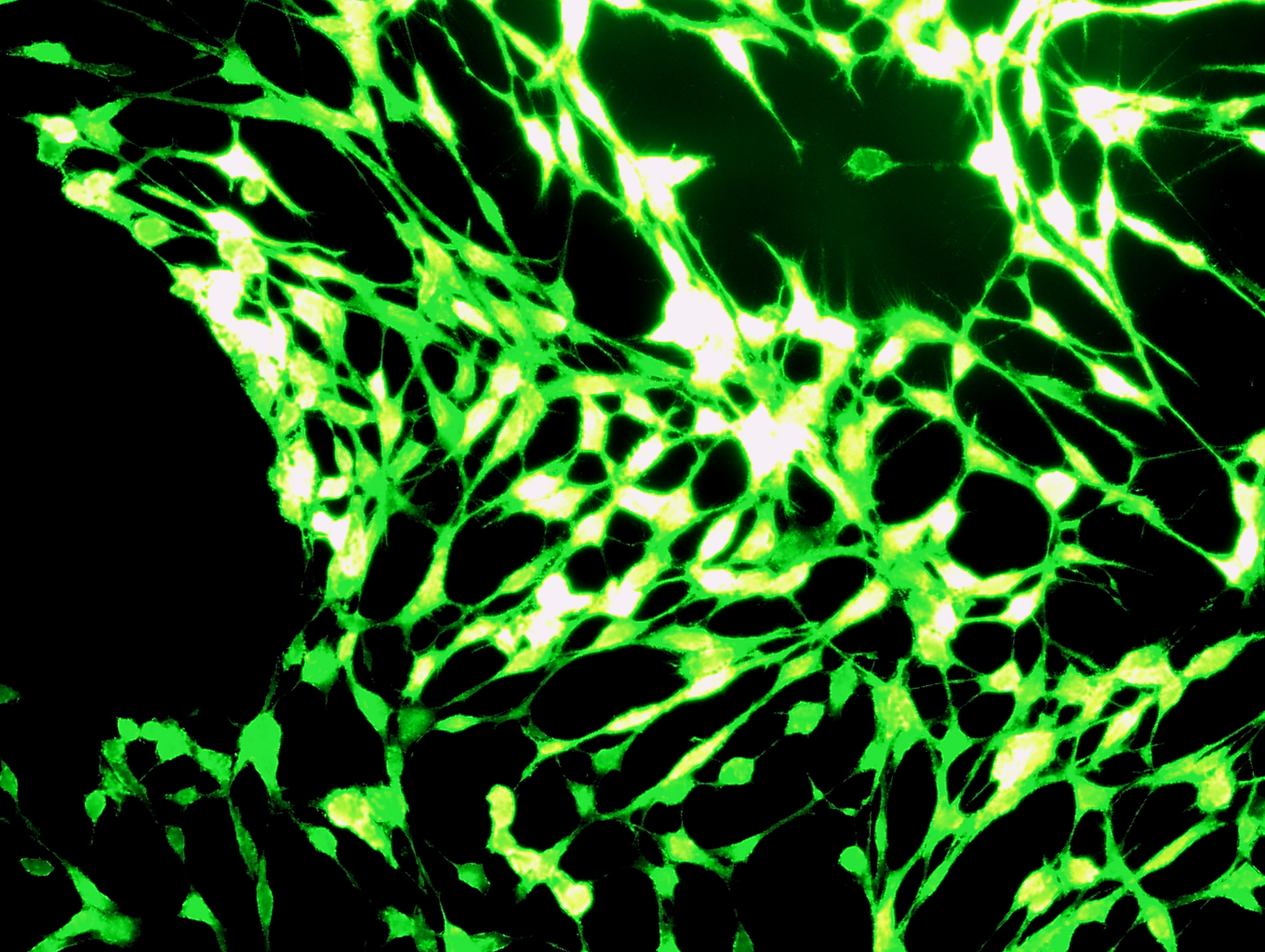

Supplement: Supplementary file 9 [file Data_Sheet_9.ZIP › dcfda/hypoxia.tif]

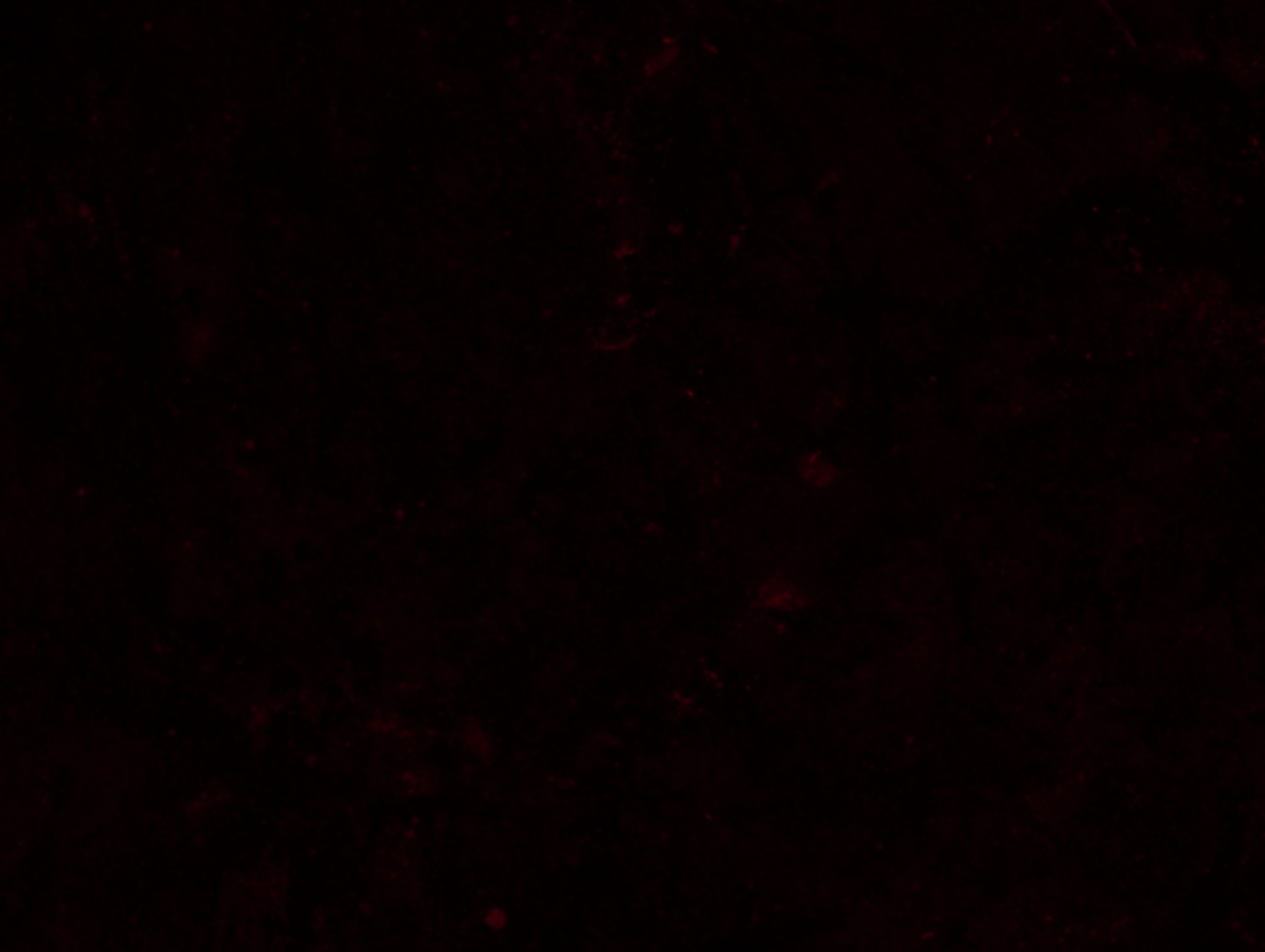

Supplement: Supplementary file 9 [file Data_Sheet_9.ZIP › DHE/control.tif]

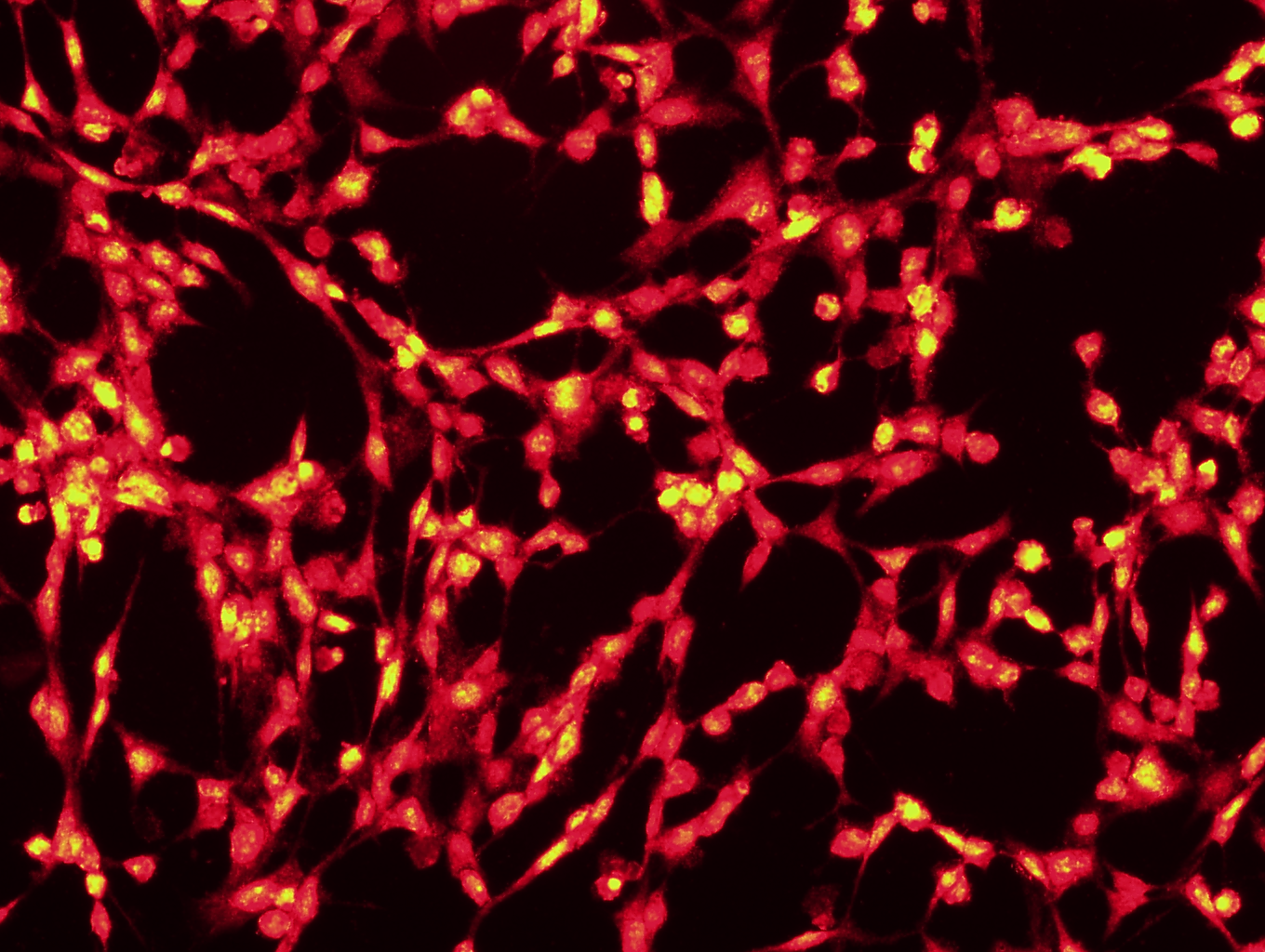

Supplement: Supplementary file 9 [file Data_Sheet_9.ZIP › DHE/hypoxia.tif]

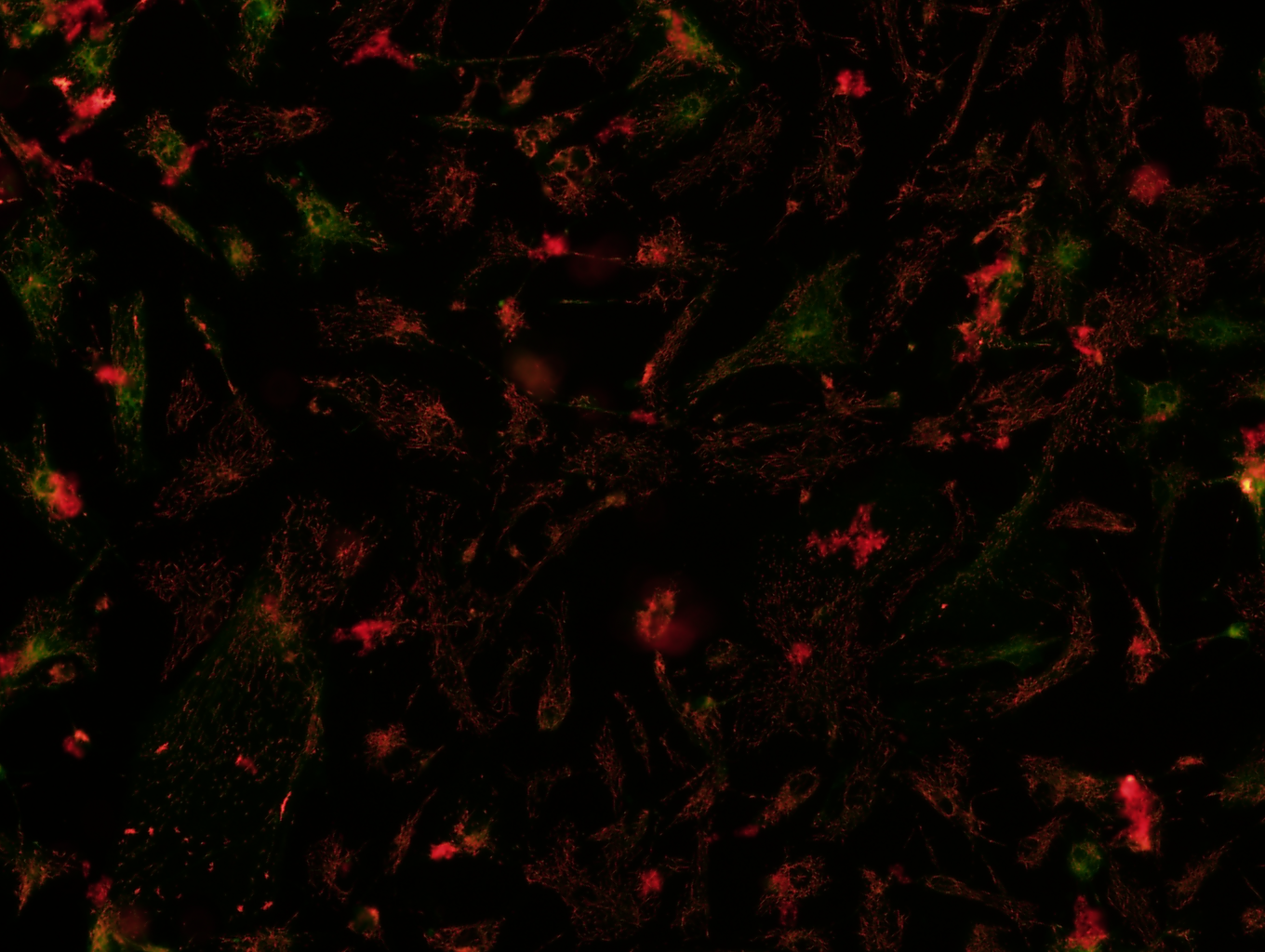

Supplement: Supplementary file 9 [file Data_Sheet_9.ZIP › jc1/control.tif]

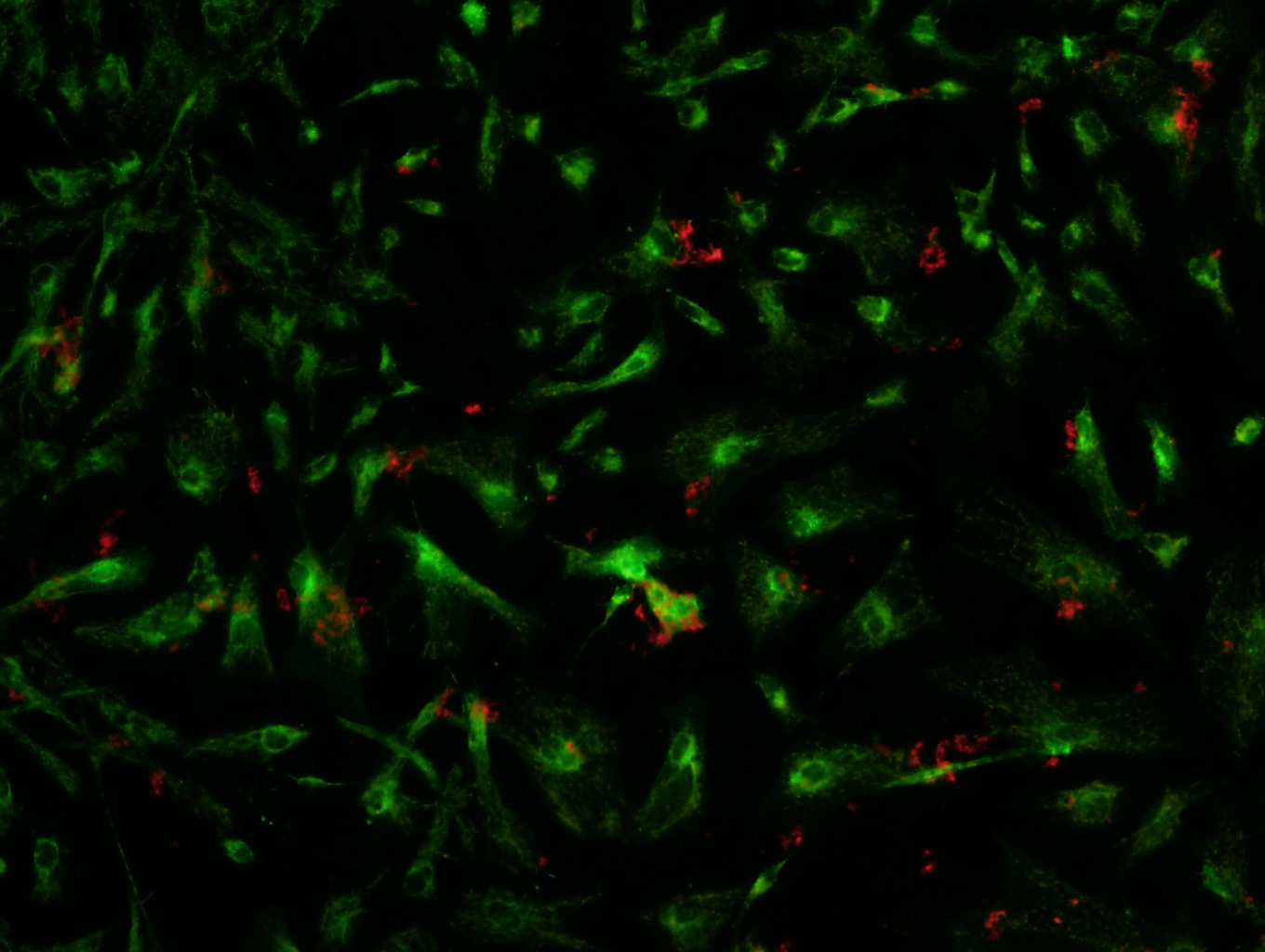

Supplement: Supplementary file 9 [file Data_Sheet_9.ZIP › jc1/hypoxia.tif]
