# Supplementary figures and images for: High-altitude cerebral hypoxia promotes mitochondrial dysfunction and apoptosis of mouse neurons (part 2 of 2)
Source: Front Mol Neurosci. 2023 Jul 12;16:1216947. doi: 10.3389/fnmol.2023.1216947 (PMC10370763; doi:10.3389/fnmol.2023.1216947)

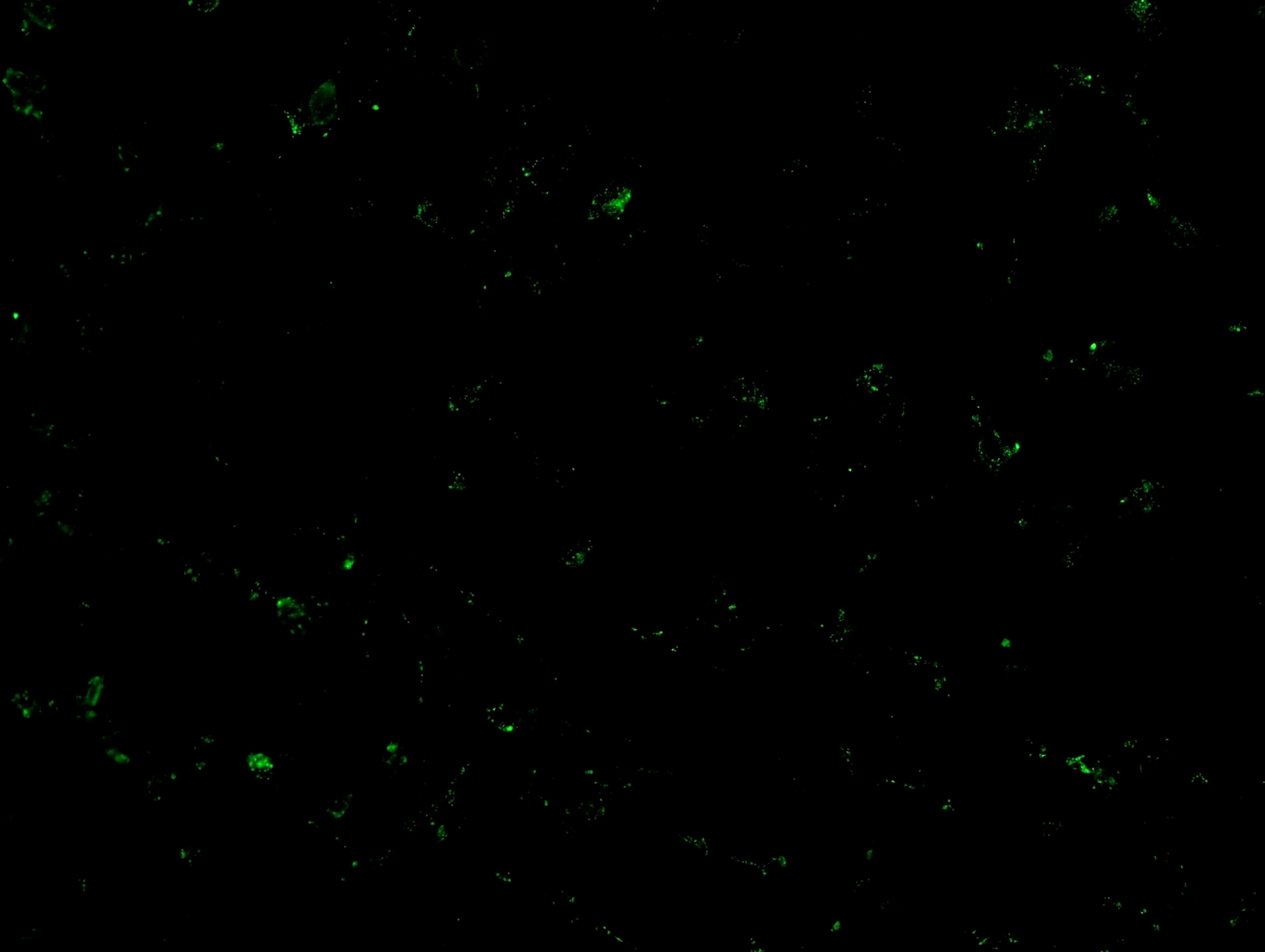

Supplement: Supplementary file 9 [file Data_Sheet_9.ZIP › mptp/control.tif]

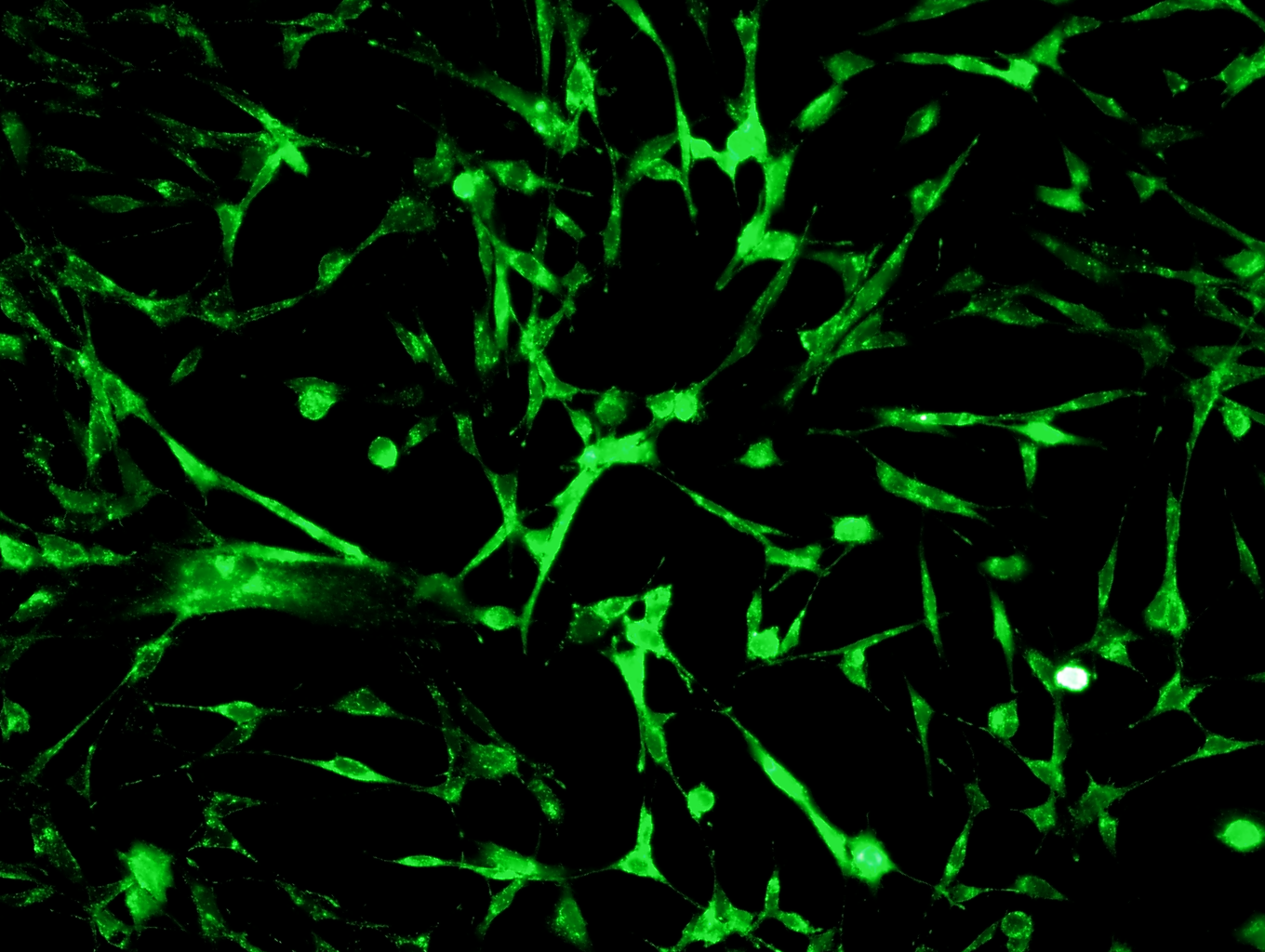

Supplement: Supplementary file 9 [file Data_Sheet_9.ZIP › mptp/hypoxia.tif]

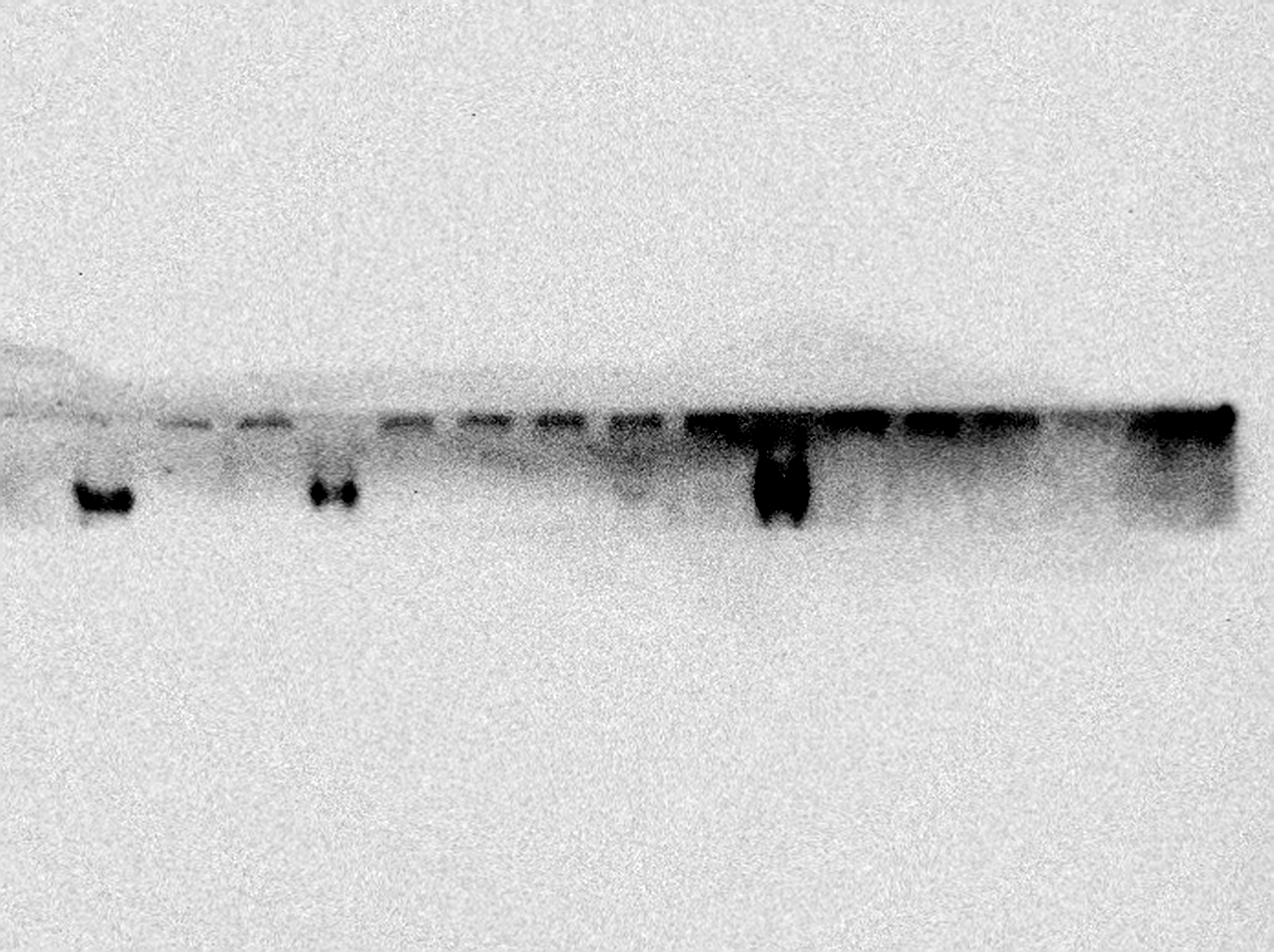

Supplement: Supplementary file 10 [file Data_Sheet_10.ZIP › 1-616-1.tif]

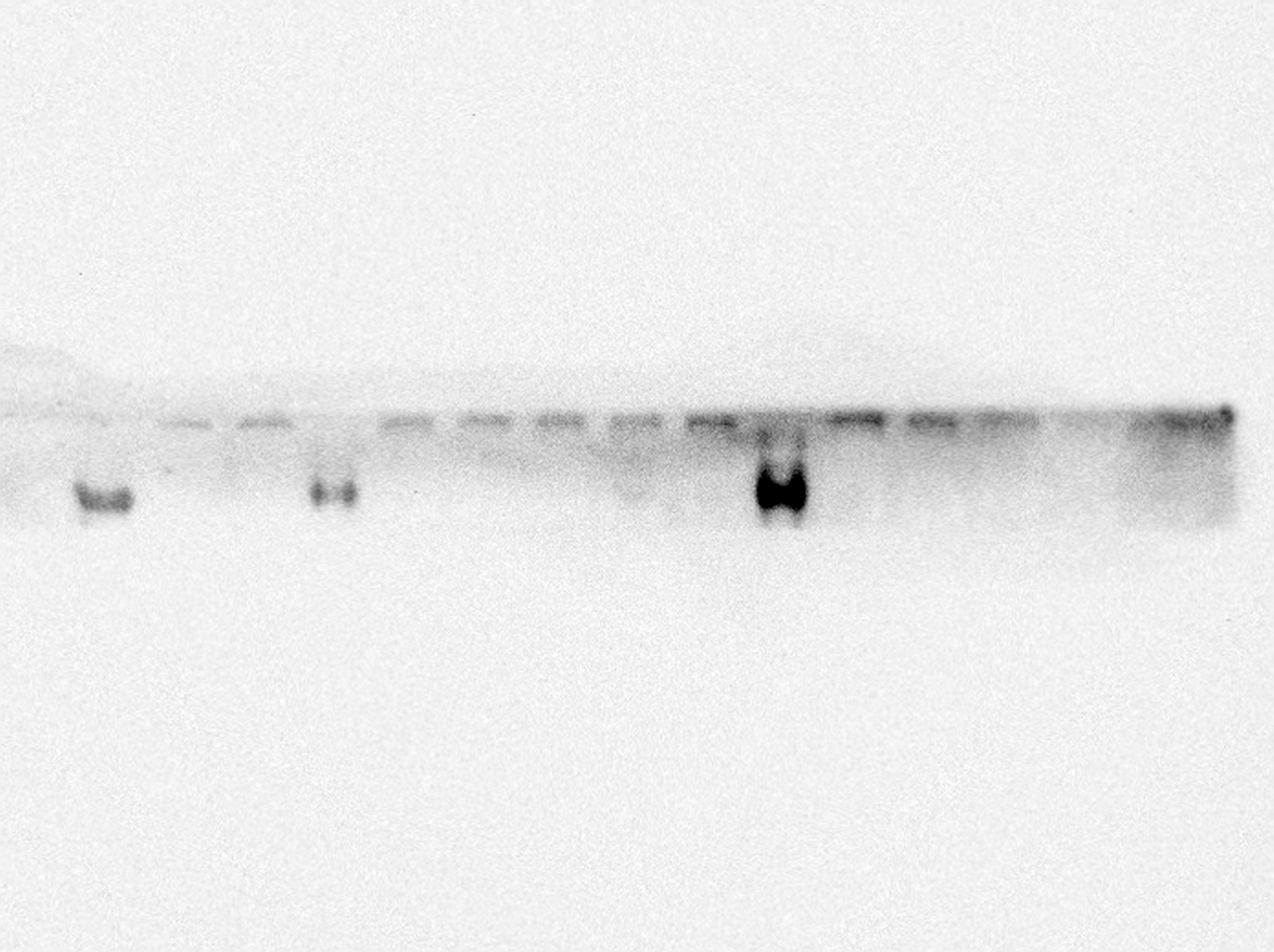

Supplement: Supplementary file 10 [file Data_Sheet_10.ZIP › 1-616-2.tif]

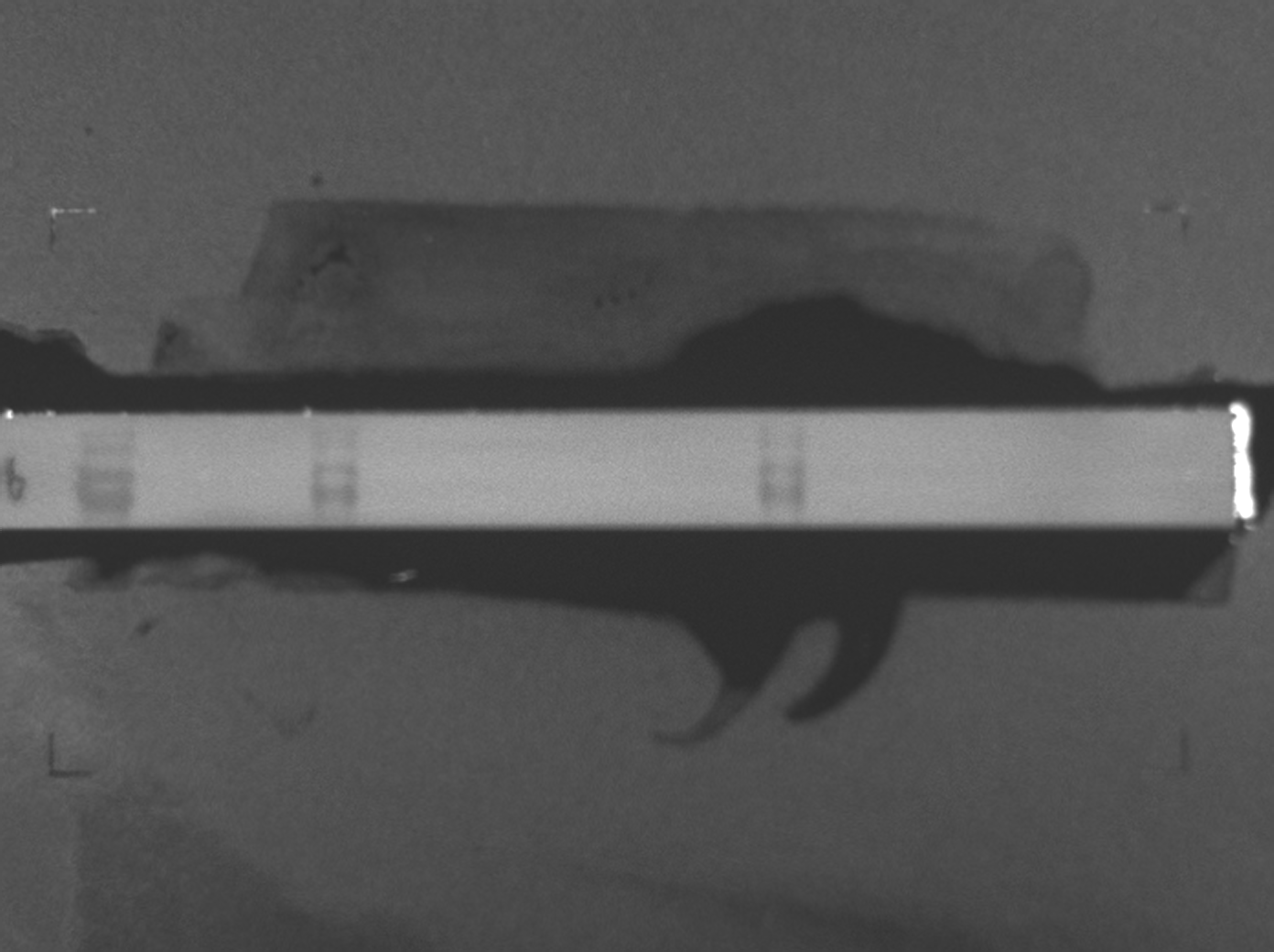

Supplement: Supplementary file 10 [file Data_Sheet_10.ZIP › 1-616-4.tif]

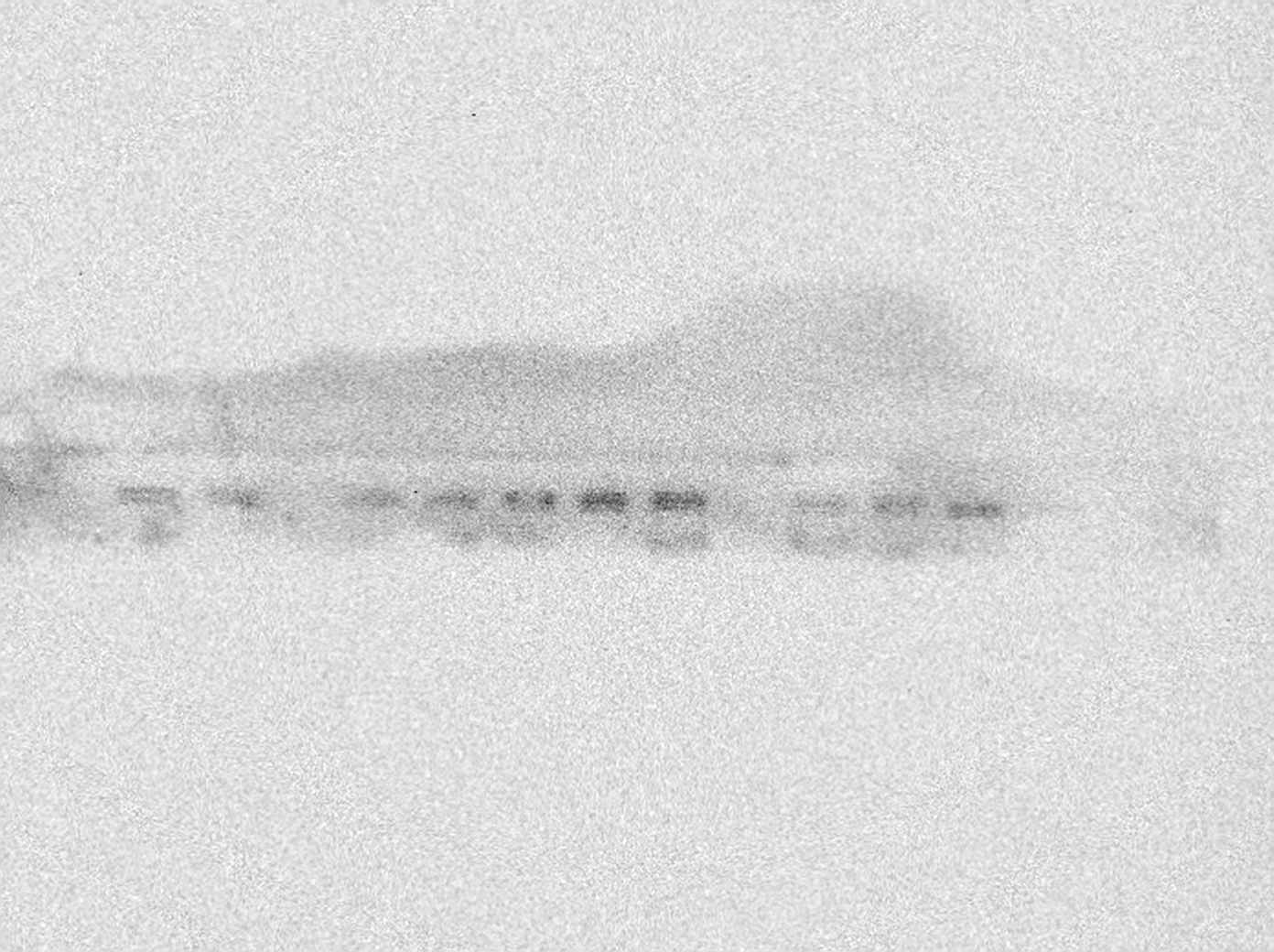

Supplement: Supplementary file 10 [file Data_Sheet_10.ZIP › 2-637-1.tif]

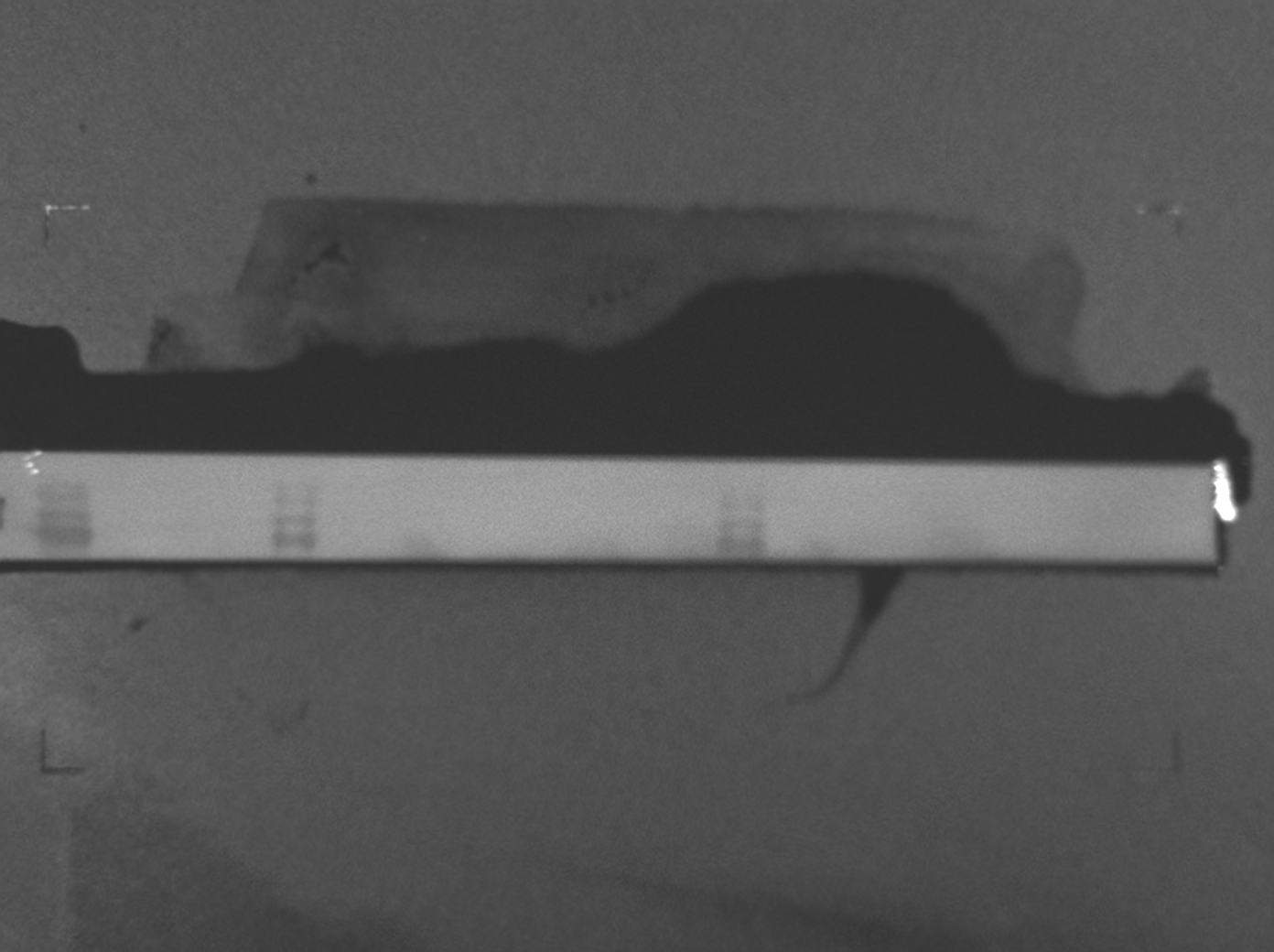

Supplement: Supplementary file 10 [file Data_Sheet_10.ZIP › 2-637-2.tif]

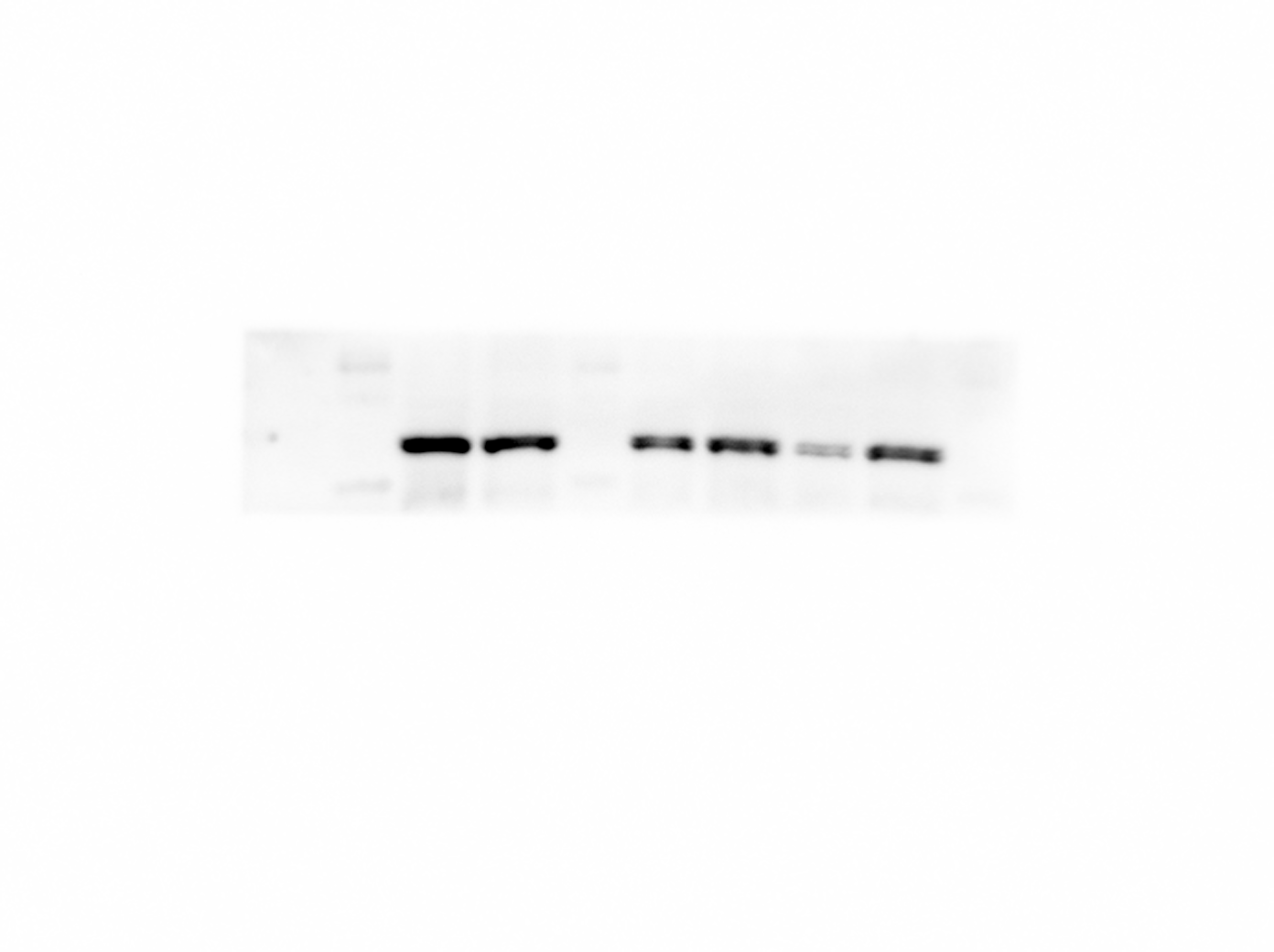

Supplement: Supplementary file 10 [file Data_Sheet_10.ZIP › actin.tif]

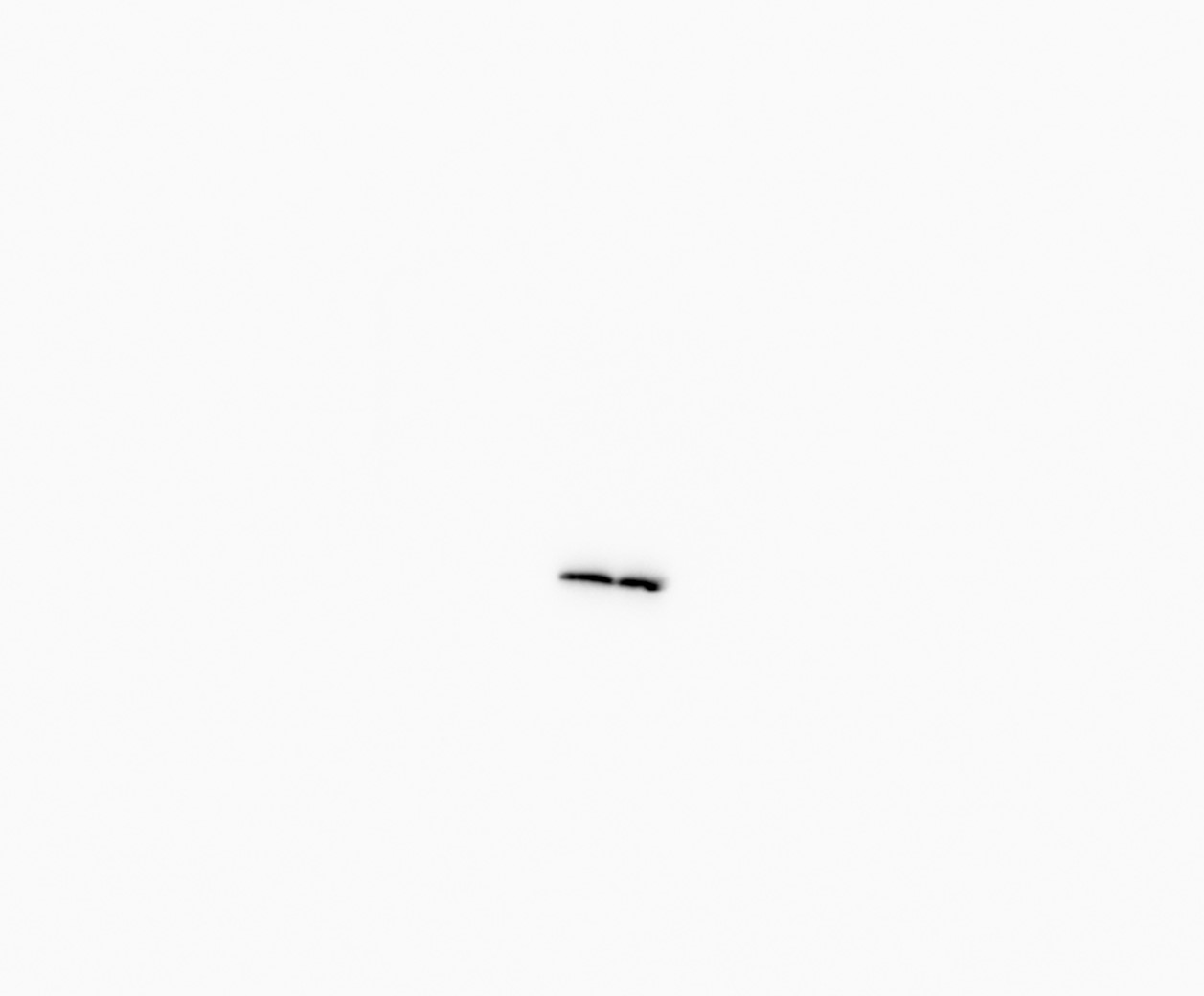

Supplement: Supplementary file 10 [file Data_Sheet_10.ZIP › drp1/IM002099 (2).jpg]

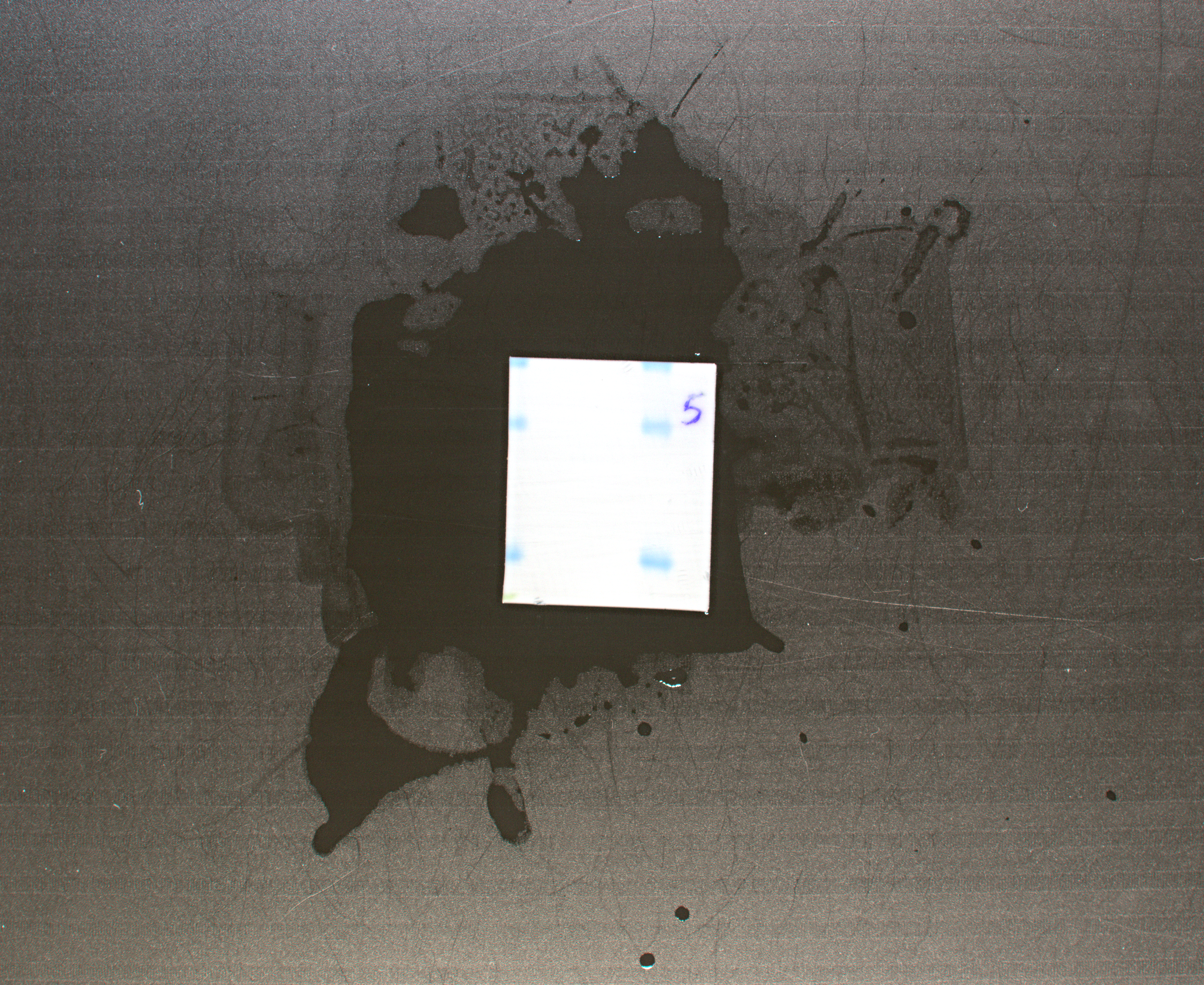

Supplement: Supplementary file 10 [file Data_Sheet_10.ZIP › drp1/IM002100.Tif]

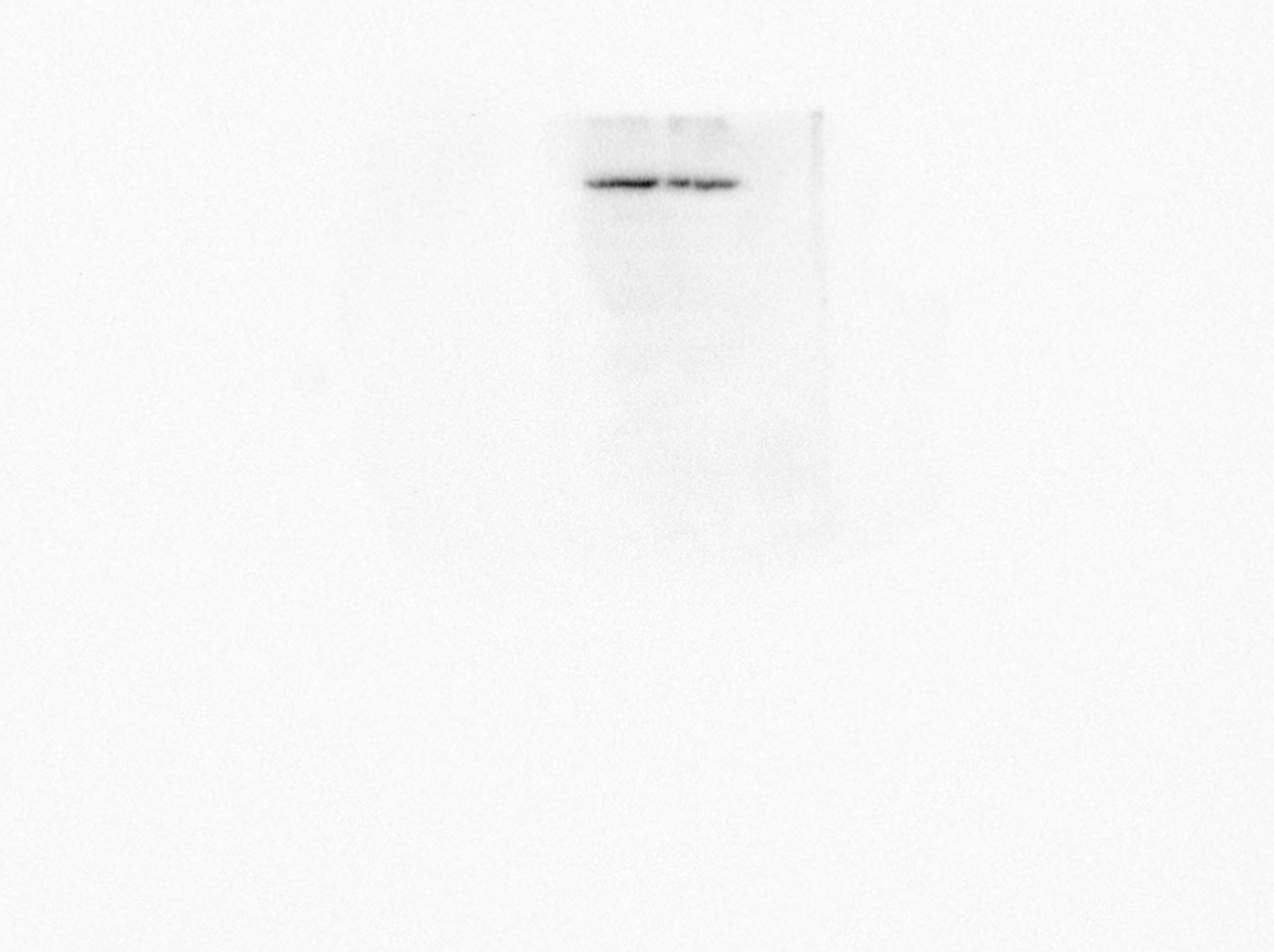

Supplement: Supplementary file 10 [file Data_Sheet_10.ZIP › mfn2-1.tif]

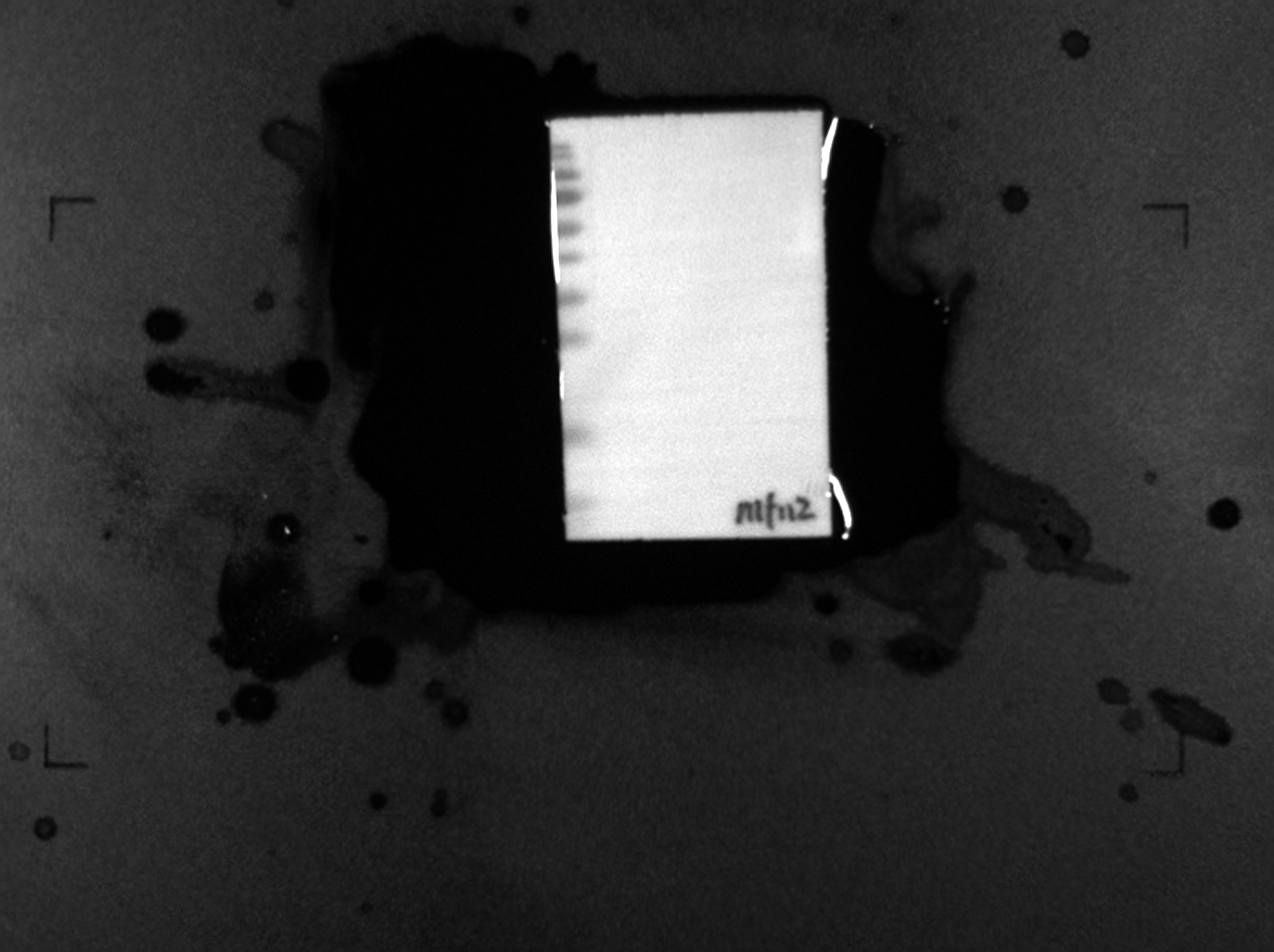

Supplement: Supplementary file 10 [file Data_Sheet_10.ZIP › mfn2-2.tif]

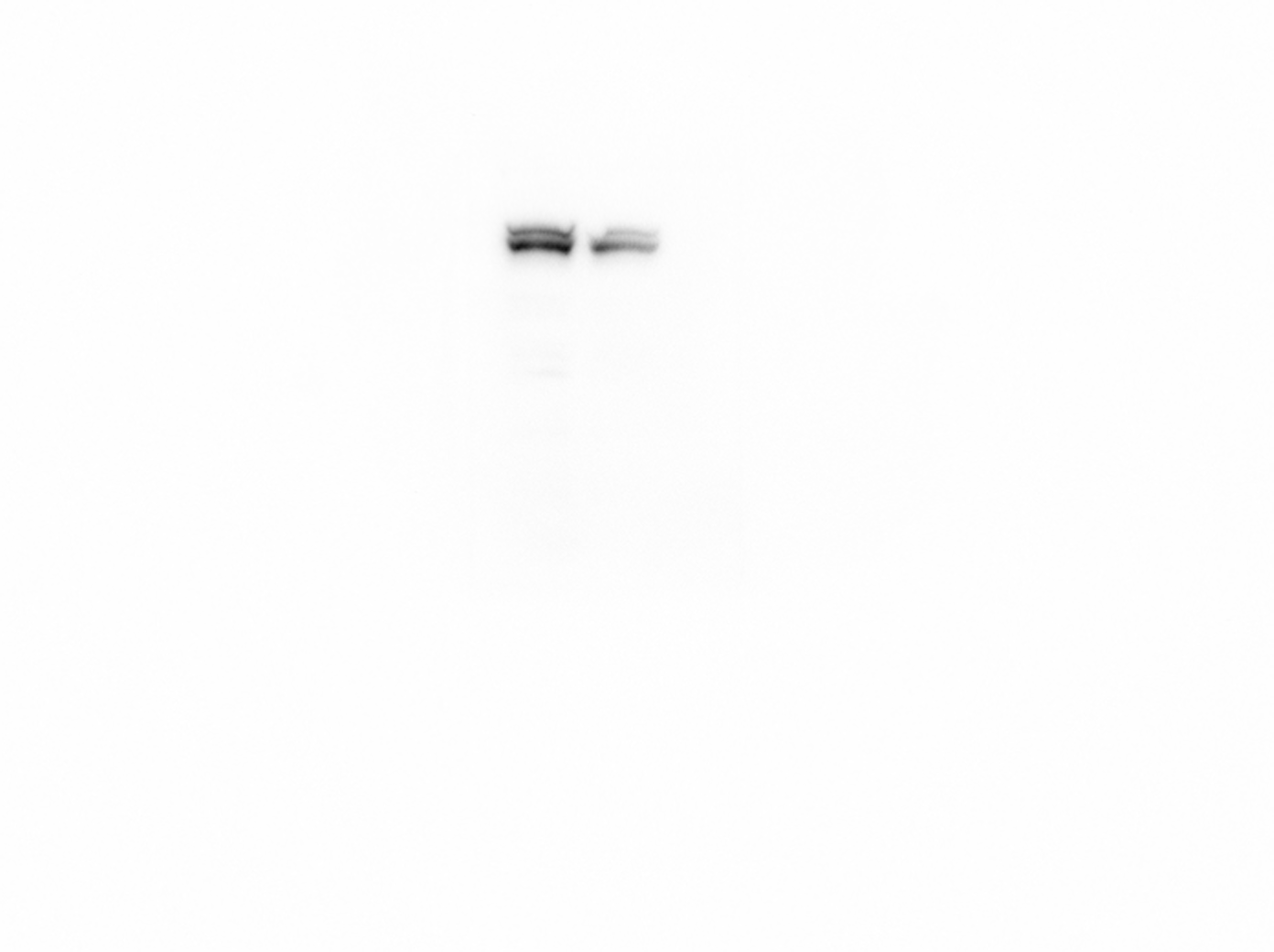

Supplement: Supplementary file 10 [file Data_Sheet_10.ZIP › opa-1.tif]

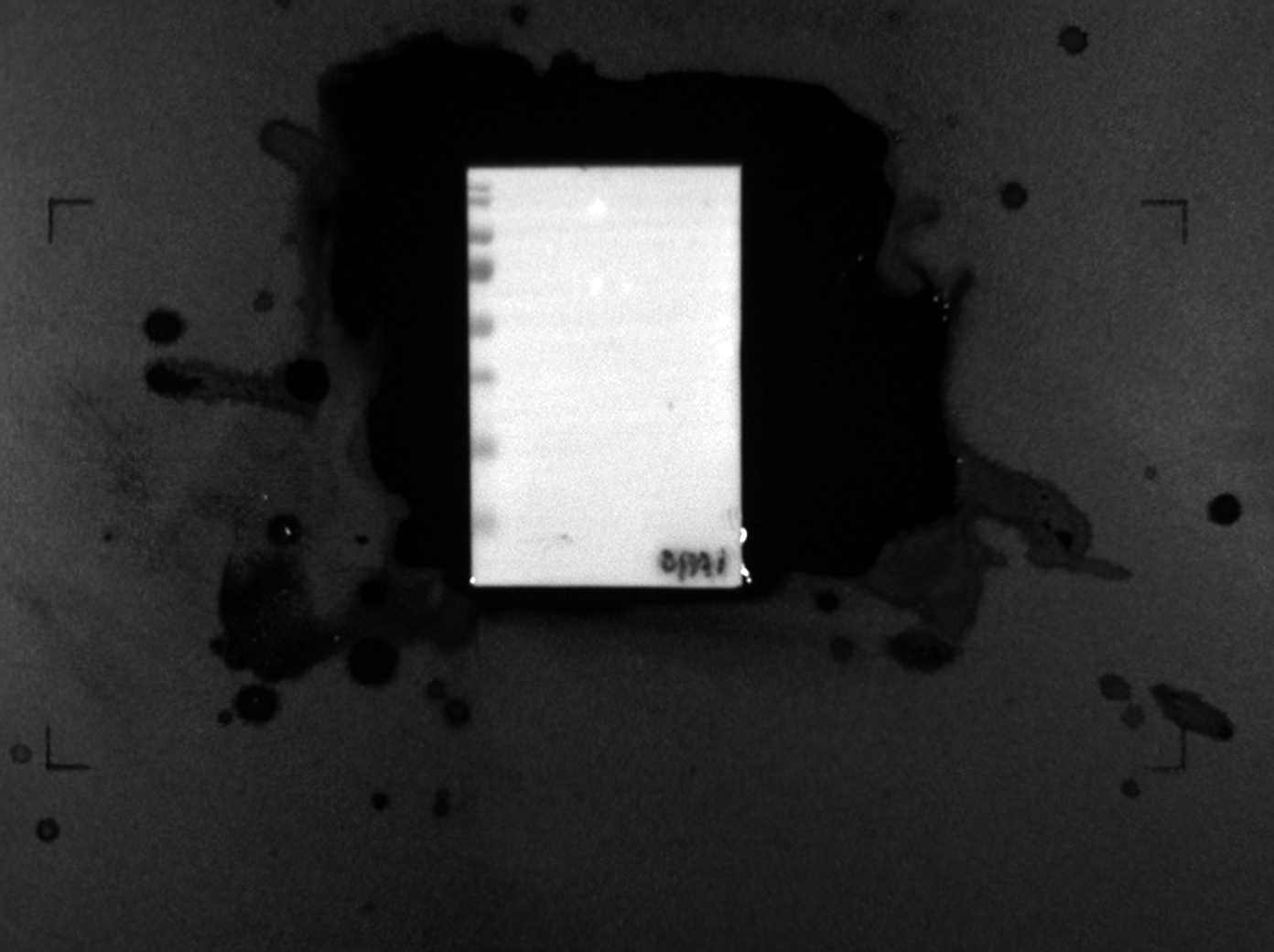

Supplement: Supplementary file 10 [file Data_Sheet_10.ZIP › opa-2.tif]

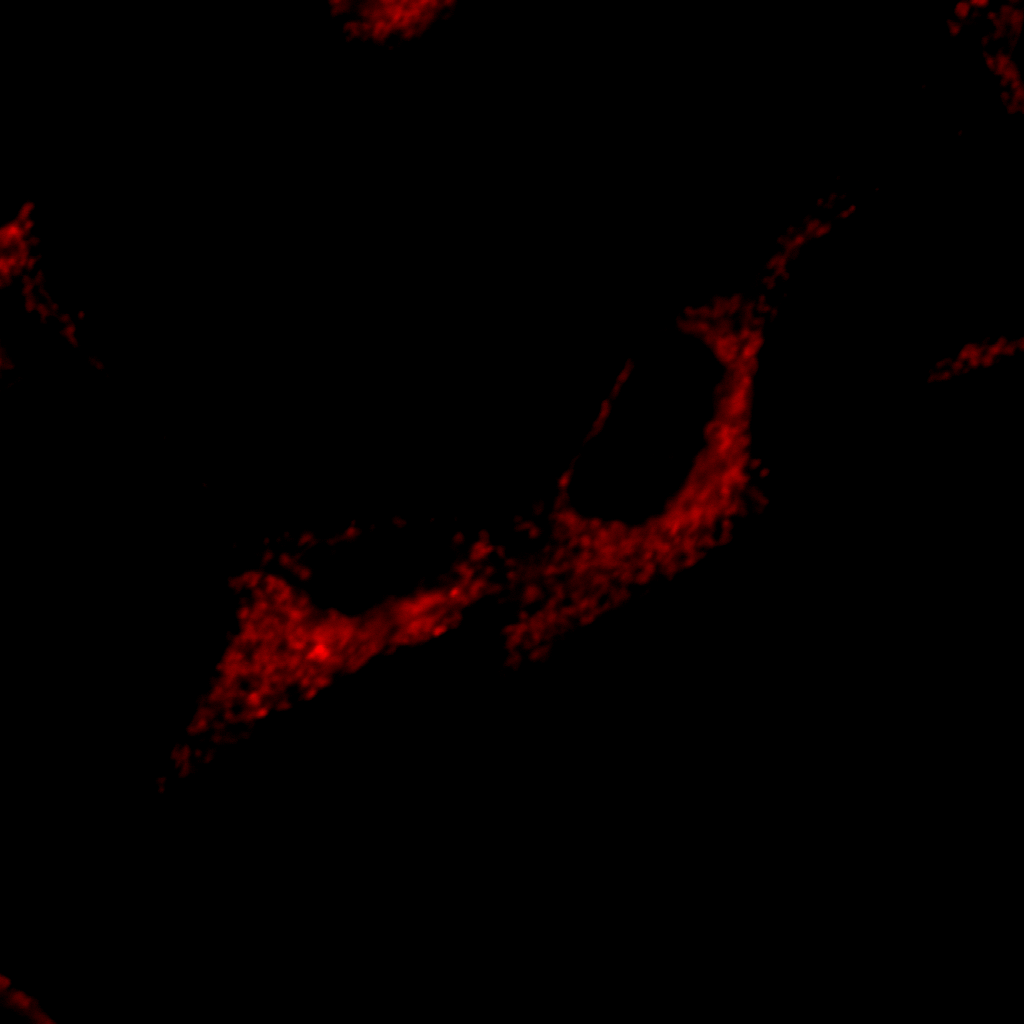

Supplement: Supplementary file 11 [file Data_Sheet_11.ZIP › control/1-2X180---All.tif]

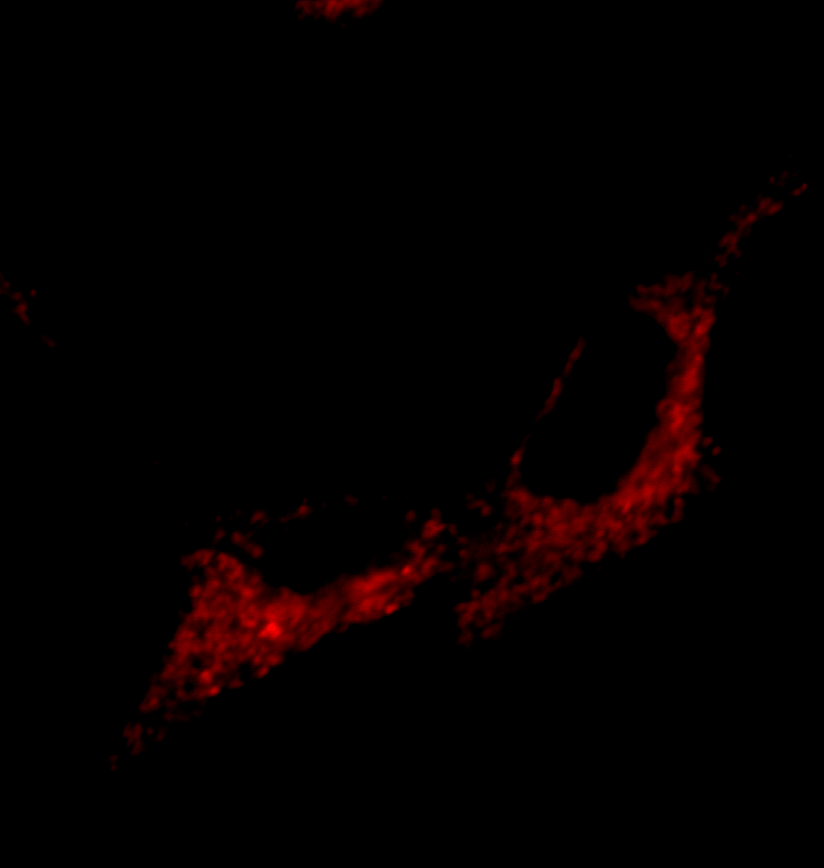

Supplement: Supplementary file 11 [file Data_Sheet_11.ZIP › control/1-2X180---montage.tif]

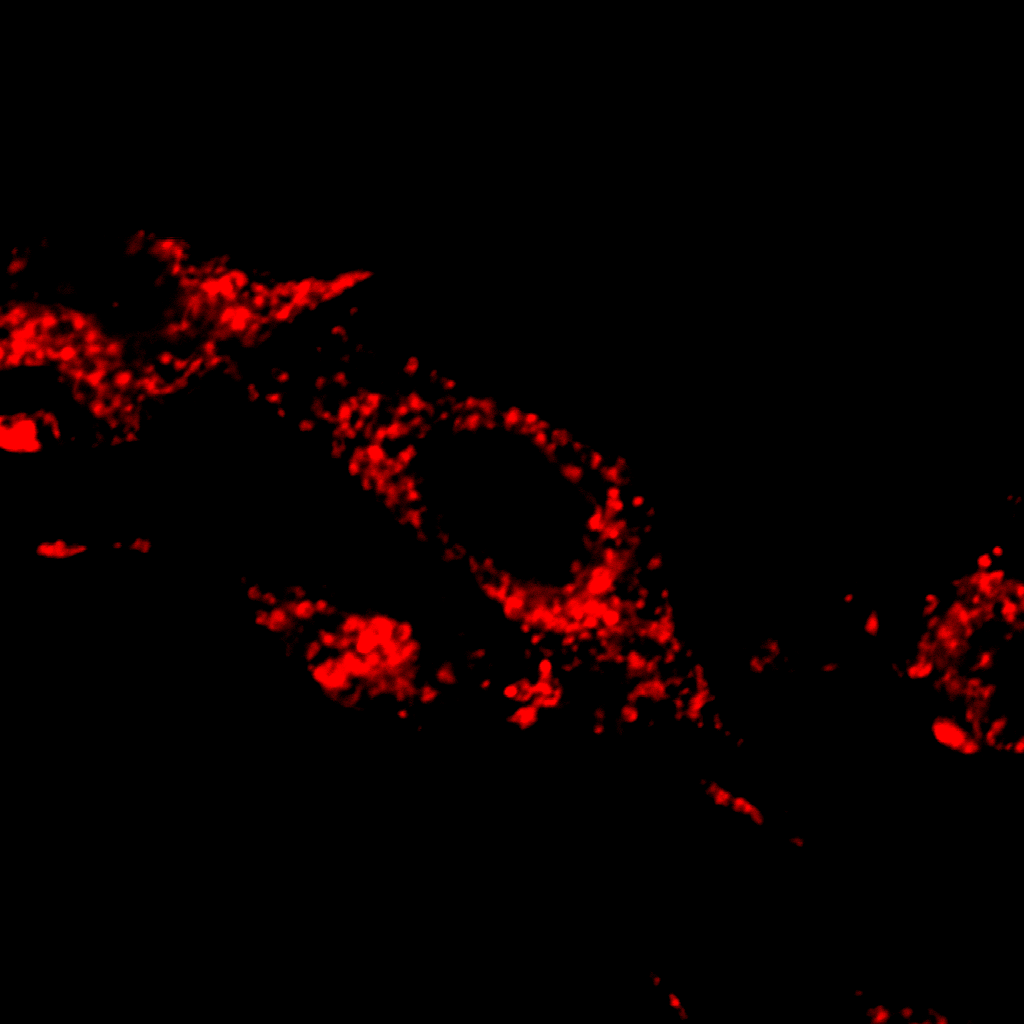

Supplement: Supplementary file 11 [file Data_Sheet_11.ZIP › hypoxia/3-6X180---All.tif]

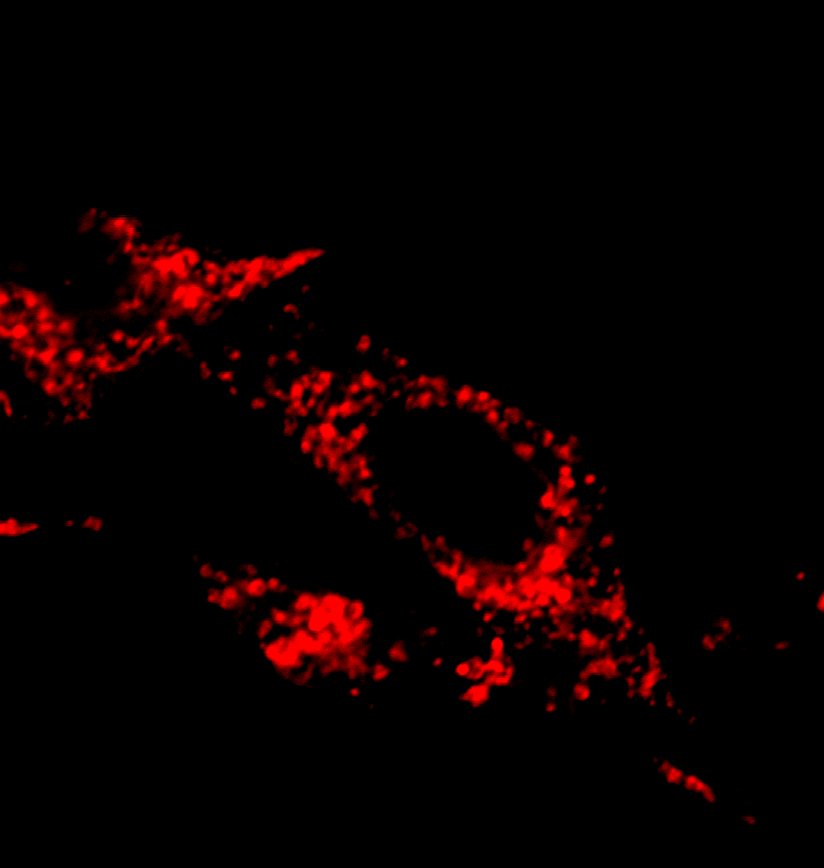

Supplement: Supplementary file 11 [file Data_Sheet_11.ZIP › hypoxia/3-6X180---montage.tif]

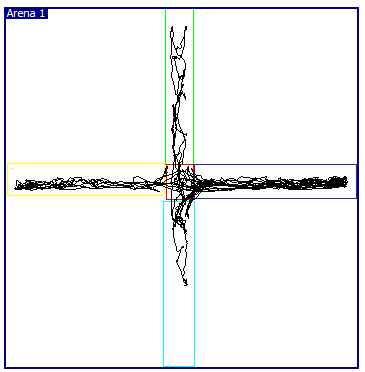

Supplement: Supplementary file 12 [file Data_Sheet_12.ZIP › Elevated Plus Maze/1.bmp]

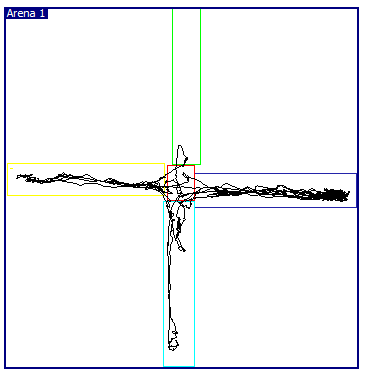

Supplement: Supplementary file 12 [file Data_Sheet_12.ZIP › Elevated Plus Maze/6.bmp]

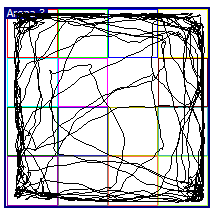

Supplement: Supplementary file 12 [file Data_Sheet_12.ZIP › open field/control.bmp]

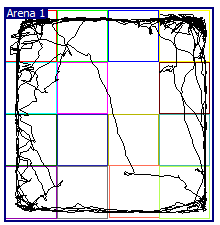

Supplement: Supplementary file 12 [file Data_Sheet_12.ZIP › open field/hypoxia.bmp]
